# COPD, PRISm and lung function reduction affect the brain cortical structure: a Mendelian randomization study

**DOI:** 10.1186/s12890-024-03150-2

**Published:** 2024-07-15

**Authors:** Chuangsen Fang, Ao Li, Yanming Li

**Affiliations:** 1 Peking University Fifth School of Clinical Medicine, Beijing, 100730 China; 2 Department of Respiratory and Critical Care Medicine, Beijing Hospital, National Center of Gerontology, Institute of Geriatric Medicine, Chinese Academy of Medical Sciences, Beijing, 100730 China

**Keywords:** Chronic obstructive pulmonary disease, Lung function, Brain cortical structural, Mendelian randomization

## Abstract

Chronic obstructive pulmonary disease (COPD) has been associated with alterations in the brain cortical structure. Nonetheless, the causality between COPD and brain cortical structure has not been determined. In the present study, we used Mendelian randomization (MR) analysis to explore the causal effects of genetic predicated COPD on brain cortical structure, namely cortical surface area (SA) and cortical thickness (TH). Genetic association summary data for COPD were obtained from the FinnGen consortium (*N* = 358,369; Ncase = 20,066). PRISm summary genetic data were retrieved from a case–control GWAS conducted in the UK Biobank (*N* = 296,282). Lung function indices, including forced expiratory volume in one second (FEV1), forced vital capacity (FVC), and FEV1/FVC, were extracted from a meta-analysis of the UK Biobank and SpiroMeta consortium (*N* = 400,102). Brain cortical structure data were obtained from the ENIGMA consortium (*N* = 51,665). Inverse-variance weighted (IVW) method was used as the primary analysis, and a series of sensitivity tests were exploited to evaluate the heterogeneity and pleiotropy of our results. The results identified potential causal effects of COPD on several brain cortical specifications, including pars orbitalis, cuneus and inferior parietal gyrus. Furthermore, genetic predicated lung function index (FEV1, FVC and FEV1/FVC), as well as PRISm, also has causal effects on brain cortical structure. According to our results, a total of 15 functional specifications were influenced by lung function index and PRISm. These findings contribute to understanding the causal effects of COPD and lung function to brain cortical structure.

**Supplementary Information:**

The online version contains supplementary material available at 10.1186/s12890-024-03150-2.

## Introduction

Chronic obstructive pulmonary disease (COPD) is a significant global health issue. It is the third leading cause of death worldwide, causing 3.23 million deaths in 2019 [1]. In 2019, there were 212.3 million prevalent cases of COPD globally [2, 3]. The disability-adjusted life years (DALYs) rates for COPD was 74.4 million respectively [2].Pre-COPD is a concept that refers to individuals who do not meet the spirometric criteria for the diagnosis of COPD but exhibit significant lung pathology and respiratory symptoms and a study reported that the age-standardized prevalences of pre-COPD was 7.2% (95%CI 5.9%-8.8%) [4–7]. The prevalence of predicated preserved ratio impaired (PRISm) in the general population ranges from around 5–20% across different studies [8–10], and it tends to be higher in smokers and middle-aged adults [11, 12]. PRISm is linked to higher risks of developing COPD, cardiovascular disease, and all-cause mortality compared to normal spirometry [13, 14].

COPD has been associated with alterations in the brain's cortical structure. These alterations include changes in both gray matter (GM) and white matter (WM) regions of the brain. Gray matter reductions have been observed in several brain regions in COPD patients, and these reductions are associated with disease severity [15, 16]. The affected regions are mainly confined to the limbic or paralimbic structures and frontal cortices [15]. In particular, the prefrontal networks of COPD patients show abnormal activation [17]. A study found a 1.1% decrement in normalized gray matter volume associated with COPD, which is greater than the annual rate of gray matter volume loss typically seen in aging [18]. White matter alterations have also been reported in COPD patients. Diffuse injury was found in white matter in patients with stable COPD [17]. There is reduced white matter integrity throughout the brain in stable non-hypoxemic COPD patients [19]. On the other hand, several population-based studies have found an association between PRISm and increased risk of incident dementia and cognitive impairment [20]. Cortical thickness and surface area are also affected in COPD patients. Reduced cortical thickness has been observed broadly in motor, parietal, and prefrontal cortices [21]. However, most of the previous studies have described these phenomena and there is insufficient evidence for a causal relationship between COPD and cortical structural changes. In this study, we aimed to investigate the causal effects of genetically predicted COPD and lung function indices on brain cortical structure using Mendelian randomization (MR) analysis. Additionally, we explored whether poor lung function in individuals not diagnosed with COPD, specifically preserved ratio impaired spirometry (PRISm), also affects brain cortical structure.

## Method

**Fig. 1:**
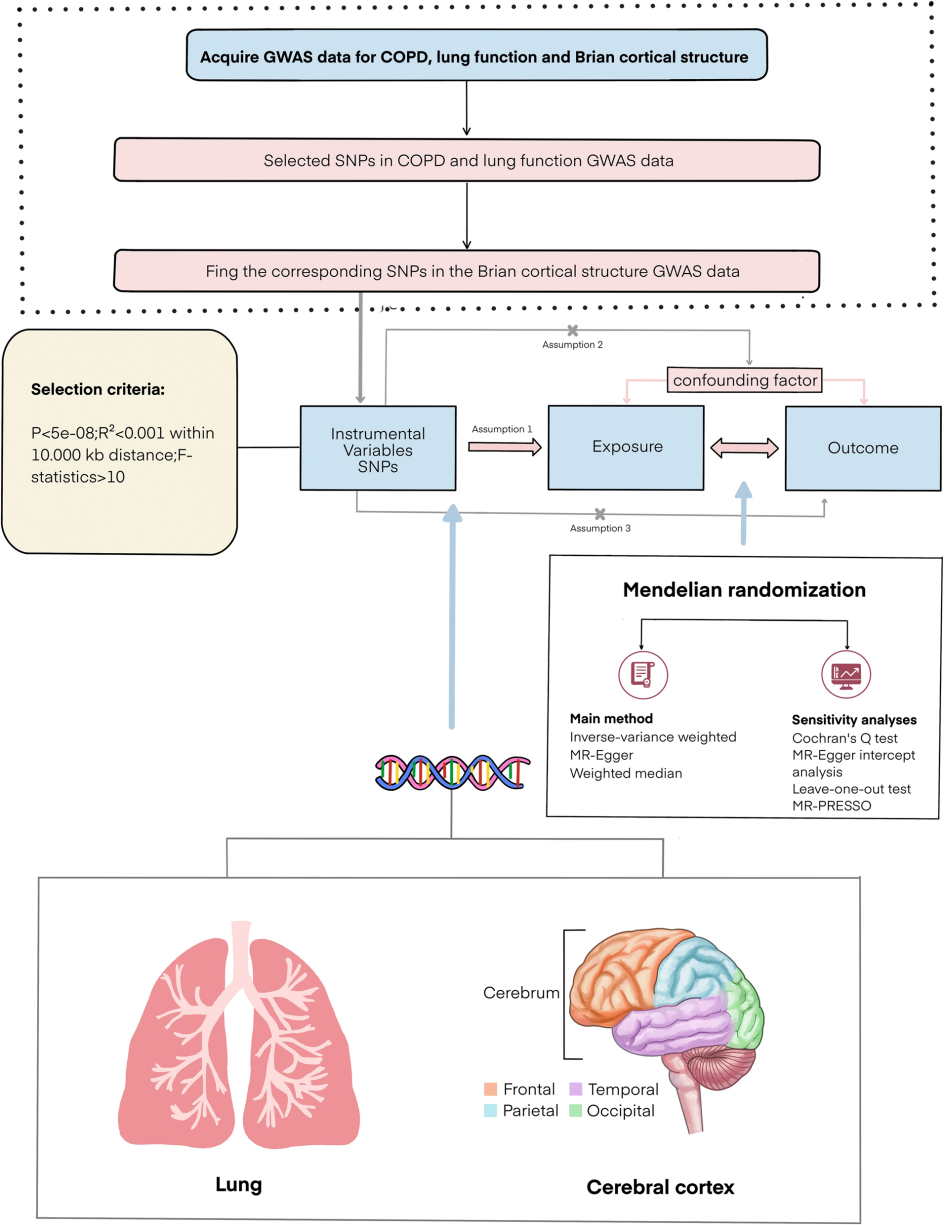
Overview of study design. We explorethe causality association between COPD, lung function and brain cortical structure using MR analysis. Assumption 1: relevance assumption, genetic variants significantly associate with the exposure trait; Assumption 2: genetic variants must be independent of any confounders that influence the association between exposure and outcome; Assumption 3: genetic variants can affect outcome only though exposure, instead of any alternative pathways;

In this study, we designed a Mendelian randomization study to explore the causal relationship between COPD, PRISm, lung function, and cortical structure. The technical roadmap is shown in Fig. 1 and the Data source of genetic variables used for MR analysis in Table 1. Mendelian randomization (MR) is a useful tool to identify the causal effect of the exposure on the outcome [22]. MR uses single nucleotide polymorphisms (SNPs) as instrumental variables and relies on equally, randomly, and independently distributed genetic variants during meiosis, which effectively avoids the influence of confounding and reverse causes [23]. We utilized human genetic data within the MR framework to reveal the effect of COPD and PRISm on the brain cortex structure, defined as human brain cortical surface area (SA) and cortical thickness (TH), as detected using MRI. COPD, PRISm and lung function GWAS data were used to provide the MR estimates.

**Table 1:** Data source of genetic variables used for MR analysis

Phenotype	Type of trait	Consortium	Sample size	No. case	PMID
COPD	Binary	FinnGen	358,369	20,066	NA
FEV1	Continuous	UK Biobank; SpiroMeta	400,102	NA	30,804,560
FVC	Continuous	UK Biobank; SpiroMeta	400,102	NA	30,804,560
FEV1/FVC	Continuous	UK Biobank; SpiroMeta	400,102	NA	30,804,560
Preserved ratio impaired spirometry (PRISm)	Binary	UK Biobank;	296,282	38,639	38,097,206
Brian cortex surface area	Continuous	ENIGMA	51,665	NA	32,193,296
Brian cortex thickness	Continuous	ENIGMA	51,665	NA	32,193,296

*COPD* Chronic obstructive pulmonary diseases, *FEV1* Forced expiratory volume in 1 s, *FVC* Forced vital capacity

### Data source

#### COPD and PRISm

FinnGen is a nationwide repository of data on genetic factors constructed in Finland. We retrieved genetic association summary data for COPD from the latest R10 data release (*N* = 358,369; Ncase = 20,066, Ncontrols = 338,303). COPD was diagnosed according to the International Classification of Disease (ICD) codes.

PRISm is diagnosed with FEV1 < 80% predicted and FEV1/FVC ratio ≥ 0.70. We retrieved summary genetic data of PRISm from a case–control GWAS conducted in UK biobank, with sample size 296,282 people. The GWAS was adjusted for sex, body mass index, age and smoking status [24].

#### Lung function

Genetic association summary data of lung function index, namely forced expiratory volume in one second (FEV1), forced vital capacity (FVC) and FEV1/FVC were extracted from a meta-analysis of the UK Biobank (*N* = 400,102) and the SpiroMeta consortium (*N* = 79,055) [25]. The GWAS was adjusted for age, age^2^, height and smoking status. The UK Biobank is the largest repository of data on genetic and environmental factors constructed in the United Kingdom between 2006 and 2010. There were 321,047 participantsrecruited from UK Biobank. The SpiroMeta consortium recruited over 79,000 participants from 22 cohorts of European ancestry. Assessment of lung function in the UK Biobank was conducted by medical care personnel using a Vitalograph Pneumotrac 6800 spirometer and the assessment of lung function in SpiroMeta consortium varies from different studies.

#### Brain cortical structure

Summary data of brain cortical structure were obtained from the ENIGMA Consortium [26]. There were 51,665 participants across 60 cohorts were recruited in this study and the brain cortical surface area and thickness were scanned with T1-weighted magnetic resonance imaging. Only European-ancestry participants were included to conduct MR analysis. The 34 functional specification of brain cortex were roughly divided according to the Desikan-Killiany atlas and the regional boundaries were determined by gyral anatomy labelled from between the depths of the sulci [27]. We here conducted MR study from COPD and lung function to the global measures (total surface area and average thickness), as well as SA and TH of 34 brain cortical regions with or without the global weighted estimates of the entire brain. Genetic data with global control means that the SA and TH of specific regions across the SA and TH of entire brain cortex, while genetic data without global control means that the SA and TH of specific regions regardless of the whole brain SA and TH.

### Instrument variable selection

Genetic variants which meet the following criteria were selected as instrumental variables: (1) Genetic variants significantly correlated with exposure trait (*P* < 5e-08). AS only limited SNPs were selected for PRISm in this context, we loosen the criteria to 5e-06 for PRISm(2) Weak genetic instruments with *F* < 10 were removed. (3) Linkage disequilibrium (LD) was excluded by clumping procedure with R2 = 0.001 within a clumping distance of 10,000 kb. European samples from 1000 Genome Project were used as reference. (4) Palindromic SNPs were removed after harmonizing exposure and outcome data. (5) Genetic variants do not significantly associate with outcome data. (6) we remove outliers that detected by MRPRESSO and re-conduct MR analysis. Summary data of SNPs that are used as genetic instruments for exposure trait can be found in Tables S1-S5.

Generally, there were 16, 27, 253, 225, and 284 genetic variants selected as instrumental variables for COPD, PRISm, FEV1, FVC, and FEV1/FVC, respectively. These selected genetic variants were regarded as strong instrumental variables as F was larger than 10. MR analysis was conducted to explore the causal effects of COPD and lung function on global SA/TH as well as 34 functional regions with and without global control.

### Mendelian randomization analysis

We conducted a comprehensive MR analysis in determining the causal effects of COPD, PRISm and lung function to global brain cortical SA/TH as well as 34 functional regions (with or without global controls). Three different methods were employed, namely inverse variance weighted (IVW), weighted median and MR-Egger. IVW was determined as the primary outcome as it provides the most robustness and precise estimates when all genetic variants meet three MR assumptions. Although being less efficient, weighted median and MR-Egger were conducted to complement IVW estimates as they offer more robustness estimates in a broader context (wider confidence interval) [28, 29]. MR estimates are reported as beta as outcome data are continuous traits.

For significant results, we used IVW Cochran’s Q test to assess heterogeneity of estimates. Pleiotropy was determined by MR-Egger intercept analysis. Heterogeneity and pleiotropy verify the robustness of MR estimates by evaluating the differentiation of instrumental variable variations and the specificity of causal direction. Moreover, MRPRESSO also were implemented to test outliers [30]. If outliers were detected by MRPRESSO, we removed it and re-conduct the MR analysis. We also conducted leave-one-out analysis to evaluate the influence of single SNP on the overall MR estimates. A funnel plot was exploited to illustrate directional pleiotropy.

Analyses were conducted with the R package “TwoSampleMR” [31] (version.4.25) and “MendelianRandomization” [32] in R (version 3.6.1).

## Results

**Fig. 2:**
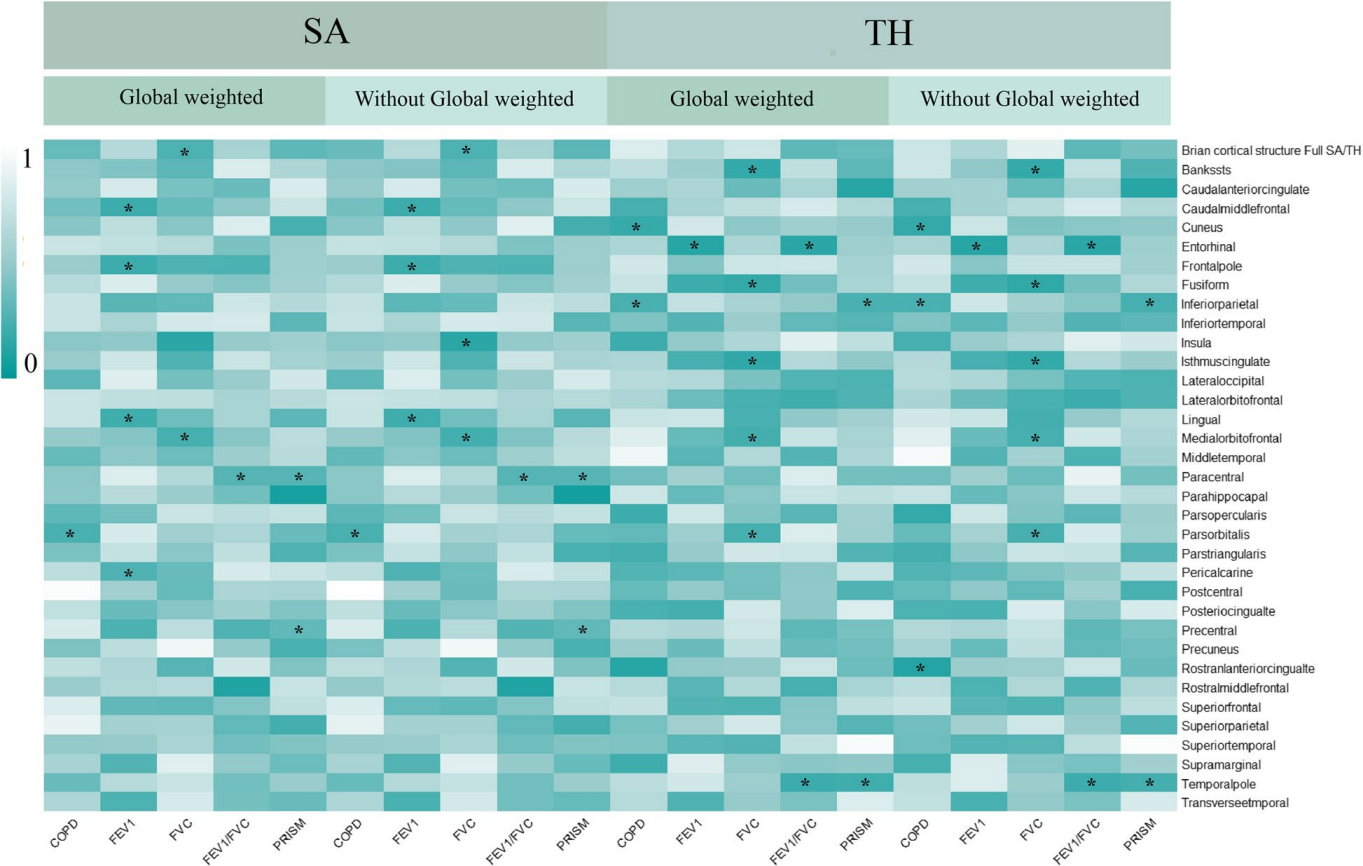
Heatmap of the causal effects of COPD, PRISm and lung function index on brain cortical structure. The nominally significant *P*-value were shown in the figure with *. COPD, chronic obstructive pulmonary diseases; FEV1: forced expiratory volume in 1 s; FVC: forced vital capacity; PRISm: preserved ratio impaired spirometry; SA: surface area; TH: thickness

**Fig. 3:**
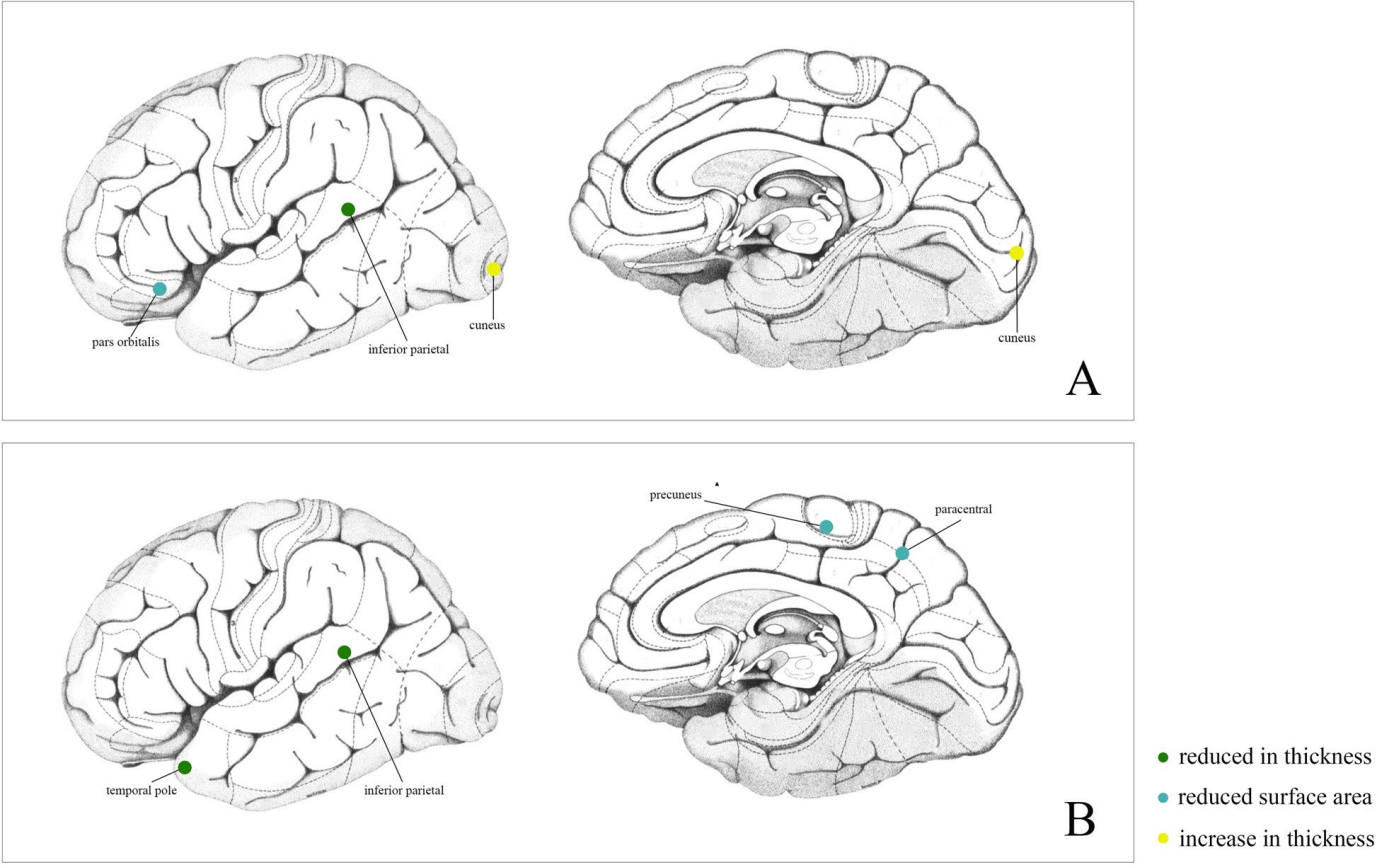
**A** The relationship between genetic predicated cortical surface area and thickness and genetic predicated COPD; **B** The relationship between genetic predicated cortical surface area and thickness and genetic predicated PRISm

**Fig. 4:**
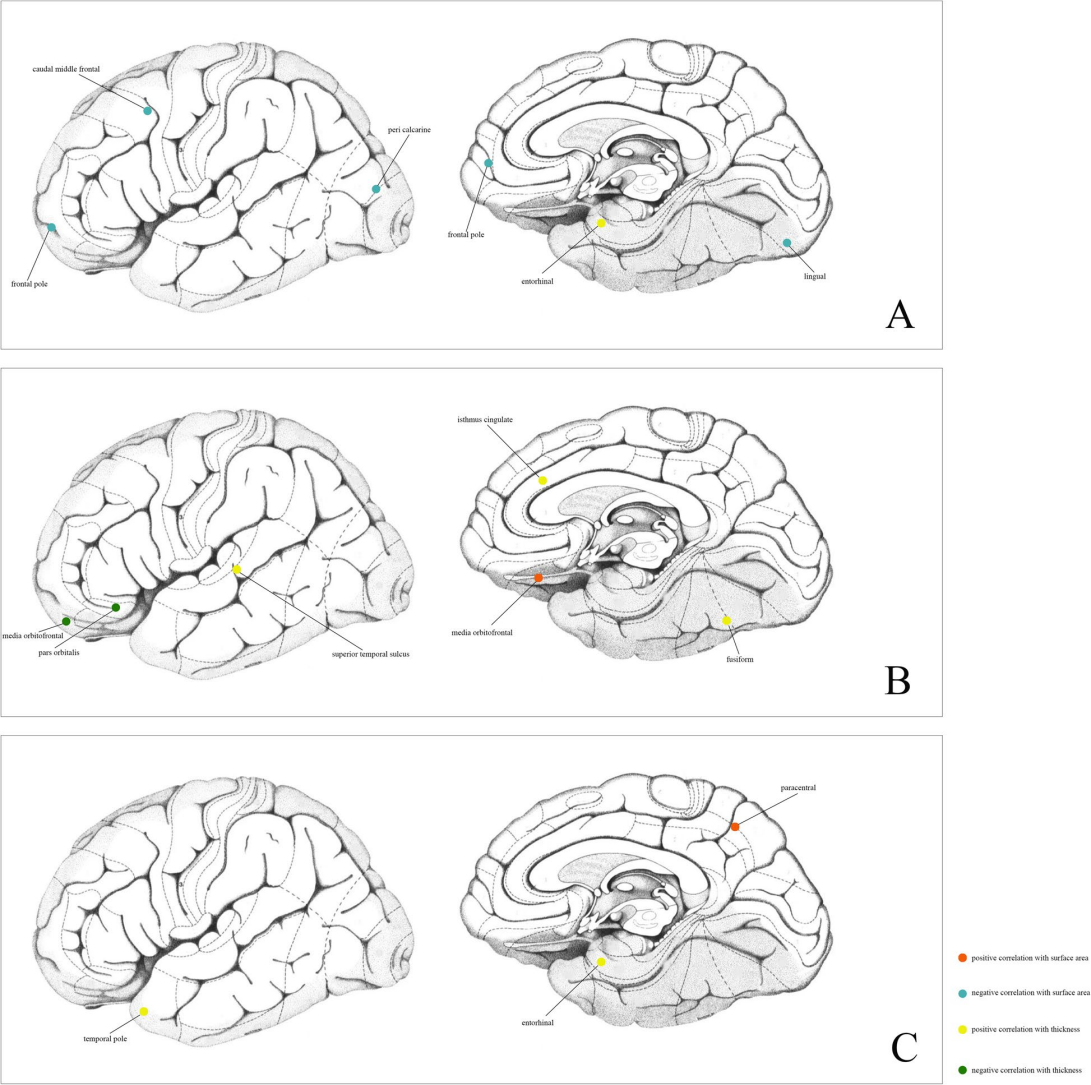
**A** The relationship between genetic predicated cortical surface area and thickness and genetic predicated FEV1; **B** The relationship between genetic predicated cortical surface area and thickness and genetic predicated FVC; **C** The relationship between genetic predicated cortical surface area and thickness and genetic predicated FEV1/FVC

Overall, we have found a causal relationship between COPD, PRISm and structural changes in certain cortical regions, including SA and TH, as well as a correlation between lung function indicators and cortical structures (Figs. 2, 3 and 4).

### Causal effects of COPD to brain cortical structure

A total of 16 SNPs were chosen as instrumental variables of COPD. MR analysis indicated that genetic predicated COPD decreased the SA of pars orbitalis with (βgc = -3.351, 95%CI = -6.468 ~ -0.234, *P* = 0.035) and without global control (βnon-gc = -3.338,95%CI = -6.456 ~ -0.223, *P* = 0.036). No heterogeneity was examined with IVW Cochran’s Q test (Q = 15.469, *P* = 0.418). MR-Egger intercept analysis and MRPRESSO do not tested pleiotropy (Intercept = 0.338, *P* = 0.299; MRPRESSO global *P* = 0.408). No outliers were identified with MRPRESSO and leave-one-out analysis as well as funnel plot. Besides, genetic predicated COPD was found to negatively associated with the thickness of inferior parietal gyrus (βgc = -0.005, 95%CI = -0.009 ~ -0.001, *P* = 0.014), while positive causal association was determined between COPD and cuneus gyrus (βgc = 0.006, 95%CI = 0.0003 ~ 0.012, *P* = 0.039). Similar results were also observed in MR analysis between COPD and functional regions without global weighted, except for the significant causal effects from COPD to rostral anterior cingulate gyrus (βnon-gc = -0.009, 95%CI = -0.018 ~ -0.0001, *P* = 0.048).

### Causal effects of PRISm to brain cortical structure

PRISm, also referred as a precursor of COPD, has been detected to causally affect brain cortex regions. Specifically, genetic predicted PRISm was negatively associated with SA of paracentral gyrus (βgc = -99.274, 95%CI: -178.125 ~ -20.422, *P* = 0.014) whereas positively associated with SA of precuneus gyrus (βgc = 268.312, 95%CI: 119.437 ~ 417.187, *P* < 0.001). Similar results were presented in cortical SA genetic data without global control. On the other hand, genetic predicted PRISm had causal effects on TH of several cortical regions. It negatively associated with TH of inferiorparietal gyrus (βgc = -0.049, 95%CI: -0.089 ~ -0.009, *P* = 0.016) and temporapole gyrus (βgc = -0.150, 95%CI: -0.293 ~ -0.007, *P* = 0.014). In these results, heterogeneity was not examined with IVW Cocran’s Q test, MRPRESSO global p test and funnel plot as well as leave-one-out analysis. Besides, MR-Egger intercept analysis determined that no pleiotropy existed.

### Causal effects of lung function to brain cortical structure

At the global level, genetic predicated FVC was positively associated with global surficial area (βgc = 1576.610, 95%CI:416.457 ~ 2736.762, *P* = 0.008) but no significant effects on global cortical thickness were discovered. Heterogeneity was indicated by IVW Cochran’s Q test (Q = 426.058, *P* < 0.001). No horizontal pleiotropy was discovered with MR-Egger intercept analysis (Intercept = -64.126, *P* = 0.113). MRPRESSO and LOOA identified outliers and MRPRESSO provided estimates after removing them (βgc = 1224.838, 95%CI:167.626 ~ 2282.050, *P* = 0.024). However, genetic predicated FEV1 and FEV1/FVC did not have significant effects on global brain cortical surficial area and thickness.

**Fig. 5:**
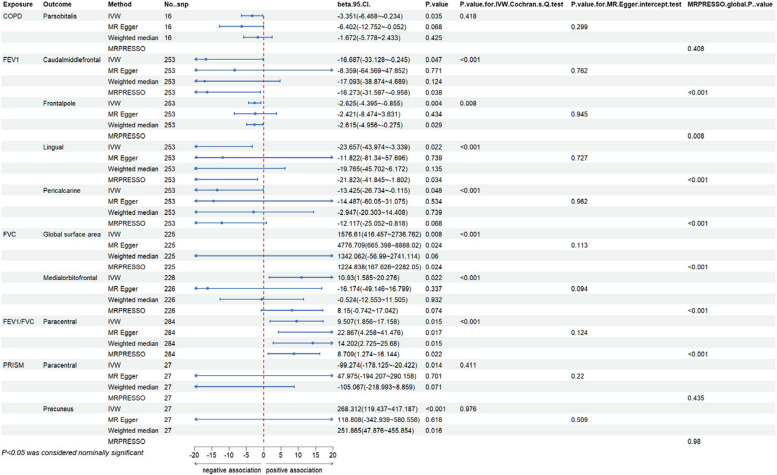
MR estimates of COPD, PRISm and lung function to brain cortical SA with global control

**Fig. 6:**
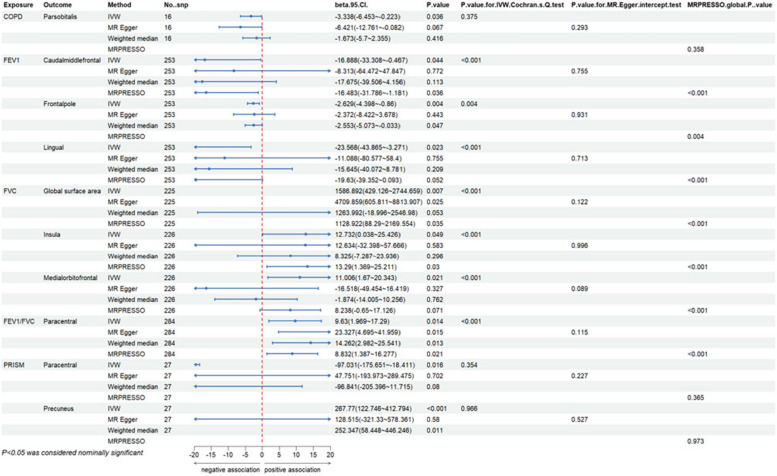
MR estimates of COPD, PRISm and lung function to brain cortical SA without global control

**Fig. 7:**
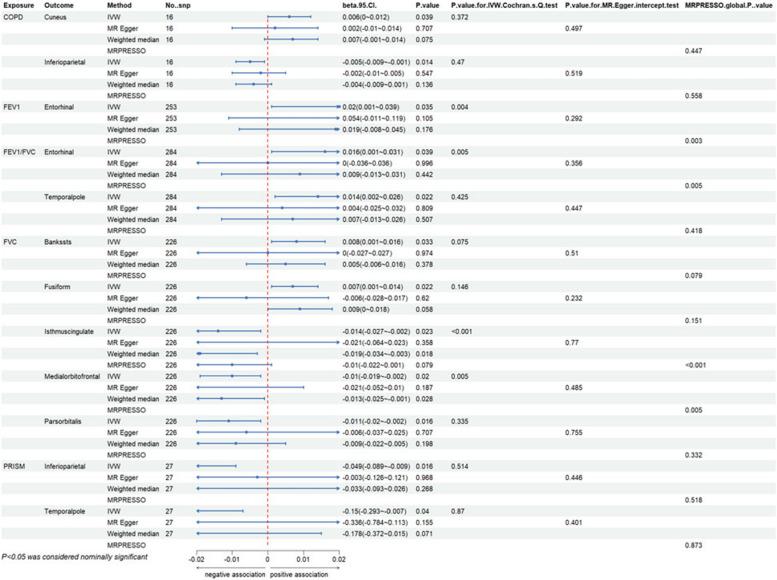
MR estimates of COPD, PRISm and lung function to brain cortical TH with global control

**Fig. 8:**
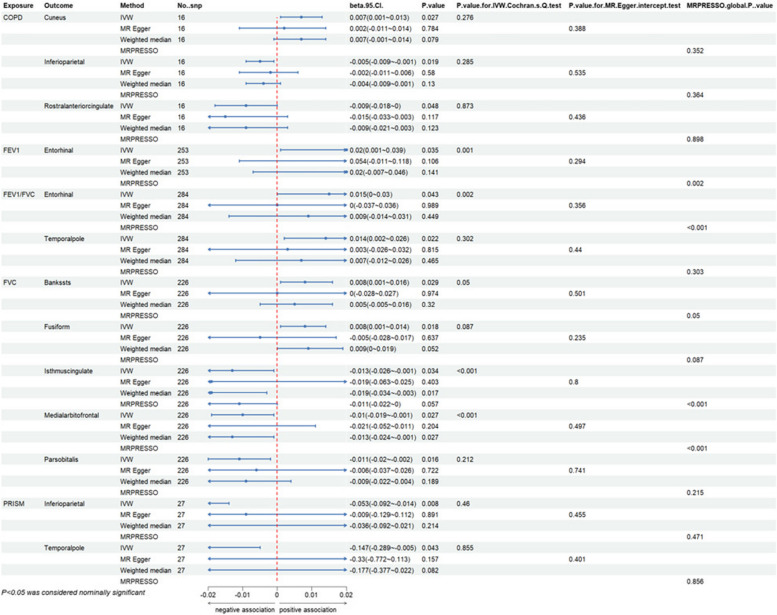
MR estimates of COPD, PRISm and lung function to brain cortical TH without global control

At the functional region levels, we performed MR analysis between lung function index and 34 functional specifications. We discovered that three different lung function index influence 12 functional regions with global weighted genetic data. Specifically, FEV1 had significantly causal effects on the SA of caudal middle frontal (βgc = -16.687, 95%CI: -33.128 ~ -0.245, *P* = 0.047), frontal pole (βgc = -2.625, 95%CI: -4.395 ~ -0.855, *P* = 0.004), lingual (βgc = -23.657, 95%CI: -43.974 ~ -3.339, *P* = 0.022) and pericalcarine gyrus (βgc = -13.425, 95%CI: -226.734 ~ -0.115, *P* = 0.048). Heterogeneity was found in all these results with IVW Cochran’ Q test, whereas pleiotropy was not detected. Notably, the causal effects of FEV1 on SA of pericalcarine gyrus turns to marginally significant in results without global weighted (βnon-gc = -13.315, 95%CI: -26.654 ~ 0.024, *P* = 0.050). Besides, genetic predicated FEV1 positively associated with the thickness of entorhinal area in both global weighted (βgc = 0.020, 95%CI: 0.001 ~ 0.039, *P* = 0.035) and non-global weighted genetic data. There were also several suggestive regions potentially affected by two other measures of lung function, comprising medial orbitofrontal, insula, the bank of the superior temporal sulcus (BANKSSTS), fusiform, isthmus of cingulate, pars orbitalis, paracentral, entorhinal and temporal pole gyrus (Figs. 5, 6, 7 and 8).

Sensitivity test for heterogeneity and pleiotropy, including IVW Cochran’s Q test, MR-Egger intercept test and MRPRESSO were exploited for all MR estimates. Although heterogeneity was identified in several results, it is acceptable as we used random-effects IVW [33]. No pleiotropy was detected by MR-Egger intercept analysis. Forest plot, Scatter plot, funnel plot and leave-one-out analysis plot of significant results were provided in Supplementary Figures.

## Discussion

This Mendelian randomization study provides new insights into the potential causal relationship between COPD and cortical structural changes. Previous studies have found that COPD patients may have a higher risk of cognitive impairment and neurological complications [34, 35]. Our research demonstrates that COPD can casually affects the surface area or thickness of specific areas of cerebral cortex. Additionally, we further explored the relationship between PRISM, lung function, and cortical structure, and found that cortical structure changes may occur in the early stages of the COPD, as well as in the compromised lung function.

COPD has been associated with cerebral cortical changes, as revealed by previous studies. These changes are not characterized by generalized cortical degeneration, but rather specific alterations in gray matter and functional activity in certain brain regions. Structural and functional changes in the brain are believed to be related to the cognitive impairments often observed in COPD patients. To elucidate the causal relationship between COPD and cerebral cortical structure, we performed this MR study to analyze COPD and lung function index including FEV1, FVC, and FEV1/FVC, and their association with cerebral cortical structure.

In the present study, we identified potential causal effects of COPD on several brain cortical specifications, including pars orbitalis, cuneus and inferior parietal gyrus. To further decipher the lung-brain axis, we used genetic data of lung function index, FEV1, FVC and FEV1/FVC as instrumental variables and conducted MR analysis with genetic data of brain cortical structure. According to our results, a total of 15 functional specifications were influenced by lung function index (Figs. 5–8). Noteworthy, several estimates may deviate from logical expectation. Normally, higher FEV1 and FVC indicated optimized lung function hence should result in larger SA and TH. Nonetheless, our results revealed that genetically predicted FEV1 was negatively associated with the SA of the caudal middle frontal, frontal pole, lingual, and pericalcarine gyri, and genetically predicted FVC was negatively associated with the isthmus cingulate, medial orbital frontal, and pars orbitalis gyri. These results all passed sensitivity tests and were considered robust.

The results of Mendelian randomization studies on COPD and cortical structures suggest that the decrease in surface area of the pars orbitalis may be associated with COPD. The pars orbitalis, a component of the inferior frontal gyrus, is involved in several key brain functions. It helps integrate semantic and emotional processing, facilitates recognition of facial expressions, and contributes to inhibitory control, which is crucial for regulating impulsive behavior [36, 37]. Additionally, it plays a role in the interaction between working memory and semantic processing, influencing how information is stored and retrieved [38]. Reductions in the surface area of pars orbitalis are linked to neurodevelopmental and neurodegenerative processes. In children with internalizing and externalizing disorders, the pars orbitalis shows significant reduction, indicating a potential vulnerability to psychiatric disorders [39]. Additionally, longitudinal studies suggest that normal aging involves decelerated volume changes [40]. The inferior parietal lobule (IPL) within the brain's parietal lobe is vital for various neural functions, including attention, sensory integration, language, and social cognition [41, 42]. Reduced cortical thickness in the IPL is associated with conditions like high myopia and obsessive–compulsive disorder (OCD), suggesting potential neurological or psychiatric implications [43]. However, these associations do not imply causation, and further research is needed to understand the significance of IPL thickness reduction across different contexts.

Currently, there is no direct evidence to establish that PRISm leads to neurological complications such as cognitive impairment. However, some studies suggest that PRISm may increase the risk of developing dementia [44]. To further elucidate this causal relationship, we conducted a Mendelian randomization study on PRISm and cortical structure. Our results indicate that PRISm is associated with a reduction in the surface area of the paracentral lobule and precuneus, as well as a decrease in the thickness of the inferior parietal lobule and temporal poles. The physiological functions of these brain regions encompass a variety of roles, including vision, memory, language, and emotion [45]. This suggests that neural damage may commence in the early stages of COPD, and that examining specific cortical structures could aid in early diagnosis.

In this study, we also investigated the causality association between genetic predicated lung function and brain cortical structure, and identified several functional regions that were influenced by lung function. There is evidence to suggest that poor lung function is associated with cognitive decline and changes in brain structure [17, 46, 47]. A study within the Rush Memory and Aging Project found that low lung function was related to faster decline in global cognition and domain-specific functions, including episodic memory, semantic memory, working memory, visuospatial ability, and perceptual speed [46]. Another study indicated that poor lung function is related to impaired cognition and adverse findings on brain imaging [48]. COPD patients exhibit progressive structural impairments, which correlate with levels of lung function impairment and cognitive deficits. This suggests that persistent reductions in lung function may lead to atrophy in specific brain regions, ultimately resulting in cognitive impairment [17]. Although there is no direct evidence, pulmonary hypoplasia in pre-COPD and even non-COPD patients may also cause structural and functional changes in the cerebral cortex.

Our study found that COPD and decreased lung function may lead to an increase in the surface area and thickness of certain brain functional areas. These changes may be caused by various factors such as chronic hypoxia, mitochondrial dysfunction, and alterations in neurovascular coupling. Chronic hypoxia can trigger compensatory mechanisms, including angiogenesis, which may increase cortical surface area [17, 49]. Additionally, oxidative stress in COPD may lead to mitochondrial dysfunction in brain cells, resulting in cell swelling [50, 51]. Furthermore, studies have shown that the relationship between neural activity and cerebral blood flow is disrupted in COPD patients, and this abnormal neurovascular coupling may contribute to cortical changes [49].

To the best of our knowledge, this is the first study that investigate the causal effects of COPD and lung function to brain cortical structure by using MR analysis. However, there were some limitations in this study. First, the results can only be employed in European ancestry instead of other populations. Secondly, our findings only provided the causal effects of COPD and lung function index to brain cortical regions, and due to the paucity of relevant studies, the underlying mechanisms of brain cortical structure changes caused by COPD and lung function index remains unknown. Finally, the primary goal of MR is to examine the causal association effects of genetic predicated COPD, lung function index to brain cortical structure, hence we can not conclude the size of estimates effects.

## Conclusion

This Mendelian randomization study establishes a causal relationship between COPD, PRISm, lung function indices, and changes in brain cortical structure. Significant associations were observed between COPD and structural alterations in specific cortical regions, such as the pars orbitalis, cuneus, and inferior parietal gyrus. PRISm, as a precursor to COPD, also affects cortical surface area and thickness, underscoring the importance of early detection and management. Furthermore, we have detected compromised lung function, as indicated by FEV1 and FVC, is linked to structural brain changes, elucidating the neuropsychological impairments observed in COPD patients. These findings imply that monitoring cortical changes could be useful for assessing neurological complications in COPD patients.

Future research should investigate the underlying mechanisms and potential therapeutic interventions. Despite the robust MR analysis, this study has limitations, including its focus on individuals of European ancestry and the inability to quantify effect sizes. Further studies are required to validate these findings in diverse populations and to understand the broader implications of lung function on brain health, ultimately aiming to improve the quality of life for those with COPD and related conditions.

### Supplementary Information

Supplementary Material 1.

Supplementary Material 2.

Supplementary Material 3.

Supplementary Material 4.

Supplementary Material 5.

Supplementary Material 6.

Supplementary Material 7.

Supplementary Material 8.

Supplementary Material 9.

Supplementary Material 10.

Supplementary Material 11.

Supplementary Material 12.

## Data Availability

The datasets analyzed during the current study are publicly available. COPD: Finland https://www.finngen.fi/en/access_results: PRISM: https://gwas.mrcieu.ac.uk/datasets/ieu-b-5112/； Lung function: https://www.ebi.ac.uk/gwas/studies/GCST007429； https://www.ebi.ac.uk/gwas/studies/GCST007431；https://www.ebi.ac.uk/gwas/studies/GCST007432；
