# Supplementary material for: COPD, PRISm and lung function reduction affect the brain cortical structure: a Mendelian randomization study
Source: BMC Pulm Med. 2024 Jul 15;24:341. doi: 10.1186/s12890-024-03150-2 (PMC11251327; doi:10.1186/s12890-024-03150-2)
Supplement: Supplementary file 1 — Supplementary Material 1. [file 12890_2024_3150_MOESM1_ESM.docx]

|  | SNP | effect_allele.exposure | other_allele.exposure | beta.exposure | eaf.exposure | pval.exposure | se.exposure | samplesize | F |
| --- | --- | --- | --- | --- | --- | --- | --- | --- | --- |
| 1 | rs12501071 | T | C | -0.0599897 | 0.518755 | 2.38E-08 | 0.0107471 | 358,369 | 31.1580881 |
| 2 | rs13270042 | G | A | 0.0957491 | 0.108197 | 1.60E-08 | 0.0169441 | 358,369 | 31.9324609 |
| 3 | rs141669463 | T | C | -0.182227 | 0.0302738 | 2.49E-08 | 0.0326905 | 358,369 | 31.0729384 |
| 4 | rs16969968 | A | G | 0.203335 | 0.330349 | 6.02E-74 | 0.0111774 | 358,369 | 330.934995 |
| 5 | rs28406364 | T | C | 0.0611286 | 0.42894 | 1.68E-08 | 0.010835 | 358,369 | 31.8295917 |
| 6 | rs2855655 | C | T | 0.0626758 | 0.658388 | 3.43E-08 | 0.0113584 | 358,369 | 30.4484686 |
| 7 | rs28929474 | T | C | 0.331723 | 0.0197907 | 1.59E-20 | 0.0357199 | 358,369 | 86.2443592 |
| 8 | rs56223946 | T | C | -0.116542 | 0.0999858 | 2.81E-10 | 0.0184728 | 358,369 | 39.8015034 |
| 9 | rs6011779 | T | C | -0.094956 | 0.755669 | 1.25E-14 | 0.0123147 | 358,369 | 59.4562003 |
| 10 | rs60249782 | A | G | 0.0606246 | 0.376841 | 4.58E-08 | 0.0110894 | 358,369 | 29.8869553 |
| 11 | rs60892124 | T | C | -0.102612 | 0.0998195 | 2.56E-08 | 0.0184242 | 358,369 | 31.0183739 |
| 12 | rs708461 | C | G | 0.0785043 | 0.205551 | 1.98E-09 | 0.0130848 | 358,369 | 35.9958732 |
| 13 | rs7671167 | T | C | 0.0603276 | 0.504635 | 1.90E-08 | 0.0107322 | 358,369 | 31.5976353 |
| 14 | rs7676321 | A | G | 0.0663966 | 0.716697 | 3.13E-08 | 0.0119985 | 358,369 | 30.6222974 |
| 15 | rs7783505 | G | C | -0.062488 | 0.372162 | 2.43E-08 | 0.0112022 | 358,369 | 31.1162037 |
| 16 | rs8089390 | T | C | -0.0710206 | 0.276642 | 3.81E-09 | 0.0120533 | 358,369 | 34.7181633 |

Table S1Selected genetic instrumental variables of COPD
