# Supplementary material for: COPD, PRISm and lung function reduction affect the brain cortical structure: a Mendelian randomization study
Source: BMC Pulm Med. 2024 Jul 15;24:341. doi: 10.1186/s12890-024-03150-2 (PMC11251327; doi:10.1186/s12890-024-03150-2)
Supplement: Supplementary file 2 — Supplementary Material 2. [file 12890_2024_3150_MOESM2_ESM.docx]

|  | SNP | effect_allele.exposure | other_allele.exposure | beta.exposure | eaf.exposure | se.exposure | pval.exposure | samplesize | F |
| --- | --- | --- | --- | --- | --- | --- | --- | --- | --- |
| 1 | rs10005540 | T | C | 0.0144 | 0.6145 | 0.0024 | 3.08E-09 | 400,102 | 36 |
| 2 | rs10059996 | T | G | -0.0139 | 0.3548 | 0.0025 | 3.73E-08 | 400,102 | 30.9136 |
| 3 | rs1008833 | A | G | -0.0287 | 0.8541 | 0.0033 | 4.55E-18 | 400,102 | 75.63728 |
| 4 | rs10125279 | A | G | 0.0139 | 0.3844 | 0.0024 | 6.84E-09 | 400,102 | 33.5434 |
| 5 | rs10141786 | A | G | 0.0182 | 0.4017 | 0.0024 | 3.05E-14 | 400,102 | 57.50694 |
| 6 | rs10193804 | C | G | -0.0138 | 0.3478 | 0.0025 | 1.91E-08 | 400,102 | 30.4704 |
| 7 | rs10273455 | A | C | -0.0149 | 0.5518 | 0.0023 | 1.89E-10 | 400,102 | 41.96786 |
| 8 | rs10444582 | A | C | 0.0159 | 0.6547 | 0.0025 | 1.25E-10 | 400,102 | 40.4496 |
| 9 | rs10489880 | A | G | -0.0155 | 0.7214 | 0.0026 | 2.73E-09 | 400,102 | 35.53994 |
| 10 | rs10490551 | A | T | 0.0191 | 0.2772 | 0.0026 | 1.45E-13 | 400,102 | 53.96598 |
| 11 | rs10498672 | C | G | 0.0309 | 0.8227 | 0.003 | 1.90E-24 | 400,102 | 106.09 |
| 12 | rs1054661 | T | C | -0.0139 | 0.6528 | 0.0024 | 1.09E-08 | 400,102 | 33.5434 |
| 13 | rs10771754 | A | G | 0.0158 | 0.5931 | 0.0024 | 2.00E-11 | 400,102 | 43.34028 |
| 14 | rs10773000 | T | G | -0.0139 | 0.3325 | 0.0025 | 2.63E-08 | 400,102 | 30.9136 |
| 15 | rs10781518 | A | G | 0.014 | 0.5487 | 0.0024 | 2.88E-09 | 400,102 | 34.02778 |
| 16 | rs10838435 | C | G | 0.0211 | 0.1439 | 0.0033 | 1.46E-10 | 400,102 | 40.88246 |
| 17 | rs10850377 | A | G | 0.0195 | 0.3408 | 0.0025 | 4.02E-15 | 400,102 | 60.84 |
| 18 | rs10929647 | A | C | -0.0129 | 0.5133 | 0.0023 | 2.98E-08 | 400,102 | 31.45747 |
| 19 | rs10947550 | A | G | 0.0153 | 0.5578 | 0.0023 | 5.17E-11 | 400,102 | 44.25142 |
| 20 | rs10997955 | T | C | -0.0177 | 0.5888 | 0.0024 | 1.20E-13 | 400,102 | 54.39063 |
| 21 | rs1102077 | A | C | 0.0215 | 0.7574 | 0.0027 | 4.21E-15 | 400,102 | 63.40878 |
| 22 | rs1106370 | A | G | 0.0138 | 0.4213 | 0.0024 | 4.33E-09 | 400,102 | 33.0625 |
| 23 | rs11075744 | A | G | -0.0209 | 0.5725 | 0.0024 | 9.91E-19 | 400,102 | 75.83507 |
| 24 | rs11078321 | T | C | 0.0137 | 0.4724 | 0.0023 | 4.75E-09 | 400,102 | 35.48015 |
| 25 | rs11085744 | T | C | -0.0153 | 0.5595 | 0.0024 | 7.83E-11 | 400,102 | 40.64063 |
| 26 | rs11125611 | A | T | 0.0187 | 0.6283 | 0.0024 | 1.06E-14 | 400,102 | 60.71007 |
| 27 | rs11176001 | A | C | -0.0291 | 0.1309 | 0.0035 | 4.88E-17 | 400,102 | 69.12735 |
| 28 | rs11191841 | T | C | -0.0168 | 0.4914 | 0.0023 | 6.21E-13 | 400,102 | 53.3535 |
| 29 | rs112126001 | A | T | 0.0321 | 0.9036 | 0.004 | 6.68E-16 | 400,102 | 64.40063 |
| 30 | rs11241689 | T | C | -0.0173 | 0.4388 | 0.0024 | 2.16E-13 | 400,102 | 51.96007 |
| 31 | rs113111175 | T | C | 0.022 | 0.1219 | 0.0036 | 1.11E-09 | 400,102 | 37.34568 |
| 32 | rs113707721 | C | G | 0.0336 | 0.8655 | 0.0034 | 1.70E-22 | 400,102 | 97.6609 |
| 33 | rs11621587 | C | G | 0.0343 | 0.1821 | 0.003 | 9.58E-30 | 400,102 | 130.7211 |
| 34 | rs11657029 | A | G | -0.0246 | 0.1891 | 0.003 | 1.39E-16 | 400,102 | 67.24 |
| 35 | rs116894149 | T | C | -0.0292 | 0.0653 | 0.0047 | 6.66E-10 | 400,102 | 38.59846 |
| 36 | rs11702251 | T | G | -0.0171 | 0.2232 | 0.0028 | 9.75E-10 | 400,102 | 37.29719 |
| 37 | rs117261012 | A | G | 0.0241 | 0.842 | 0.0032 | 1.01E-13 | 400,102 | 56.71973 |
| 38 | rs11739847 | A | G | -0.021 | 0.1992 | 0.0029 | 4.30E-13 | 400,102 | 52.43757 |
| 39 | rs11747434 | T | C | -0.0156 | 0.722 | 0.0026 | 2.76E-09 | 400,102 | 36 |
| 40 | rs12096239 | C | G | -0.0187 | 0.253 | 0.0027 | 2.08E-12 | 400,102 | 47.96845 |
| 41 | rs12107414 | C | G | -0.0178 | 0.1887 | 0.003 | 3.01E-09 | 400,102 | 35.20444 |
| 42 | rs12115436 | A | G | 0.0226 | 0.1671 | 0.0031 | 5.09E-13 | 400,102 | 53.1488 |
| 43 | rs12204336 | A | G | -0.014 | 0.3737 | 0.0024 | 5.72E-09 | 400,102 | 34.02778 |
| 44 | rs12212123 | T | C | 0.0263 | 0.8957 | 0.0039 | 9.42E-12 | 400,102 | 45.476 |
| 45 | rs12313454 | A | G | -0.0247 | 0.8827 | 0.0036 | 6.55E-12 | 400,102 | 47.07485 |
| 46 | rs12331869 | A | G | -0.0181 | 0.1789 | 0.0031 | 3.17E-09 | 400,102 | 34.09053 |
| 47 | rs12357270 | C | G | -0.0137 | 0.6411 | 0.0024 | 1.99E-08 | 400,102 | 32.58507 |
| 48 | rs12413039 | C | G | -0.0249 | 0.7294 | 0.0026 | 3.20E-21 | 400,102 | 91.71746 |
| 49 | rs12441675 | T | C | 0.0131 | 0.519 | 0.0023 | 2.04E-08 | 400,102 | 32.44045 |
| 50 | rs1249096 | A | G | 0.0181 | 0.5634 | 0.0024 | 2.18E-14 | 400,102 | 56.87674 |
| 51 | rs12679101 | T | C | 0.015 | 0.7471 | 0.0027 | 2.61E-08 | 400,102 | 30.8642 |
| 52 | rs12695002 | T | C | -0.0146 | 0.5887 | 0.0024 | 1.15E-09 | 400,102 | 37.00694 |
| 53 | rs12698403 | A | G | -0.0274 | 0.4418 | 0.0024 | 6.42E-31 | 400,102 | 130.3403 |
| 54 | rs12707691 | C | G | -0.0205 | 0.6642 | 0.0025 | 1.67E-16 | 400,102 | 67.24 |
| 55 | rs12737805 | A | G | 0.0203 | 0.7787 | 0.0028 | 6.57E-13 | 400,102 | 52.5625 |
| 56 | rs12825748 | C | G | 0.0198 | 0.3087 | 0.0025 | 6.27E-15 | 400,102 | 62.7264 |
| 57 | rs1286645 | A | G | 0.0167 | 0.1816 | 0.003 | 3.05E-08 | 400,102 | 30.98778 |
| 58 | rs12894780 | T | C | 0.0239 | 0.8741 | 0.0035 | 1.52E-11 | 400,102 | 46.62939 |
| 59 | rs12928404 | T | C | 0.0139 | 0.5896 | 0.0024 | 4.49E-09 | 400,102 | 33.5434 |
| 60 | rs13008330 | T | C | -0.0212 | 0.1309 | 0.0035 | 1.09E-09 | 400,102 | 36.68898 |
| 61 | rs13036142 | T | C | 0.0207 | 0.1973 | 0.0029 | 1.21E-12 | 400,102 | 50.95006 |
| 62 | rs13069228 | T | C | 0.0158 | 0.6386 | 0.0024 | 9.59E-11 | 400,102 | 43.34028 |
| 63 | rs13361953 | T | C | 0.0258 | 0.6645 | 0.0025 | 2.02E-25 | 400,102 | 106.5024 |
| 64 | rs13433809 | A | G | 0.0206 | 0.5607 | 0.0023 | 1.43E-18 | 400,102 | 80.21928 |
| 65 | rs1352658 | T | C | 0.0152 | 0.575 | 0.0024 | 1.86E-10 | 400,102 | 40.11111 |
| 66 | rs1360235 | A | G | -0.0146 | 0.5182 | 0.0023 | 3.87E-10 | 400,102 | 40.2949 |
| 67 | rs1392140 | A | G | -0.0154 | 0.7262 | 0.0026 | 4.28E-09 | 400,102 | 35.08284 |
| 68 | rs1441207 | C | G | 0.0217 | 0.26 | 0.0027 | 4.04E-16 | 400,102 | 64.59396 |
| 69 | rs149333984 | A | G | 0.036 | 0.0406 | 0.006 | 1.64E-09 | 400,102 | 36 |
| 70 | rs1499894 | T | C | 0.0196 | 0.4404 | 0.0024 | 1.02E-16 | 400,102 | 66.69444 |
| 71 | rs150459394 | A | T | -0.0319 | 0.9312 | 0.0047 | 6.85E-12 | 400,102 | 46.06655 |
| 72 | rs1530555 | T | C | 0.0186 | 0.3736 | 0.0024 | 1.32E-14 | 400,102 | 60.0625 |
| 73 | rs1543310 | T | C | -0.016 | 0.4595 | 0.0024 | 1.54E-11 | 400,102 | 44.44444 |
| 74 | rs1610263 | A | T | 0.0297 | 0.9124 | 0.0041 | 7.10E-13 | 400,102 | 52.47412 |
| 75 | rs16824937 | A | G | -0.0341 | 0.0704 | 0.0045 | 5.81E-14 | 400,102 | 57.42272 |
| 76 | rs16859007 | A | G | 0.0178 | 0.1518 | 0.0033 | 4.64E-08 | 400,102 | 29.09458 |
| 77 | rs17033254 | T | C | -0.0175 | 0.8016 | 0.0029 | 2.58E-09 | 400,102 | 36.41498 |
| 78 | rs17395720 | T | C | 0.0161 | 0.7685 | 0.0028 | 5.35E-09 | 400,102 | 33.0625 |
| 79 | rs17812980 | T | C | 0.0135 | 0.5033 | 0.0023 | 7.55E-09 | 400,102 | 34.4518 |
| 80 | rs1840168 | T | C | 0.017 | 0.2471 | 0.0027 | 3.16E-10 | 400,102 | 39.64335 |
| 81 | rs1896797 | A | G | 0.0218 | 0.4904 | 0.0023 | 1.59E-20 | 400,102 | 89.83743 |
| 82 | rs1909121 | A | G | 0.0281 | 0.0762 | 0.0044 | 2.10E-10 | 400,102 | 40.78564 |
| 83 | rs1968027 | T | C | -0.0184 | 0.2094 | 0.0029 | 1.57E-10 | 400,102 | 40.25684 |
| 84 | rs201191 | A | C | 0.0168 | 0.4858 | 0.0023 | 5.07E-13 | 400,102 | 53.3535 |
| 85 | rs2023681 | A | G | 0.0364 | 0.0933 | 0.004 | 1.10E-19 | 400,102 | 82.81 |
| 86 | rs2027761 | T | C | 0.0282 | 0.113 | 0.0037 | 2.54E-14 | 400,102 | 58.08912 |
| 87 | rs2040732 | T | C | 0.0148 | 0.4167 | 0.0024 | 2.87E-10 | 400,102 | 38.02778 |
| 88 | rs2095024 | A | G | 0.0166 | 0.5882 | 0.0024 | 2.27E-12 | 400,102 | 47.84028 |
| 89 | rs211401 | T | C | -0.0145 | 0.6344 | 0.0024 | 1.81E-09 | 400,102 | 36.50174 |
| 90 | rs2125787 | A | G | 0.0131 | 0.5996 | 0.0024 | 4.26E-08 | 400,102 | 29.7934 |
| 91 | rs2153997 | A | G | -0.0147 | 0.7034 | 0.0025 | 6.71E-09 | 400,102 | 34.5744 |
| 92 | rs2162319 | A | G | -0.0256 | 0.8237 | 0.0031 | 9.28E-17 | 400,102 | 68.19563 |
| 93 | rs217554 | A | G | 0.0135 | 0.6728 | 0.0025 | 4.67E-08 | 400,102 | 29.16 |
| 94 | rs2195242 | A | G | 0.0166 | 0.2623 | 0.0027 | 3.83E-10 | 400,102 | 37.79973 |
| 95 | rs2202572 | A | C | 0.0144 | 0.3289 | 0.0025 | 5.78E-09 | 400,102 | 33.1776 |
| 96 | rs2206925 | T | C | -0.0207 | 0.3622 | 0.0025 | 3.07E-17 | 400,102 | 68.5584 |
| 97 | rs221615 | A | G | -0.017 | 0.269 | 0.0026 | 1.15E-10 | 400,102 | 42.75148 |
| 98 | rs2236180 | T | C | 0.0213 | 0.815 | 0.003 | 1.02E-12 | 400,102 | 50.41 |
| 99 | rs2271804 | A | G | 0.0338 | 0.5298 | 0.0023 | 4.74E-48 | 400,102 | 215.9622 |
| 100 | rs227728 | A | T | -0.016 | 0.3337 | 0.0025 | 9.65E-11 | 400,102 | 40.96 |
| 101 | rs2304340 | A | G | -0.014 | 0.4084 | 0.0024 | 3.72E-09 | 400,102 | 34.02778 |
| 102 | rs2345443 | A | G | 0.0221 | 0.3114 | 0.0025 | 3.03E-18 | 400,102 | 78.1456 |
| 103 | rs2393729 | T | C | -0.0139 | 0.4211 | 0.0024 | 4.78E-09 | 400,102 | 33.5434 |
| 104 | rs2445772 | C | G | -0.0153 | 0.309 | 0.0025 | 1.46E-09 | 400,102 | 37.4544 |
| 105 | rs2489009 | T | C | -0.0134 | 0.5477 | 0.0024 | 1.85E-08 | 400,102 | 31.17361 |
| 106 | rs2521564 | A | G | -0.0144 | 0.5929 | 0.0024 | 1.58E-09 | 400,102 | 36 |
| 107 | rs2544535 | T | C | -0.0216 | 0.4881 | 0.0023 | 1.08E-20 | 400,102 | 88.1966 |
| 108 | rs2545675 | T | C | 0.0153 | 0.5213 | 0.0024 | 6.75E-11 | 400,102 | 40.64063 |
| 109 | rs2571445 | A | G | -0.0285 | 0.3971 | 0.0024 | 7.24E-33 | 400,102 | 141.0156 |
| 110 | rs2579762 | A | C | 0.0289 | 0.5276 | 0.0023 | 6.20E-35 | 400,102 | 157.8847 |
| 111 | rs2640562 | T | C | -0.0153 | 0.3457 | 0.0025 | 5.95E-10 | 400,102 | 37.4544 |
| 112 | rs268717 | T | C | -0.0307 | 0.9074 | 0.004 | 1.67E-14 | 400,102 | 58.90563 |
| 113 | rs2806356 | T | C | 0.0322 | 0.8153 | 0.003 | 1.09E-26 | 400,102 | 115.2044 |
| 114 | rs2808218 | T | C | -0.0208 | 0.6778 | 0.0025 | 1.09E-16 | 400,102 | 69.2224 |
| 115 | rs2811416 | T | C | 0.0206 | 0.1166 | 0.0036 | 1.61E-08 | 400,102 | 32.74383 |
| 116 | rs2812208 | C | G | 0.0612 | 0.0212 | 0.0081 | 4.95E-14 | 400,102 | 57.08642 |
| 117 | rs2816992 | A | G | -0.0131 | 0.5879 | 0.0024 | 3.38E-08 | 400,102 | 29.7934 |
| 118 | rs28361039 | A | G | -0.0233 | 0.6412 | 0.0026 | 1.26E-19 | 400,102 | 80.30917 |
| 119 | rs28519449 | T | C | 0.0205 | 0.4043 | 0.0024 | 3.51E-18 | 400,102 | 72.96007 |
| 120 | rs2875610 | A | G | 0.0197 | 0.6562 | 0.0024 | 6.48E-16 | 400,102 | 67.37674 |
| 121 | rs2885697 | T | G | -0.0137 | 0.6664 | 0.0024 | 2.26E-08 | 400,102 | 32.58507 |
| 122 | rs2894837 | A | G | 0.0175 | 0.6398 | 0.0024 | 9.22E-13 | 400,102 | 53.1684 |
| 123 | rs2909 | A | G | 0.0238 | 0.8736 | 0.0035 | 1.49E-11 | 400,102 | 46.24 |
| 124 | rs2974389 | A | G | 0.0165 | 0.4265 | 0.0023 | 1.70E-12 | 400,102 | 51.46503 |
| 125 | rs303752 | A | G | -0.0154 | 0.409 | 0.0024 | 1.29E-10 | 400,102 | 41.17361 |
| 126 | rs308381 | A | G | 0.0328 | 0.9591 | 0.0059 | 3.12E-08 | 400,102 | 30.90606 |
| 127 | rs3106209 | A | C | 0.0138 | 0.3288 | 0.0025 | 3.65E-08 | 400,102 | 30.4704 |
| 128 | rs3118008 | A | C | 0.0162 | 0.2768 | 0.0026 | 6.70E-10 | 400,102 | 38.82249 |
| 129 | rs3133084 | A | G | 0.0188 | 0.7659 | 0.0028 | 9.73E-12 | 400,102 | 45.08163 |
| 130 | rs3212656 | T | C | -0.0244 | 0.7891 | 0.0029 | 1.72E-17 | 400,102 | 70.79191 |
| 131 | rs3253 | T | C | 0.0162 | 0.3141 | 0.0025 | 1.28E-10 | 400,102 | 41.9904 |
| 132 | rs330925 | A | G | -0.0171 | 0.3092 | 0.0026 | 2.54E-11 | 400,102 | 43.25592 |
| 133 | rs34712979 | A | G | -0.0567 | 0.2561 | 0.0027 | 9.32E-97 | 400,102 | 441 |
| 134 | rs35089989 | T | C | -0.0306 | 0.7791 | 0.0028 | 1.15E-27 | 400,102 | 119.4337 |
| 135 | rs35506 | A | T | 0.0197 | 0.7161 | 0.0026 | 3.38E-14 | 400,102 | 57.40976 |
| 136 | rs35568625 | T | C | -0.0163 | 0.4652 | 0.0024 | 1.48E-11 | 400,102 | 46.12674 |
| 137 | rs35750964 | T | C | -0.02 | 0.7559 | 0.0027 | 1.87E-13 | 400,102 | 54.86968 |
| 138 | rs3746429 | T | C | -0.0209 | 0.164 | 0.0031 | 2.18E-11 | 400,102 | 45.45369 |
| 139 | rs3751837 | T | C | -0.0235 | 0.2202 | 0.0028 | 6.35E-17 | 400,102 | 70.44005 |
| 140 | rs3795948 | T | C | -0.0157 | 0.5181 | 0.0023 | 1.10E-11 | 400,102 | 46.59546 |
| 141 | rs3827382 | A | G | -0.0225 | 0.0926 | 0.004 | 1.86E-08 | 400,102 | 31.64063 |
| 142 | rs3856802 | T | C | 0.0139 | 0.5452 | 0.0023 | 2.97E-09 | 400,102 | 36.52363 |
| 143 | rs4074726 | T | C | 0.0149 | 0.2924 | 0.0026 | 7.07E-09 | 400,102 | 32.84172 |
| 144 | rs415170 | C | G | 0.0156 | 0.6705 | 0.0025 | 2.89E-10 | 400,102 | 38.9376 |
| 145 | rs425102 | T | G | 0.0159 | 0.7597 | 0.0027 | 6.55E-09 | 400,102 | 34.67901 |
| 146 | rs4478172 | A | C | 0.0184 | 0.7391 | 0.0026 | 3.19E-12 | 400,102 | 50.08284 |
| 147 | rs4490572 | A | G | -0.0184 | 0.4632 | 0.0024 | 9.24E-15 | 400,102 | 58.77778 |
| 148 | rs4724960 | A | G | 0.0159 | 0.6628 | 0.0025 | 1.03E-10 | 400,102 | 40.4496 |
| 149 | rs4726715 | T | G | -0.0138 | 0.4786 | 0.0024 | 4.66E-09 | 400,102 | 33.0625 |
| 150 | rs4730073 | A | C | 0.014 | 0.6432 | 0.0024 | 1.05E-08 | 400,102 | 34.02778 |
| 151 | rs4737183 | A | G | -0.0148 | 0.5264 | 0.0024 | 3.21E-10 | 400,102 | 38.02778 |
| 152 | rs4741893 | C | G | -0.0229 | 0.415 | 0.0024 | 4.98E-22 | 400,102 | 91.0434 |
| 153 | rs4748734 | T | G | -0.014 | 0.6931 | 0.0025 | 2.61E-08 | 400,102 | 31.36 |
| 154 | rs4800410 | A | C | -0.0197 | 0.5945 | 0.0024 | 1.25E-16 | 400,102 | 67.37674 |
| 155 | rs4803982 | T | C | 0.0172 | 0.1859 | 0.003 | 9.23E-09 | 400,102 | 32.87111 |
| 156 | rs4809327 | T | C | -0.0267 | 0.6756 | 0.0025 | 1.33E-26 | 400,102 | 114.0624 |
| 157 | rs4834214 | A | G | -0.0146 | 0.5515 | 0.0024 | 5.64E-10 | 400,102 | 37.00694 |
| 158 | rs4836246 | A | G | -0.0139 | 0.3388 | 0.0025 | 1.96E-08 | 400,102 | 30.9136 |
| 159 | rs4885681 | T | C | 0.0186 | 0.7248 | 0.0026 | 1.83E-12 | 400,102 | 51.17751 |
| 160 | rs4889599 | T | C | 0.0222 | 0.6284 | 0.0024 | 2.43E-20 | 400,102 | 85.5625 |
| 161 | rs4918429 | C | G | 0.0149 | 0.6236 | 0.0024 | 7.41E-10 | 400,102 | 38.5434 |
| 162 | rs4936101 | A | G | 0.0138 | 0.4049 | 0.0024 | 7.79E-09 | 400,102 | 33.0625 |
| 163 | rs4952571 | C | G | -0.0155 | 0.6783 | 0.0025 | 4.94E-10 | 400,102 | 38.44 |
| 164 | rs4955548 | A | C | 0.0159 | 0.3325 | 0.0025 | 2.01E-10 | 400,102 | 40.4496 |
| 165 | rs4968200 | C | G | -0.0219 | 0.1421 | 0.0033 | 4.54E-11 | 400,102 | 44.04132 |
| 166 | rs513953 | A | G | -0.0273 | 0.2542 | 0.0027 | 1.24E-24 | 400,102 | 102.2346 |
| 167 | rs552320 | A | G | -0.0164 | 0.309 | 0.0025 | 7.98E-11 | 400,102 | 43.0336 |
| 168 | rs55747751 | A | G | -0.0247 | 0.0808 | 0.0043 | 1.37E-08 | 400,102 | 32.99567 |
| 169 | rs55915240 | A | T | -0.0188 | 0.1863 | 0.003 | 5.05E-10 | 400,102 | 39.27111 |
| 170 | rs55916581 | T | C | -0.0151 | 0.7364 | 0.0026 | 1.12E-08 | 400,102 | 33.72929 |
| 171 | rs55951163 | A | G | 0.0175 | 0.3794 | 0.0024 | 4.55E-13 | 400,102 | 53.1684 |
| 172 | rs57380671 | T | C | -0.0182 | 0.8111 | 0.003 | 8.34E-10 | 400,102 | 36.80444 |
| 173 | rs57389113 | A | G | -0.0191 | 0.1701 | 0.0031 | 1.07E-09 | 400,102 | 37.9615 |
| 174 | rs59831071 | A | G | -0.013 | 0.4684 | 0.0024 | 3.62E-08 | 400,102 | 29.34028 |
| 175 | rs6032942 | C | G | 0.0173 | 0.2325 | 0.0027 | 3.47E-10 | 400,102 | 41.05487 |
| 176 | rs6042162 | T | C | 0.0144 | 0.6846 | 0.0025 | 8.59E-09 | 400,102 | 33.1776 |
| 177 | rs6066089 | C | G | 0.022 | 0.3059 | 0.0025 | 3.39E-18 | 400,102 | 77.44 |
| 178 | rs6103666 | A | G | -0.0181 | 0.2711 | 0.0026 | 4.83E-12 | 400,102 | 48.46302 |
| 179 | rs61482805 | C | G | 0.0178 | 0.6696 | 0.0025 | 1.03E-12 | 400,102 | 50.6944 |
| 180 | rs62091797 | T | G | -0.0258 | 0.8629 | 0.0034 | 5.52E-14 | 400,102 | 57.58131 |
| 181 | rs62126364 | A | G | 0.0302 | 0.219 | 0.0028 | 3.76E-27 | 400,102 | 116.3316 |
| 182 | rs62147658 | A | G | -0.0247 | 0.1264 | 0.0035 | 2.42E-12 | 400,102 | 49.80327 |
| 183 | rs62191107 | T | C | -0.0286 | 0.8013 | 0.0029 | 1.65E-22 | 400,102 | 97.2604 |
| 184 | rs62316310 | A | G | 0.0176 | 0.2603 | 0.0027 | 4.53E-11 | 400,102 | 42.49108 |
| 185 | rs6425501 | A | G | -0.0182 | 0.6477 | 0.0024 | 1.04E-13 | 400,102 | 57.50694 |
| 186 | rs6445932 | T | G | -0.0287 | 0.7519 | 0.0027 | 3.82E-26 | 400,102 | 112.989 |
| 187 | rs6450095 | A | T | -0.016 | 0.2011 | 0.0029 | 4.30E-08 | 400,102 | 30.43995 |
| 188 | rs6462481 | T | C | -0.0161 | 0.2314 | 0.0028 | 5.28E-09 | 400,102 | 33.0625 |
| 189 | rs6470697 | A | C | -0.0283 | 0.9014 | 0.0039 | 6.55E-13 | 400,102 | 52.65549 |
| 190 | rs6500126 | A | G | 0.0144 | 0.3388 | 0.0025 | 4.91E-09 | 400,102 | 33.1776 |
| 191 | rs6501455 | A | G | 0.0296 | 0.5015 | 0.0023 | 1.28E-36 | 400,102 | 165.6257 |
| 192 | rs6539952 | A | C | -0.017 | 0.2615 | 0.0027 | 3.50E-10 | 400,102 | 39.64335 |
| 193 | rs659398 | T | C | -0.0175 | 0.2719 | 0.0027 | 4.35E-11 | 400,102 | 42.0096 |
| 194 | rs661857 | T | C | -0.0148 | 0.5156 | 0.0023 | 3.08E-10 | 400,102 | 41.40643 |
| 195 | rs6692738 | A | G | 0.0185 | 0.2853 | 0.0026 | 8.67E-13 | 400,102 | 50.6287 |
| 196 | rs6697620 | A | G | 0.0226 | 0.4343 | 0.0024 | 1.10E-21 | 400,102 | 88.67361 |
| 197 | rs6723572 | T | C | -0.0256 | 0.818 | 0.003 | 1.46E-17 | 400,102 | 72.81778 |
| 198 | rs6740092 | A | T | 0.0227 | 0.8549 | 0.0033 | 1.07E-11 | 400,102 | 47.31772 |
| 199 | rs676274 | A | G | 0.014 | 0.5823 | 0.0024 | 2.47E-09 | 400,102 | 34.02778 |
| 200 | rs6780171 | A | T | -0.0174 | 0.3129 | 0.0025 | 6.18E-12 | 400,102 | 48.4416 |
| 201 | rs6807089 | T | C | 0.0131 | 0.5359 | 0.0024 | 2.86E-08 | 400,102 | 29.7934 |
| 202 | rs6830774 | T | C | 0.0145 | 0.5331 | 0.0023 | 6.76E-10 | 400,102 | 39.7448 |
| 203 | rs6904757 | A | G | 0.0157 | 0.634 | 0.0024 | 1.58E-10 | 400,102 | 42.7934 |
| 204 | rs7028528 | A | C | -0.0149 | 0.6721 | 0.0025 | 2.39E-09 | 400,102 | 35.5216 |
| 205 | rs7092539 | T | C | -0.0222 | 0.815 | 0.003 | 1.79E-13 | 400,102 | 54.76 |
| 206 | rs7116641 | T | G | 0.0185 | 0.6829 | 0.0025 | 1.21E-13 | 400,102 | 54.76 |
| 207 | rs7139311 | T | C | 0.0131 | 0.5354 | 0.0023 | 1.69E-08 | 400,102 | 32.44045 |
| 208 | rs71490394 | A | G | 0.0264 | 0.3671 | 0.0024 | 1.66E-27 | 400,102 | 121 |
| 209 | rs7198383 | T | C | 0.0155 | 0.6825 | 0.0025 | 6.78E-10 | 400,102 | 38.44 |
| 210 | rs72643433 | A | G | -0.019 | 0.251 | 0.0027 | 2.10E-12 | 400,102 | 49.51989 |
| 211 | rs72718111 | A | C | -0.016 | 0.7425 | 0.0027 | 2.85E-09 | 400,102 | 35.1166 |
| 212 | rs72737267 | T | C | 0.0211 | 0.1228 | 0.0036 | 3.27E-09 | 400,102 | 34.35262 |
| 213 | rs72894568 | A | G | -0.0427 | 0.9755 | 0.0076 | 2.23E-08 | 400,102 | 31.56666 |
| 214 | rs72995681 | T | G | 0.0167 | 0.6286 | 0.0024 | 5.21E-12 | 400,102 | 48.4184 |
| 215 | rs731052 | T | C | 0.0381 | 0.067 | 0.0046 | 2.06E-16 | 400,102 | 68.60161 |
| 216 | rs73154306 | T | C | 0.025 | 0.7283 | 0.0026 | 2.27E-21 | 400,102 | 92.45562 |
| 217 | rs73182224 | A | G | -0.0178 | 0.2389 | 0.0029 | 6.00E-10 | 400,102 | 37.6742 |
| 218 | rs7424771 | A | G | -0.0169 | 0.4468 | 0.0024 | 6.57E-13 | 400,102 | 49.58507 |
| 219 | rs7516060 | A | G | 0.0138 | 0.6359 | 0.0024 | 1.99E-08 | 400,102 | 33.0625 |
| 220 | rs7517716 | A | C | -0.0172 | 0.4681 | 0.0023 | 2.09E-13 | 400,102 | 55.92439 |
| 221 | rs755249 | T | C | -0.0202 | 0.2328 | 0.0027 | 1.52E-13 | 400,102 | 55.97257 |
| 222 | rs76219171 | A | G | -0.0283 | 0.0601 | 0.005 | 1.26E-08 | 400,102 | 32.0356 |
| 223 | rs7639737 | T | C | 0.0325 | 0.7954 | 0.0029 | 4.18E-29 | 400,102 | 125.5945 |
| 224 | rs7663740 | T | G | 0.0405 | 0.5438 | 0.0023 | 1.17E-66 | 400,102 | 310.0662 |
| 225 | rs7700595 | A | G | -0.0227 | 0.8762 | 0.0036 | 1.94E-10 | 400,102 | 39.76003 |
| 226 | rs7733410 | A | G | 0.0342 | 0.4407 | 0.0024 | 7.12E-47 | 400,102 | 203.0625 |
| 227 | rs7753563 | A | G | -0.0201 | 0.2323 | 0.0028 | 4.14E-13 | 400,102 | 51.53189 |
| 228 | rs7806296 | A | G | -0.019 | 0.36 | 0.0024 | 4.19E-15 | 400,102 | 62.67361 |
| 229 | rs78101726 | A | G | 0.0334 | 0.8459 | 0.0032 | 7.72E-25 | 400,102 | 108.9414 |
| 230 | rs7838717 | T | C | -0.0217 | 0.3631 | 0.0025 | 1.83E-18 | 400,102 | 75.3424 |
| 231 | rs78579285 | T | C | -0.0205 | 0.182 | 0.003 | 1.85E-11 | 400,102 | 46.69444 |
| 232 | rs7906816 | A | G | -0.0312 | 0.9426 | 0.005 | 5.74E-10 | 400,102 | 38.9376 |
| 233 | rs7949728 | C | G | 0.0152 | 0.5902 | 0.0024 | 1.70E-10 | 400,102 | 40.11111 |
| 234 | rs7977418 | T | C | 0.0322 | 0.5415 | 0.0023 | 1.42E-43 | 400,102 | 196 |
| 235 | rs8006310 | A | G | 0.0139 | 0.5525 | 0.0024 | 4.74E-09 | 400,102 | 33.5434 |
| 236 | rs8030071 | A | C | 0.0137 | 0.3545 | 0.0024 | 1.95E-08 | 400,102 | 32.58507 |
| 237 | rs8033889 | T | G | -0.0301 | 0.2192 | 0.0028 | 7.08E-27 | 400,102 | 115.5625 |
| 238 | rs803909 | T | G | 0.0212 | 0.4591 | 0.0024 | 2.08E-19 | 400,102 | 78.02778 |
| 239 | rs8040868 | T | C | 0.0186 | 0.6078 | 0.0024 | 7.92E-15 | 400,102 | 60.0625 |
| 240 | rs8067252 | T | C | 0.0192 | 0.229 | 0.0028 | 1.26E-11 | 400,102 | 47.02041 |
| 241 | rs8089099 | A | G | 0.0163 | 0.2746 | 0.0026 | 4.35E-10 | 400,102 | 39.30325 |
| 242 | rs849135 | A | G | 0.0198 | 0.4999 | 0.0023 | 1.05E-17 | 400,102 | 74.10964 |
| 243 | rs881755 | T | C | -0.0246 | 0.4844 | 0.0023 | 9.06E-26 | 400,102 | 114.397 |
| 244 | rs886444 | A | G | 0.0136 | 0.405 | 0.0024 | 8.37E-09 | 400,102 | 32.11111 |
| 245 | rs930527 | T | C | 0.0131 | 0.4136 | 0.0024 | 3.12E-08 | 400,102 | 29.7934 |
| 246 | rs9350191 | T | C | -0.0236 | 0.8478 | 0.0033 | 4.68E-13 | 400,102 | 51.14417 |
| 247 | rs9357446 | A | G | -0.0134 | 0.5169 | 0.0023 | 8.12E-09 | 400,102 | 33.94329 |
| 248 | rs9385988 | A | G | -0.0279 | 0.7232 | 0.0026 | 1.40E-26 | 400,102 | 115.1494 |
| 249 | rs944190 | T | G | -0.0156 | 0.5905 | 0.0024 | 5.71E-11 | 400,102 | 42.25 |
| 250 | rs979453 | A | G | 0.0189 | 0.6745 | 0.0025 | 3.61E-14 | 400,102 | 57.1536 |
| 251 | rs9807668 | T | C | 0.0294 | 0.0946 | 0.004 | 1.39E-13 | 400,102 | 54.0225 |
| 252 | rs9819463 | T | C | 0.0224 | 0.7958 | 0.0029 | 1.14E-14 | 400,102 | 59.66231 |
| 253 | rs9970286 | A | G | 0.0172 | 0.3291 | 0.0025 | 5.91E-12 | 400,102 | 47.3344 |

Table S2 Selected genetic instrumental variables of FEV1
