# Supplementary material for: COPD, PRISm and lung function reduction affect the brain cortical structure: a Mendelian randomization study
Source: BMC Pulm Med. 2024 Jul 15;24:341. doi: 10.1186/s12890-024-03150-2 (PMC11251327; doi:10.1186/s12890-024-03150-2)
Supplement: Supplementary file 3 — Supplementary Material 3. [file 12890_2024_3150_MOESM3_ESM.docx]

|  | SNP | effect_allele.exposure | other_allele.exposure | beta.exposure | eaf.exposure | se.exposure | pval.exposure | samplesize | F |
| --- | --- | --- | --- | --- | --- | --- | --- | --- | --- |
| 1 | rs10005540 | T | C | 0.0173 | 0.615 | 0.0024 | 1.53E-12 | 400,102 | 51.9600694 |
| 2 | rs1007330 | T | C | 0.0239 | 0.383 | 0.0024 | 2.17E-23 | 400,102 | 99.1684028 |
| 3 | rs1008833 | A | G | -0.0244 | 0.854 | 0.0033 | 2.41E-13 | 400,102 | 54.6703398 |
| 4 | rs10089933 | T | C | 0.0165 | 0.757 | 0.0027 | 1.81E-09 | 400,102 | 37.345679 |
| 5 | rs10214450 | T | C | 0.0378 | 0.0464 | 0.0056 | 1.20E-11 | 400,102 | 45.5625 |
| 6 | rs10251085 | A | G | 0.0166 | 0.243 | 0.0028 | 2.31E-09 | 400,102 | 35.1479592 |
| 7 | rs10273455 | A | C | -0.0149 | 0.552 | 0.0023 | 2.15E-10 | 400,102 | 41.9678639 |
| 8 | rs10448340 | T | G | 0.0152 | 0.679 | 0.0025 | 1.88E-09 | 400,102 | 36.9664 |
| 9 | rs1047912 | T | C | 0.0167 | 0.299 | 0.0025 | 4.85E-11 | 400,102 | 44.6224 |
| 10 | rs10489880 | A | G | -0.0166 | 0.721 | 0.0026 | 2.38E-10 | 400,102 | 40.7633136 |
| 11 | rs10492367 | T | G | 0.0236 | 0.188 | 0.003 | 2.88E-15 | 400,102 | 61.8844444 |
| 12 | rs10498635 | T | C | 0.0362 | 0.183 | 0.003 | 1.34E-32 | 400,102 | 145.604444 |
| 13 | rs10508438 | A | G | -0.0173 | 0.336 | 0.0025 | 3.08E-12 | 400,102 | 47.8864 |
| 14 | rs1052939 | A | G | -0.0147 | 0.262 | 0.0027 | 4.82E-08 | 400,102 | 29.6419753 |
| 15 | rs1054661 | T | C | -0.0169 | 0.653 | 0.0024 | 4.61E-12 | 400,102 | 49.5850694 |
| 16 | rs10761722 | C | G | 0.0188 | 0.185 | 0.003 | 4.97E-10 | 400,102 | 39.2711111 |
| 17 | rs10850377 | A | G | 0.0159 | 0.341 | 0.0025 | 1.82E-10 | 400,102 | 40.4496 |
| 18 | rs10922140 | A | G | 0.0139 | 0.448 | 0.0024 | 7.18E-09 | 400,102 | 33.5434028 |
| 19 | rs10929647 | A | C | -0.0136 | 0.513 | 0.0023 | 5.64E-09 | 400,102 | 34.9640832 |
| 20 | rs10997955 | T | C | -0.0182 | 0.589 | 0.0024 | 3.32E-14 | 400,102 | 57.5069444 |
| 21 | rs11051013 | T | C | 0.0164 | 0.592 | 0.0024 | 5.11E-12 | 400,102 | 46.6944444 |
| 22 | rs11074546 | T | G | 0.0169 | 0.262 | 0.0027 | 1.99E-10 | 400,102 | 39.1783265 |
| 23 | rs1109391 | T | G | 0.0172 | 0.568 | 0.0024 | 2.42E-13 | 400,102 | 51.3611111 |
| 24 | rs11103381 | A | C | 0.0238 | 0.673 | 0.0025 | 4.86E-21 | 400,102 | 90.6304 |
| 25 | rs11241692 | T | C | 0.0136 | 0.56 | 0.0024 | 9.46E-09 | 400,102 | 32.1111111 |
| 26 | rs1125909 | A | G | 0.0139 | 0.694 | 0.0025 | 3.61E-08 | 400,102 | 30.9136 |
| 27 | rs1154862 | T | C | -0.0141 | 0.678 | 0.0025 | 1.68E-08 | 400,102 | 31.8096 |
| 28 | rs116336057 | C | G | -0.0178 | 0.182 | 0.0031 | 5.65E-09 | 400,102 | 32.9698231 |
| 29 | rs11702251 | T | G | -0.0184 | 0.223 | 0.0028 | 5.90E-11 | 400,102 | 43.1836735 |
| 30 | rs11718554 | A | G | 0.0263 | 0.656 | 0.0025 | 1.68E-26 | 400,102 | 110.6704 |
| 31 | rs11722225 | T | C | -0.0561 | 0.933 | 0.0047 | 1.78E-32 | 400,102 | 142.472159 |
| 32 | rs11727676 | T | C | 0.0325 | 0.905 | 0.004 | 8.05E-16 | 400,102 | 66.015625 |
| 33 | rs11747434 | T | C | -0.015 | 0.722 | 0.0026 | 1.23E-08 | 400,102 | 33.2840237 |
| 34 | rs11767893 | T | C | 0.0163 | 0.314 | 0.0027 | 1.60E-09 | 400,102 | 36.4458162 |
| 35 | rs1192414 | A | G | 0.0204 | 0.179 | 0.0031 | 2.69E-11 | 400,102 | 43.3048907 |
| 36 | rs1207776 | T | C | -0.0151 | 0.362 | 0.0024 | 6.30E-10 | 400,102 | 39.5850694 |
| 37 | rs1215 | A | G | 0.0217 | 0.857 | 0.0033 | 9.64E-11 | 400,102 | 43.2405877 |
| 38 | rs12204336 | A | G | -0.0155 | 0.374 | 0.0024 | 1.46E-10 | 400,102 | 41.7100694 |
| 39 | rs12413039 | C | G | -0.019 | 0.73 | 0.0026 | 7.09E-13 | 400,102 | 53.4023669 |
| 40 | rs1247943 | A | G | 0.0144 | 0.533 | 0.0024 | 1.02E-09 | 400,102 | 36 |
| 41 | rs1249093 | A | G | 0.0206 | 0.538 | 0.0024 | 3.93E-18 | 400,102 | 73.6736111 |
| 42 | rs12497779 | T | G | -0.0319 | 0.233 | 0.0028 | 1.85E-30 | 400,102 | 129.797194 |
| 43 | rs12543555 | A | G | 0.0229 | 0.793 | 0.0029 | 4.91E-15 | 400,102 | 62.3555291 |
| 44 | rs12571363 | A | C | -0.0314 | 0.107 | 0.0038 | 2.06E-16 | 400,102 | 68.2797784 |
| 45 | rs12698403 | A | G | -0.0167 | 0.442 | 0.0024 | 2.37E-12 | 400,102 | 48.4184028 |
| 46 | rs12713676 | T | C | -0.0159 | 0.656 | 0.0025 | 2.58E-10 | 400,102 | 40.4496 |
| 47 | rs12727617 | T | C | 0.024 | 0.243 | 0.0027 | 2.31E-18 | 400,102 | 79.0123457 |
| 48 | rs12737805 | A | G | 0.0206 | 0.779 | 0.0028 | 3.58E-13 | 400,102 | 54.127551 |
| 49 | rs12825748 | C | G | 0.0196 | 0.309 | 0.0025 | 1.68E-14 | 400,102 | 61.4656 |
| 50 | rs12945803 | T | C | 0.02 | 0.782 | 0.0028 | 1.88E-12 | 400,102 | 51.0204082 |
| 51 | rs13009582 | A | G | 0.0158 | 0.451 | 0.0023 | 1.52E-11 | 400,102 | 47.1909263 |
| 52 | rs13035369 | T | C | 0.0201 | 0.869 | 0.0035 | 8.66E-09 | 400,102 | 32.9804082 |
| 53 | rs13137088 | T | C | -0.0137 | 0.505 | 0.0024 | 7.26E-09 | 400,102 | 32.5850694 |
| 54 | rs13198515 | A | G | 0.0174 | 0.732 | 0.0026 | 4.71E-11 | 400,102 | 44.7869822 |
| 55 | rs13216391 | A | G | 0.0351 | 0.821 | 0.003 | 6.63E-31 | 400,102 | 136.89 |
| 56 | rs13220614 | T | C | 0.0139 | 0.4 | 0.0024 | 7.14E-09 | 400,102 | 33.5434028 |
| 57 | rs13227429 | T | C | 0.0163 | 0.439 | 0.0024 | 8.83E-12 | 400,102 | 46.1267361 |
| 58 | rs13430465 | T | C | 0.0368 | 0.0803 | 0.0043 | 1.87E-17 | 400,102 | 73.2417523 |
| 59 | rs13433809 | A | G | 0.0203 | 0.561 | 0.0024 | 6.80E-18 | 400,102 | 71.5434028 |
| 60 | rs1383304 | A | G | 0.0243 | 0.868 | 0.0035 | 2.59E-12 | 400,102 | 48.2032653 |
| 61 | rs1392140 | A | G | -0.0181 | 0.726 | 0.0026 | 7.02E-12 | 400,102 | 48.4630178 |
| 62 | rs1423127 | T | C | 0.0165 | 0.382 | 0.0024 | 8.39E-12 | 400,102 | 47.265625 |
| 63 | rs143384 | A | G | 0.0239 | 0.596 | 0.0024 | 1.03E-23 | 400,102 | 99.1684028 |
| 64 | rs1459222 | T | G | -0.0142 | 0.358 | 0.0025 | 6.43E-09 | 400,102 | 32.2624 |
| 65 | rs148561062 | A | G | -0.0313 | 0.0547 | 0.0052 | 1.82E-09 | 400,102 | 36.2311391 |
| 66 | rs1490384 | T | C | 0.0211 | 0.498 | 0.0024 | 3.13E-19 | 400,102 | 77.2934028 |
| 67 | rs1534566 | T | C | 0.017 | 0.338 | 0.0025 | 1.05E-11 | 400,102 | 46.24 |
| 68 | rs1543852 | A | G | 0.0131 | 0.402 | 0.0024 | 4.85E-08 | 400,102 | 29.7934028 |
| 69 | rs1554902 | A | C | 0.0145 | 0.457 | 0.0024 | 1.22E-09 | 400,102 | 36.5017361 |
| 70 | rs156838 | A | G | -0.0151 | 0.358 | 0.0025 | 1.08E-09 | 400,102 | 36.4816 |
| 71 | rs1610265 | T | C | -0.0379 | 0.0769 | 0.0044 | 8.81E-18 | 400,102 | 74.1947314 |
| 72 | rs1668091 | T | C | -0.0173 | 0.681 | 0.0025 | 5.24E-12 | 400,102 | 47.8864 |
| 73 | rs17009288 | A | C | -0.0251 | 0.706 | 0.0026 | 3.68E-22 | 400,102 | 93.1967456 |
| 74 | rs1708302 | T | C | 0.0186 | 0.5 | 0.0023 | 1.56E-15 | 400,102 | 65.3988658 |
| 75 | rs17168761 | T | C | -0.0205 | 0.121 | 0.0036 | 1.10E-08 | 400,102 | 32.4266975 |
| 76 | rs17280293 | A | G | 0.0699 | 0.973 | 0.0073 | 8.57E-22 | 400,102 | 91.6871833 |
| 77 | rs17466747 | A | G | -0.0142 | 0.339 | 0.0025 | 1.14E-08 | 400,102 | 32.2624 |
| 78 | rs1776634 | T | G | 0.0161 | 0.733 | 0.0026 | 1.17E-09 | 400,102 | 38.3446746 |
| 79 | rs17767210 | T | C | -0.0226 | 0.592 | 0.0024 | 3.45E-21 | 400,102 | 88.6736111 |
| 80 | rs17855177 | T | C | 0.017 | 0.337 | 0.0025 | 9.49E-12 | 400,102 | 46.24 |
| 81 | rs1801144 | C | G | 0.0236 | 0.362 | 0.0025 | 8.72E-22 | 400,102 | 89.1136 |
| 82 | rs1862342 | T | C | 0.0157 | 0.448 | 0.0024 | 3.83E-11 | 400,102 | 42.7934028 |
| 83 | rs1887667 | T | G | -0.0133 | 0.559 | 0.0024 | 1.41E-08 | 400,102 | 30.7100694 |
| 84 | rs1954179 | A | G | -0.0145 | 0.645 | 0.0025 | 4.85E-09 | 400,102 | 33.64 |
| 85 | rs1972503 | A | G | -0.0146 | 0.525 | 0.0023 | 4.07E-10 | 400,102 | 40.294896 |
| 86 | rs2095024 | A | G | 0.0161 | 0.588 | 0.0024 | 1.43E-11 | 400,102 | 45.0017361 |
| 87 | rs2118022 | T | C | 0.0145 | 0.643 | 0.0025 | 3.81E-09 | 400,102 | 33.64 |
| 88 | rs2146098 | A | G | -0.018 | 0.645 | 0.0024 | 2.03E-13 | 400,102 | 56.25 |
| 89 | rs2195242 | A | G | 0.0157 | 0.262 | 0.0027 | 4.60E-09 | 400,102 | 33.8120713 |
| 90 | rs2202572 | A | C | 0.0162 | 0.329 | 0.0025 | 7.01E-11 | 400,102 | 41.9904 |
| 91 | rs2209237 | A | T | -0.0137 | 0.645 | 0.0025 | 2.65E-08 | 400,102 | 30.0304 |
| 92 | rs2240147 | A | C | 0.0206 | 0.117 | 0.0037 | 2.44E-08 | 400,102 | 30.9978086 |
| 93 | rs2256154 | T | C | -0.0153 | 0.659 | 0.0025 | 7.35E-10 | 400,102 | 37.4544 |
| 94 | rs2273101 | T | C | 0.0182 | 0.247 | 0.0027 | 1.71E-11 | 400,102 | 45.4375857 |
| 95 | rs2286425 | T | C | -0.0209 | 0.526 | 0.0024 | 6.53E-19 | 400,102 | 75.8350694 |
| 96 | rs2304340 | A | G | -0.0158 | 0.408 | 0.0024 | 4.06E-11 | 400,102 | 43.3402778 |
| 97 | rs2345443 | A | G | 0.0181 | 0.311 | 0.0025 | 1.36E-12 | 400,102 | 52.4176 |
| 98 | rs2393729 | T | C | -0.0168 | 0.421 | 0.0024 | 2.04E-12 | 400,102 | 49 |
| 99 | rs2445772 | C | G | -0.0161 | 0.309 | 0.0025 | 2.52E-10 | 400,102 | 41.4736 |
| 100 | rs2474032 | T | C | -0.0133 | 0.563 | 0.0024 | 2.29E-08 | 400,102 | 30.7100694 |
| 101 | rs2502136 | T | C | -0.0132 | 0.469 | 0.0024 | 2.37E-08 | 400,102 | 30.25 |
| 102 | rs2521879 | A | G | 0.0203 | 0.196 | 0.003 | 1.16E-11 | 400,102 | 45.7877778 |
| 103 | rs2571445 | A | G | -0.022 | 0.397 | 0.0024 | 4.53E-20 | 400,102 | 84.0277778 |
| 104 | rs2609261 | A | G | -0.0248 | 0.215 | 0.0029 | 5.24E-18 | 400,102 | 73.1319857 |
| 105 | rs2637254 | A | G | -0.0224 | 0.511 | 0.0024 | 1.68E-21 | 400,102 | 87.1111111 |
| 106 | rs268717 | T | C | -0.034 | 0.907 | 0.004 | 3.23E-17 | 400,102 | 72.25 |
| 107 | rs2702200 | A | G | -0.0134 | 0.556 | 0.0024 | 1.34E-08 | 400,102 | 31.1736111 |
| 108 | rs274792 | T | C | 0.0183 | 0.687 | 0.0025 | 4.19E-13 | 400,102 | 53.5824 |
| 109 | rs2812208 | C | G | 0.0623 | 0.0212 | 0.0082 | 2.30E-14 | 400,102 | 57.7229328 |
| 110 | rs2816992 | A | G | -0.0164 | 0.588 | 0.0024 | 7.33E-12 | 400,102 | 46.6944444 |
| 111 | rs28472312 | T | C | -0.0159 | 0.701 | 0.0026 | 1.00E-09 | 400,102 | 37.397929 |
| 112 | rs28519449 | T | C | 0.0209 | 0.404 | 0.0024 | 1.27E-18 | 400,102 | 75.8350694 |
| 113 | rs2885697 | T | G | -0.0172 | 0.666 | 0.0025 | 2.67E-12 | 400,102 | 47.3344 |
| 114 | rs2967516 | A | G | -0.0152 | 0.709 | 0.0026 | 3.72E-09 | 400,102 | 34.1775148 |
| 115 | rs2974389 | A | G | 0.0155 | 0.426 | 0.0024 | 4.73E-11 | 400,102 | 41.7100694 |
| 116 | rs2976602 | T | C | 0.0133 | 0.46 | 0.0024 | 2.27E-08 | 400,102 | 30.7100694 |
| 117 | rs2999158 | T | C | 0.0145 | 0.335 | 0.0025 | 6.24E-09 | 400,102 | 33.64 |
| 118 | rs314262 | A | G | 0.0164 | 0.546 | 0.0024 | 3.84E-12 | 400,102 | 46.6944444 |
| 119 | rs3253 | T | C | 0.0182 | 0.314 | 0.0025 | 7.83E-13 | 400,102 | 52.9984 |
| 120 | rs34752570 | A | G | 0.0159 | 0.454 | 0.0024 | 2.46E-11 | 400,102 | 43.890625 |
| 121 | rs34811474 | A | G | 0.0176 | 0.23 | 0.0028 | 3.32E-10 | 400,102 | 39.5102041 |
| 122 | rs35189772 | A | G | 0.0193 | 0.859 | 0.0035 | 2.41E-08 | 400,102 | 30.4073469 |
| 123 | rs35480566 | A | G | -0.0221 | 0.56 | 0.0024 | 1.25E-20 | 400,102 | 84.7934028 |
| 124 | rs35506 | A | T | 0.0232 | 0.716 | 0.0026 | 6.88E-19 | 400,102 | 79.6213018 |
| 125 | rs35688953 | T | C | 0.0234 | 0.77 | 0.0028 | 4.30E-17 | 400,102 | 69.8418367 |
| 126 | rs3751837 | T | C | -0.0308 | 0.22 | 0.0028 | 9.07E-28 | 400,102 | 121 |
| 127 | rs3790076 | T | G | -0.0238 | 0.573 | 0.0024 | 1.23E-23 | 400,102 | 98.3402778 |
| 128 | rs3924361 | A | G | 0.0177 | 0.553 | 0.0024 | 4.90E-14 | 400,102 | 54.390625 |
| 129 | rs41310284 | A | C | 0.0279 | 0.101 | 0.004 | 1.70E-12 | 400,102 | 48.650625 |
| 130 | rs4132748 | T | C | -0.02 | 0.307 | 0.0026 | 6.96E-15 | 400,102 | 59.1715976 |
| 131 | rs4396807 | C | G | -0.0137 | 0.323 | 0.0025 | 4.19E-08 | 400,102 | 30.0304 |
| 132 | rs4490572 | A | G | -0.0163 | 0.463 | 0.0024 | 8.54E-12 | 400,102 | 46.1267361 |
| 133 | rs4652333 | C | G | 0.0172 | 0.312 | 0.0025 | 1.19E-11 | 400,102 | 47.3344 |
| 134 | rs470450 | A | G | 0.0157 | 0.686 | 0.0025 | 4.82E-10 | 400,102 | 39.4384 |
| 135 | rs4737183 | A | G | -0.0174 | 0.526 | 0.0024 | 1.67E-13 | 400,102 | 52.5625 |
| 136 | rs4803981 | T | C | -0.0173 | 0.814 | 0.003 | 9.48E-09 | 400,102 | 33.2544444 |
| 137 | rs4877815 | A | G | -0.0151 | 0.525 | 0.0024 | 1.67E-10 | 400,102 | 39.5850694 |
| 138 | rs4885681 | T | C | 0.018 | 0.725 | 0.0027 | 1.31E-11 | 400,102 | 44.4444444 |
| 139 | rs4924525 | A | C | -0.0171 | 0.522 | 0.0023 | 3.44E-13 | 400,102 | 55.2759924 |
| 140 | rs4936101 | A | G | 0.0146 | 0.405 | 0.0024 | 1.26E-09 | 400,102 | 37.0069444 |
| 141 | rs4938337 | A | T | -0.0204 | 0.874 | 0.0036 | 1.83E-08 | 400,102 | 32.1111111 |
| 142 | rs4952564 | A | G | -0.0172 | 0.681 | 0.0025 | 6.97E-12 | 400,102 | 47.3344 |
| 143 | rs4959021 | T | C | 0.0295 | 0.188 | 0.0031 | 5.67E-21 | 400,102 | 90.5567118 |
| 144 | rs509317 | A | T | -0.018 | 0.172 | 0.0031 | 6.44E-09 | 400,102 | 33.7148803 |
| 145 | rs521074 | T | C | 0.0139 | 0.669 | 0.0025 | 2.85E-08 | 400,102 | 30.9136 |
| 146 | rs555520 | C | G | -0.0155 | 0.658 | 0.0025 | 6.51E-10 | 400,102 | 38.44 |
| 147 | rs55695529 | A | T | -0.022 | 0.854 | 0.0033 | 4.82E-11 | 400,102 | 44.4444444 |
| 148 | rs5763825 | A | G | -0.02 | 0.884 | 0.0036 | 4.01E-08 | 400,102 | 30.8641975 |
| 149 | rs58507021 | T | C | -0.0145 | 0.696 | 0.0026 | 1.45E-08 | 400,102 | 31.102071 |
| 150 | rs5992134 | T | G | 0.0208 | 0.239 | 0.0027 | 3.65E-14 | 400,102 | 59.3470508 |
| 151 | rs60150206 | A | G | 0.0447 | 0.0906 | 0.0041 | 1.63E-27 | 400,102 | 118.863177 |
| 152 | rs6090040 | A | C | -0.013 | 0.479 | 0.0024 | 4.60E-08 | 400,102 | 29.3402778 |
| 153 | rs6117690 | T | C | -0.0159 | 0.242 | 0.0028 | 8.38E-09 | 400,102 | 32.2461735 |
| 154 | rs62193248 | A | T | 0.0151 | 0.66 | 0.0025 | 1.79E-09 | 400,102 | 36.4816 |
| 155 | rs62213409 | T | C | -0.0206 | 0.294 | 0.0026 | 1.48E-15 | 400,102 | 62.7751479 |
| 156 | rs62347029 | A | T | 0.016 | 0.226 | 0.0028 | 1.72E-08 | 400,102 | 32.6530612 |
| 157 | rs62511595 | A | G | -0.0135 | 0.624 | 0.0024 | 2.86E-08 | 400,102 | 31.640625 |
| 158 | rs62579839 | T | C | -0.0157 | 0.716 | 0.0026 | 1.91E-09 | 400,102 | 36.4630178 |
| 159 | rs6431620 | T | G | 0.0185 | 0.789 | 0.0029 | 1.40E-10 | 400,102 | 40.6956005 |
| 160 | rs6485443 | T | C | -0.0198 | 0.317 | 0.0025 | 3.53E-15 | 400,102 | 62.7264 |
| 161 | rs6554237 | T | C | 0.0145 | 0.486 | 0.0024 | 7.53E-10 | 400,102 | 36.5017361 |
| 162 | rs6571778 | A | G | -0.0137 | 0.353 | 0.0024 | 2.12E-08 | 400,102 | 32.5850694 |
| 163 | rs6595403 | T | C | 0.0155 | 0.576 | 0.0024 | 8.79E-11 | 400,102 | 41.7100694 |
| 164 | rs661857 | T | C | -0.0161 | 0.516 | 0.0024 | 8.28E-12 | 400,102 | 45.0017361 |
| 165 | rs6658835 | A | G | -0.0219 | 0.732 | 0.0027 | 2.09E-16 | 400,102 | 65.7901235 |
| 166 | rs66726071 | A | G | 0.0253 | 0.863 | 0.0034 | 2.01E-13 | 400,102 | 55.3711073 |
| 167 | rs6740092 | A | T | 0.026 | 0.855 | 0.0034 | 9.66E-15 | 400,102 | 58.4775087 |
| 168 | rs6754311 | T | C | -0.0227 | 0.734 | 0.0027 | 7.35E-17 | 400,102 | 70.6844993 |
| 169 | rs6755553 | T | C | -0.0147 | 0.546 | 0.0024 | 6.14E-10 | 400,102 | 37.515625 |
| 170 | rs6775611 | T | C | 0.015 | 0.338 | 0.0025 | 2.23E-09 | 400,102 | 36 |
| 171 | rs6806825 | T | C | -0.0148 | 0.351 | 0.0025 | 1.87E-09 | 400,102 | 35.0464 |
| 172 | rs6859730 | A | T | 0.0206 | 0.328 | 0.0025 | 8.76E-17 | 400,102 | 67.8976 |
| 173 | rs6867471 | T | C | 0.0133 | 0.363 | 0.0024 | 4.90E-08 | 400,102 | 30.7100694 |
| 174 | rs6886191 | A | G | -0.0152 | 0.442 | 0.0024 | 1.42E-10 | 400,102 | 40.1111111 |
| 175 | rs6938343 | T | C | 0.0158 | 0.232 | 0.0028 | 1.80E-08 | 400,102 | 31.8418367 |
| 176 | rs6949451 | T | C | 0.0133 | 0.461 | 0.0023 | 1.17E-08 | 400,102 | 33.4385633 |
| 177 | rs6956494 | T | C | 0.0131 | 0.403 | 0.0024 | 4.24E-08 | 400,102 | 29.7934028 |
| 178 | rs6968518 | T | C | 0.0186 | 0.434 | 0.0024 | 5.98E-15 | 400,102 | 60.0625 |
| 179 | rs7029491 | C | G | -0.0173 | 0.667 | 0.0025 | 5.79E-12 | 400,102 | 47.8864 |
| 180 | rs7041139 | T | C | -0.015 | 0.322 | 0.0025 | 2.23E-09 | 400,102 | 36 |
| 181 | rs715285 | A | G | 0.0185 | 0.542 | 0.0024 | 5.46E-15 | 400,102 | 59.4184028 |
| 182 | rs7182990 | A | G | -0.0168 | 0.494 | 0.0023 | 8.10E-13 | 400,102 | 53.3534972 |
| 183 | rs719615 | A | G | -0.0215 | 0.477 | 0.0024 | 7.02E-20 | 400,102 | 80.2517361 |
| 184 | rs7198383 | T | C | 0.0178 | 0.682 | 0.0025 | 1.73E-12 | 400,102 | 50.6944 |
| 185 | rs7203231 | T | G | 0.0129 | 0.511 | 0.0024 | 4.93E-08 | 400,102 | 28.890625 |
| 186 | rs7233091 | A | G | -0.0141 | 0.454 | 0.0023 | 1.78E-09 | 400,102 | 37.5822306 |
| 187 | rs723588 | T | C | -0.0235 | 0.857 | 0.0034 | 2.62E-12 | 400,102 | 47.7724913 |
| 188 | rs72776472 | A | G | 0.0213 | 0.766 | 0.0028 | 1.65E-14 | 400,102 | 57.8686224 |
| 189 | rs72845046 | A | T | -0.0376 | 0.115 | 0.0037 | 1.26E-24 | 400,102 | 103.26954 |
| 190 | rs732132 | T | C | -0.0332 | 0.258 | 0.0027 | 6.13E-35 | 400,102 | 151.198903 |
| 191 | rs736281 | T | C | 0.0144 | 0.4 | 0.0024 | 2.14E-09 | 400,102 | 36 |
| 192 | rs7414828 | A | G | -0.0337 | 0.0696 | 0.0046 | 2.14E-13 | 400,102 | 53.6715501 |
| 193 | rs7572476 | T | C | -0.0193 | 0.455 | 0.0024 | 2.96E-16 | 400,102 | 64.6684028 |
| 194 | rs76109497 | A | G | 0.0172 | 0.174 | 0.0031 | 3.45E-08 | 400,102 | 30.7845994 |
| 195 | rs7613360 | T | C | -0.0143 | 0.393 | 0.0024 | 3.40E-09 | 400,102 | 35.5017361 |
| 196 | rs76219171 | A | G | -0.0351 | 0.0601 | 0.005 | 2.09E-12 | 400,102 | 49.2804 |
| 197 | rs7648255 | A | T | -0.0148 | 0.268 | 0.0027 | 2.19E-08 | 400,102 | 30.0466392 |
| 198 | rs7738842 | C | G | 0.0156 | 0.231 | 0.0028 | 2.05E-08 | 400,102 | 31.0408163 |
| 199 | rs77862575 | T | C | -0.0349 | 0.964 | 0.0063 | 3.01E-08 | 400,102 | 30.6880826 |
| 200 | rs7806296 | A | G | -0.0177 | 0.36 | 0.0024 | 2.87E-13 | 400,102 | 54.390625 |
| 201 | rs78101726 | A | G | 0.0265 | 0.846 | 0.0033 | 4.61E-16 | 400,102 | 64.4857668 |
| 202 | rs7838717 | T | C | -0.0234 | 0.363 | 0.0025 | 6.47E-21 | 400,102 | 87.6096 |
| 203 | rs7943381 | A | G | -0.0158 | 0.745 | 0.0027 | 3.37E-09 | 400,102 | 34.2441701 |
| 204 | rs7946009 | T | C | 0.0165 | 0.341 | 0.0025 | 3.20E-11 | 400,102 | 43.56 |
| 205 | rs7949728 | C | G | 0.0185 | 0.59 | 0.0024 | 9.53E-15 | 400,102 | 59.4184028 |
| 206 | rs7977418 | T | C | 0.0379 | 0.541 | 0.0023 | 9.57E-59 | 400,102 | 271.533081 |
| 207 | rs8027022 | A | G | 0.017 | 0.494 | 0.0023 | 2.77E-13 | 400,102 | 54.63138 |
| 208 | rs803751 | T | C | -0.0246 | 0.345 | 0.0025 | 1.13E-23 | 400,102 | 96.8256 |
| 209 | rs8062719 | A | G | -0.0192 | 0.364 | 0.0024 | 3.19E-15 | 400,102 | 64 |
| 210 | rs8067252 | T | C | 0.023 | 0.229 | 0.0029 | 7.94E-16 | 400,102 | 62.901308 |
| 211 | rs8104651 | T | C | -0.0138 | 0.562 | 0.0024 | 4.91E-09 | 400,102 | 33.0625 |
| 212 | rs8125514 | A | G | -0.0292 | 0.676 | 0.0025 | 3.37E-31 | 400,102 | 136.4224 |
| 213 | rs8181164 | T | C | -0.0136 | 0.502 | 0.0024 | 9.29E-09 | 400,102 | 32.1111111 |
| 214 | rs878471 | A | G | -0.0278 | 0.58 | 0.0024 | 1.50E-31 | 400,102 | 134.173611 |
| 215 | rs891903 | A | G | -0.0233 | 0.254 | 0.0027 | 9.60E-18 | 400,102 | 74.4705075 |
| 216 | rs931794 | A | G | 0.0172 | 0.664 | 0.0025 | 4.60E-12 | 400,102 | 47.3344 |
| 217 | rs9321170 | A | G | 0.0153 | 0.725 | 0.0026 | 6.60E-09 | 400,102 | 34.6286982 |
| 218 | rs9435733 | T | C | -0.0231 | 0.482 | 0.0023 | 3.35E-23 | 400,102 | 100.871456 |
| 219 | rs9438626 | C | G | 0.0175 | 0.21 | 0.0028 | 8.06E-10 | 400,102 | 39.0625 |
| 220 | rs9472541 | A | T | -0.0154 | 0.285 | 0.0026 | 2.47E-09 | 400,102 | 35.0828402 |
| 221 | rs9643242 | A | G | 0.0189 | 0.154 | 0.0033 | 6.61E-09 | 400,102 | 32.8016529 |
| 222 | rs979012 | T | C | -0.026 | 0.363 | 0.0024 | 1.20E-26 | 400,102 | 117.361111 |
| 223 | rs9927855 | T | C | 0.0168 | 0.304 | 0.0026 | 7.69E-11 | 400,102 | 41.7514793 |
| 224 | rs9947743 | A | G | -0.0185 | 0.787 | 0.0029 | 1.13E-10 | 400,102 | 40.6956005 |
| 225 | rs9950661 | T | C | -0.0141 | 0.501 | 0.0023 | 1.47E-09 | 400,102 | 37.5822306 |

Table S3 Selected genetic instrumental variables of FVC
