# Supplementary material for: COPD, PRISm and lung function reduction affect the brain cortical structure: a Mendelian randomization study
Source: BMC Pulm Med. 2024 Jul 15;24:341. doi: 10.1186/s12890-024-03150-2 (PMC11251327; doi:10.1186/s12890-024-03150-2)
Supplement: Supplementary file 4 — Supplementary Material 4. [file 12890_2024_3150_MOESM4_ESM.docx]

|  | SNP | effect_allele.exposure | other_allele.exposure | beta.exposure | eaf.exposure | se.exposure | pval.exposure | samplesize | F |
| --- | --- | --- | --- | --- | --- | --- | --- | --- | --- |
| 1 | rs10059996 | T | G | -0.0352 | 0.355 | 0.0026 | 1.53E-42 | 400,102 | 183.289941 |
| 2 | rs10172023 | C | G | -0.0146 | 0.666 | 0.0025 | 7.46E-09 | 400,102 | 34.1056 |
| 3 | rs10400826 | A | G | -0.0192 | 0.189 | 0.003 | 1.92E-10 | 400,102 | 40.96 |
| 4 | rs10460533 | A | G | 0.0211 | 0.285 | 0.0026 | 5.93E-16 | 400,102 | 65.8594675 |
| 5 | rs1050785 | A | C | -0.0182 | 0.602 | 0.0025 | 3.00E-13 | 400,102 | 52.9984 |
| 6 | rs10508468 | T | C | 0.015 | 0.621 | 0.0024 | 8.70E-10 | 400,102 | 39.0625 |
| 7 | rs1054085 | T | C | -0.0155 | 0.376 | 0.0025 | 2.96E-10 | 400,102 | 38.44 |
| 8 | rs10748849 | T | C | 0.0178 | 0.393 | 0.0024 | 2.88E-13 | 400,102 | 55.0069444 |
| 9 | rs10775456 | A | G | 0.0141 | 0.319 | 0.0026 | 4.19E-08 | 400,102 | 29.4097633 |
| 10 | rs10831984 | A | G | -0.0234 | 0.546 | 0.0024 | 7.25E-23 | 400,102 | 95.0625 |
| 11 | rs10836366 | T | C | 0.0191 | 0.749 | 0.0027 | 2.33E-12 | 400,102 | 50.042524 |
| 12 | rs10851744 | T | C | -0.0197 | 0.867 | 0.0035 | 1.45E-08 | 400,102 | 31.6808163 |
| 13 | rs10904220 | A | G | 0.014 | 0.341 | 0.0025 | 2.19E-08 | 400,102 | 31.36 |
| 14 | rs10983184 | T | C | 0.0273 | 0.64 | 0.0025 | 9.05E-28 | 400,102 | 119.2464 |
| 15 | rs10987386 | T | C | -0.0259 | 0.187 | 0.0031 | 4.51E-17 | 400,102 | 69.8033299 |
| 16 | rs11000805 | C | G | -0.0223 | 0.187 | 0.0031 | 5.37E-13 | 400,102 | 51.7471384 |
| 17 | rs11042775 | A | G | 0.0145 | 0.356 | 0.0025 | 5.59E-09 | 400,102 | 33.64 |
| 18 | rs11067615 | T | G | -0.0162 | 0.487 | 0.0024 | 1.15E-11 | 400,102 | 45.5625 |
| 19 | rs11098196 | T | G | -0.0196 | 0.508 | 0.0024 | 2.42E-16 | 400,102 | 66.6944444 |
| 20 | rs111231071 | T | C | 0.0343 | 0.0717 | 0.0046 | 1.24E-13 | 400,102 | 55.5997164 |
| 21 | rs111628829 | A | G | 0.0335 | 0.0438 | 0.0058 | 7.70E-09 | 400,102 | 33.3605826 |
| 22 | rs11172113 | T | C | -0.0227 | 0.589 | 0.0024 | 7.04E-21 | 400,102 | 89.4600694 |
| 23 | rs11200633 | T | C | -0.0192 | 0.216 | 0.0029 | 3.94E-11 | 400,102 | 43.8335315 |
| 24 | rs112009052 | A | T | 0.143 | 0.014 | 0.0104 | 3.82E-43 | 400,102 | 189.0625 |
| 25 | rs11234768 | T | C | 0.0303 | 0.846 | 0.0033 | 5.07E-20 | 400,102 | 84.3057851 |
| 26 | rs112731178 | T | C | 0.04 | 0.0958 | 0.0041 | 5.76E-23 | 400,102 | 95.1814396 |
| 27 | rs113336697 | T | C | 0.0262 | 0.0887 | 0.0042 | 7.01E-10 | 400,102 | 38.9138322 |
| 28 | rs115503647 | A | G | 0.038 | 0.948 | 0.0053 | 1.20E-12 | 400,102 | 51.4061944 |
| 29 | rs1155612 | T | C | 0.0285 | 0.491 | 0.0024 | 6.50E-33 | 400,102 | 141.015625 |
| 30 | rs115830429 | A | G | 0.0586 | 0.0237 | 0.008 | 1.93E-13 | 400,102 | 53.655625 |
| 31 | rs11596235 | T | C | -0.0143 | 0.309 | 0.0026 | 3.47E-08 | 400,102 | 30.25 |
| 32 | rs11620380 | A | C | -0.027 | 0.105 | 0.0039 | 4.60E-12 | 400,102 | 47.9289941 |
| 33 | rs11637595 | T | C | 0.0174 | 0.273 | 0.0027 | 8.24E-11 | 400,102 | 41.5308642 |
| 34 | rs11648508 | T | G | 0.0332 | 0.683 | 0.0026 | 9.86E-39 | 400,102 | 163.053254 |
| 35 | rs11653958 | A | G | 0.0197 | 0.743 | 0.0028 | 9.17E-13 | 400,102 | 49.5012755 |
| 36 | rs117014247 | T | C | -0.0445 | 0.024 | 0.0079 | 2.09E-08 | 400,102 | 31.7296908 |
| 37 | rs11709963 | T | C | 0.0178 | 0.837 | 0.0032 | 3.68E-08 | 400,102 | 30.9414063 |
| 38 | rs11738281 | T | C | 0.0183 | 0.799 | 0.003 | 7.85E-10 | 400,102 | 37.21 |
| 39 | rs11755954 | T | C | 0.0203 | 0.156 | 0.0033 | 5.47E-10 | 400,102 | 37.8411387 |
| 40 | rs11771259 | C | G | 0.0275 | 0.881 | 0.0036 | 5.03E-14 | 400,102 | 58.3526235 |
| 41 | rs117827273 | C | G | 0.0262 | 0.85 | 0.0033 | 4.92E-15 | 400,102 | 63.0339761 |
| 42 | rs11824553 | A | C | -0.0247 | 0.0815 | 0.0044 | 1.68E-08 | 400,102 | 31.5129132 |
| 43 | rs11888819 | A | C | -0.0255 | 0.726 | 0.0027 | 6.87E-22 | 400,102 | 89.1975309 |
| 44 | rs1192415 | A | G | 0.044 | 0.812 | 0.003 | 2.28E-47 | 400,102 | 215.111111 |
| 45 | rs12023835 | T | C | 0.02 | 0.602 | 0.0024 | 2.55E-16 | 400,102 | 69.4444444 |
| 46 | rs12034227 | A | C | 0.0248 | 0.664 | 0.0025 | 3.81E-23 | 400,102 | 98.4064 |
| 47 | rs12134019 | T | C | 0.0311 | 0.14 | 0.0035 | 2.44E-19 | 400,102 | 78.9559184 |
| 48 | rs12134534 | T | C | -0.0249 | 0.409 | 0.0024 | 6.96E-25 | 400,102 | 107.640625 |
| 49 | rs12189242 | A | C | 0.0185 | 0.247 | 0.0027 | 1.21E-11 | 400,102 | 46.9478738 |
| 50 | rs1219514 | A | G | 0.0251 | 0.0789 | 0.0044 | 1.47E-08 | 400,102 | 32.5418388 |
| 51 | rs12202314 | T | C | -0.0209 | 0.675 | 0.0025 | 2.17E-16 | 400,102 | 69.8896 |
| 52 | rs1233553 | A | G | -0.0286 | 0.934 | 0.0051 | 2.82E-08 | 400,102 | 31.4479047 |
| 53 | rs12402871 | T | C | -0.017 | 0.194 | 0.003 | 1.75E-08 | 400,102 | 32.1111111 |
| 54 | rs1245035 | A | C | -0.014 | 0.626 | 0.0025 | 1.61E-08 | 400,102 | 31.36 |
| 55 | rs12470864 | A | G | -0.0203 | 0.385 | 0.0024 | 1.04E-16 | 400,102 | 71.5434028 |
| 56 | rs12522114 | A | C | -0.0367 | 0.265 | 0.0027 | 1.47E-41 | 400,102 | 184.758573 |
| 57 | rs12525532 | T | C | 0.015 | 0.37 | 0.0025 | 1.07E-09 | 400,102 | 36 |
| 58 | rs12575589 | T | C | 0.0205 | 0.121 | 0.0037 | 2.68E-08 | 400,102 | 30.6975895 |
| 59 | rs12607689 | T | G | -0.0158 | 0.591 | 0.0024 | 6.83E-11 | 400,102 | 43.3402778 |
| 60 | rs12609373 | T | C | 0.0172 | 0.776 | 0.0029 | 1.83E-09 | 400,102 | 35.17717 |
| 61 | rs12627254 | T | G | 0.0357 | 0.128 | 0.0035 | 6.85E-24 | 400,102 | 104.04 |
| 62 | rs12635856 | T | C | -0.0187 | 0.173 | 0.0032 | 3.59E-09 | 400,102 | 34.1494141 |
| 63 | rs12698403 | A | G | -0.0236 | 0.442 | 0.0024 | 1.48E-22 | 400,102 | 96.6944444 |
| 64 | rs12705390 | A | G | -0.0173 | 0.201 | 0.003 | 6.18E-09 | 400,102 | 33.2544444 |
| 65 | rs12715478 | A | G | 0.025 | 0.594 | 0.0024 | 1.35E-24 | 400,102 | 108.506944 |
| 66 | rs1274475 | A | G | 0.0168 | 0.392 | 0.0025 | 8.30E-12 | 400,102 | 45.1584 |
| 67 | rs12781987 | T | G | -0.0201 | 0.217 | 0.0029 | 5.88E-12 | 400,102 | 48.039239 |
| 68 | rs12875005 | T | G | -0.0182 | 0.681 | 0.0025 | 7.86E-13 | 400,102 | 52.9984 |
| 69 | rs12918140 | C | G | -0.027 | 0.115 | 0.0038 | 6.72E-13 | 400,102 | 50.4847645 |
| 70 | rs12935657 | A | G | 0.0154 | 0.247 | 0.0027 | 1.89E-08 | 400,102 | 32.5322359 |
| 71 | rs1293994 | A | G | 0.0154 | 0.442 | 0.0024 | 1.80E-10 | 400,102 | 41.1736111 |
| 72 | rs12941356 | A | G | 0.0139 | 0.413 | 0.0024 | 8.74E-09 | 400,102 | 33.5434028 |
| 73 | rs1294417 | T | C | -0.0311 | 0.458 | 0.0024 | 3.93E-39 | 400,102 | 167.918403 |
| 74 | rs13030866 | T | C | -0.015 | 0.351 | 0.0025 | 1.95E-09 | 400,102 | 36 |
| 75 | rs13069228 | T | C | 0.0138 | 0.639 | 0.0025 | 3.20E-08 | 400,102 | 30.4704 |
| 76 | rs13199674 | A | G | -0.0137 | 0.443 | 0.0024 | 1.34E-08 | 400,102 | 32.5850694 |
| 77 | rs13332450 | T | C | -0.0385 | 0.925 | 0.0046 | 3.55E-17 | 400,102 | 70.0496219 |
| 78 | rs13361953 | T | C | 0.0412 | 0.664 | 0.0025 | 6.45E-60 | 400,102 | 271.5904 |
| 79 | rs1391805 | A | G | -0.019 | 0.761 | 0.0028 | 7.54E-12 | 400,102 | 46.0459184 |
| 80 | rs141942982 | T | G | -0.0362 | 0.106 | 0.0039 | 9.57E-21 | 400,102 | 86.156476 |
| 81 | rs1424393 | C | G | 0.015 | 0.622 | 0.0024 | 8.11E-10 | 400,102 | 39.0625 |
| 82 | rs1429555 | T | C | 0.0508 | 0.0689 | 0.0047 | 2.10E-27 | 400,102 | 116.823902 |
| 83 | rs1441207 | C | G | 0.0181 | 0.26 | 0.0027 | 2.94E-11 | 400,102 | 44.9396433 |
| 84 | rs1441358 | T | G | 0.0642 | 0.664 | 0.0025 | 4.12E-145 | 400,102 | 659.4624 |
| 85 | rs1452909 | A | G | -0.0185 | 0.251 | 0.0028 | 2.17E-11 | 400,102 | 43.6543367 |
| 86 | rs146612591 | C | G | -0.052 | 0.977 | 0.008 | 8.86E-11 | 400,102 | 42.25 |
| 87 | rs1528628 | A | G | -0.0142 | 0.455 | 0.0024 | 2.39E-09 | 400,102 | 35.0069444 |
| 88 | rs1529672 | A | C | 0.0423 | 0.174 | 0.0031 | 1.73E-41 | 400,102 | 186.190427 |
| 89 | rs1561073 | A | T | 0.0201 | 0.734 | 0.0027 | 6.43E-14 | 400,102 | 55.4197531 |
| 90 | rs1566834 | T | C | 0.015 | 0.662 | 0.0025 | 2.18E-09 | 400,102 | 36 |
| 91 | rs1570203 | A | G | 0.0246 | 0.53 | 0.0024 | 5.78E-25 | 400,102 | 105.0625 |
| 92 | rs1593438 | A | C | -0.0171 | 0.652 | 0.0025 | 1.11E-11 | 400,102 | 46.7856 |
| 93 | rs163694 | T | C | 0.0166 | 0.461 | 0.0024 | 4.98E-12 | 400,102 | 47.8402778 |
| 94 | rs16909922 | A | G | 0.0514 | 0.905 | 0.0041 | 3.31E-36 | 400,102 | 157.165973 |
| 95 | rs17129265 | T | C | 0.0195 | 0.167 | 0.0032 | 1.57E-09 | 400,102 | 37.1337891 |
| 96 | rs17513531 | T | C | 0.0162 | 0.344 | 0.0025 | 1.25E-10 | 400,102 | 41.9904 |
| 97 | rs1756281 | A | G | 0.0237 | 0.699 | 0.0026 | 1.38E-19 | 400,102 | 83.0902367 |
| 98 | rs17666332 | T | G | 0.0267 | 0.724 | 0.0027 | 9.22E-24 | 400,102 | 97.7901235 |
| 99 | rs17681353 | A | G | 0.0353 | 0.961 | 0.0062 | 9.41E-09 | 400,102 | 32.4164932 |
| 100 | rs177392 | T | C | 0.0137 | 0.595 | 0.0025 | 3.84E-08 | 400,102 | 30.0304 |
| 101 | rs1799807 | T | C | 0.0598 | 0.981 | 0.0088 | 8.59E-12 | 400,102 | 46.1782025 |
| 102 | rs1849496 | A | C | 0.0343 | 0.0616 | 0.005 | 4.96E-12 | 400,102 | 47.0596 |
| 103 | rs1855971 | A | G | 0.0191 | 0.835 | 0.0032 | 2.74E-09 | 400,102 | 35.6259766 |
| 104 | rs1896797 | A | G | 0.0292 | 0.49 | 0.0024 | 2.48E-34 | 400,102 | 148.027778 |
| 105 | rs1928160 | A | G | -0.0296 | 0.515 | 0.0024 | 5.78E-36 | 400,102 | 152.111111 |
| 106 | rs193686 | T | C | -0.0178 | 0.684 | 0.0026 | 4.07E-12 | 400,102 | 46.8698225 |
| 107 | rs1956028 | T | C | 0.0306 | 0.874 | 0.0036 | 2.37E-17 | 400,102 | 72.25 |
| 108 | rs2003254 | A | T | 0.0152 | 0.717 | 0.0027 | 1.09E-08 | 400,102 | 31.6927298 |
| 109 | rs201191 | A | C | 0.0221 | 0.486 | 0.0024 | 1.55E-20 | 400,102 | 84.7934028 |
| 110 | rs2012453 | A | G | 0.0239 | 0.411 | 0.0024 | 4.26E-23 | 400,102 | 99.1684028 |
| 111 | rs2021956 | A | G | 0.0167 | 0.811 | 0.003 | 3.68E-08 | 400,102 | 30.9877778 |
| 112 | rs2027761 | T | C | 0.0369 | 0.113 | 0.0038 | 1.31E-22 | 400,102 | 94.2943213 |
| 113 | rs2077177 | T | C | 0.0187 | 0.591 | 0.0024 | 1.21E-14 | 400,102 | 60.7100694 |
| 114 | rs2084448 | T | C | 0.0197 | 0.706 | 0.0026 | 4.65E-14 | 400,102 | 57.4097633 |
| 115 | rs2168721 | A | G | -0.0198 | 0.388 | 0.0024 | 6.89E-16 | 400,102 | 68.0625 |
| 116 | rs2173982 | C | G | -0.015 | 0.673 | 0.0025 | 2.81E-09 | 400,102 | 36 |
| 117 | rs2244592 | A | G | -0.0323 | 0.453 | 0.0024 | 4.60E-42 | 400,102 | 181.126736 |
| 118 | rs227728 | A | T | -0.0171 | 0.334 | 0.0025 | 1.20E-11 | 400,102 | 46.7856 |
| 119 | rs2285261 | A | C | 0.0191 | 0.384 | 0.0024 | 4.90E-15 | 400,102 | 63.3350694 |
| 120 | rs2297291 | A | G | -0.0136 | 0.408 | 0.0024 | 2.14E-08 | 400,102 | 32.1111111 |
| 121 | rs2302643 | A | G | -0.0193 | 0.444 | 0.0024 | 1.89E-15 | 400,102 | 64.6684028 |
| 122 | rs2324154 | A | C | 0.0157 | 0.509 | 0.0024 | 2.52E-11 | 400,102 | 42.7934028 |
| 123 | rs2333570 | C | G | -0.017 | 0.25 | 0.0027 | 5.62E-10 | 400,102 | 39.6433471 |
| 124 | rs2452995 | T | C | -0.0167 | 0.309 | 0.0026 | 9.66E-11 | 400,102 | 41.2559172 |
| 125 | rs2533889 | C | G | 0.0163 | 0.29 | 0.0026 | 4.44E-10 | 400,102 | 39.3032544 |
| 126 | rs2543350 | T | C | -0.0202 | 0.859 | 0.0034 | 4.27E-09 | 400,102 | 35.2975779 |
| 127 | rs2544536 | T | C | -0.0239 | 0.488 | 0.0024 | 4.15E-24 | 400,102 | 99.1684028 |
| 128 | rs2571445 | A | G | -0.0199 | 0.397 | 0.0024 | 3.08E-16 | 400,102 | 68.7517361 |
| 129 | rs2579762 | A | C | 0.0205 | 0.527 | 0.0024 | 9.26E-18 | 400,102 | 72.9600694 |
| 130 | rs2609280 | A | G | 0.0537 | 0.215 | 0.0029 | 1.83E-76 | 400,102 | 342.888228 |
| 131 | rs2687199 | A | G | -0.0188 | 0.676 | 0.0026 | 1.93E-13 | 400,102 | 52.2840237 |
| 132 | rs274995 | T | C | 0.0163 | 0.58 | 0.0024 | 1.47E-11 | 400,102 | 46.1267361 |
| 133 | rs2760751 | A | G | 0.0191 | 0.272 | 0.0027 | 6.30E-13 | 400,102 | 50.042524 |
| 134 | rs2762969 | C | G | 0.0151 | 0.287 | 0.0026 | 7.77E-09 | 400,102 | 33.7292899 |
| 135 | rs2794360 | A | G | 0.033 | 0.104 | 0.0039 | 2.27E-17 | 400,102 | 71.5976331 |
| 136 | rs2798641 | T | C | -0.045 | 0.183 | 0.0031 | 3.89E-48 | 400,102 | 210.718002 |
| 137 | rs2802544 | T | C | -0.0162 | 0.279 | 0.0027 | 1.81E-09 | 400,102 | 36 |
| 138 | rs2834457 | A | T | 0.0247 | 0.622 | 0.0024 | 5.16E-24 | 400,102 | 105.918403 |
| 139 | rs28490195 | T | C | 0.0142 | 0.663 | 0.0025 | 1.88E-08 | 400,102 | 32.2624 |
| 140 | rs28787109 | A | G | 0.0193 | 0.399 | 0.0024 | 1.29E-15 | 400,102 | 64.6684028 |
| 141 | rs2999089 | C | G | 0.0434 | 0.119 | 0.0037 | 3.92E-32 | 400,102 | 137.58656 |
| 142 | rs301127 | A | T | -0.0195 | 0.799 | 0.003 | 5.34E-11 | 400,102 | 42.25 |
| 143 | rs330939 | T | G | 0.0232 | 0.621 | 0.0025 | 4.46E-21 | 400,102 | 86.1184 |
| 144 | rs335651 | T | C | 0.0146 | 0.58 | 0.0024 | 1.61E-09 | 400,102 | 37.0069444 |
| 145 | rs34624010 | A | G | 0.0243 | 0.266 | 0.0027 | 2.06E-19 | 400,102 | 81 |
| 146 | rs34712979 | A | G | -0.0682 | 0.256 | 0.0028 | 4.18E-134 | 400,102 | 593.270408 |
| 147 | rs35107139 | A | C | 0.0315 | 0.596 | 0.0025 | 3.40E-36 | 400,102 | 158.76 |
| 148 | rs35195586 | T | G | -0.0408 | 0.0662 | 0.0048 | 1.77E-17 | 400,102 | 72.25 |
| 149 | rs35246838 | T | C | 0.0386 | 0.868 | 0.0036 | 1.40E-27 | 400,102 | 114.966049 |
| 150 | rs35320030 | T | C | 0.0183 | 0.832 | 0.0032 | 8.40E-09 | 400,102 | 32.7041016 |
| 151 | rs35420030 | T | C | -0.045 | 0.947 | 0.0053 | 3.08E-17 | 400,102 | 72.0897116 |
| 152 | rs35495115 | A | T | 0.0206 | 0.865 | 0.0035 | 2.82E-09 | 400,102 | 34.6416327 |
| 153 | rs35506173 | T | C | 0.0256 | 0.275 | 0.0027 | 1.79E-21 | 400,102 | 89.8984911 |
| 154 | rs36049816 | T | C | -0.0189 | 0.376 | 0.0024 | 1.21E-14 | 400,102 | 62.015625 |
| 155 | rs3732986 | A | G | 0.0157 | 0.462 | 0.0024 | 3.35E-11 | 400,102 | 42.7934028 |
| 156 | rs374597 | T | C | 0.0225 | 0.477 | 0.0024 | 2.38E-21 | 400,102 | 87.890625 |
| 157 | rs3752243 | A | G | 0.0136 | 0.57 | 0.0024 | 1.27E-08 | 400,102 | 32.1111111 |
| 158 | rs3754512 | T | C | -0.039 | 0.523 | 0.0024 | 4.35E-61 | 400,102 | 264.0625 |
| 159 | rs377731 | A | G | -0.0185 | 0.379 | 0.0025 | 5.13E-14 | 400,102 | 54.76 |
| 160 | rs3779505 | A | G | 0.0277 | 0.101 | 0.0039 | 1.73E-12 | 400,102 | 50.4464168 |
| 161 | rs4099470 | T | C | 0.0287 | 0.0545 | 0.0053 | 4.94E-08 | 400,102 | 29.3232467 |
| 162 | rs4247294 | A | G | -0.0135 | 0.44 | 0.0024 | 1.58E-08 | 400,102 | 31.640625 |
| 163 | rs4308141 | C | G | -0.0484 | 0.801 | 0.003 | 3.58E-59 | 400,102 | 260.284444 |
| 164 | rs4353138 | T | C | 0.0146 | 0.456 | 0.0024 | 7.61E-10 | 400,102 | 37.0069444 |
| 165 | rs442217 | C | G | -0.0148 | 0.332 | 0.0025 | 4.54E-09 | 400,102 | 35.0464 |
| 166 | rs4438257 | T | G | 0.0288 | 0.869 | 0.0035 | 2.18E-16 | 400,102 | 67.7093878 |
| 167 | rs4478547 | T | G | 0.0194 | 0.353 | 0.0025 | 8.00E-15 | 400,102 | 60.2176 |
| 168 | rs4721457 | T | C | 0.0244 | 0.849 | 0.0033 | 1.73E-13 | 400,102 | 54.6703398 |
| 169 | rs4848578 | T | C | -0.0173 | 0.788 | 0.0029 | 2.83E-09 | 400,102 | 35.587396 |
| 170 | rs4854343 | A | G | 0.018 | 0.828 | 0.0031 | 8.16E-09 | 400,102 | 33.7148803 |
| 171 | rs4884168 | A | G | 0.0139 | 0.639 | 0.0025 | 2.57E-08 | 400,102 | 30.9136 |
| 172 | rs4886509 | A | C | 0.0251 | 0.669 | 0.0025 | 6.10E-23 | 400,102 | 100.8016 |
| 173 | rs4982712 | A | G | -0.0189 | 0.397 | 0.0024 | 6.52E-15 | 400,102 | 62.015625 |
| 174 | rs5027392 | A | G | 0.0147 | 0.389 | 0.0025 | 2.11E-09 | 400,102 | 34.5744 |
| 175 | rs55703445 | T | C | -0.0275 | 0.219 | 0.0029 | 1.70E-21 | 400,102 | 89.9227111 |
| 176 | rs55884799 | T | C | -0.0415 | 0.827 | 0.0031 | 4.01E-40 | 400,102 | 179.21436 |
| 177 | rs55966786 | A | G | -0.029 | 0.0723 | 0.0046 | 3.85E-10 | 400,102 | 39.7448015 |
| 178 | rs56077333 | A | C | -0.0143 | 0.324 | 0.0026 | 2.22E-08 | 400,102 | 30.25 |
| 179 | rs567508 | A | G | 0.0224 | 0.842 | 0.0033 | 1.58E-11 | 400,102 | 46.0752984 |
| 180 | rs56919232 | T | C | -0.0176 | 0.786 | 0.0029 | 1.63E-09 | 400,102 | 36.8323424 |
| 181 | rs57347370 | T | G | 0.016 | 0.257 | 0.0027 | 4.97E-09 | 400,102 | 35.1165981 |
| 182 | rs5748428 | T | G | 0.0178 | 0.222 | 0.0028 | 3.80E-10 | 400,102 | 40.4132653 |
| 183 | rs5758000 | T | C | 0.0152 | 0.645 | 0.0025 | 7.30E-10 | 400,102 | 36.9664 |
| 184 | rs58095595 | A | G | 0.0192 | 0.172 | 0.0032 | 2.11E-09 | 400,102 | 36 |
| 185 | rs58769314 | T | C | 0.0174 | 0.734 | 0.0027 | 1.14E-10 | 400,102 | 41.5308642 |
| 186 | rs594476 | C | G | 0.0182 | 0.707 | 0.0026 | 5.50E-12 | 400,102 | 49 |
| 187 | rs60568503 | A | G | 0.0247 | 0.376 | 0.0025 | 1.27E-23 | 400,102 | 97.6144 |
| 188 | rs6059938 | A | G | 0.0159 | 0.486 | 0.0024 | 1.87E-11 | 400,102 | 43.890625 |
| 189 | rs61147355 | A | G | 0.0284 | 0.141 | 0.0034 | 1.54E-16 | 400,102 | 69.7716263 |
| 190 | rs6119231 | A | G | -0.0237 | 0.169 | 0.0032 | 6.30E-14 | 400,102 | 54.8525391 |
| 191 | rs6132863 | A | G | -0.0175 | 0.18 | 0.0031 | 1.33E-08 | 400,102 | 31.867846 |
| 192 | rs6134919 | T | C | -0.0137 | 0.416 | 0.0024 | 9.46E-09 | 400,102 | 32.5850694 |
| 193 | rs61603681 | A | T | 0.0172 | 0.349 | 0.0025 | 3.18E-12 | 400,102 | 47.3344 |
| 194 | rs61764202 | T | C | 0.0164 | 0.327 | 0.0025 | 1.10E-10 | 400,102 | 43.0336 |
| 195 | rs61963203 | T | G | -0.0517 | 0.979 | 0.0084 | 9.46E-10 | 400,102 | 37.8810941 |
| 196 | rs62092156 | A | G | 0.0254 | 0.0942 | 0.0041 | 4.06E-10 | 400,102 | 38.379536 |
| 197 | rs62201738 | A | C | -0.0743 | 0.922 | 0.0044 | 9.45E-63 | 400,102 | 285.149277 |
| 198 | rs62283793 | T | C | 0.034 | 0.053 | 0.0053 | 2.07E-10 | 400,102 | 41.1534354 |
| 199 | rs62289340 | T | C | 0.0167 | 0.436 | 0.0024 | 2.36E-12 | 400,102 | 48.4184028 |
| 200 | rs62316310 | A | G | 0.0272 | 0.26 | 0.0027 | 2.24E-23 | 400,102 | 101.486968 |
| 201 | rs62321845 | A | G | 0.0229 | 0.806 | 0.003 | 3.33E-14 | 400,102 | 58.2677778 |
| 202 | rs62394997 | A | G | 0.0298 | 0.0598 | 0.0051 | 4.90E-09 | 400,102 | 34.142253 |
| 203 | rs631126 | T | C | -0.0256 | 0.25 | 0.0027 | 7.24E-21 | 400,102 | 89.8984911 |
| 204 | rs6421437 | T | G | -0.0179 | 0.363 | 0.0025 | 1.73E-12 | 400,102 | 51.2656 |
| 205 | rs6433079 | A | C | -0.0223 | 0.761 | 0.0028 | 1.50E-15 | 400,102 | 63.4298469 |
| 206 | rs6470085 | A | G | 0.0142 | 0.464 | 0.0024 | 2.88E-09 | 400,102 | 35.0069444 |
| 207 | rs659398 | T | C | -0.0254 | 0.272 | 0.0027 | 5.08E-21 | 400,102 | 88.4993141 |
| 208 | rs6676141 | T | C | -0.0264 | 0.795 | 0.003 | 4.17E-19 | 400,102 | 77.44 |
| 209 | rs6722557 | C | G | -0.0204 | 0.256 | 0.0027 | 8.05E-14 | 400,102 | 57.0864198 |
| 210 | rs674621 | T | C | 0.0161 | 0.681 | 0.0026 | 3.02E-10 | 400,102 | 38.3446746 |
| 211 | rs6769798 | A | G | 0.0194 | 0.832 | 0.0033 | 4.20E-09 | 400,102 | 34.5601469 |
| 212 | rs6809164 | T | G | 0.0232 | 0.729 | 0.0027 | 5.26E-18 | 400,102 | 73.8326475 |
| 213 | rs6880851 | T | G | 0.0253 | 0.189 | 0.0031 | 1.45E-16 | 400,102 | 66.6066597 |
| 214 | rs7046050 | C | G | -0.015 | 0.709 | 0.0026 | 1.41E-08 | 400,102 | 33.2840237 |
| 215 | rs7048006 | T | C | 0.0141 | 0.587 | 0.0024 | 7.00E-09 | 400,102 | 34.515625 |
| 216 | rs7093310 | T | C | 0.034 | 0.902 | 0.004 | 2.14E-17 | 400,102 | 72.25 |
| 217 | rs7118465 | T | C | -0.0249 | 0.681 | 0.0026 | 2.09E-22 | 400,102 | 91.7174556 |
| 218 | rs7139311 | T | C | 0.0152 | 0.535 | 0.0024 | 1.45E-10 | 400,102 | 40.1111111 |
| 219 | rs7155530 | A | G | -0.0144 | 0.398 | 0.0024 | 2.61E-09 | 400,102 | 36 |
| 220 | rs7162245 | A | G | -0.0159 | 0.354 | 0.0025 | 1.72E-10 | 400,102 | 40.4496 |
| 221 | rs72651496 | A | G | -0.0169 | 0.263 | 0.0027 | 2.79E-10 | 400,102 | 39.1783265 |
| 222 | rs72673442 | T | C | 0.0517 | 0.951 | 0.0055 | 8.80E-21 | 400,102 | 88.36 |
| 223 | rs72692823 | T | C | -0.0205 | 0.851 | 0.0034 | 9.35E-10 | 400,102 | 36.3538062 |
| 224 | rs72907878 | A | G | -0.0334 | 0.134 | 0.0035 | 1.96E-21 | 400,102 | 91.0661224 |
| 225 | rs7307510 | T | C | 0.0439 | 0.187 | 0.0031 | 1.44E-46 | 400,102 | 200.542144 |
| 226 | rs73158393 | C | G | -0.0205 | 0.743 | 0.0027 | 3.56E-14 | 400,102 | 57.6474623 |
| 227 | rs73158411 | A | G | 0.023 | 0.368 | 0.0025 | 1.41E-20 | 400,102 | 84.64 |
| 228 | rs73182224 | A | G | -0.0197 | 0.239 | 0.0029 | 2.01E-11 | 400,102 | 46.1462545 |
| 229 | rs74001718 | A | T | 0.0336 | 0.956 | 0.0058 | 8.23E-09 | 400,102 | 33.5600476 |
| 230 | rs7401714 | A | G | 0.0161 | 0.426 | 0.0024 | 1.68E-11 | 400,102 | 45.0017361 |
| 231 | rs7426380 | A | G | -0.0216 | 0.551 | 0.0024 | 2.01E-19 | 400,102 | 81 |
| 232 | rs751859 | A | T | 0.0242 | 0.21 | 0.0029 | 8.77E-17 | 400,102 | 69.6361474 |
| 233 | rs755249 | T | C | -0.0239 | 0.233 | 0.0028 | 9.82E-18 | 400,102 | 72.8584184 |
| 234 | rs7555534 | T | C | 0.0174 | 0.631 | 0.0025 | 1.71E-12 | 400,102 | 48.4416 |
| 235 | rs7565460 | T | C | 0.0144 | 0.45 | 0.0024 | 1.15E-09 | 400,102 | 36 |
| 236 | rs75928020 | A | G | -0.0173 | 0.219 | 0.0029 | 1.70E-09 | 400,102 | 35.587396 |
| 237 | rs7634731 | A | G | -0.0237 | 0.736 | 0.0027 | 2.18E-18 | 400,102 | 77.0493827 |
| 238 | rs764688 | T | C | -0.0151 | 0.286 | 0.0026 | 8.34E-09 | 400,102 | 33.7292899 |
| 239 | rs76525968 | T | G | -0.0175 | 0.268 | 0.0027 | 8.03E-11 | 400,102 | 42.0096022 |
| 240 | rs77059113 | T | G | -0.0306 | 0.927 | 0.0046 | 1.80E-11 | 400,102 | 44.2514178 |
| 241 | rs7713065 | A | C | -0.0285 | 0.265 | 0.0027 | 8.21E-26 | 400,102 | 111.419753 |
| 242 | rs7733410 | A | G | 0.0505 | 0.441 | 0.0024 | 1.56E-96 | 400,102 | 442.751736 |
| 243 | rs7753012 | T | G | -0.0712 | 0.695 | 0.0026 | 4.71E-165 | 400,102 | 749.91716 |
| 244 | rs7784940 | T | G | 0.0174 | 0.796 | 0.0029 | 3.16E-09 | 400,102 | 36 |
| 245 | rs78227763 | T | C | 0.047 | 0.0315 | 0.0069 | 8.65E-12 | 400,102 | 46.3978156 |
| 246 | rs78442819 | C | G | -0.0355 | 0.2 | 0.0031 | 2.25E-31 | 400,102 | 131.139438 |
| 247 | rs7896518 | A | G | 0.0161 | 0.573 | 0.0024 | 3.71E-11 | 400,102 | 45.0017361 |
| 248 | rs7974933 | A | G | -0.023 | 0.857 | 0.0034 | 2.54E-11 | 400,102 | 45.7612457 |
| 249 | rs79898473 | T | C | -0.0308 | 0.671 | 0.0026 | 2.31E-33 | 400,102 | 140.331361 |
| 250 | rs8039637 | T | C | -0.0264 | 0.137 | 0.0035 | 2.99E-14 | 400,102 | 56.8946939 |
| 251 | rs8068952 | C | G | -0.0285 | 0.784 | 0.0029 | 1.21E-22 | 400,102 | 96.5814507 |
| 252 | rs8080108 | T | C | 0.0158 | 0.689 | 0.0026 | 1.00E-09 | 400,102 | 36.9289941 |
| 253 | rs8089099 | A | G | 0.0235 | 0.275 | 0.0027 | 1.52E-18 | 400,102 | 75.7544582 |
| 254 | rs811075 | C | G | 0.0144 | 0.351 | 0.0025 | 9.21E-09 | 400,102 | 33.1776 |
| 255 | rs838327 | A | G | 0.0151 | 0.75 | 0.0028 | 4.29E-08 | 400,102 | 29.0829082 |
| 256 | rs840467 | T | C | 0.0301 | 0.188 | 0.0031 | 1.62E-22 | 400,102 | 94.2778356 |
| 257 | rs864354 | T | G | -0.0142 | 0.434 | 0.0024 | 3.53E-09 | 400,102 | 35.0069444 |
| 258 | rs879394 | T | G | -0.0298 | 0.235 | 0.0028 | 4.71E-26 | 400,102 | 113.270408 |
| 259 | rs898614 | T | C | 0.0151 | 0.648 | 0.0025 | 1.56E-09 | 400,102 | 36.4816 |
| 260 | rs911930 | T | G | -0.0167 | 0.306 | 0.0026 | 1.30E-10 | 400,102 | 41.2559172 |
| 261 | rs9295562 | T | C | -0.0159 | 0.306 | 0.0026 | 5.38E-10 | 400,102 | 37.397929 |
| 262 | rs9321207 | A | G | -0.0153 | 0.419 | 0.0024 | 2.40E-10 | 400,102 | 40.640625 |
| 263 | rs9333118 | A | C | -0.0275 | 0.094 | 0.004 | 1.05E-11 | 400,102 | 47.265625 |
| 264 | rs9351958 | C | G | 0.0345 | 0.202 | 0.003 | 3.23E-31 | 400,102 | 132.25 |
| 265 | rs9403133 | A | G | -0.017 | 0.242 | 0.0028 | 1.51E-09 | 400,102 | 36.8622449 |
| 266 | rs941747 | T | C | -0.0139 | 0.468 | 0.0024 | 4.56E-09 | 400,102 | 33.5434028 |
| 267 | rs943613 | T | C | -0.0132 | 0.446 | 0.0024 | 3.30E-08 | 400,102 | 30.25 |
| 268 | rs9533803 | T | C | -0.0261 | 0.213 | 0.0029 | 2.92E-19 | 400,102 | 81 |
| 269 | rs9600261 | A | G | 0.0207 | 0.184 | 0.0031 | 2.11E-11 | 400,102 | 44.5879292 |
| 270 | rs9634470 | T | C | -0.0208 | 0.735 | 0.0027 | 2.73E-14 | 400,102 | 59.3470508 |
| 271 | rs9636166 | A | C | 0.0355 | 0.874 | 0.0036 | 3.66E-23 | 400,102 | 97.2415123 |
| 272 | rs9661687 | T | C | -0.0271 | 0.863 | 0.0035 | 6.11E-15 | 400,102 | 59.9518367 |
| 273 | rs979455 | T | C | 0.0173 | 0.675 | 0.0025 | 9.22E-12 | 400,102 | 47.8864 |
| 274 | rs9850438 | T | C | -0.019 | 0.828 | 0.0032 | 2.06E-09 | 400,102 | 35.2539063 |
| 275 | rs985256 | A | C | 0.0179 | 0.218 | 0.0029 | 5.86E-10 | 400,102 | 38.098692 |
| 276 | rs987068 | C | G | -0.0296 | 0.689 | 0.0026 | 1.46E-30 | 400,102 | 129.609467 |
| 277 | rs9874847 | T | C | 0.0278 | 0.143 | 0.0034 | 6.04E-16 | 400,102 | 66.8546713 |
| 278 | rs988799 | C | G | -0.0288 | 0.793 | 0.0029 | 9.89E-23 | 400,102 | 98.6254459 |
| 279 | rs9888193 | T | G | -0.0148 | 0.727 | 0.0026 | 2.42E-08 | 400,102 | 32.4023669 |
| 280 | rs9905278 | T | C | -0.0208 | 0.492 | 0.0024 | 2.90E-18 | 400,102 | 75.1111111 |
| 281 | rs9912501 | T | C | 0.0239 | 0.522 | 0.0024 | 6.07E-24 | 400,102 | 99.1684028 |
| 282 | rs995758 | T | C | 0.0707 | 0.398 | 0.0024 | 1.48E-185 | 400,102 | 867.793403 |
| 283 | rs996865 | T | C | -0.0475 | 0.0752 | 0.0046 | 1.82E-25 | 400,102 | 106.628072 |
| 284 | rs9970286 | A | G | 0.0236 | 0.329 | 0.0025 | 1.92E-20 | 400,102 | 89.1136 |

Table S4 Selected genetic instrumental variables of FEV1/FVC
