# Supplementary figures and images for: COPD, PRISm and lung function reduction affect the brain cortical structure: a Mendelian randomization study (part 2 of 2)
Source: BMC Pulm Med. 2024 Jul 15;24:341. doi: 10.1186/s12890-024-03150-2 (PMC11251327; doi:10.1186/s12890-024-03150-2)

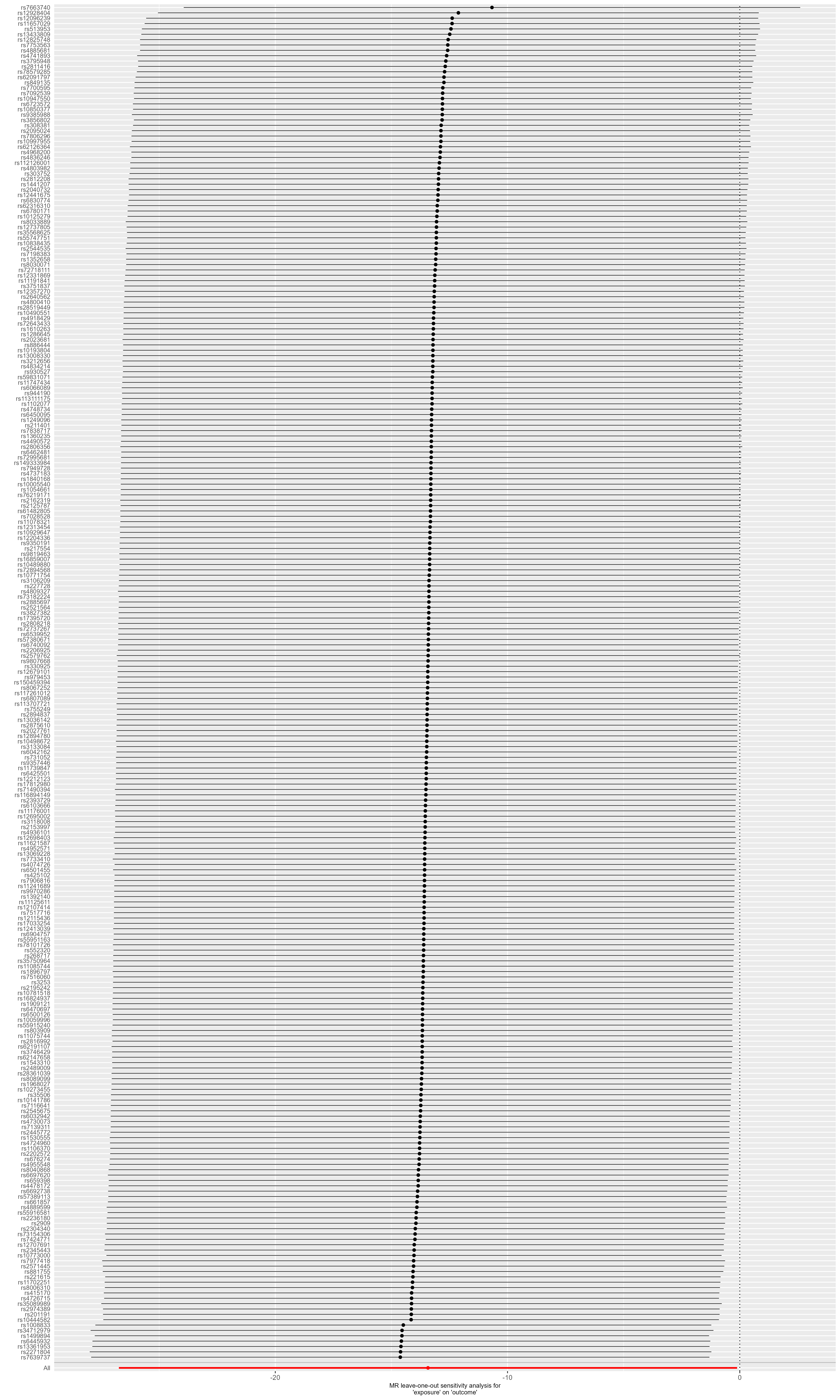

Supplement: Supplementary file 12 — Supplementary Material 12. [file 12890_2024_3150_MOESM12_ESM.zip › Supplementary Figure/leave-one-out analysis/Cortex Surface area/LOOA_FEV1_pericalcarine_surfavg.png]

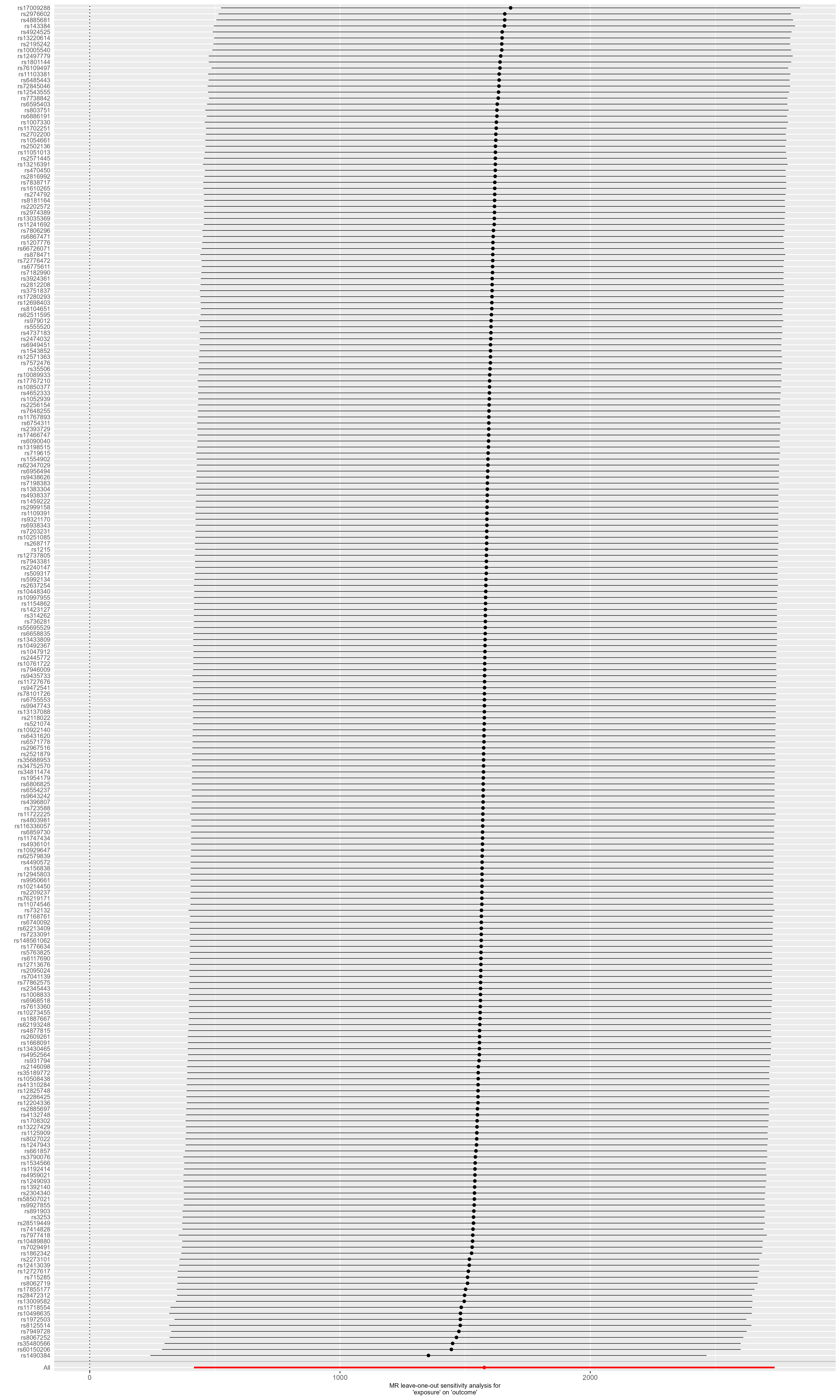

Supplement: Supplementary file 12 — Supplementary Material 12. [file 12890_2024_3150_MOESM12_ESM.zip › Supplementary Figure/leave-one-out analysis/Cortex Surface area/LOOA_FVC_Full_SurfArea.png]

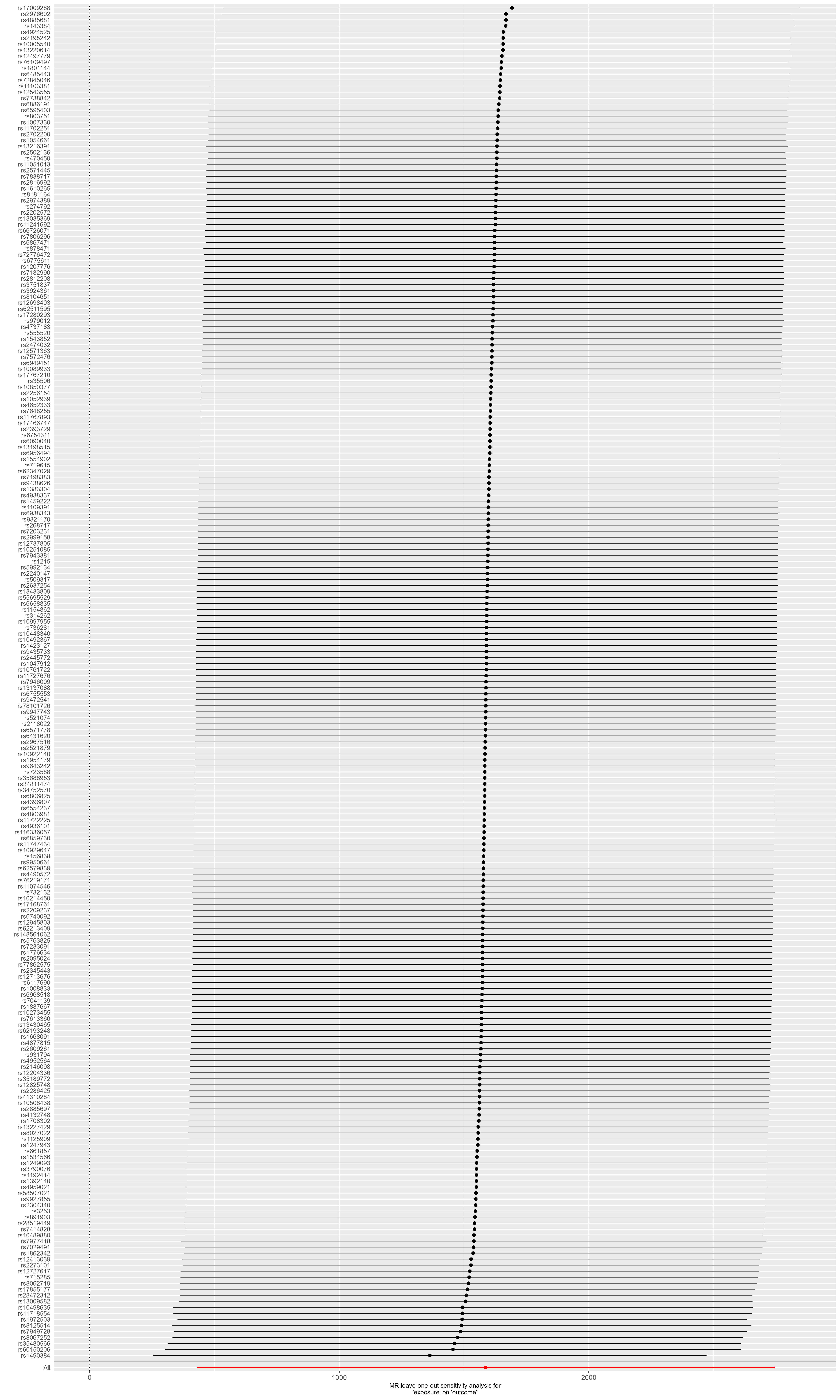

Supplement: Supplementary file 12 — Supplementary Material 12. [file 12890_2024_3150_MOESM12_ESM.zip › Supplementary Figure/leave-one-out analysis/Cortex Surface area/LOOA_FVC_Full_SurfArea_noGC.png]

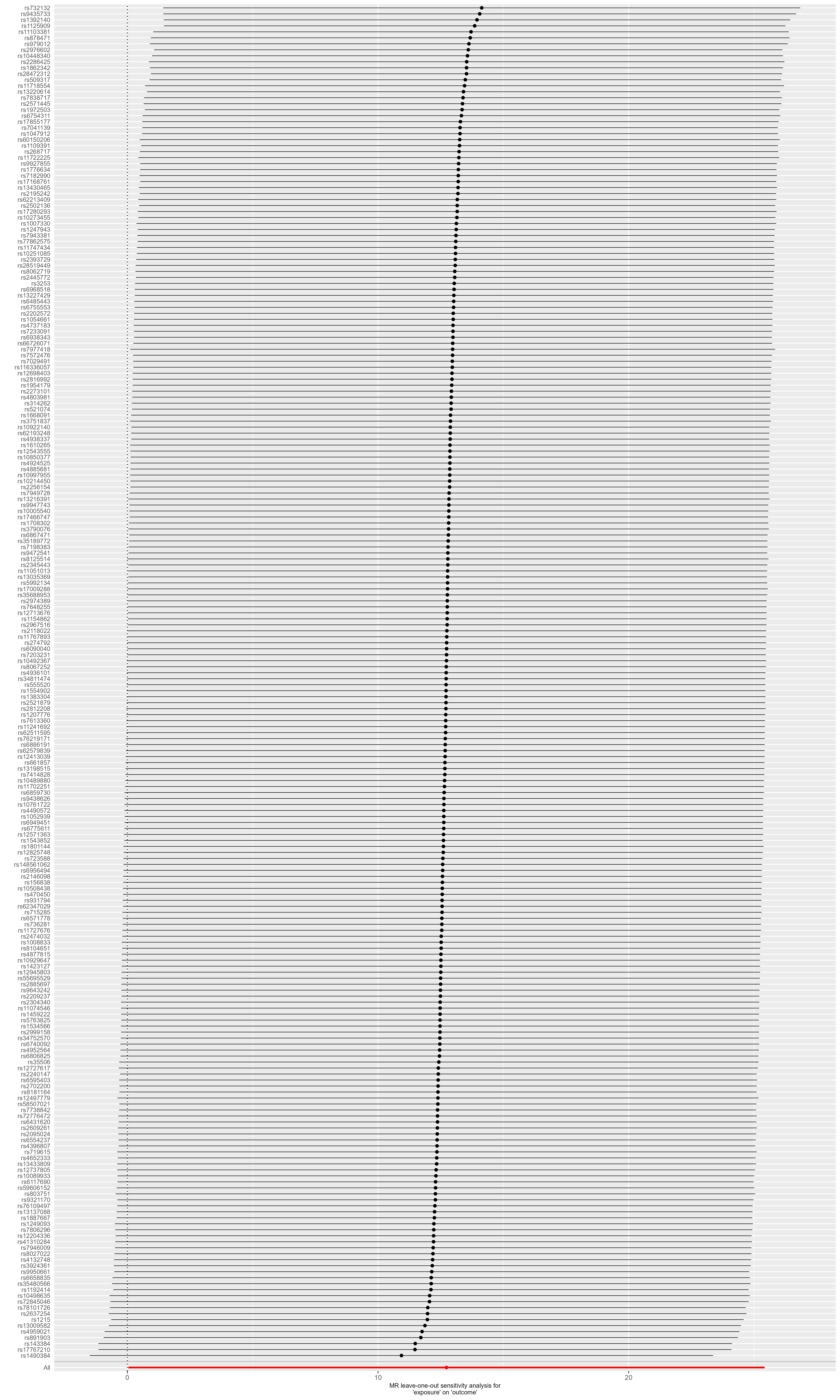

Supplement: Supplementary file 12 — Supplementary Material 12. [file 12890_2024_3150_MOESM12_ESM.zip › Supplementary Figure/leave-one-out analysis/Cortex Surface area/LOOA_FVC_insula_surfavg_noGC.png]

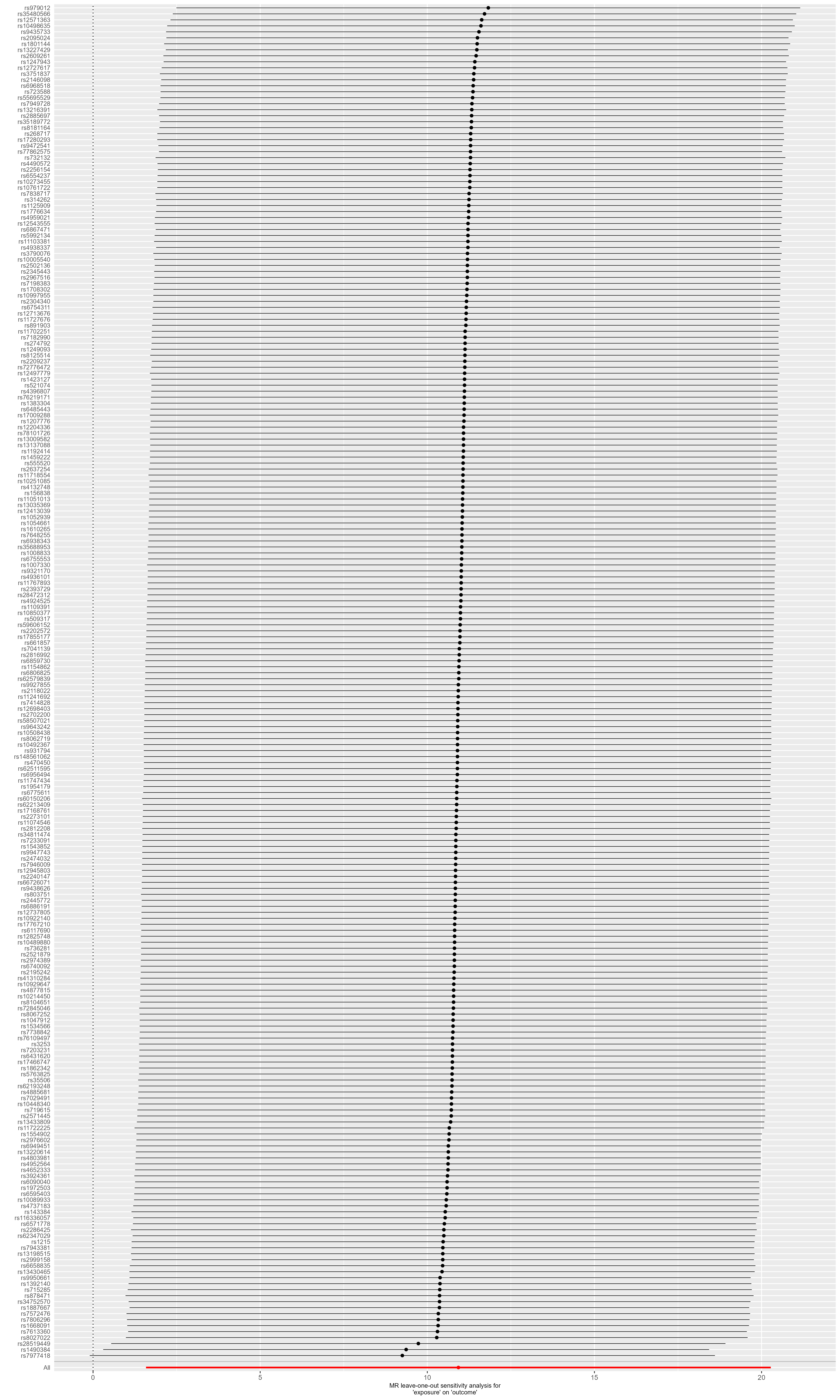

Supplement: Supplementary file 12 — Supplementary Material 12. [file 12890_2024_3150_MOESM12_ESM.zip › Supplementary Figure/leave-one-out analysis/Cortex Surface area/LOOA_FVC_medialorbitofrontal_surfavg.png]

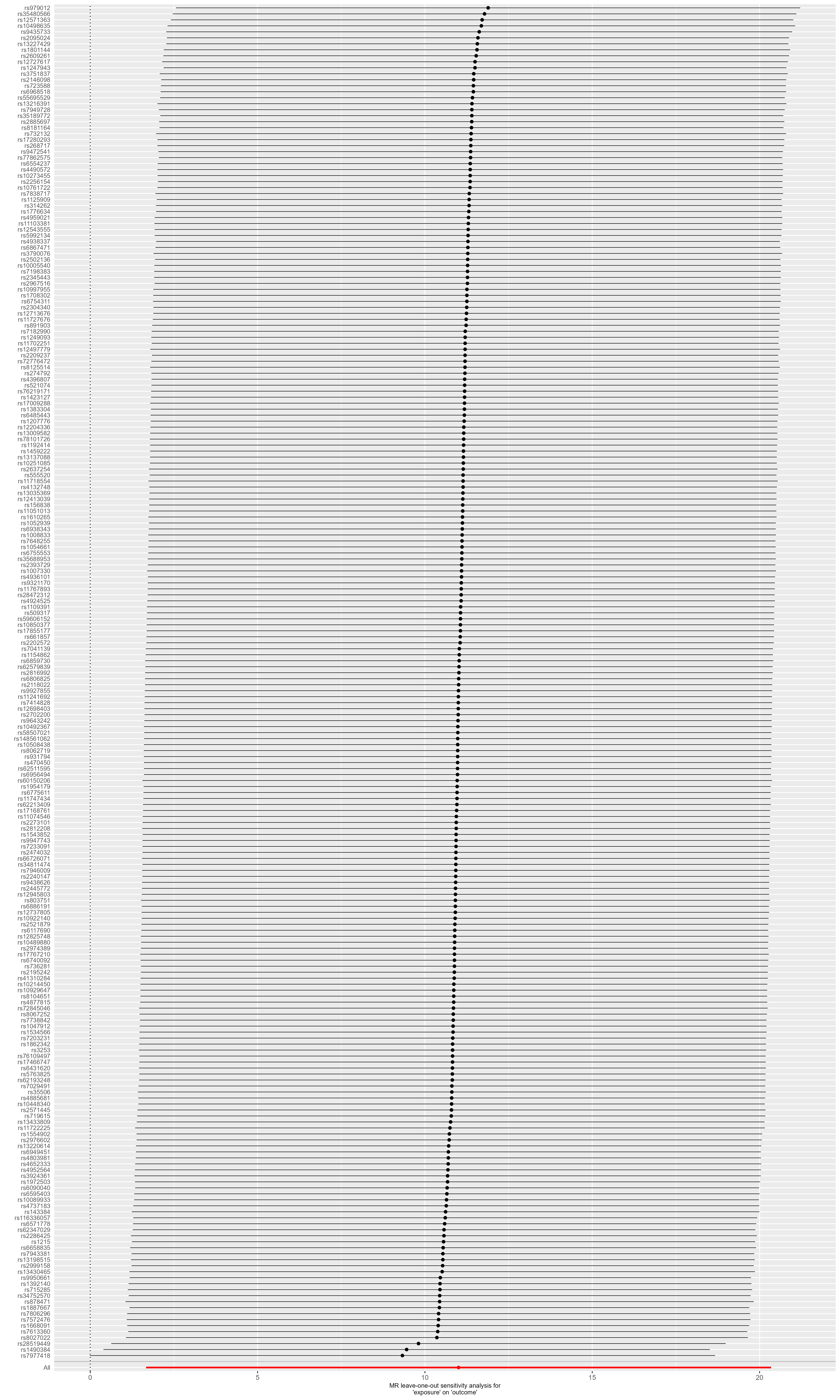

Supplement: Supplementary file 12 — Supplementary Material 12. [file 12890_2024_3150_MOESM12_ESM.zip › Supplementary Figure/leave-one-out analysis/Cortex Surface area/LOOA_FVC_medialorbitofrontal_surfavg_noGC.png]

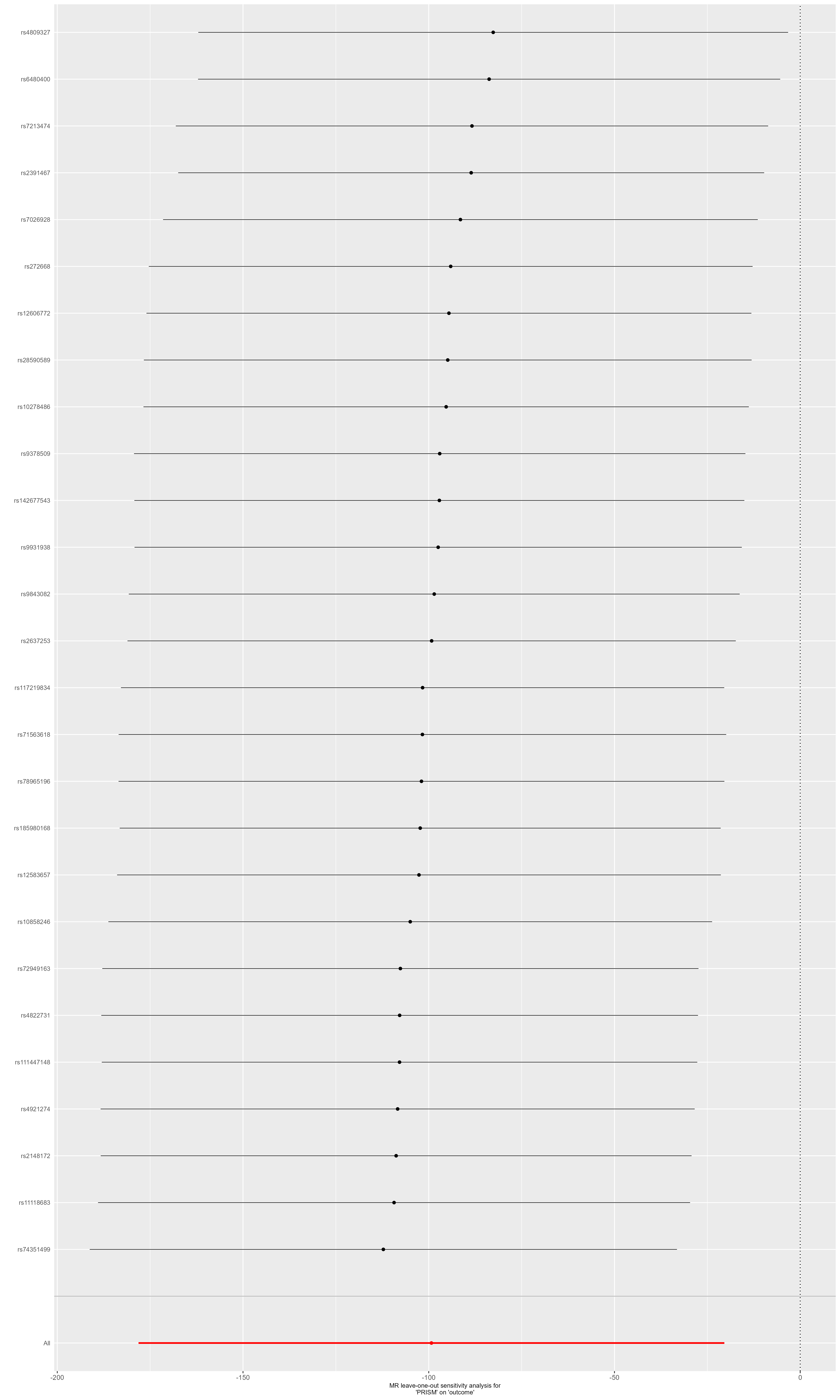

Supplement: Supplementary file 12 — Supplementary Material 12. [file 12890_2024_3150_MOESM12_ESM.zip › Supplementary Figure/leave-one-out analysis/Cortex Surface area/LOOA_PRISM_paracentral_surfavg_GC.png]

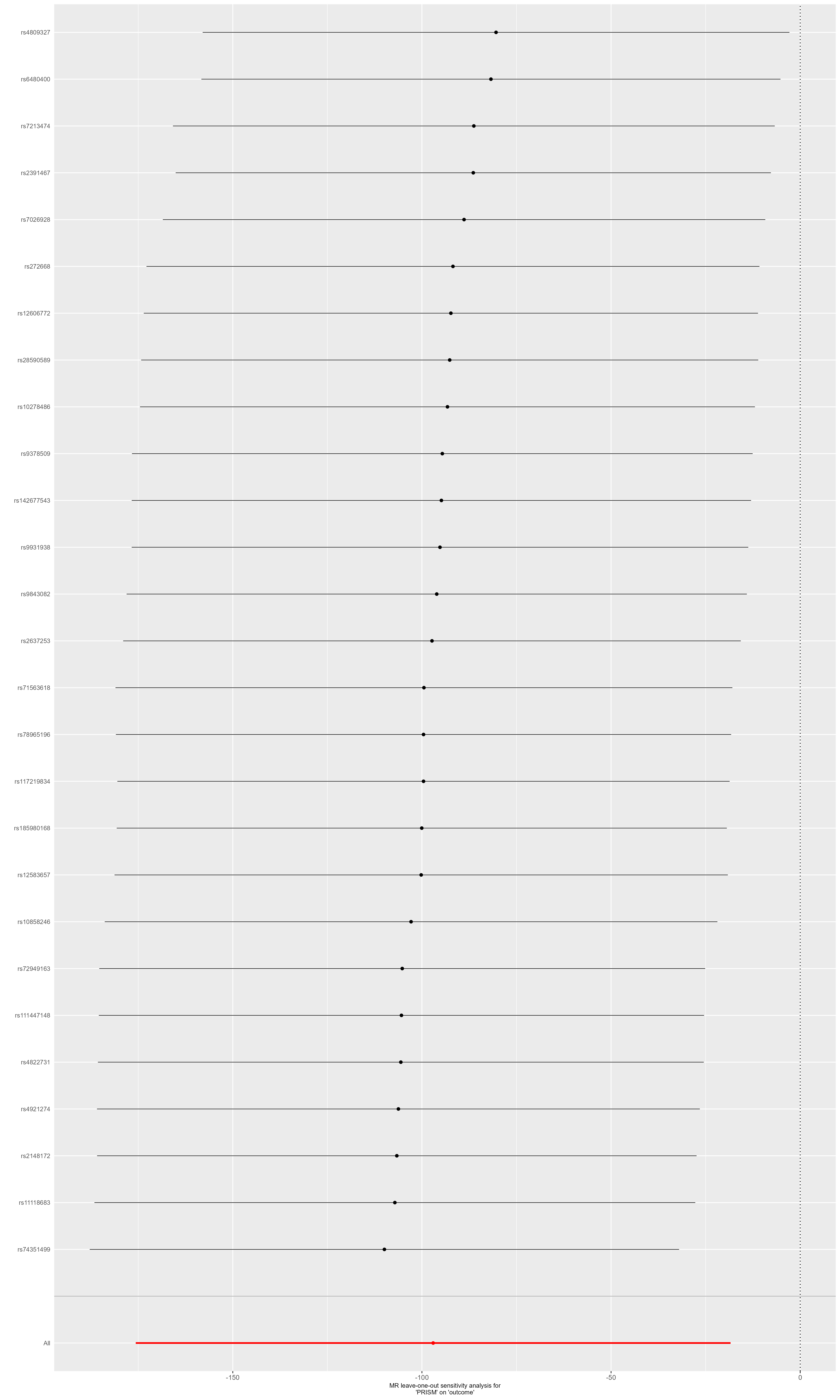

Supplement: Supplementary file 12 — Supplementary Material 12. [file 12890_2024_3150_MOESM12_ESM.zip › Supplementary Figure/leave-one-out analysis/Cortex Surface area/LOOA_PRISM_paracentral_surfavg_noGC.png]

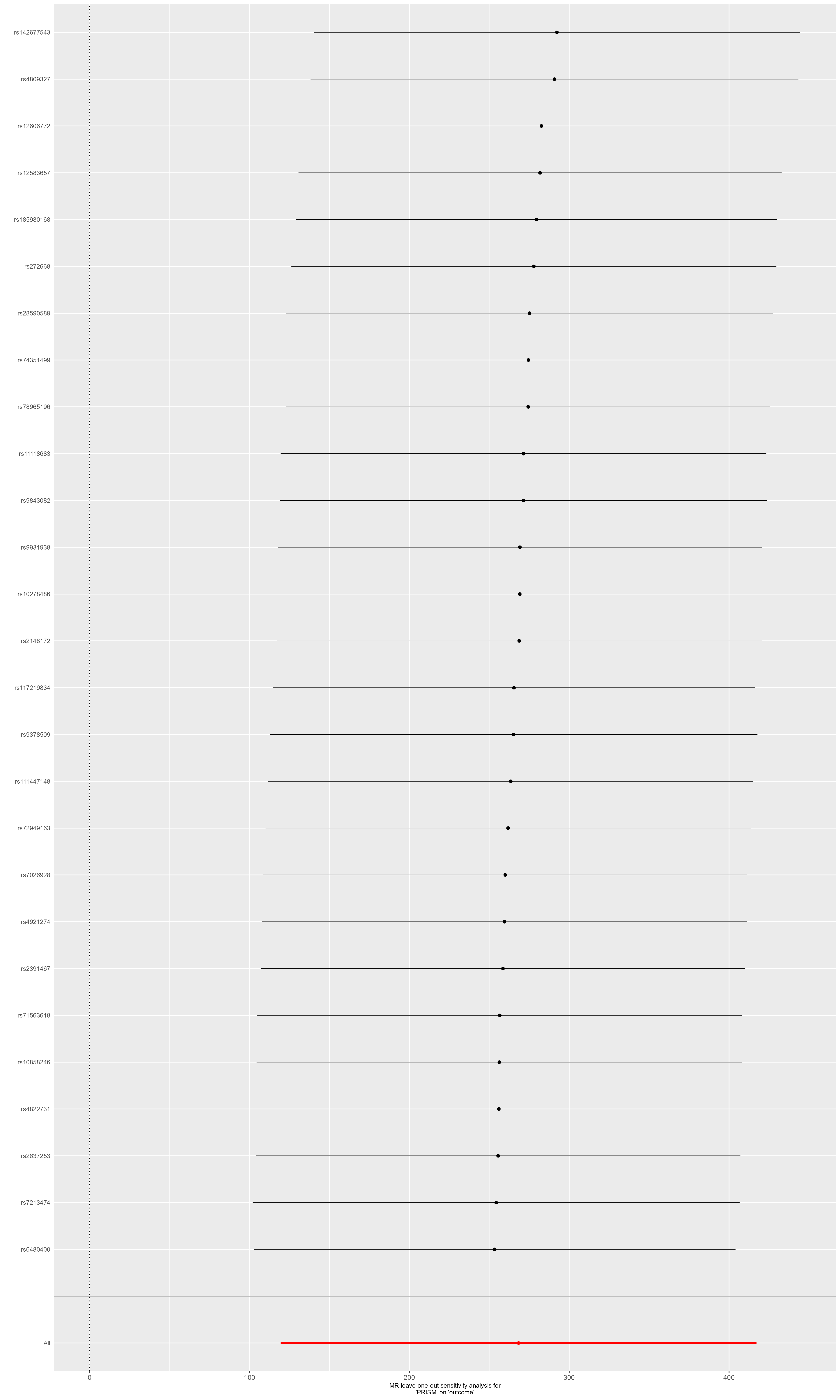

Supplement: Supplementary file 12 — Supplementary Material 12. [file 12890_2024_3150_MOESM12_ESM.zip › Supplementary Figure/leave-one-out analysis/Cortex Surface area/LOOA_PRISM_precuneus_surfavg_GC.png]

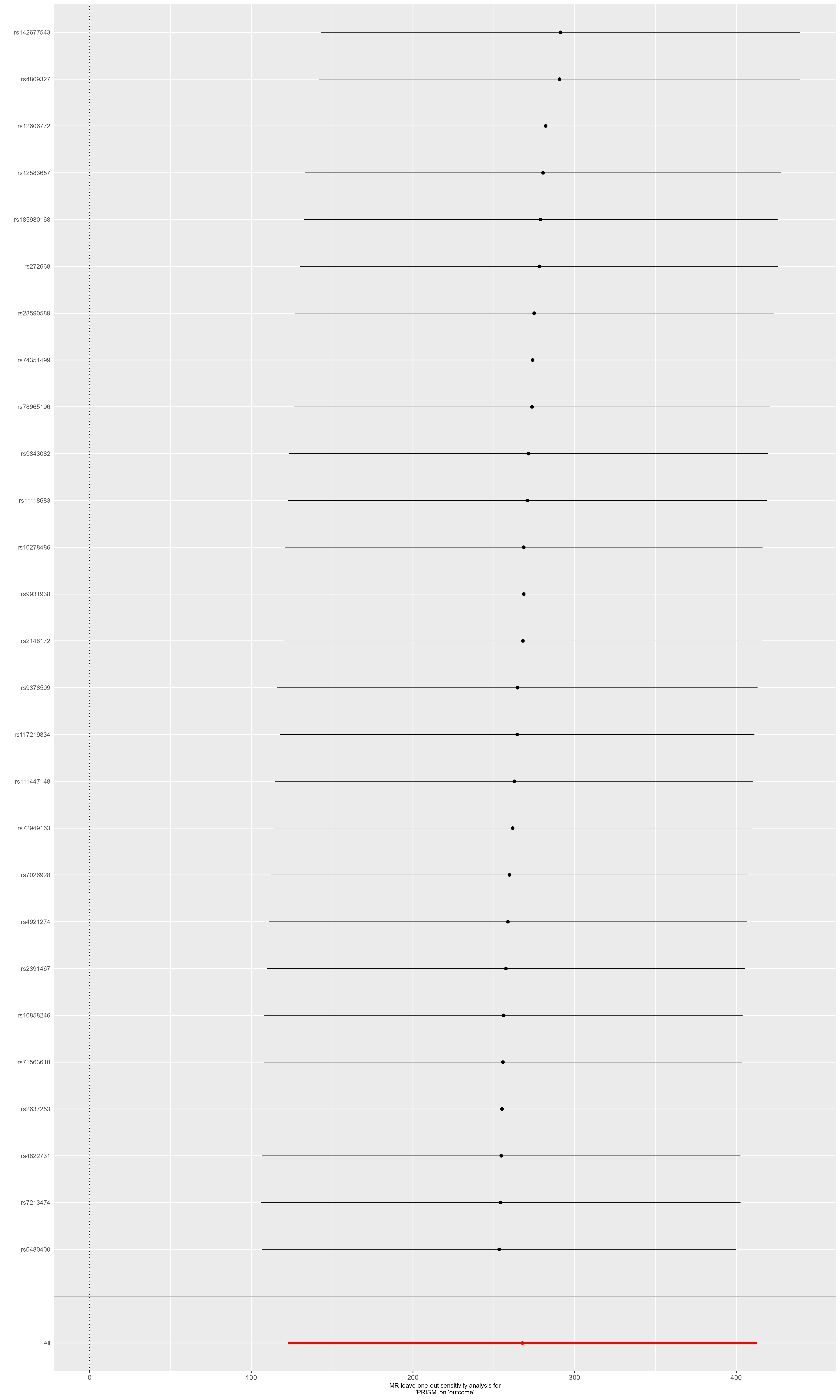

Supplement: Supplementary file 12 — Supplementary Material 12. [file 12890_2024_3150_MOESM12_ESM.zip › Supplementary Figure/leave-one-out analysis/Cortex Surface area/LOOA_PRISM_precuneus_surfavg_noGC.png]

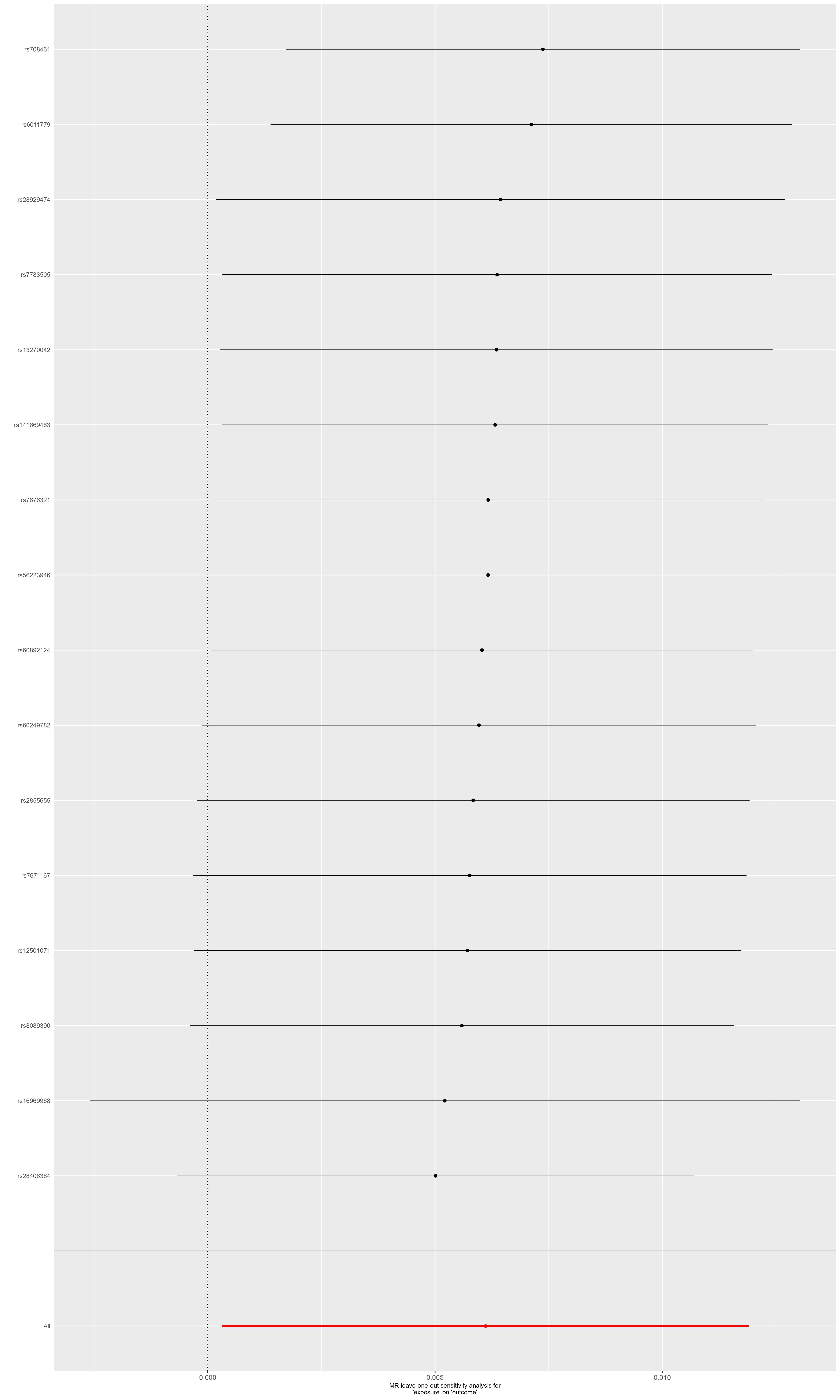

Supplement: Supplementary file 12 — Supplementary Material 12. [file 12890_2024_3150_MOESM12_ESM.zip › Supplementary Figure/leave-one-out analysis/Cortex Thickness/LOOA_COPD_cuneus_thickavg.png]

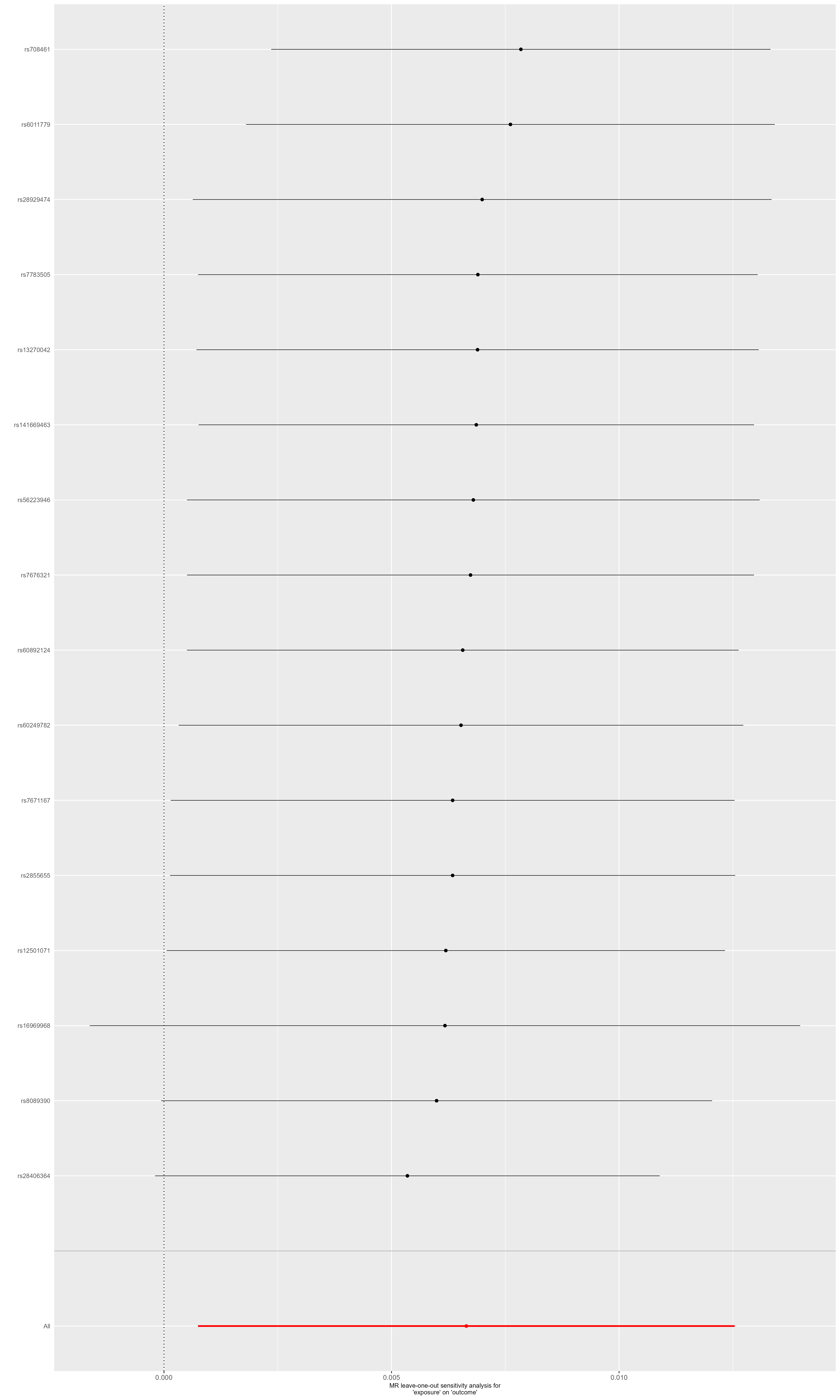

Supplement: Supplementary file 12 — Supplementary Material 12. [file 12890_2024_3150_MOESM12_ESM.zip › Supplementary Figure/leave-one-out analysis/Cortex Thickness/LOOA_COPD_cuneus_thickavg_noGC.png]

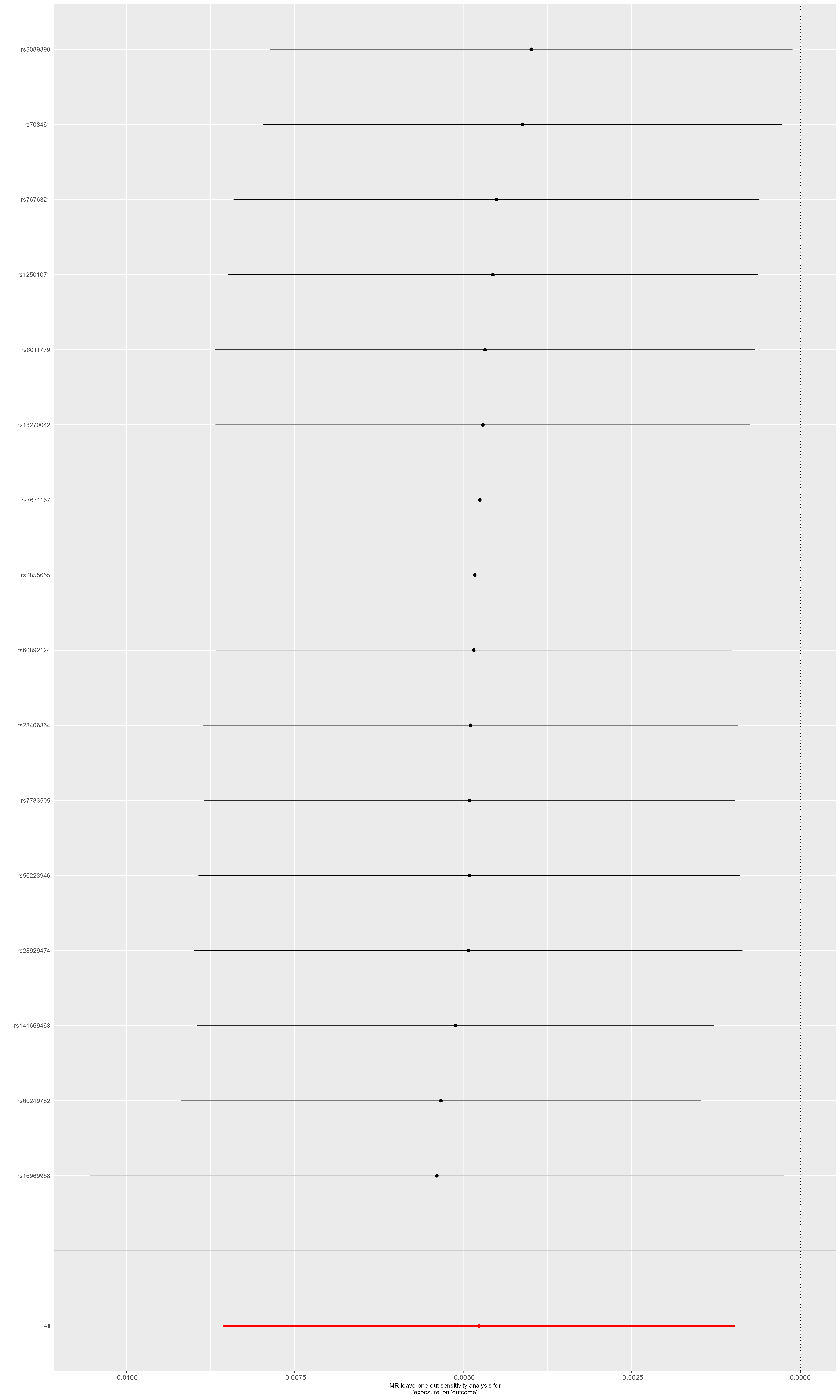

Supplement: Supplementary file 12 — Supplementary Material 12. [file 12890_2024_3150_MOESM12_ESM.zip › Supplementary Figure/leave-one-out analysis/Cortex Thickness/LOOA_COPD_inferiorparietal_thickavg.png]

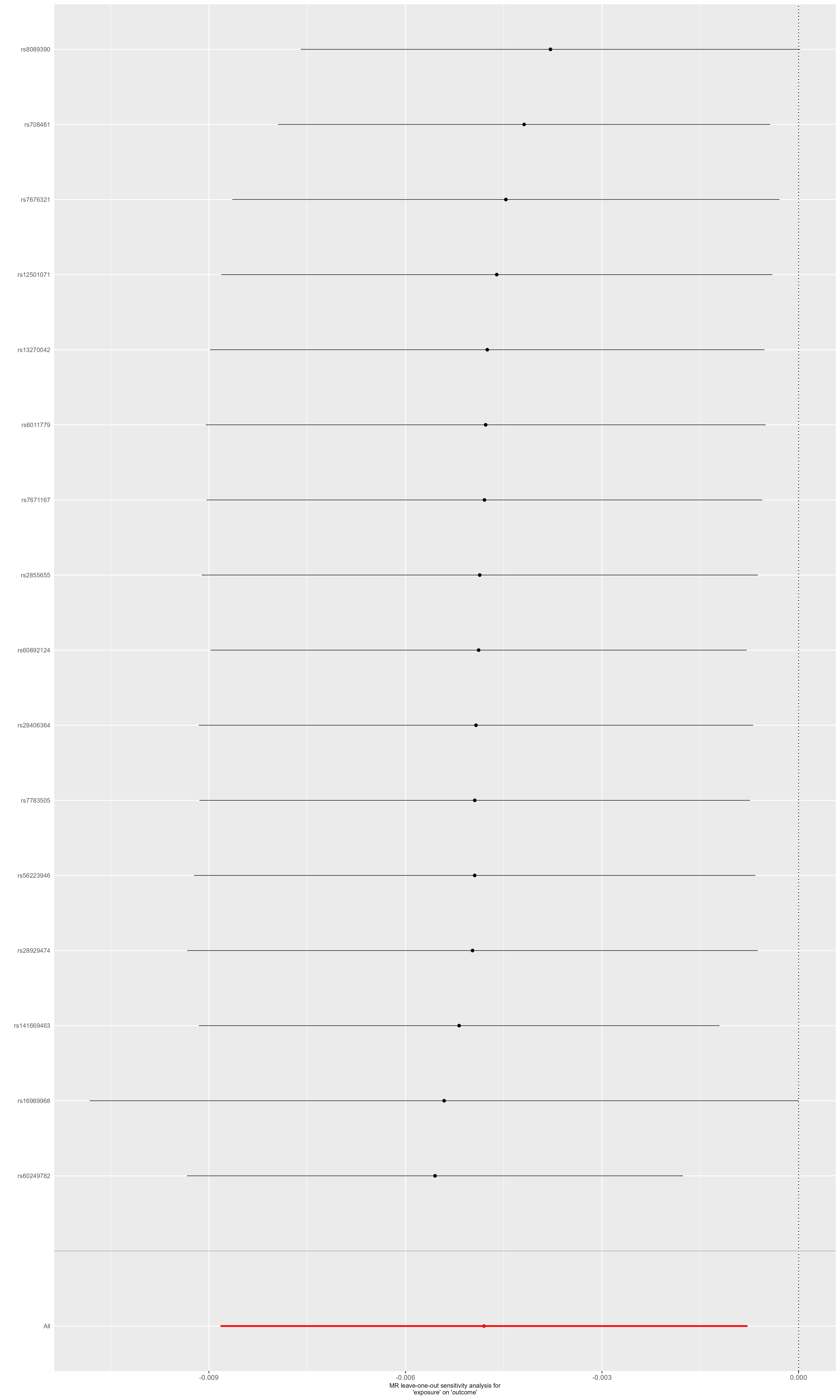

Supplement: Supplementary file 12 — Supplementary Material 12. [file 12890_2024_3150_MOESM12_ESM.zip › Supplementary Figure/leave-one-out analysis/Cortex Thickness/LOOA_COPD_inferiorparietal_thickavg_noGC.png]

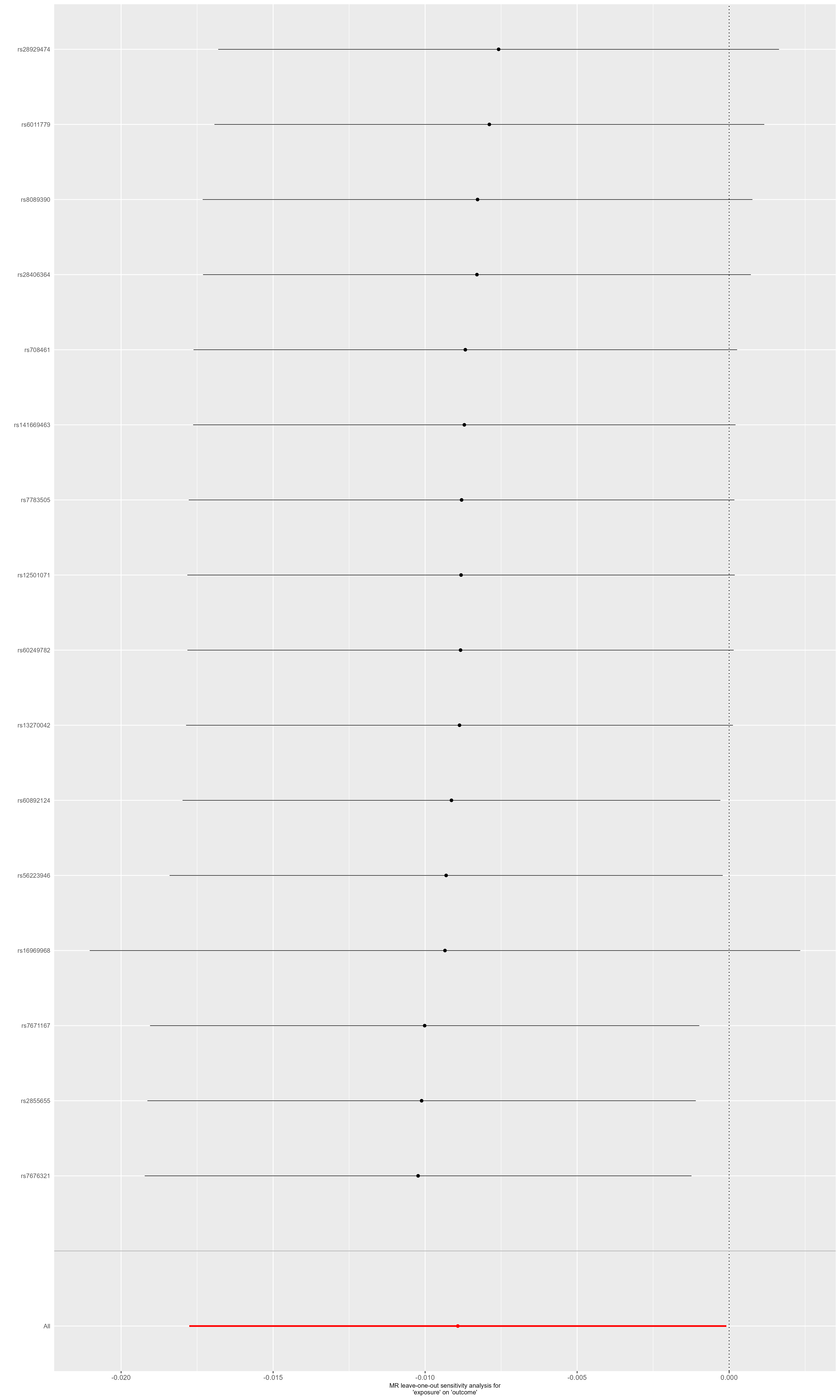

Supplement: Supplementary file 12 — Supplementary Material 12. [file 12890_2024_3150_MOESM12_ESM.zip › Supplementary Figure/leave-one-out analysis/Cortex Thickness/LOOA_COPD_rostralanteriorcingulate_thickavg_noGC.png]

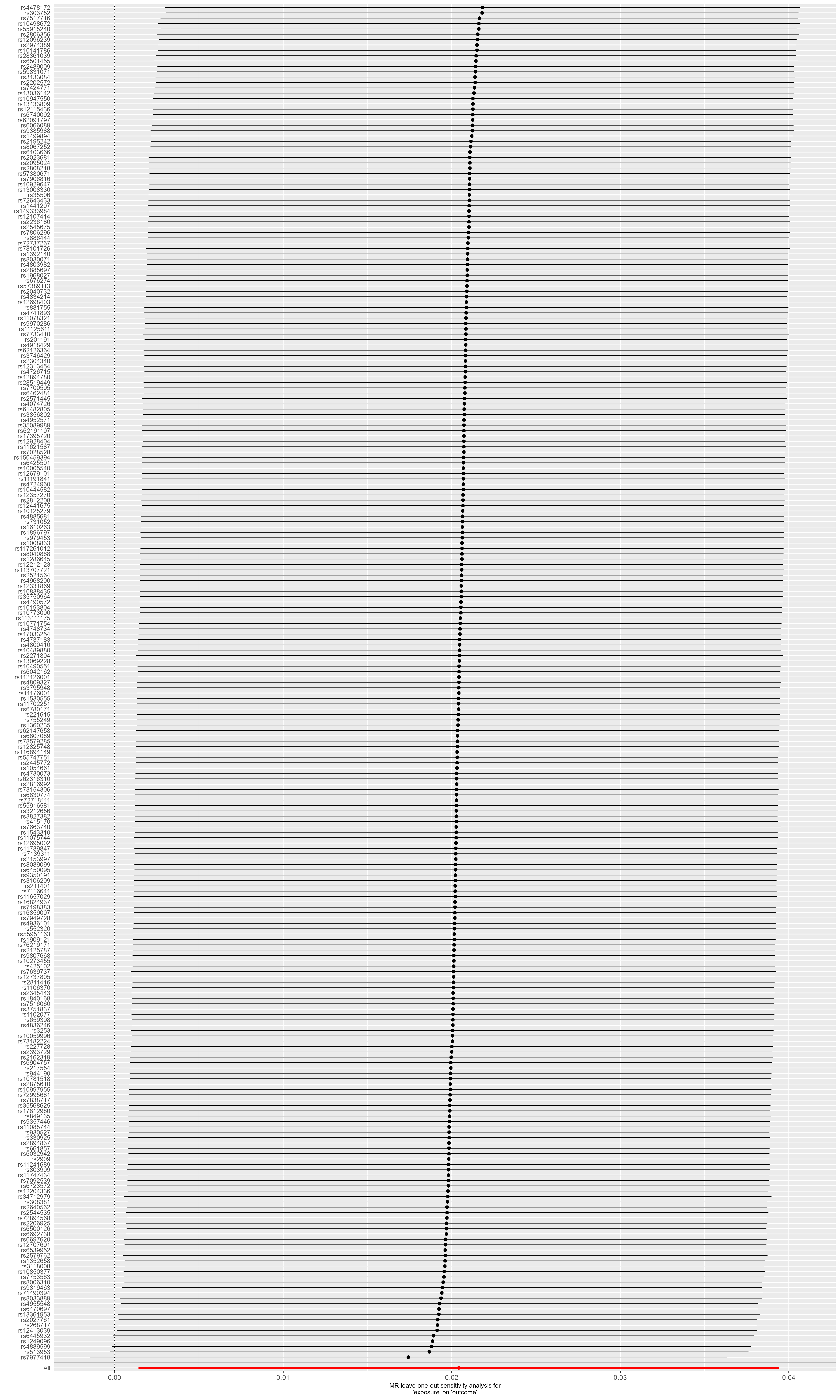

Supplement: Supplementary file 12 — Supplementary Material 12. [file 12890_2024_3150_MOESM12_ESM.zip › Supplementary Figure/leave-one-out analysis/Cortex Thickness/LOOA_FEV1_entorhinal_thickavg.png]

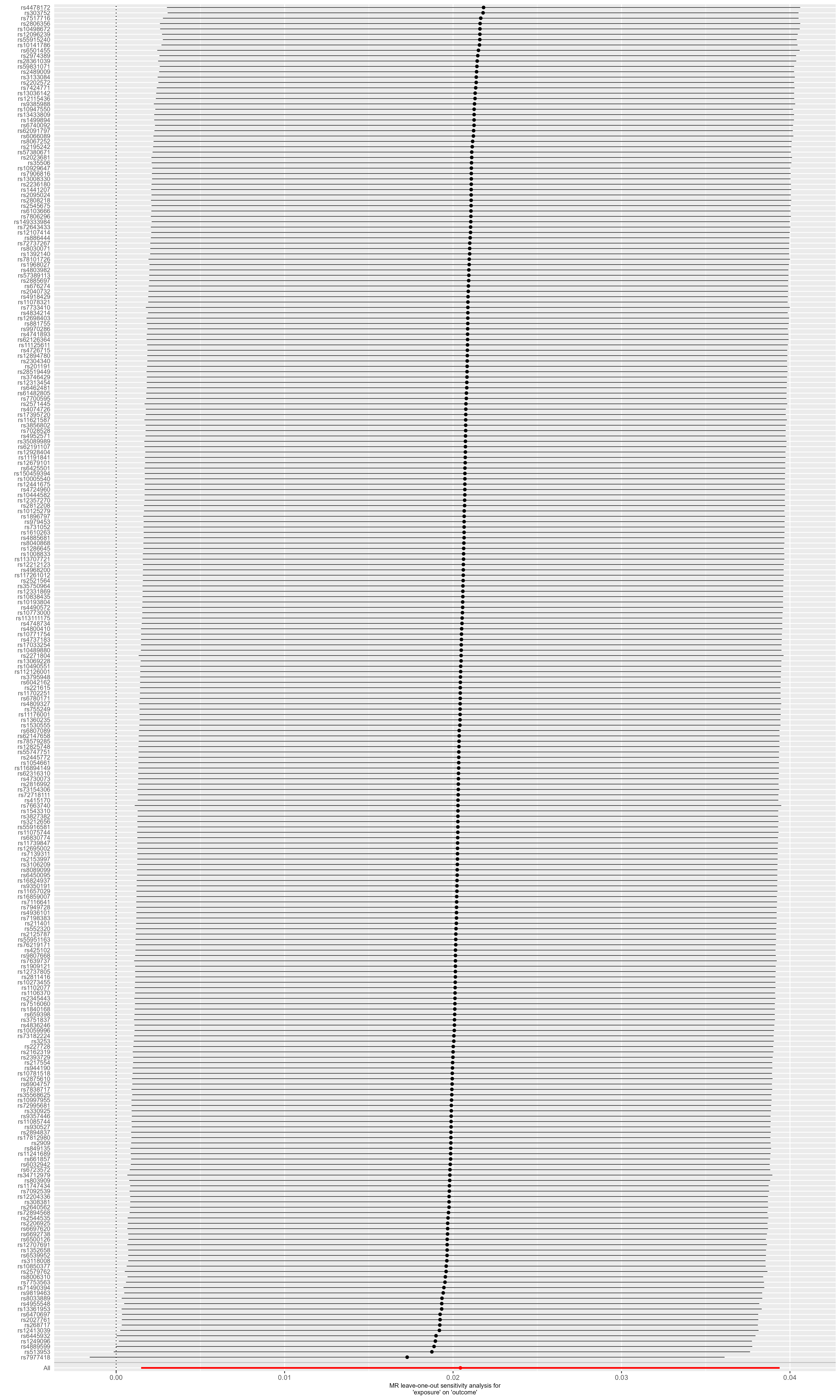

Supplement: Supplementary file 12 — Supplementary Material 12. [file 12890_2024_3150_MOESM12_ESM.zip › Supplementary Figure/leave-one-out analysis/Cortex Thickness/LOOA_FEV1_entorhinal_thickavg_noGC.png]

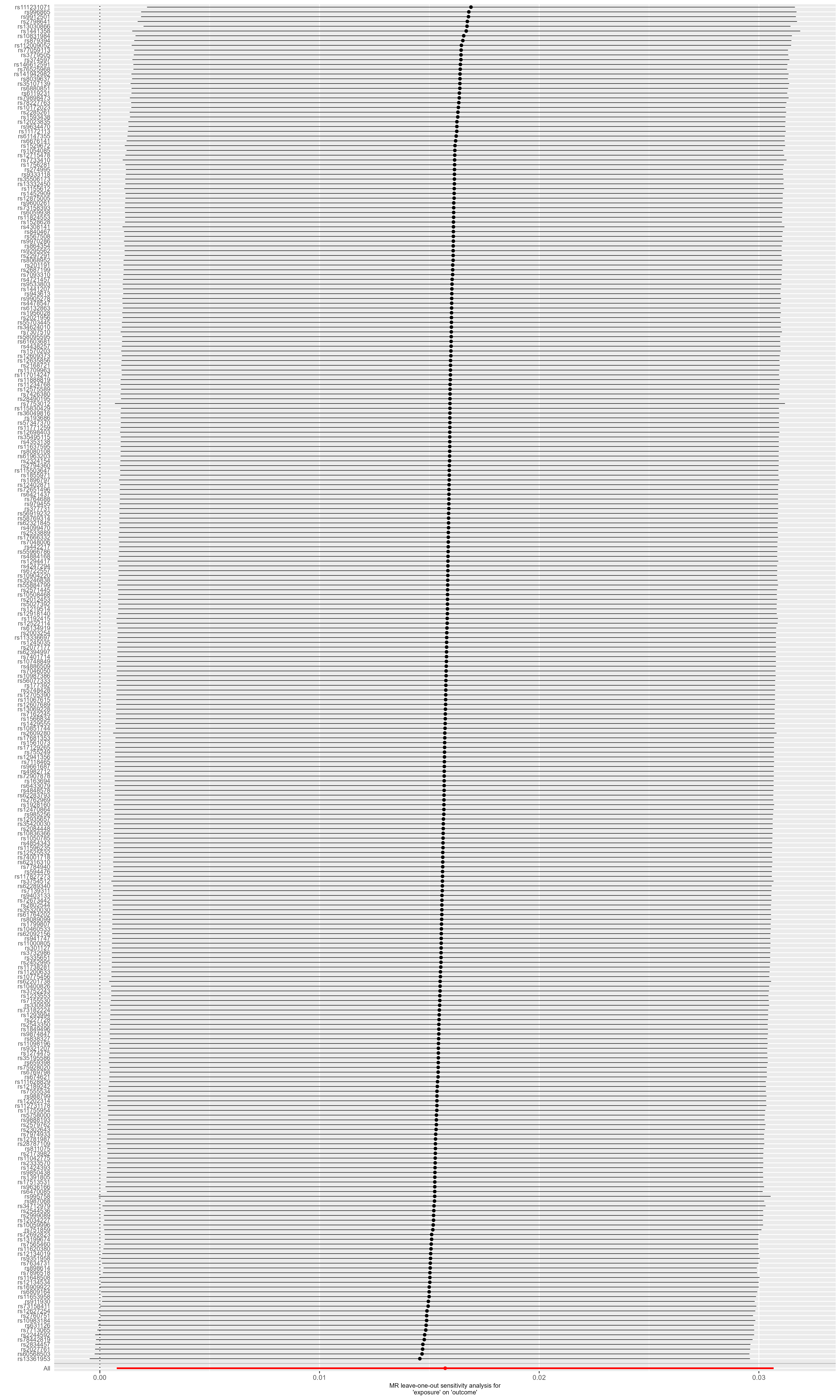

Supplement: Supplementary file 12 — Supplementary Material 12. [file 12890_2024_3150_MOESM12_ESM.zip › Supplementary Figure/leave-one-out analysis/Cortex Thickness/LOOA_FEV1_FVC_entorhinal_thickavg.png]

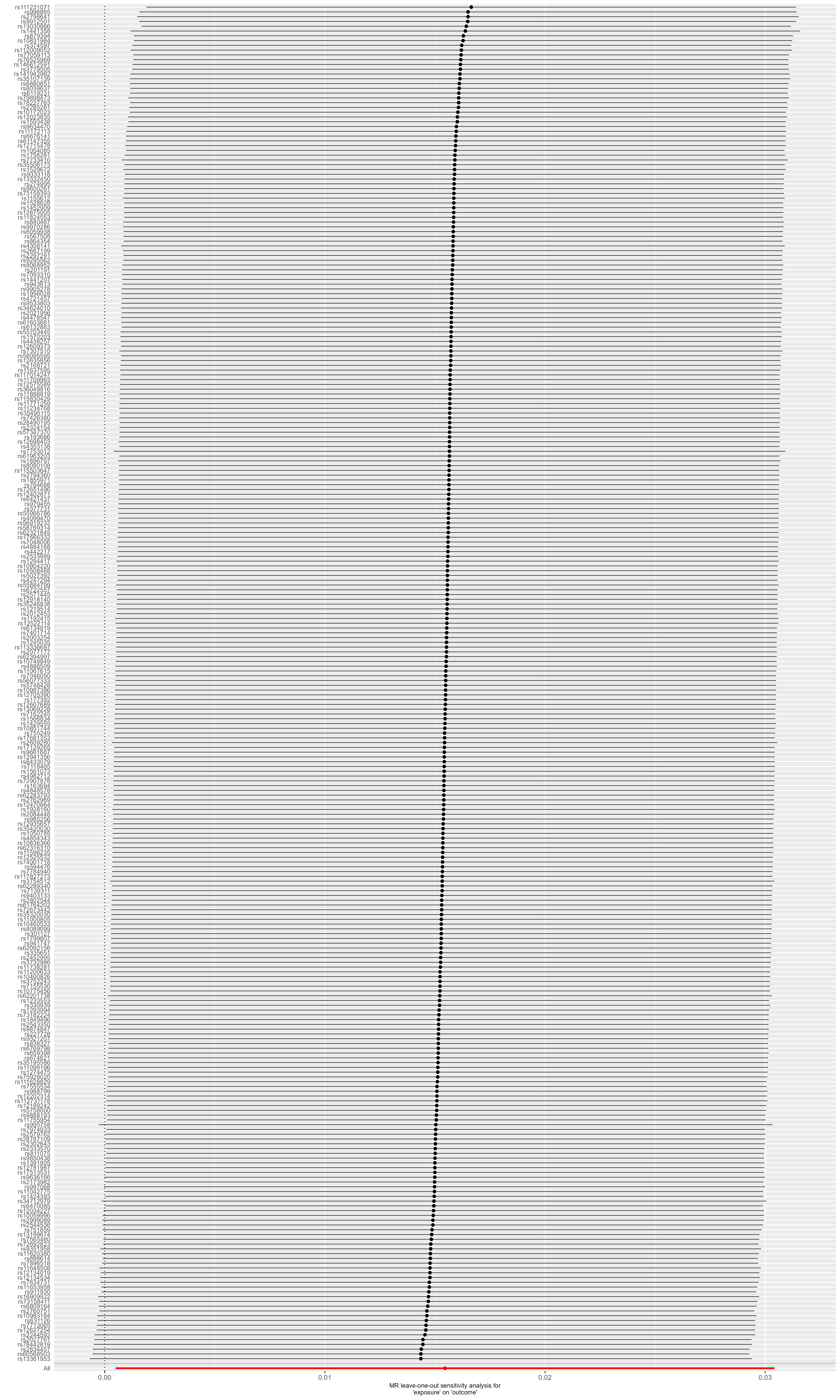

Supplement: Supplementary file 12 — Supplementary Material 12. [file 12890_2024_3150_MOESM12_ESM.zip › Supplementary Figure/leave-one-out analysis/Cortex Thickness/LOOA_FEV1_FVC_entorhinal_thickavg_noGC.png]

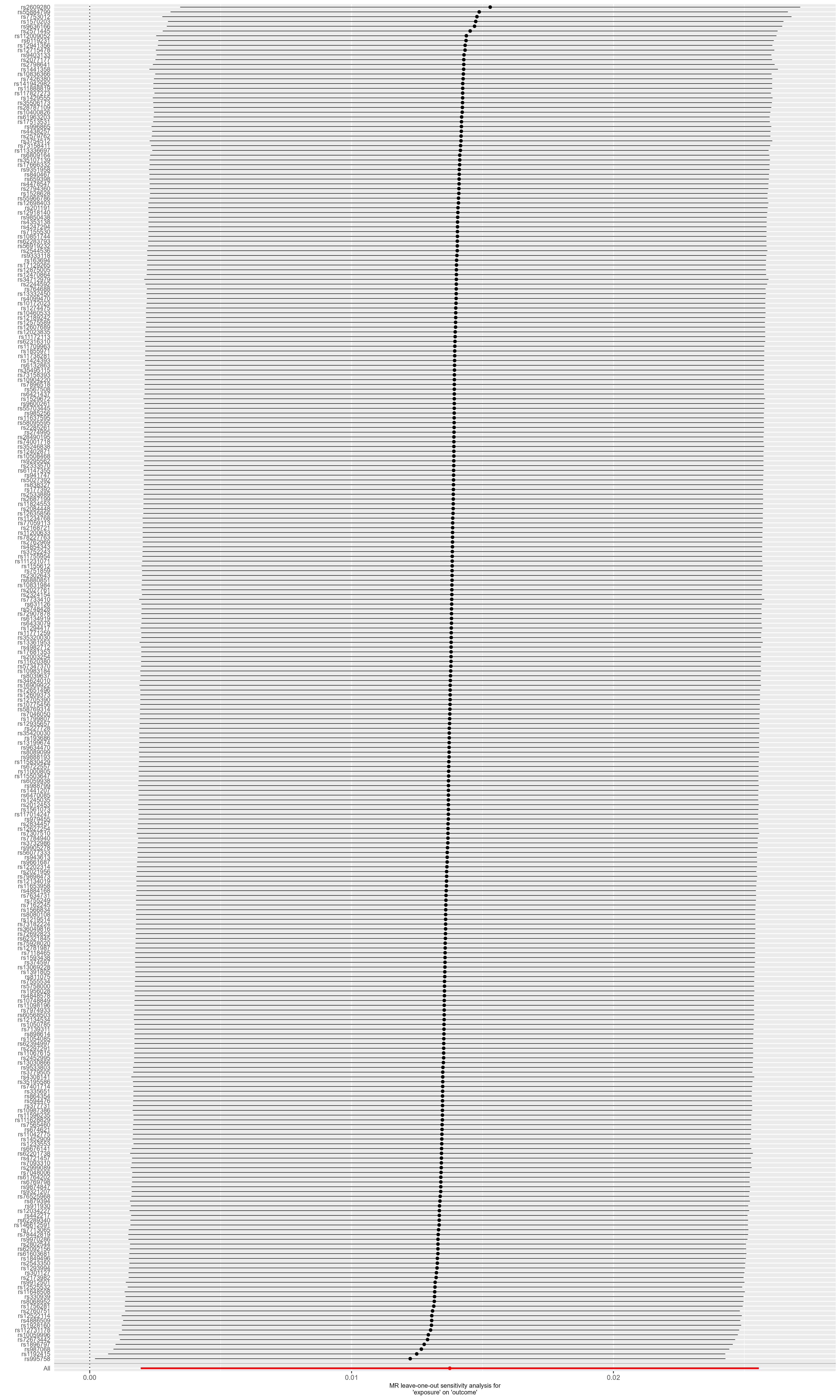

Supplement: Supplementary file 12 — Supplementary Material 12. [file 12890_2024_3150_MOESM12_ESM.zip › Supplementary Figure/leave-one-out analysis/Cortex Thickness/LOOA_FEV1_FVC_temporalpole_thickavg.png]

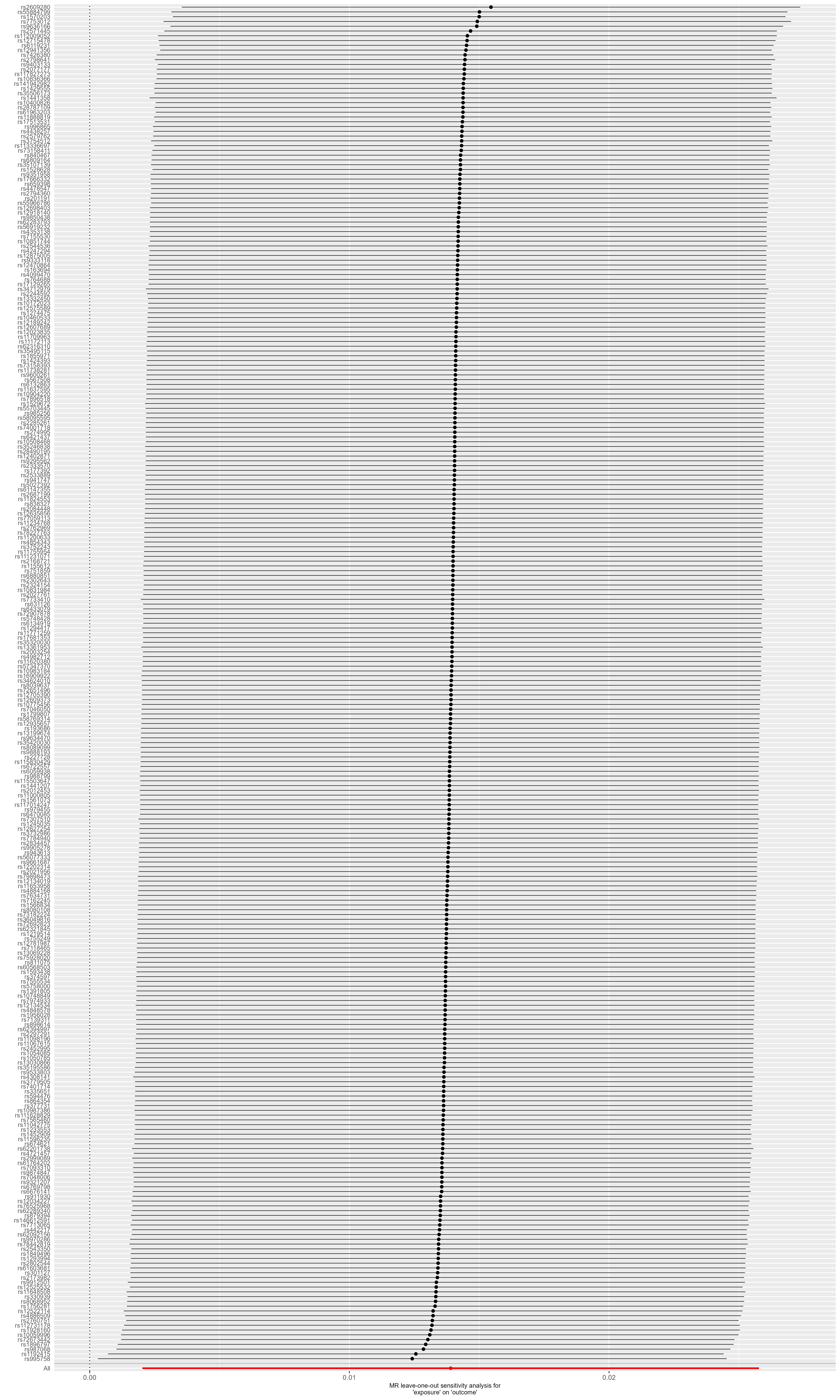

Supplement: Supplementary file 12 — Supplementary Material 12. [file 12890_2024_3150_MOESM12_ESM.zip › Supplementary Figure/leave-one-out analysis/Cortex Thickness/LOOA_FEV1_FVC_temporalpole_thickavg_noGC.png]

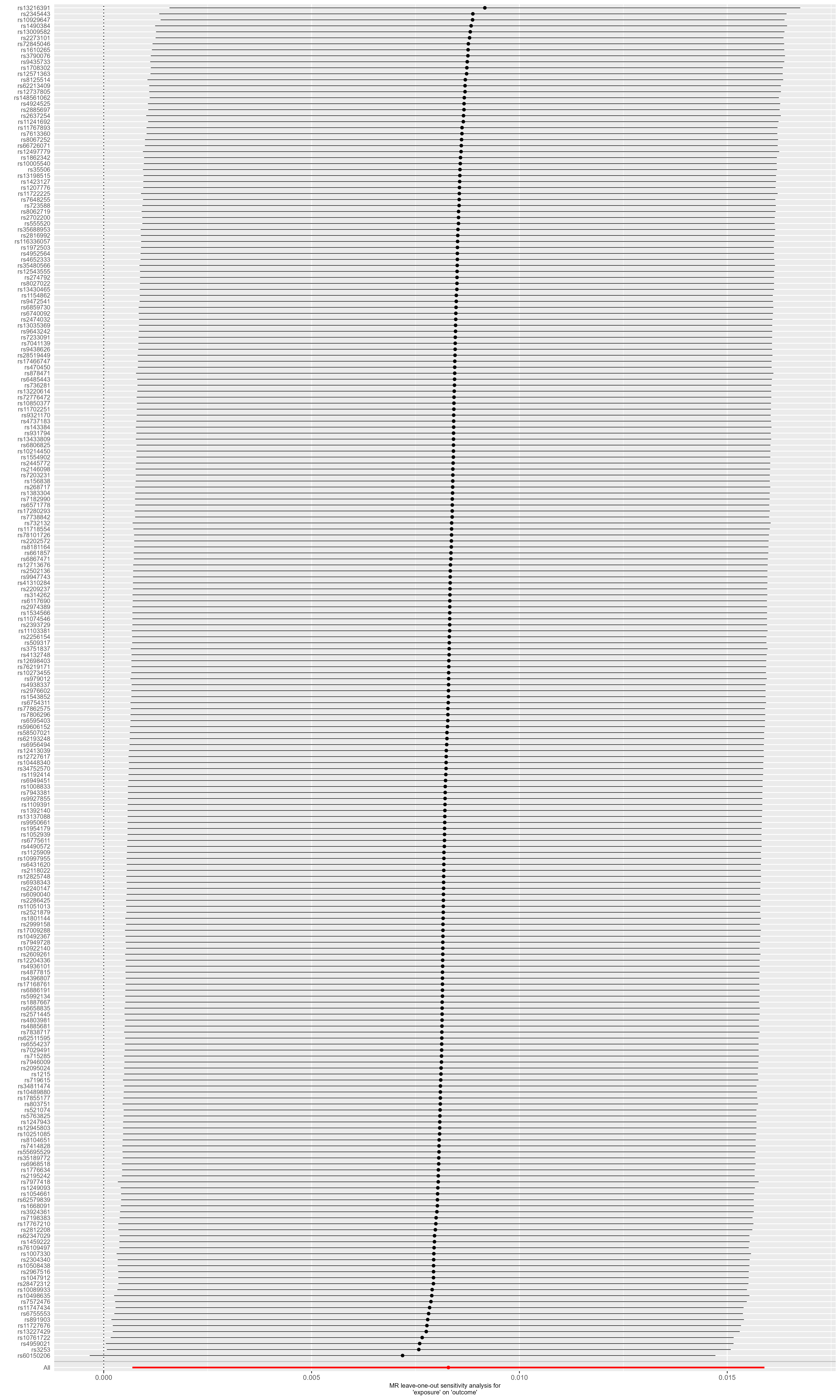

Supplement: Supplementary file 12 — Supplementary Material 12. [file 12890_2024_3150_MOESM12_ESM.zip › Supplementary Figure/leave-one-out analysis/Cortex Thickness/LOOA_FVC_bankssts_thickavg.png]

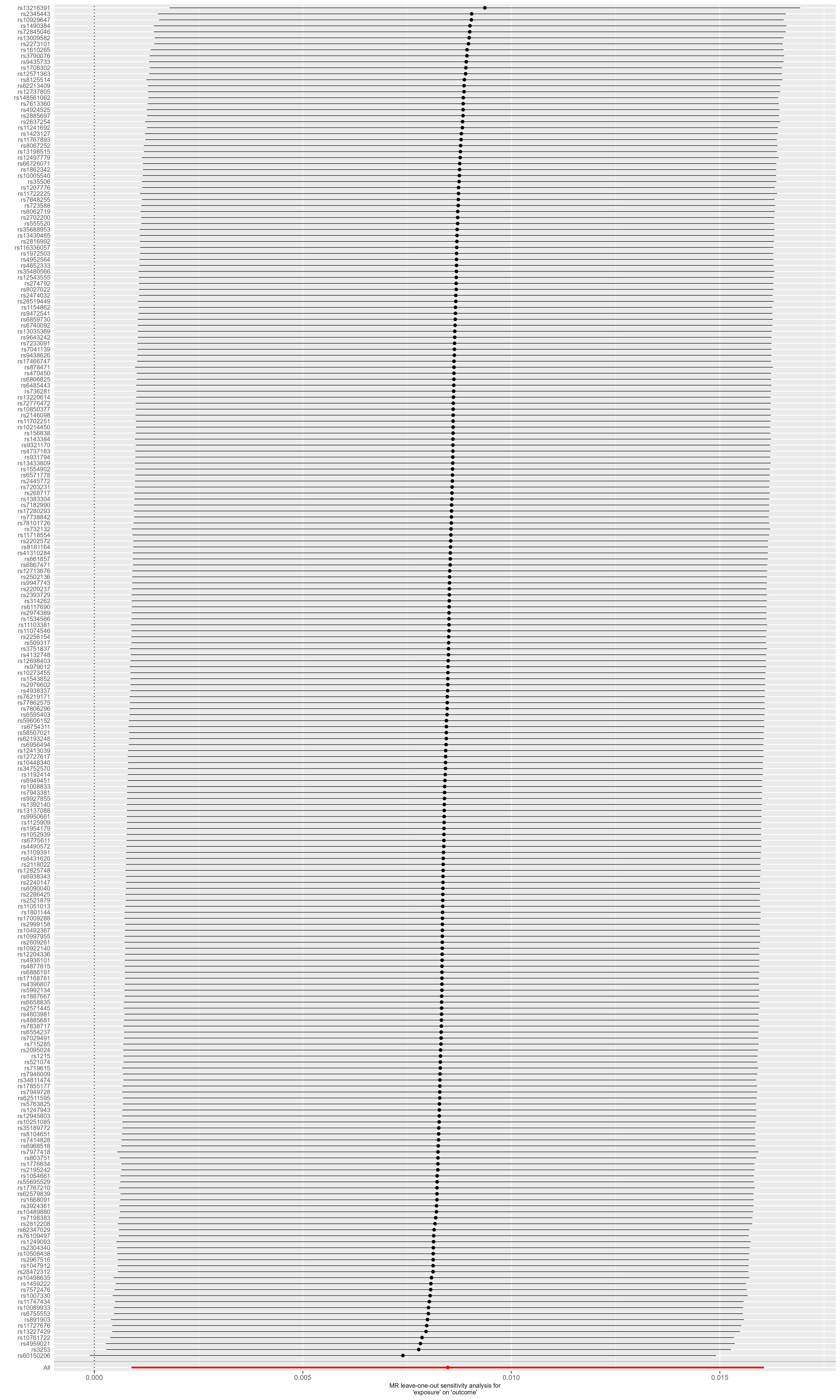

Supplement: Supplementary file 12 — Supplementary Material 12. [file 12890_2024_3150_MOESM12_ESM.zip › Supplementary Figure/leave-one-out analysis/Cortex Thickness/LOOA_FVC_bankssts_thickavg_noGC.png]

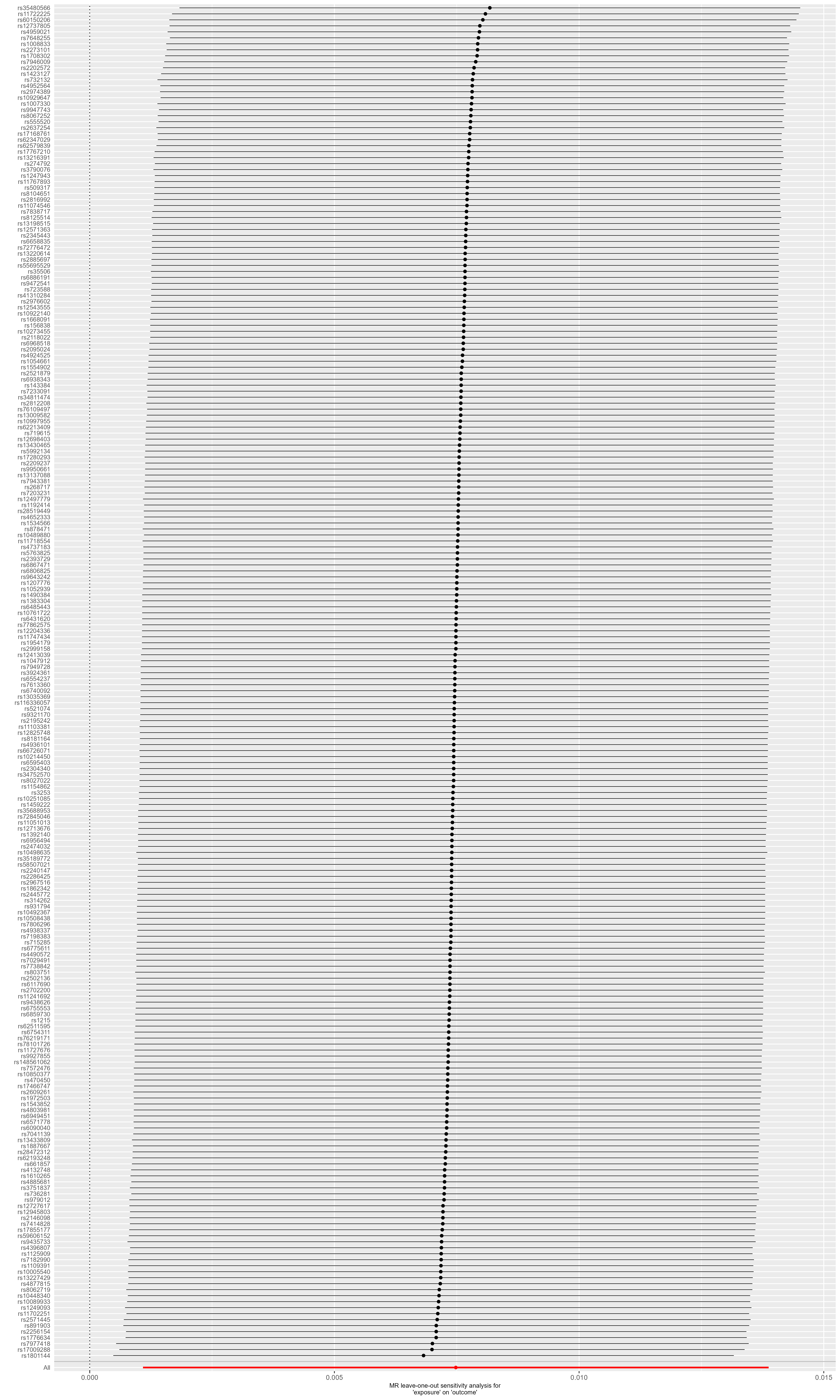

Supplement: Supplementary file 12 — Supplementary Material 12. [file 12890_2024_3150_MOESM12_ESM.zip › Supplementary Figure/leave-one-out analysis/Cortex Thickness/LOOA_FVC_fusiform_thickavg.png]

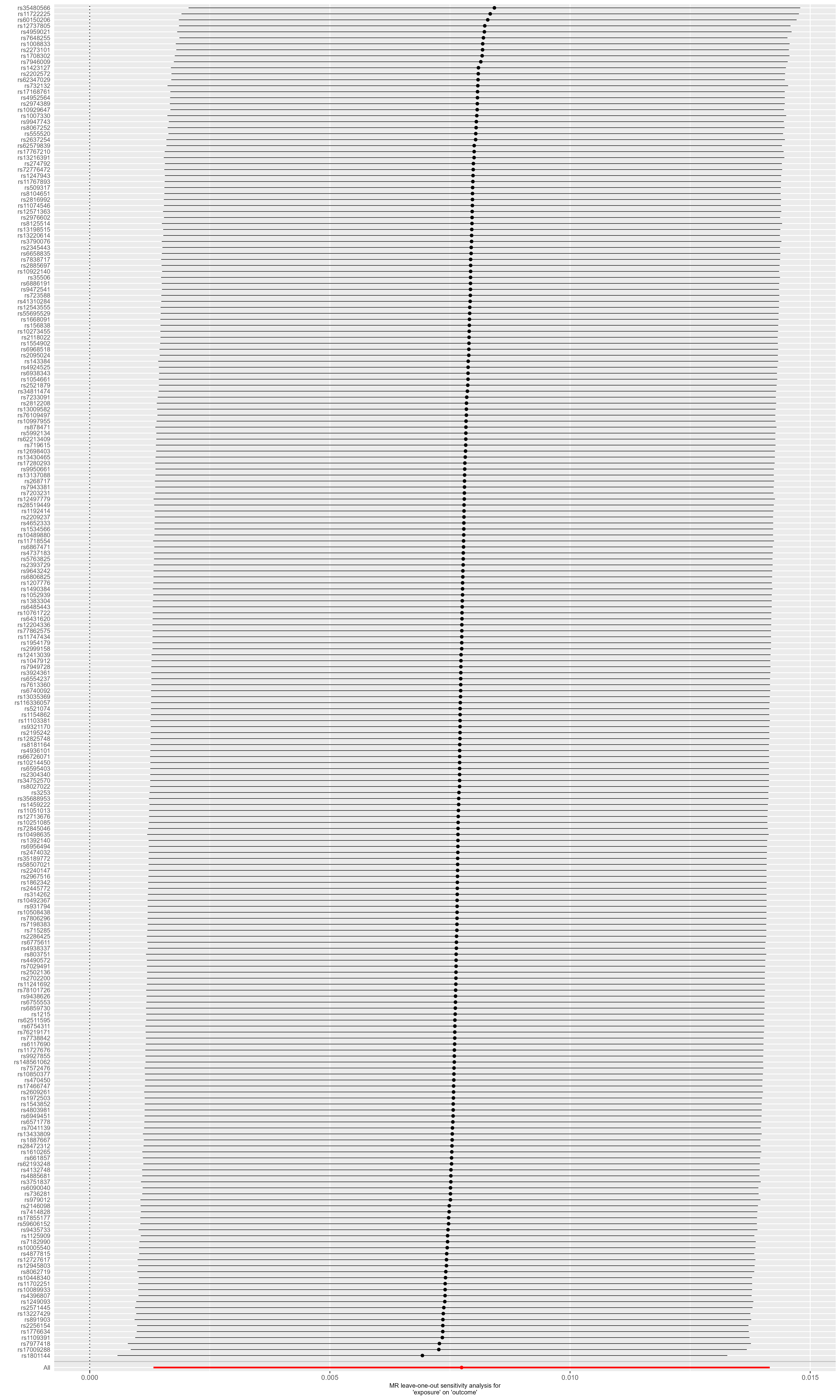

Supplement: Supplementary file 12 — Supplementary Material 12. [file 12890_2024_3150_MOESM12_ESM.zip › Supplementary Figure/leave-one-out analysis/Cortex Thickness/LOOA_FVC_fusiform_thickavg_noGC.png]

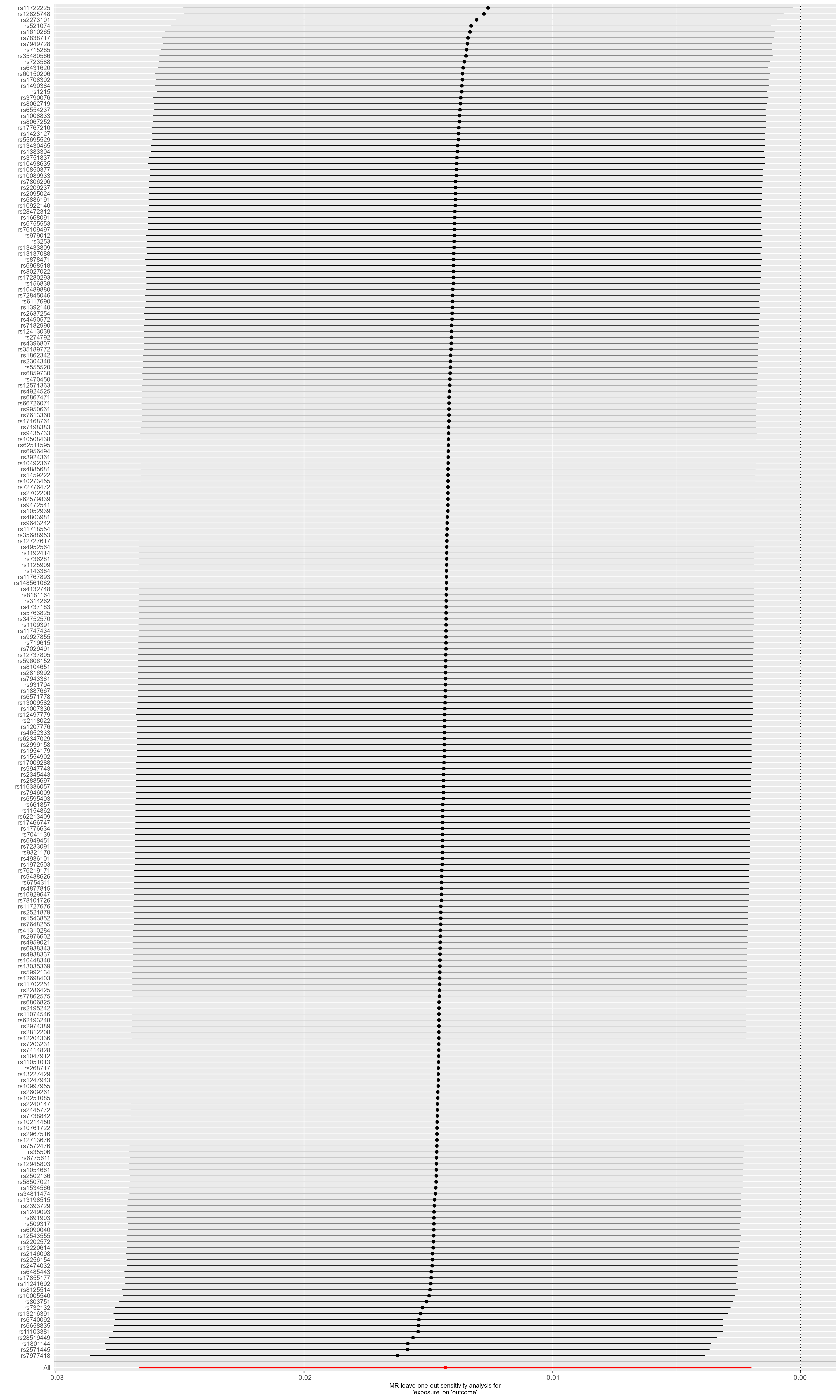

Supplement: Supplementary file 12 — Supplementary Material 12. [file 12890_2024_3150_MOESM12_ESM.zip › Supplementary Figure/leave-one-out analysis/Cortex Thickness/LOOA_FVC_isthmuscingulate_thickavg.png]

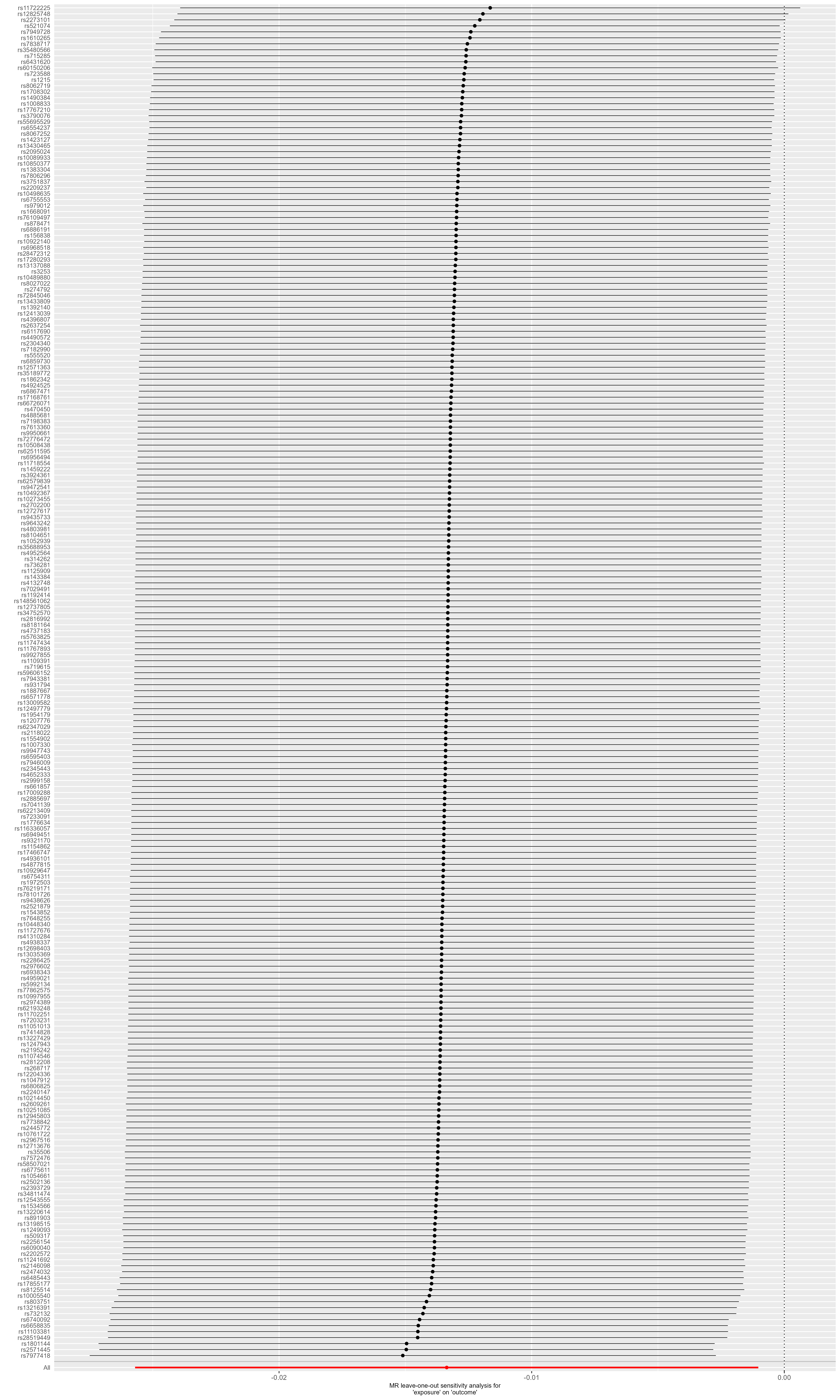

Supplement: Supplementary file 12 — Supplementary Material 12. [file 12890_2024_3150_MOESM12_ESM.zip › Supplementary Figure/leave-one-out analysis/Cortex Thickness/LOOA_FVC_isthmuscingulate_thickavg_noGC.png]

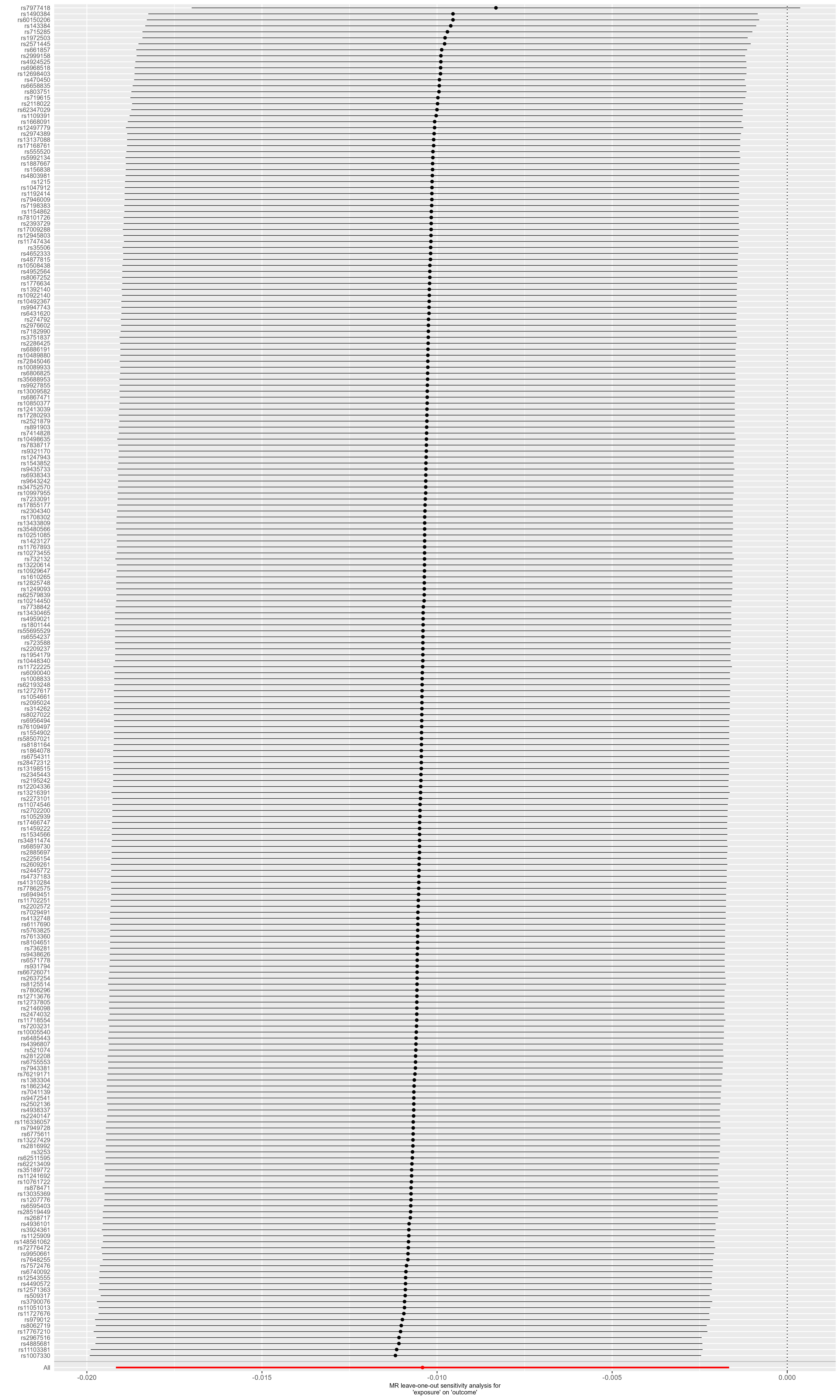

Supplement: Supplementary file 12 — Supplementary Material 12. [file 12890_2024_3150_MOESM12_ESM.zip › Supplementary Figure/leave-one-out analysis/Cortex Thickness/LOOA_FVC_medialorbitofrontal_thickavg.png]

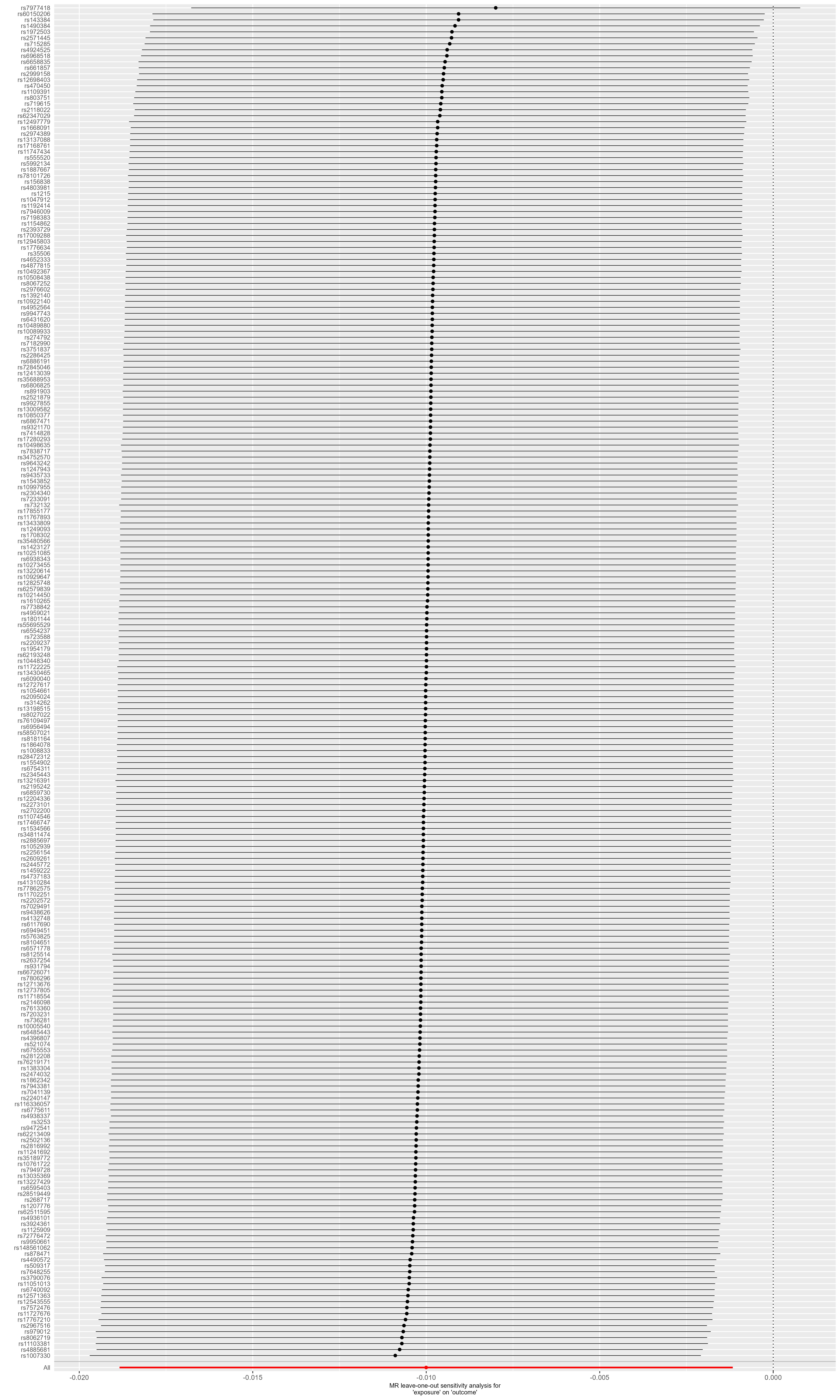

Supplement: Supplementary file 12 — Supplementary Material 12. [file 12890_2024_3150_MOESM12_ESM.zip › Supplementary Figure/leave-one-out analysis/Cortex Thickness/LOOA_FVC_medialorbitofrontal_thickavg_noGC.png]

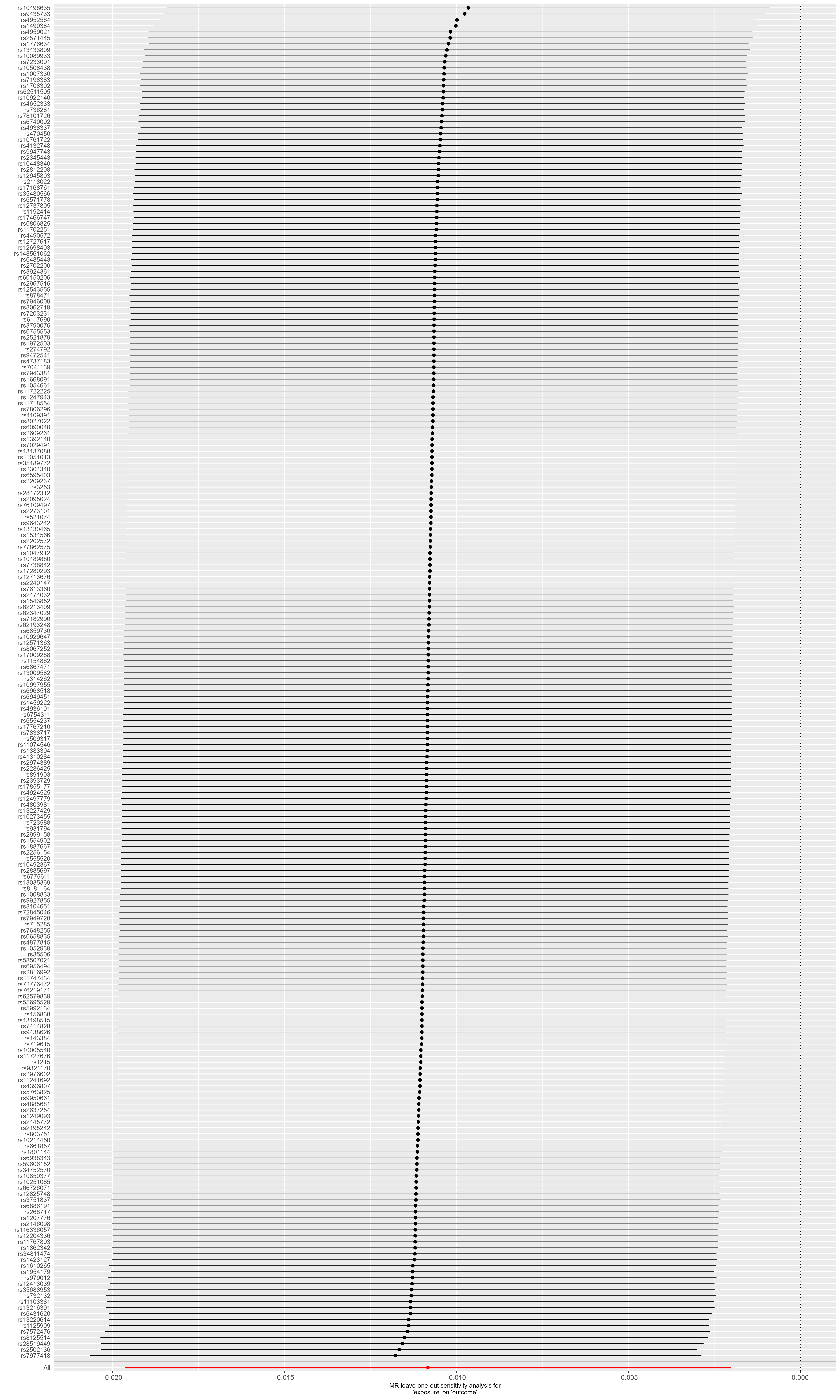

Supplement: Supplementary file 12 — Supplementary Material 12. [file 12890_2024_3150_MOESM12_ESM.zip › Supplementary Figure/leave-one-out analysis/Cortex Thickness/LOOA_FVC_parsorbitalis_thickavg.png]

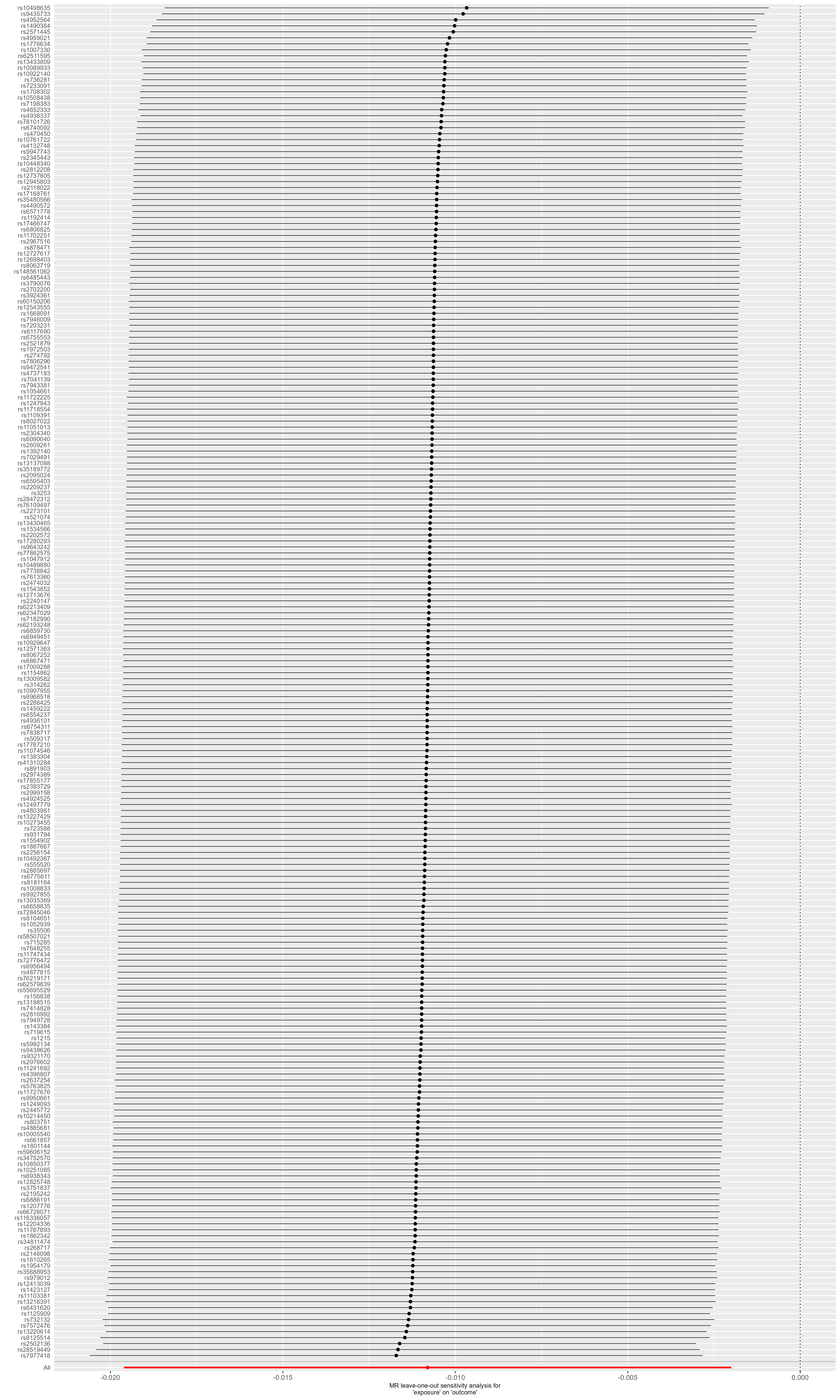

Supplement: Supplementary file 12 — Supplementary Material 12. [file 12890_2024_3150_MOESM12_ESM.zip › Supplementary Figure/leave-one-out analysis/Cortex Thickness/LOOA_FVC_parsorbitalis_thickavg_noGC.png]

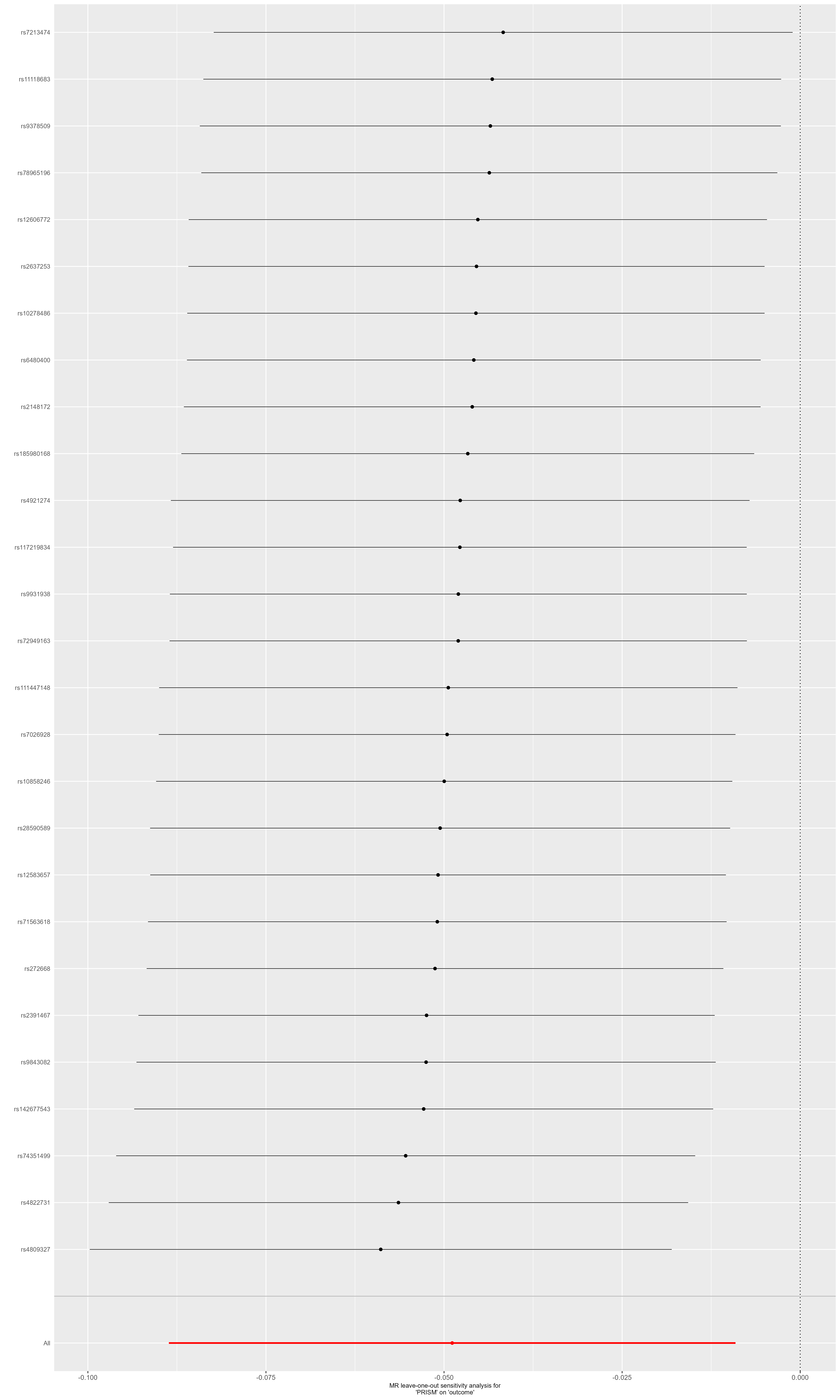

Supplement: Supplementary file 12 — Supplementary Material 12. [file 12890_2024_3150_MOESM12_ESM.zip › Supplementary Figure/leave-one-out analysis/Cortex Thickness/LOOA_PRISM_inferiorparietal_thickavg_GC.png]

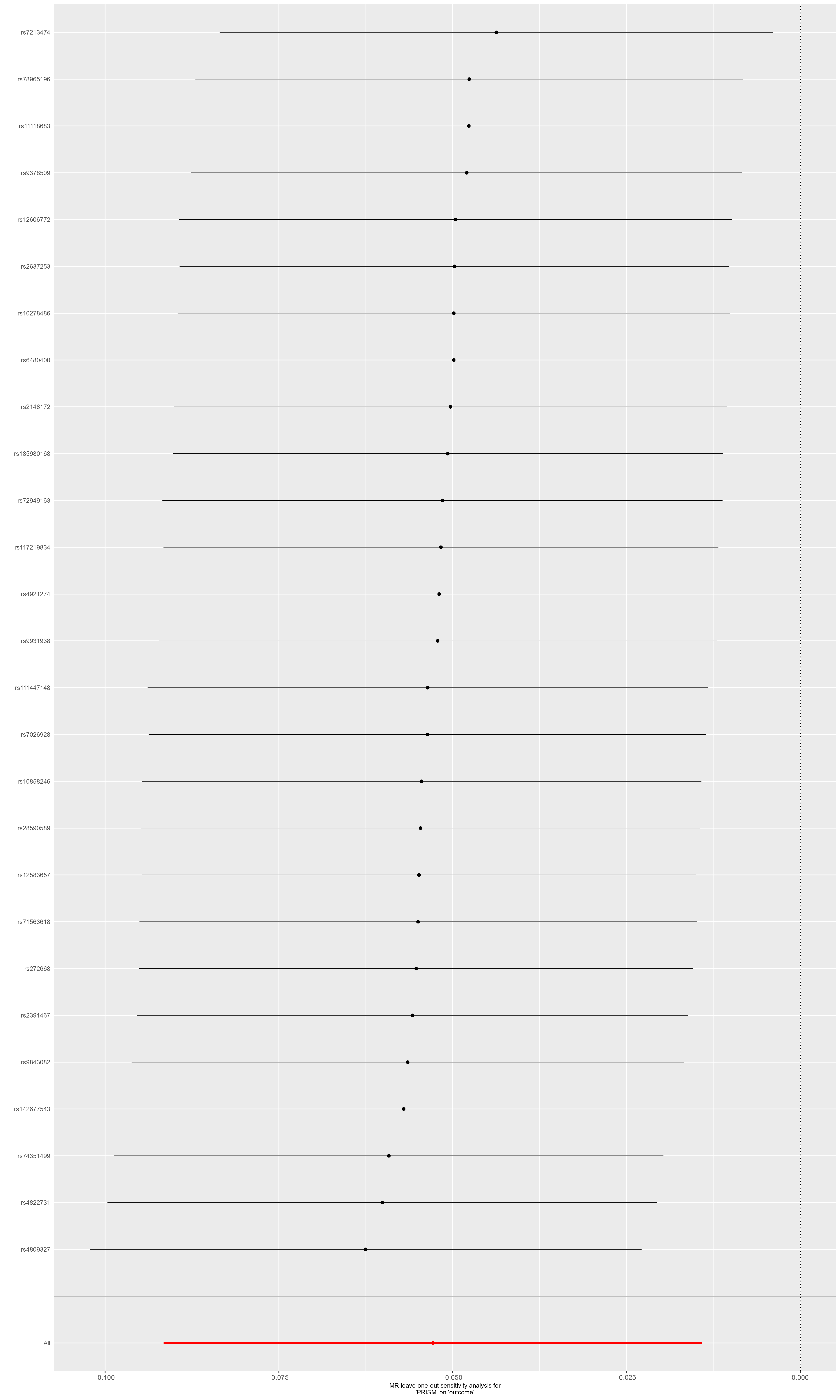

Supplement: Supplementary file 12 — Supplementary Material 12. [file 12890_2024_3150_MOESM12_ESM.zip › Supplementary Figure/leave-one-out analysis/Cortex Thickness/LOOA_PRISM_inferiorparietal_thickavg_noGC.png]

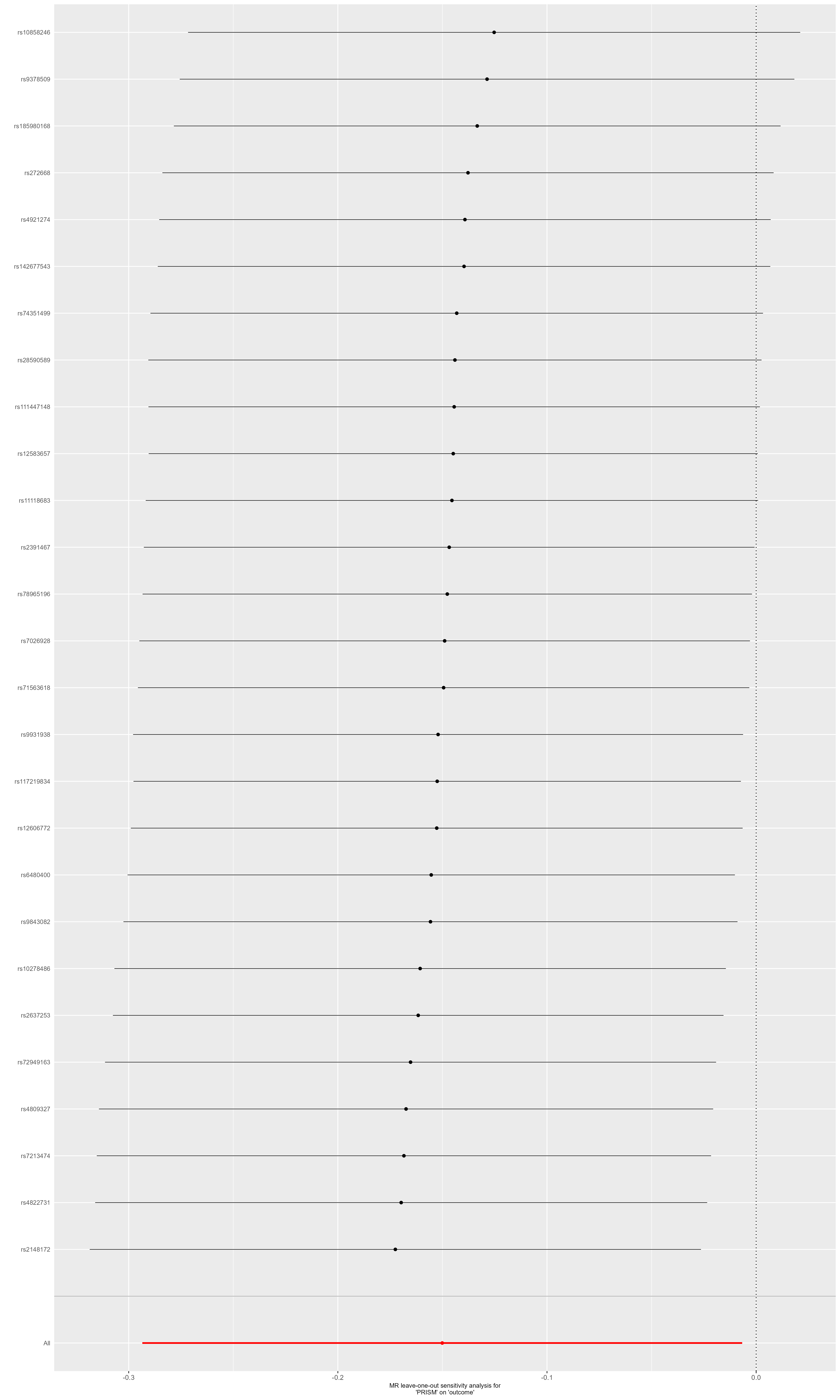

Supplement: Supplementary file 12 — Supplementary Material 12. [file 12890_2024_3150_MOESM12_ESM.zip › Supplementary Figure/leave-one-out analysis/Cortex Thickness/LOOA_PRISM_temporalpole_thickavg_GC.png]

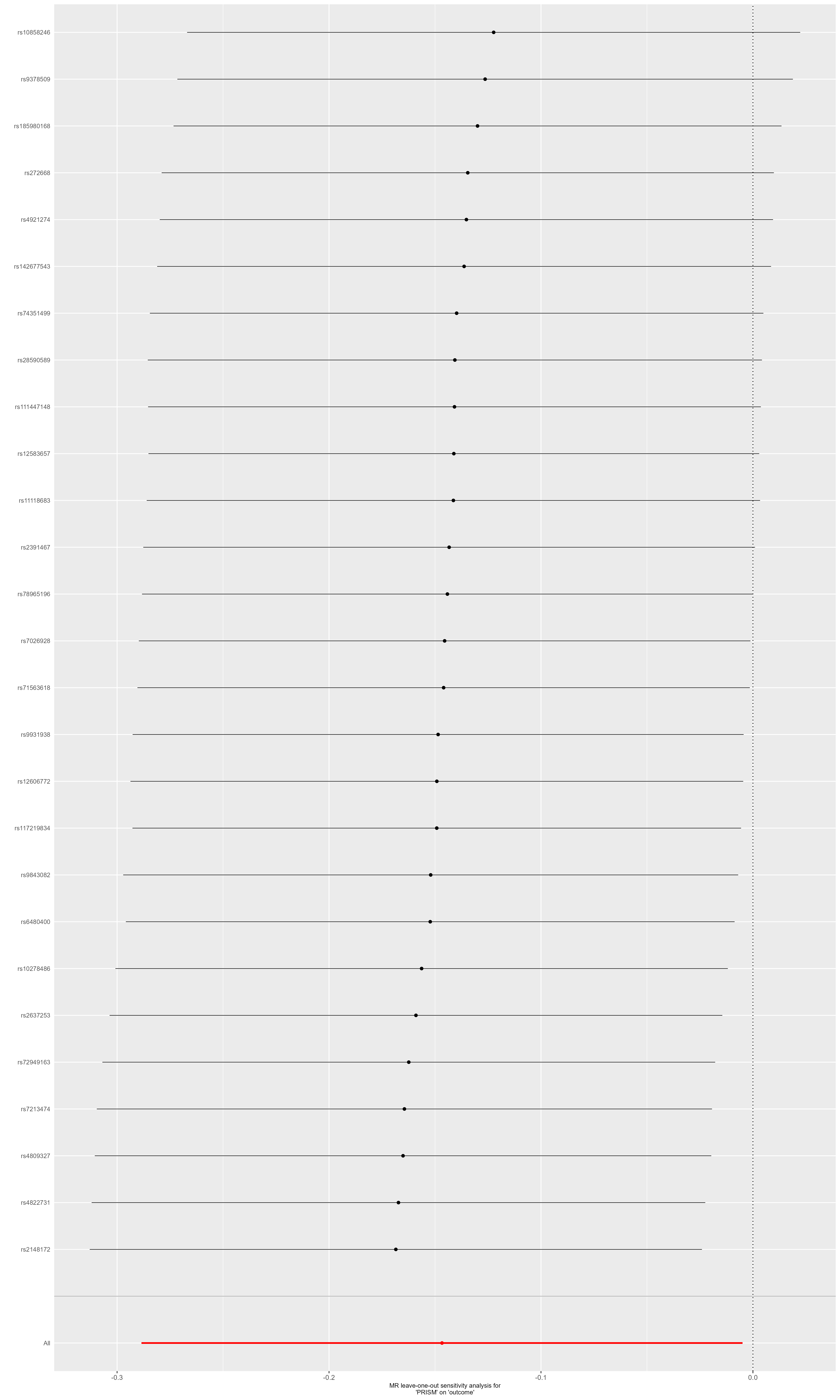

Supplement: Supplementary file 12 — Supplementary Material 12. [file 12890_2024_3150_MOESM12_ESM.zip › Supplementary Figure/leave-one-out analysis/Cortex Thickness/LOOA_PRISM_temporalpole_thickavg_noGC.png]

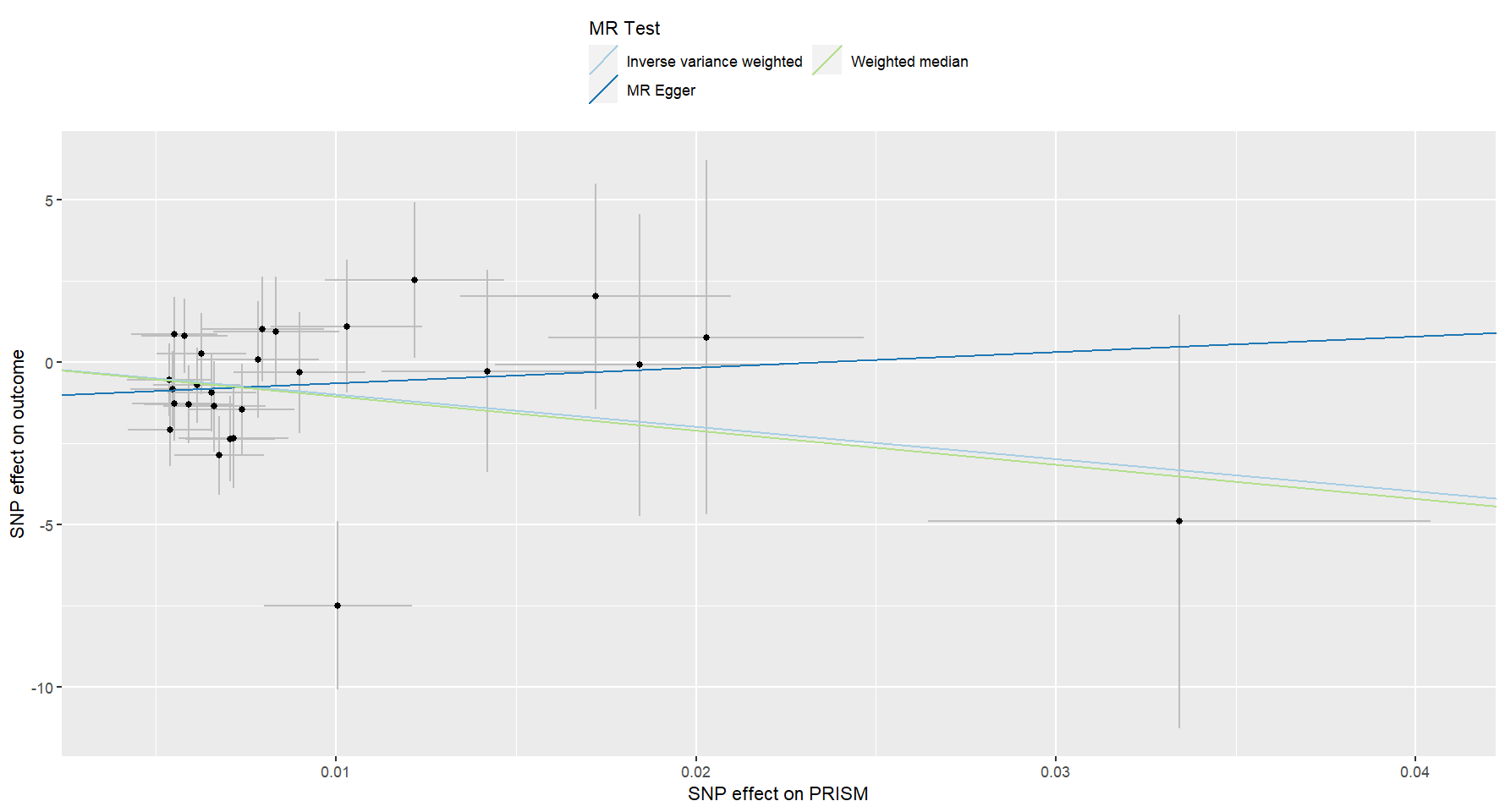

Supplement: Supplementary file 12 — Supplementary Material 12. [file 12890_2024_3150_MOESM12_ESM.zip › Supplementary Figure/scatter plot/Cortex Surface area/scatter_plot_PRISM_paracentral_surfavg_GC.png]

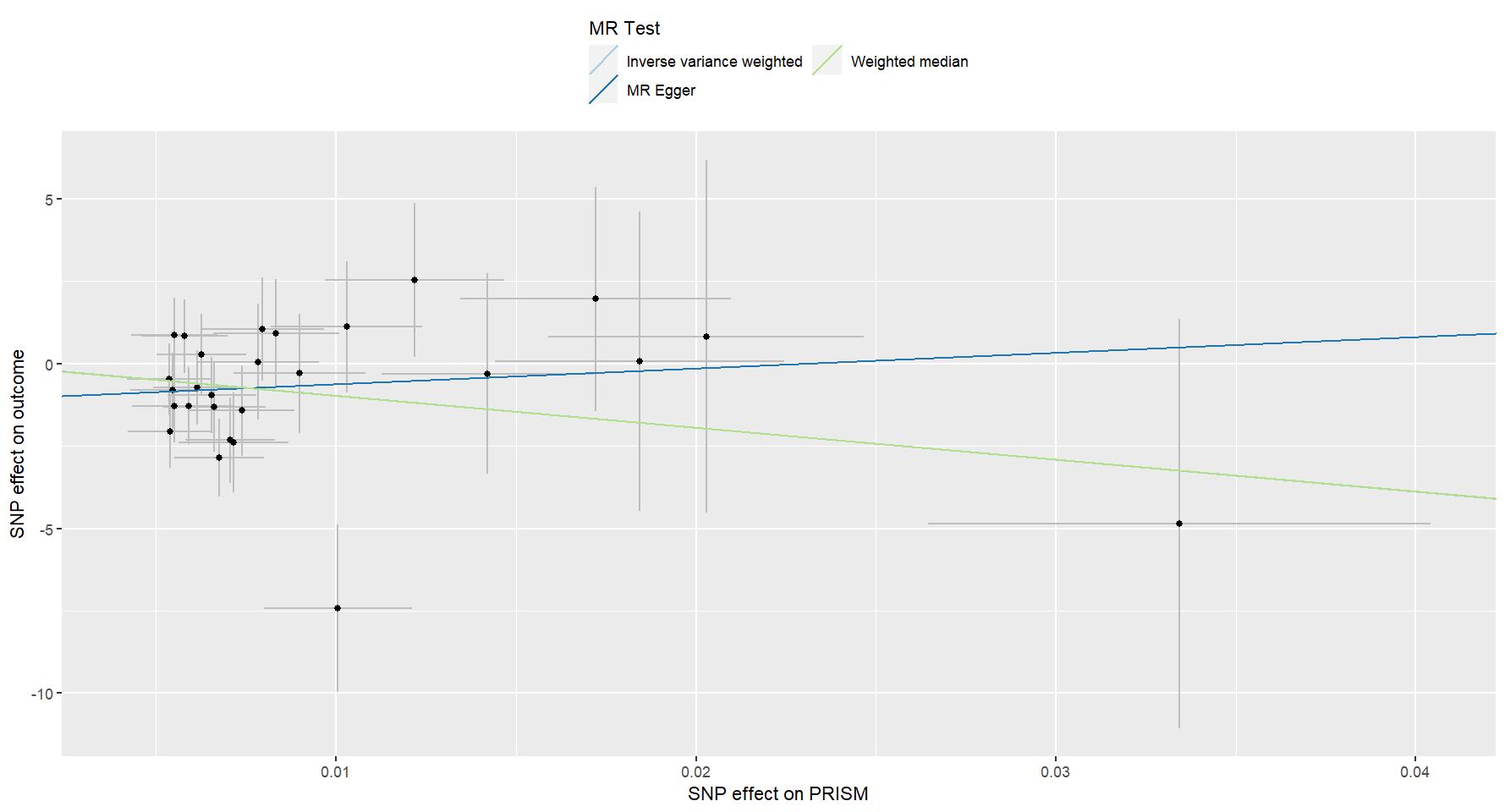

Supplement: Supplementary file 12 — Supplementary Material 12. [file 12890_2024_3150_MOESM12_ESM.zip › Supplementary Figure/scatter plot/Cortex Surface area/scatter_plot_PRISM_paracentral_surfavg_noGC.png]

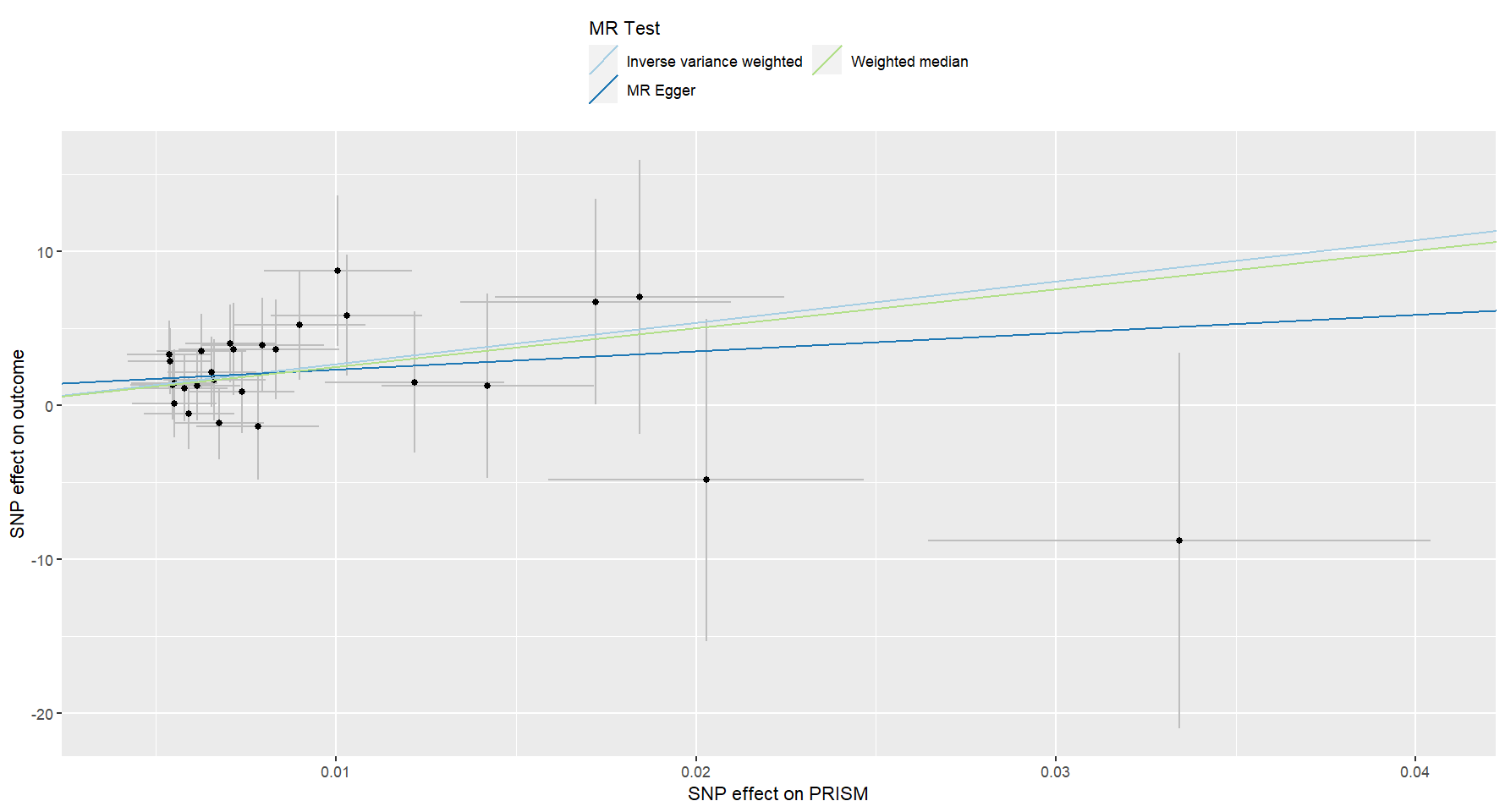

Supplement: Supplementary file 12 — Supplementary Material 12. [file 12890_2024_3150_MOESM12_ESM.zip › Supplementary Figure/scatter plot/Cortex Surface area/scatter_plot_PRISM_precuneus_surfavg_GC.png]

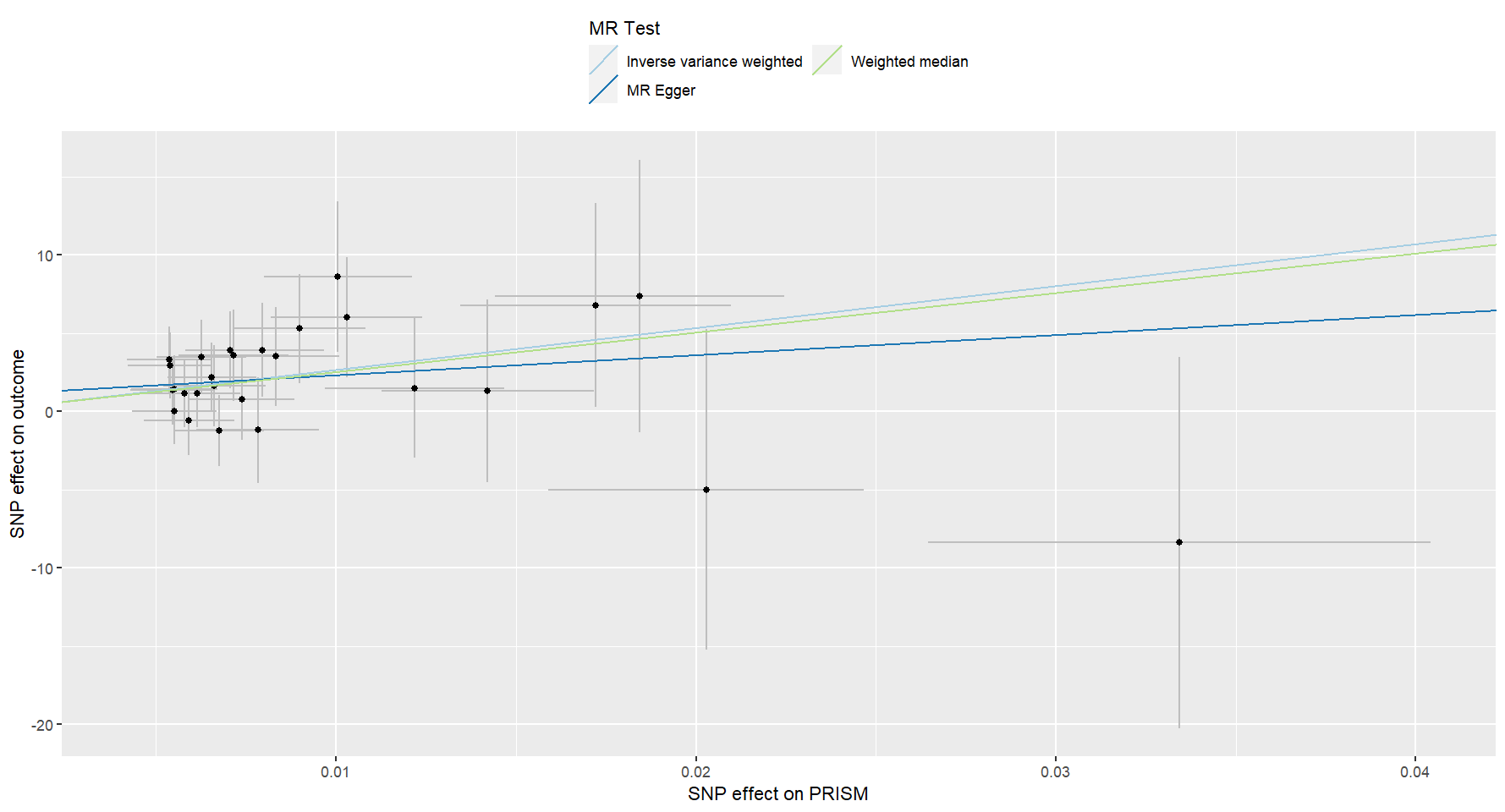

Supplement: Supplementary file 12 — Supplementary Material 12. [file 12890_2024_3150_MOESM12_ESM.zip › Supplementary Figure/scatter plot/Cortex Surface area/scatter_plot_PRISM_precuneus_surfavg_noGC.png]

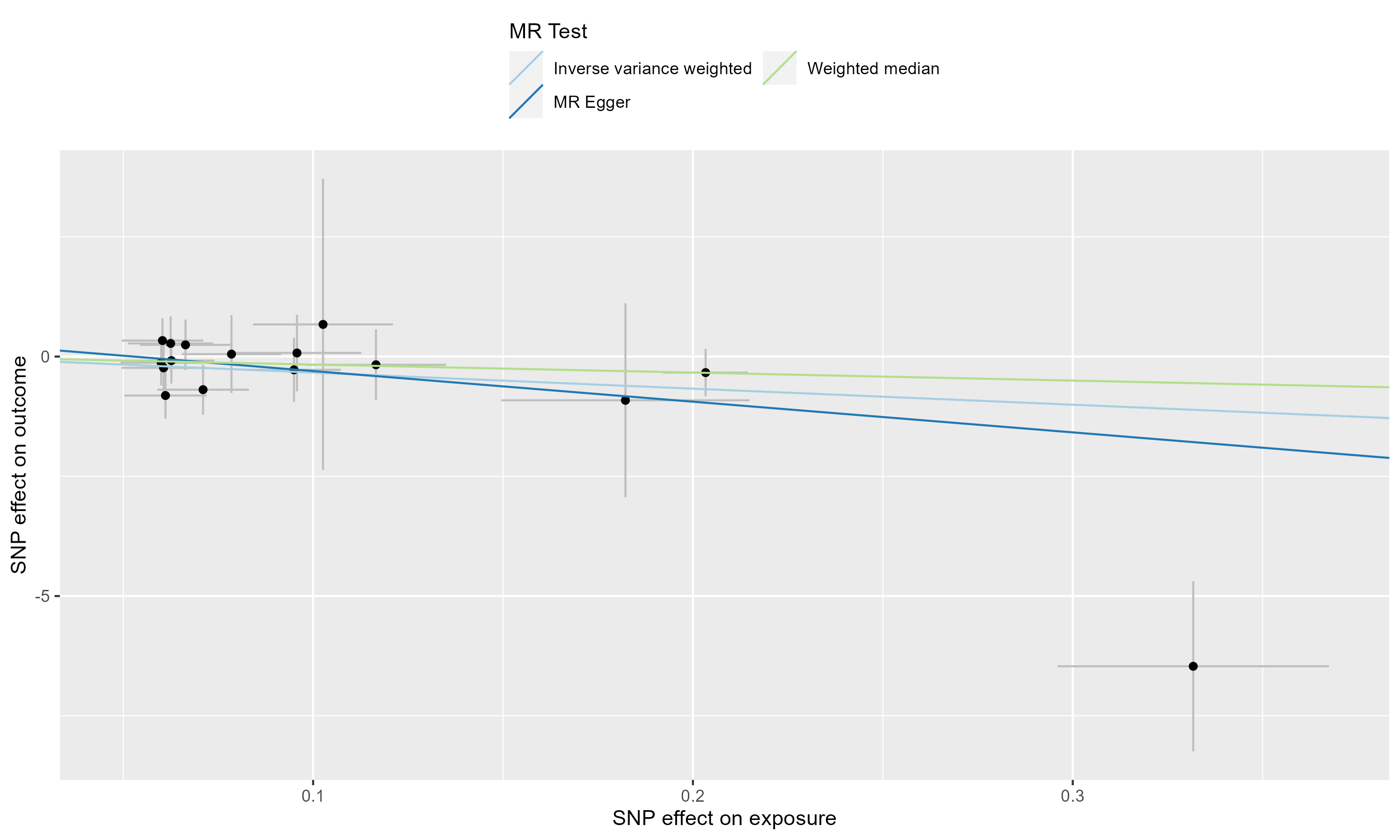

Supplement: Supplementary file 12 — Supplementary Material 12. [file 12890_2024_3150_MOESM12_ESM.zip › Supplementary Figure/scatter plot/Cortex Surface area/scatter_plotCOPD_parsorbitalis_surfavg.png]

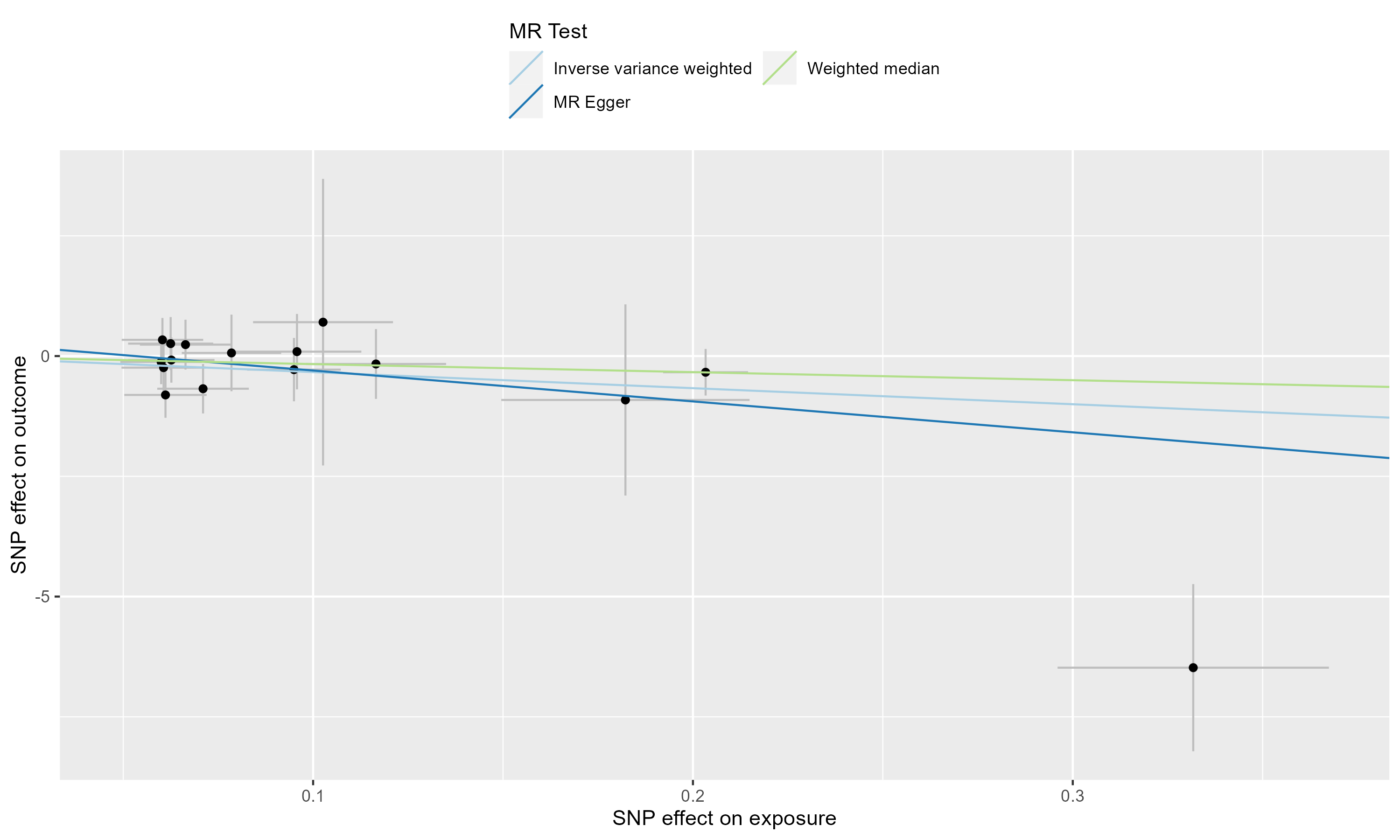

Supplement: Supplementary file 12 — Supplementary Material 12. [file 12890_2024_3150_MOESM12_ESM.zip › Supplementary Figure/scatter plot/Cortex Surface area/scatter_plotCOPD_parsorbitalis_surfavg_noGC.png]

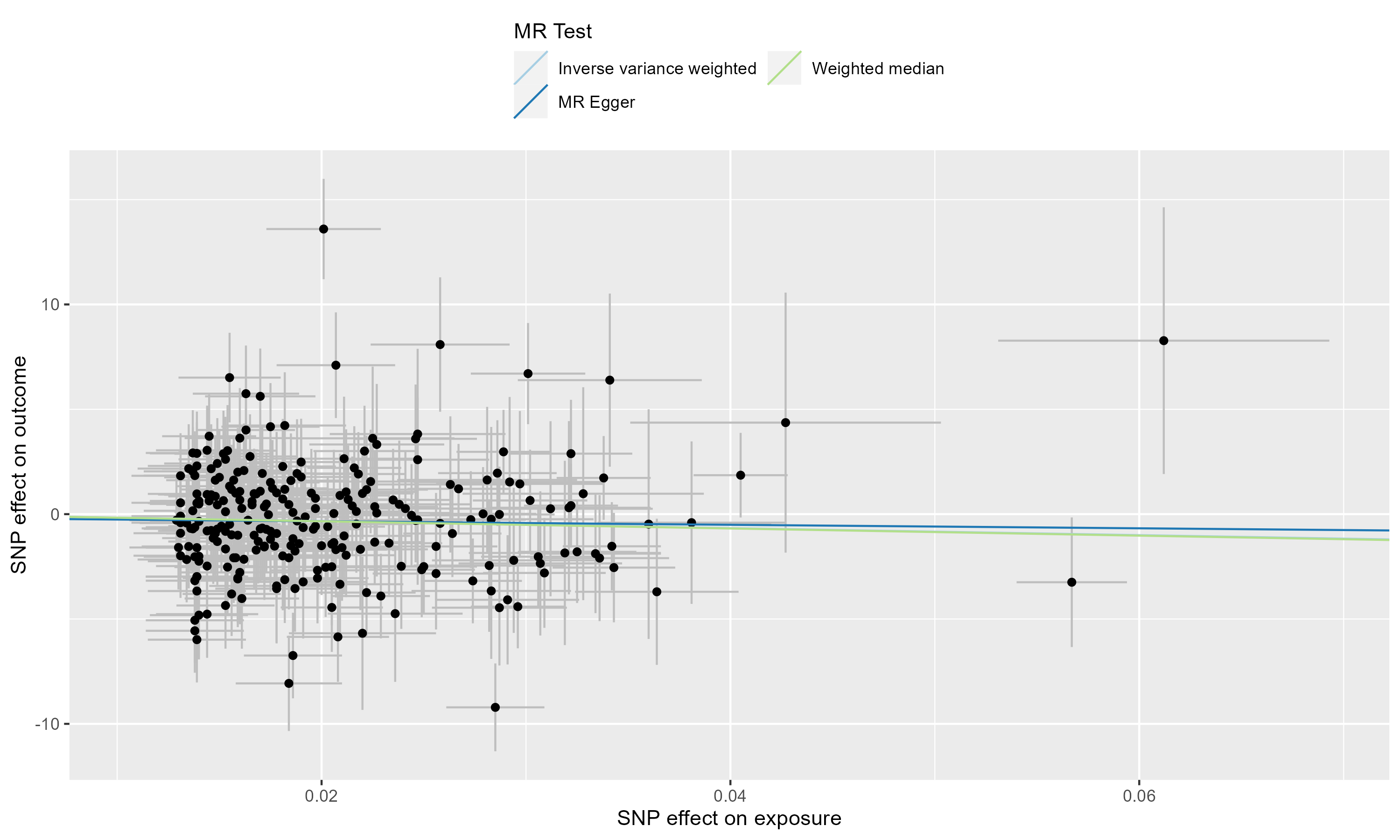

Supplement: Supplementary file 12 — Supplementary Material 12. [file 12890_2024_3150_MOESM12_ESM.zip › Supplementary Figure/scatter plot/Cortex Surface area/scatter_plotFEV1_caudalmiddlefrontal_surfavg.png]

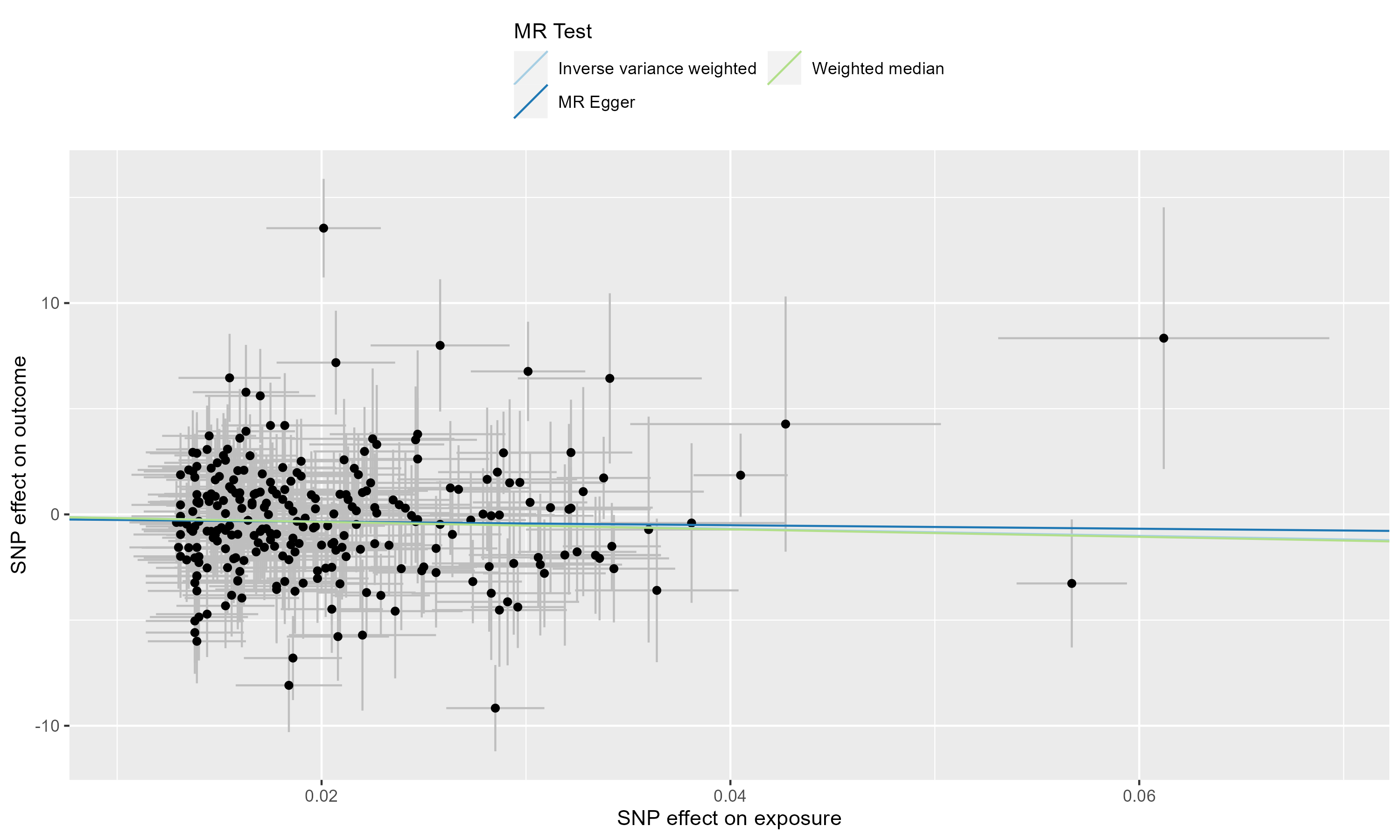

Supplement: Supplementary file 12 — Supplementary Material 12. [file 12890_2024_3150_MOESM12_ESM.zip › Supplementary Figure/scatter plot/Cortex Surface area/scatter_plotFEV1_caudalmiddlefrontal_surfavg_noGC.png]

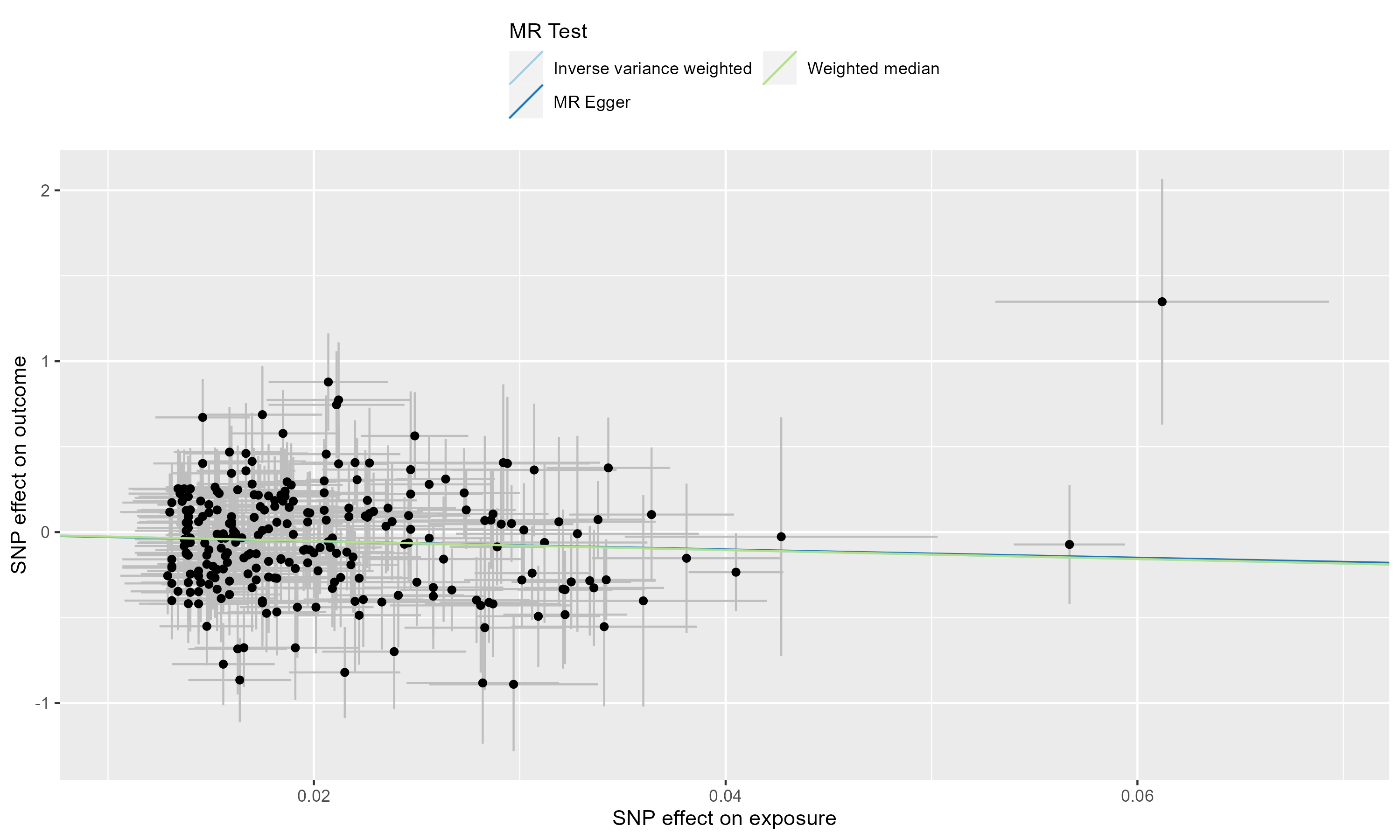

Supplement: Supplementary file 12 — Supplementary Material 12. [file 12890_2024_3150_MOESM12_ESM.zip › Supplementary Figure/scatter plot/Cortex Surface area/scatter_plotFEV1_frontalpole_surfavg.png]

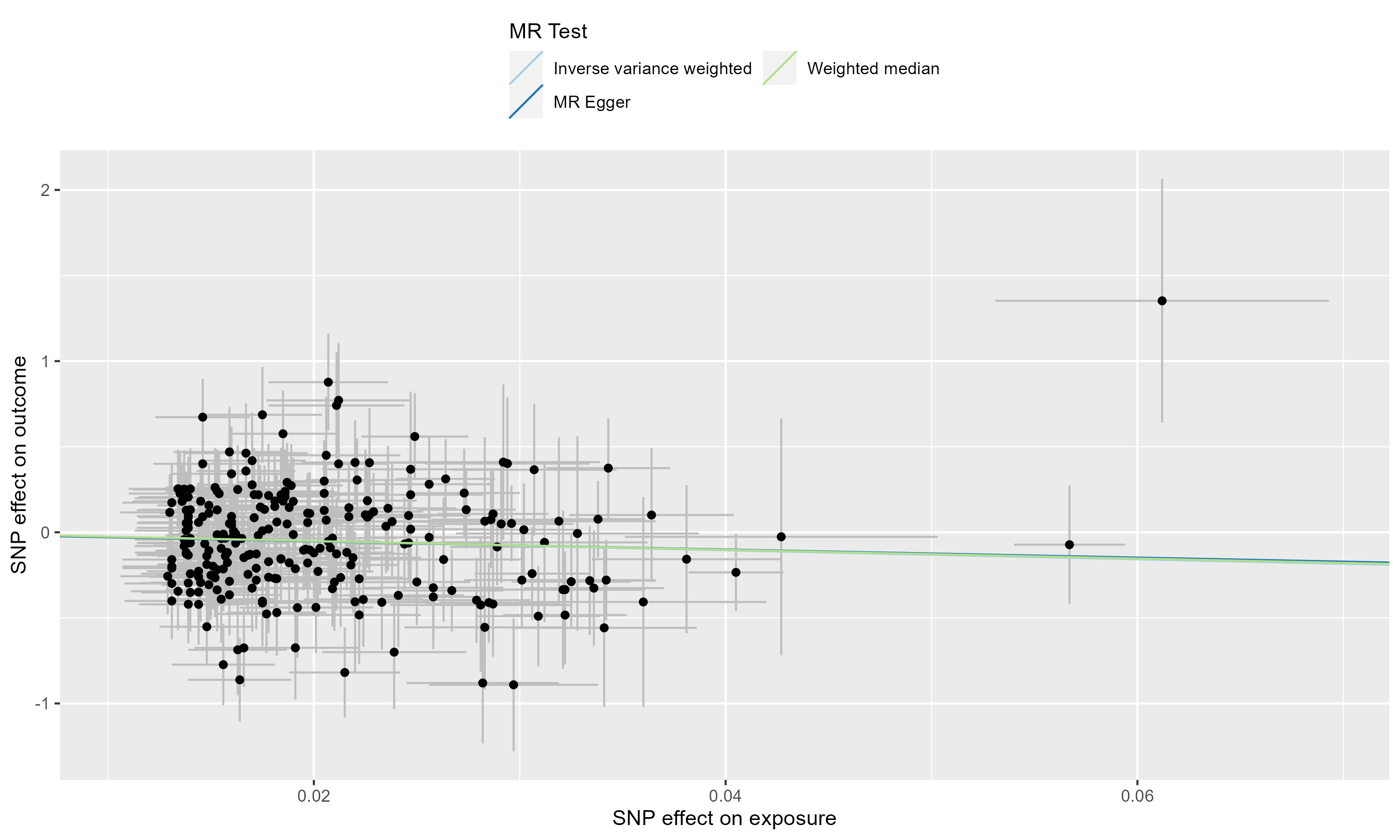

Supplement: Supplementary file 12 — Supplementary Material 12. [file 12890_2024_3150_MOESM12_ESM.zip › Supplementary Figure/scatter plot/Cortex Surface area/scatter_plotFEV1_frontalpole_surfavg_noGC.png]

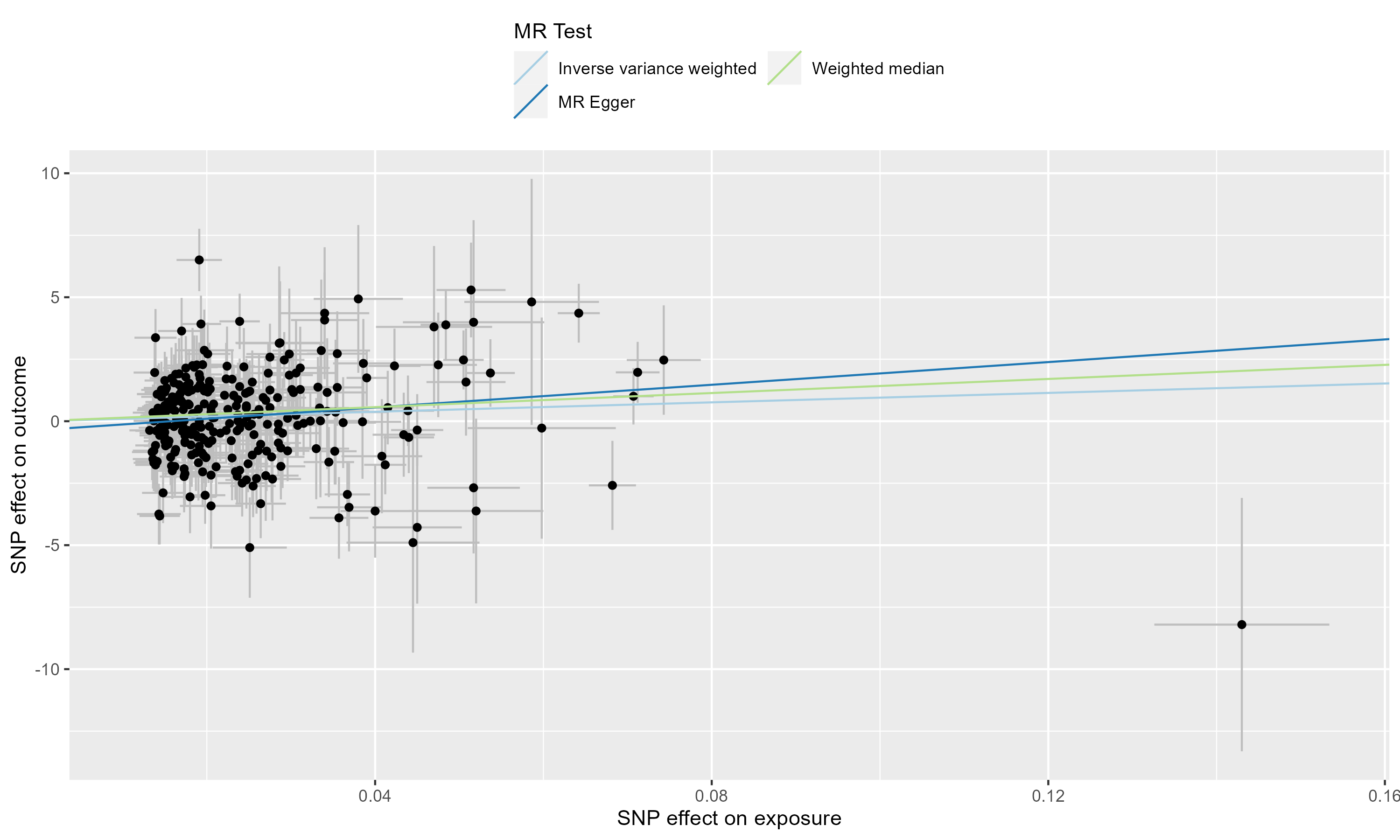

Supplement: Supplementary file 12 — Supplementary Material 12. [file 12890_2024_3150_MOESM12_ESM.zip › Supplementary Figure/scatter plot/Cortex Surface area/scatter_plotFEV1_FVC_paracentral_surfavg.png]

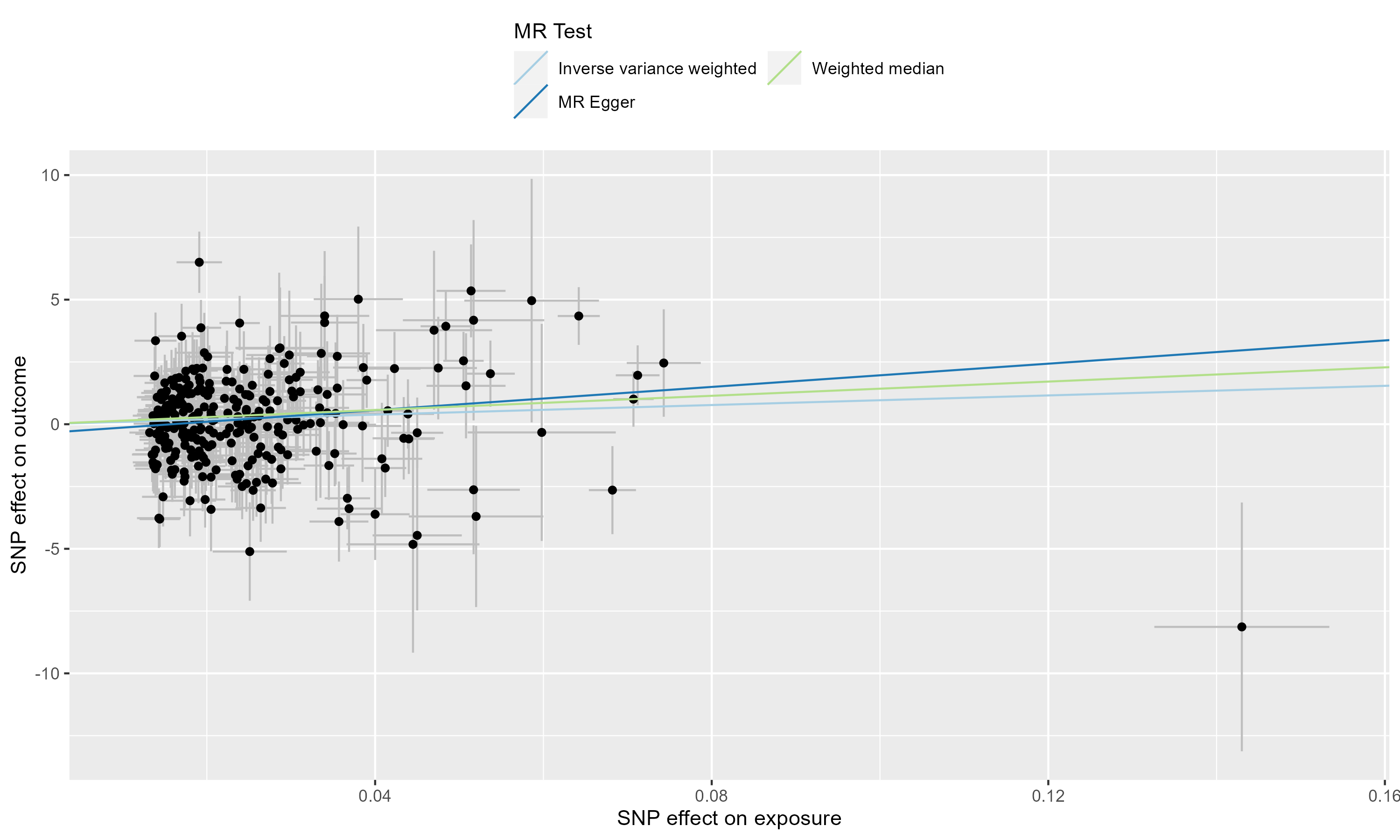

Supplement: Supplementary file 12 — Supplementary Material 12. [file 12890_2024_3150_MOESM12_ESM.zip › Supplementary Figure/scatter plot/Cortex Surface area/scatter_plotFEV1_FVC_paracentral_surfavg_noGC.png]

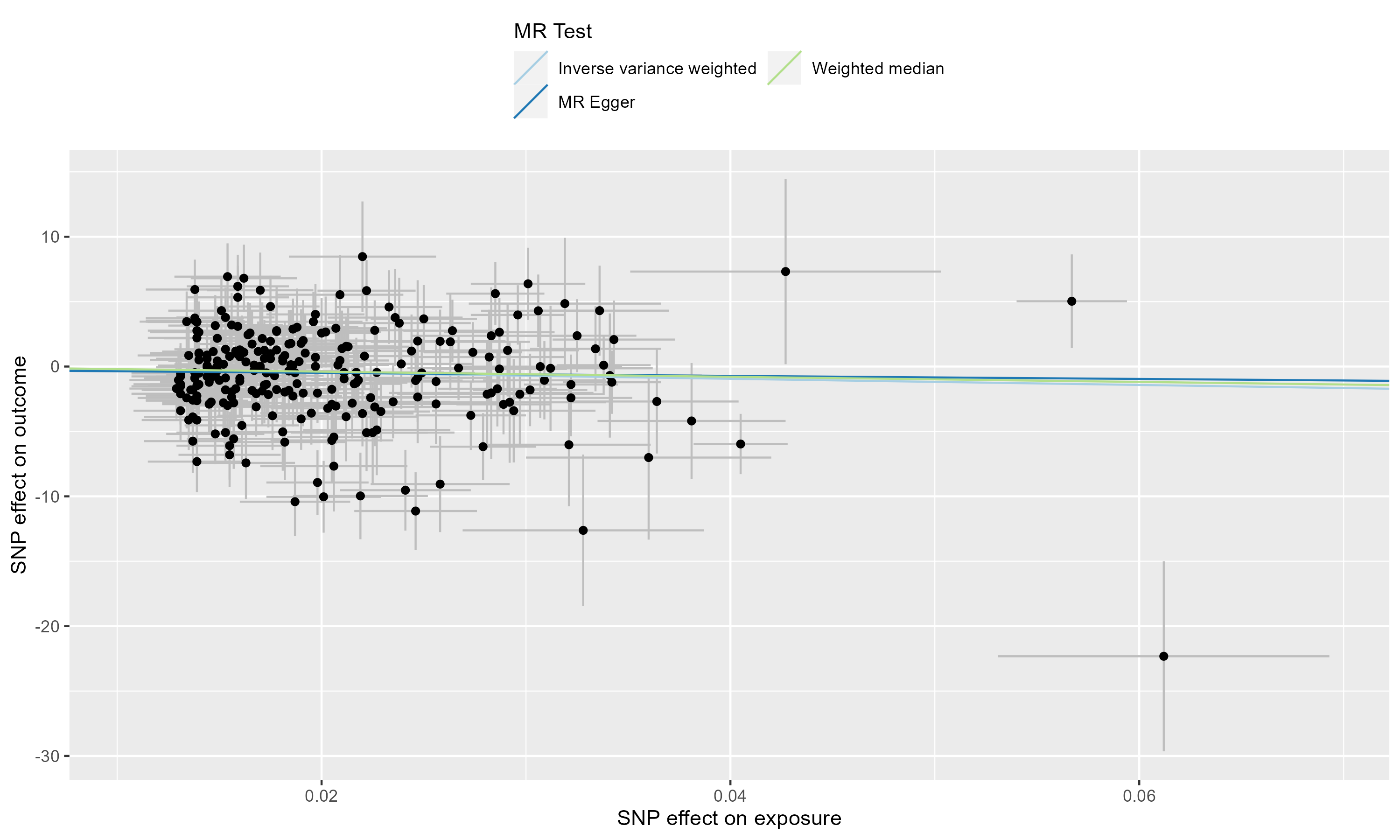

Supplement: Supplementary file 12 — Supplementary Material 12. [file 12890_2024_3150_MOESM12_ESM.zip › Supplementary Figure/scatter plot/Cortex Surface area/scatter_plotFEV1_lingual_surfavg.png]

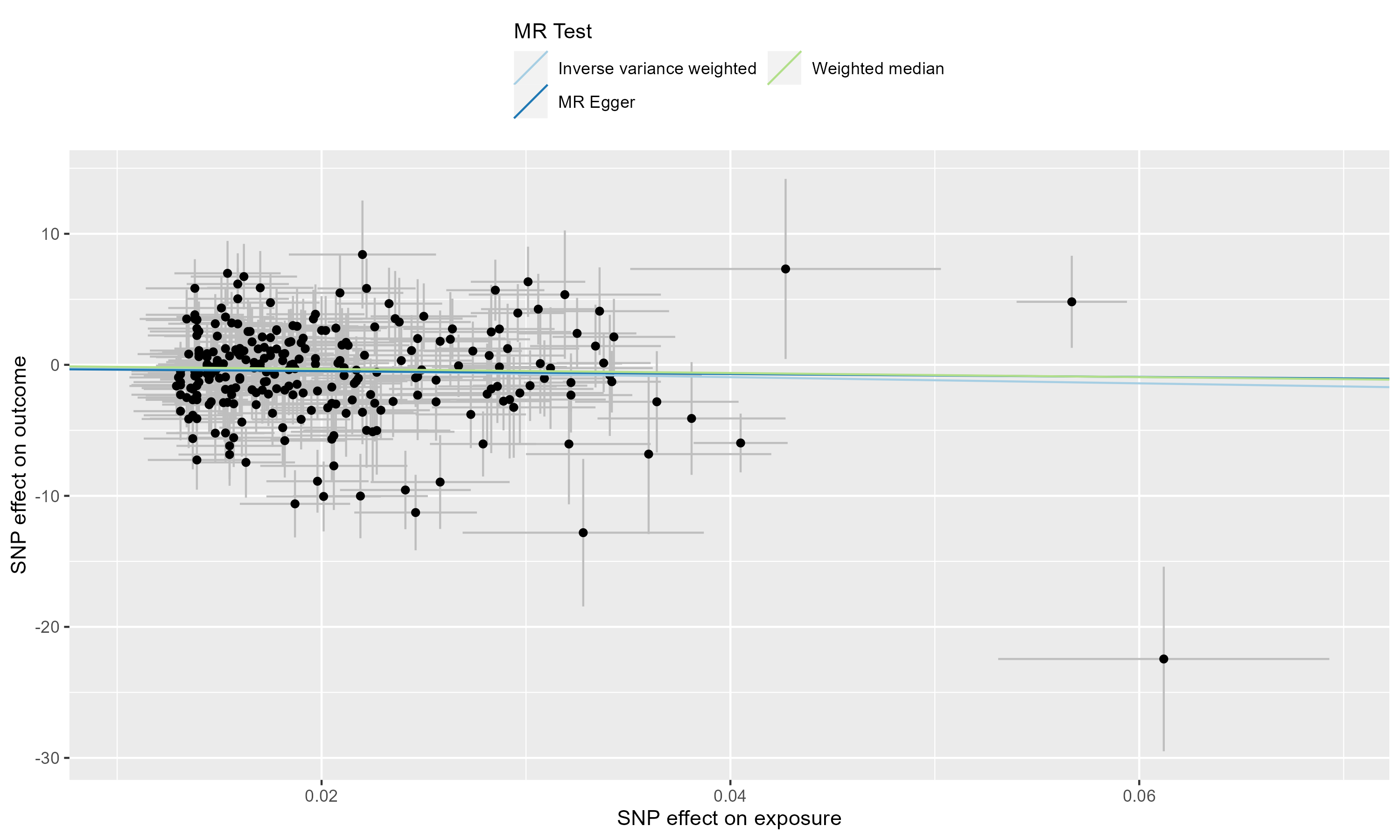

Supplement: Supplementary file 12 — Supplementary Material 12. [file 12890_2024_3150_MOESM12_ESM.zip › Supplementary Figure/scatter plot/Cortex Surface area/scatter_plotFEV1_lingual_surfavg_noGC.png]

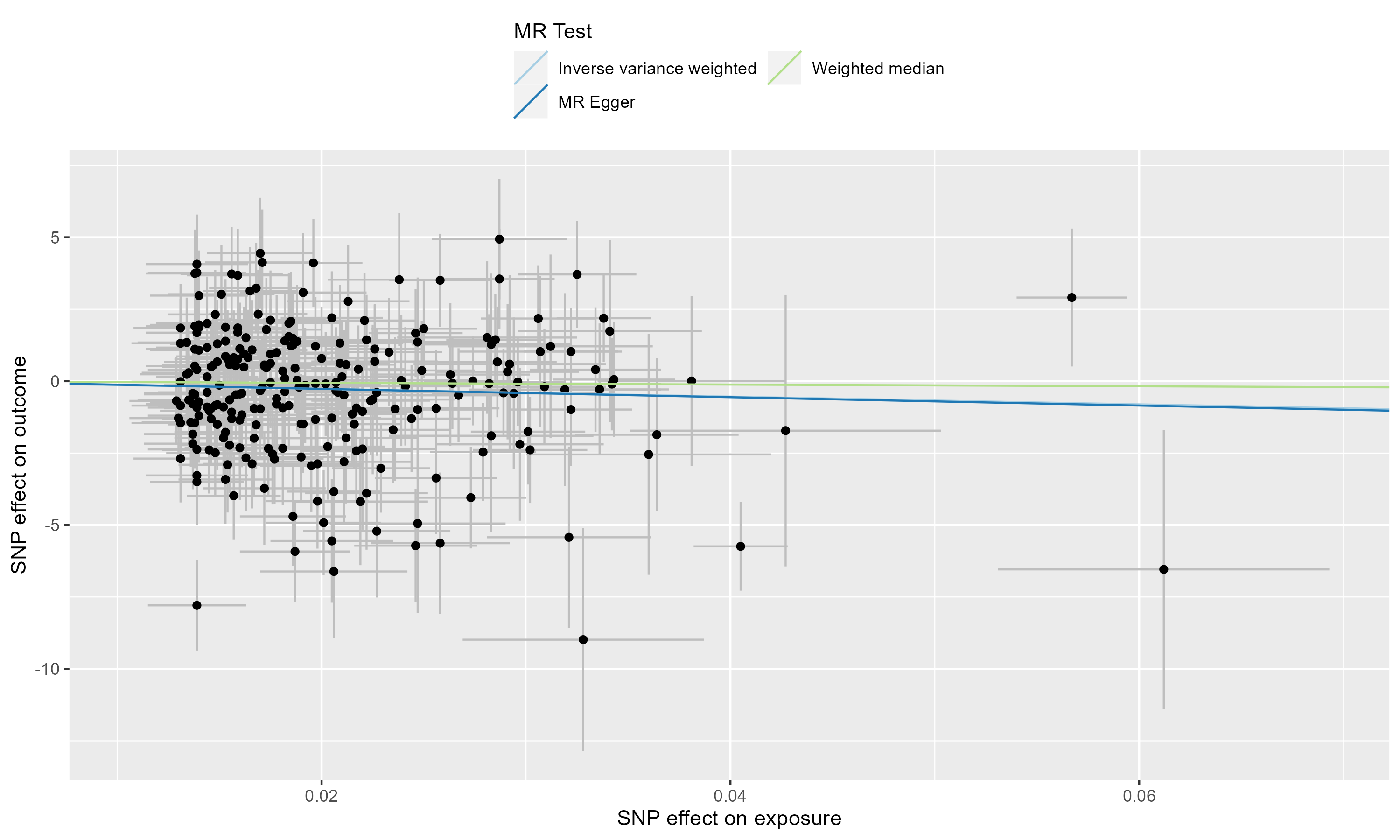

Supplement: Supplementary file 12 — Supplementary Material 12. [file 12890_2024_3150_MOESM12_ESM.zip › Supplementary Figure/scatter plot/Cortex Surface area/scatter_plotFEV1_pericalcarine_surfavg.png]

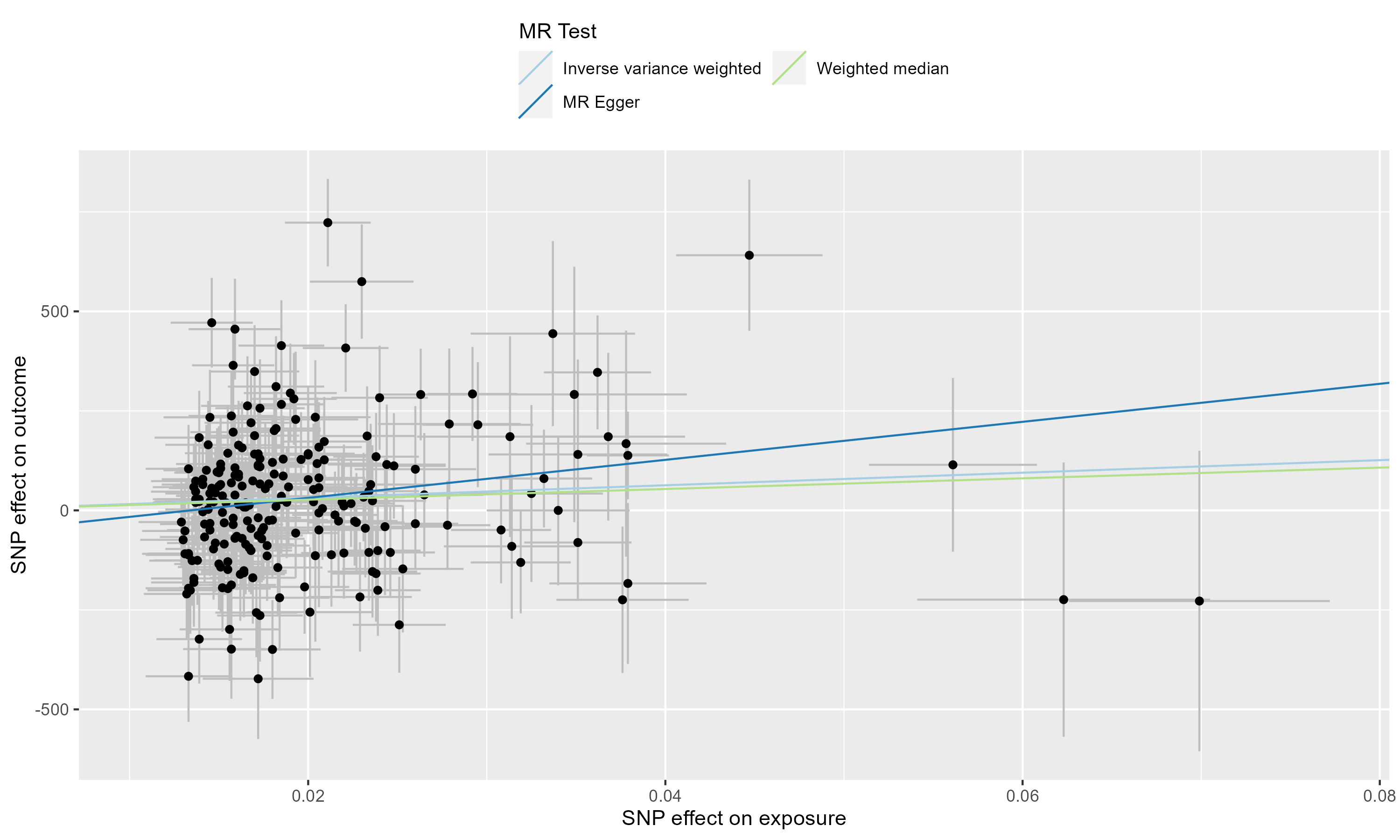

Supplement: Supplementary file 12 — Supplementary Material 12. [file 12890_2024_3150_MOESM12_ESM.zip › Supplementary Figure/scatter plot/Cortex Surface area/scatter_plotFVC_Full_SurfArea.png]

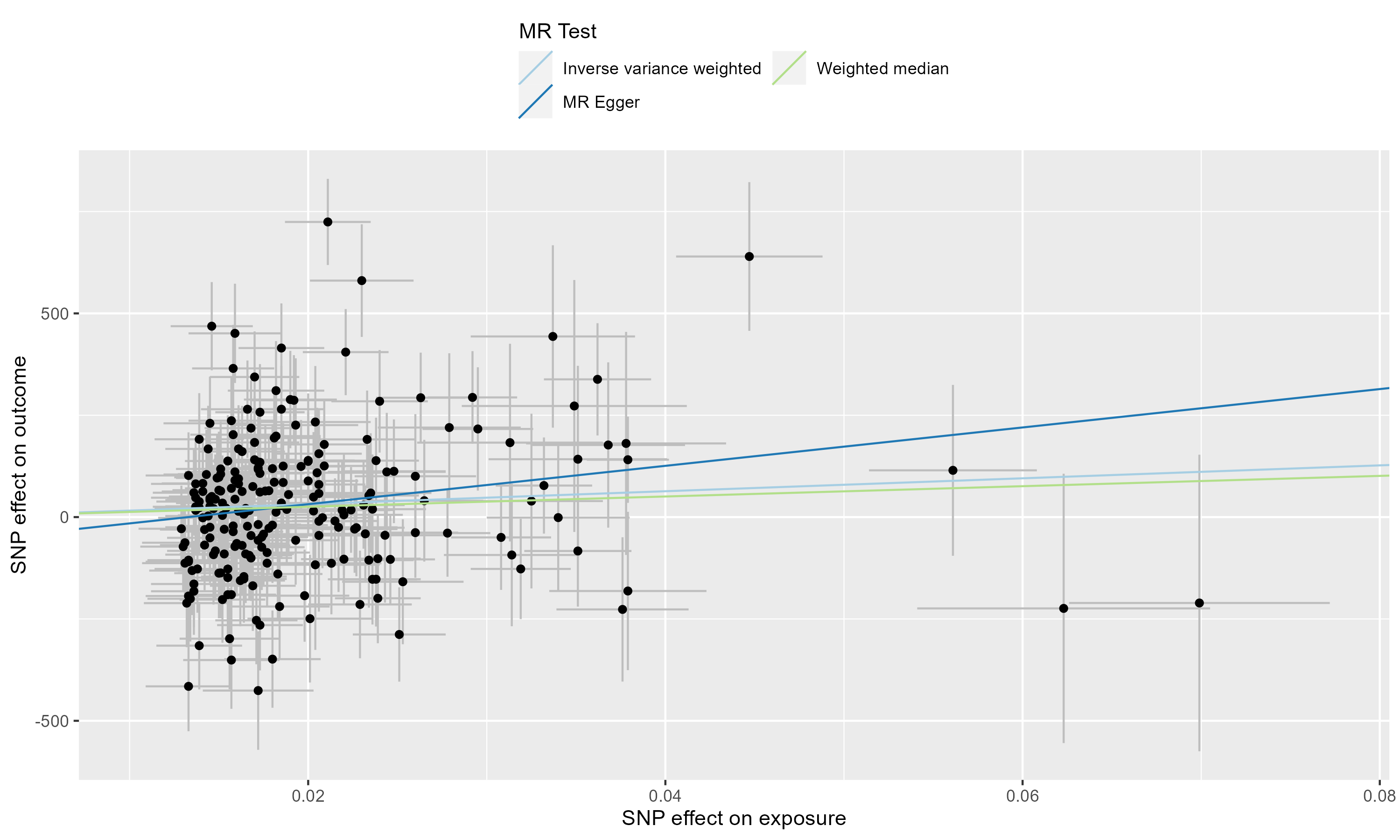

Supplement: Supplementary file 12 — Supplementary Material 12. [file 12890_2024_3150_MOESM12_ESM.zip › Supplementary Figure/scatter plot/Cortex Surface area/scatter_plotFVC_Full_SurfArea_noGC.png]

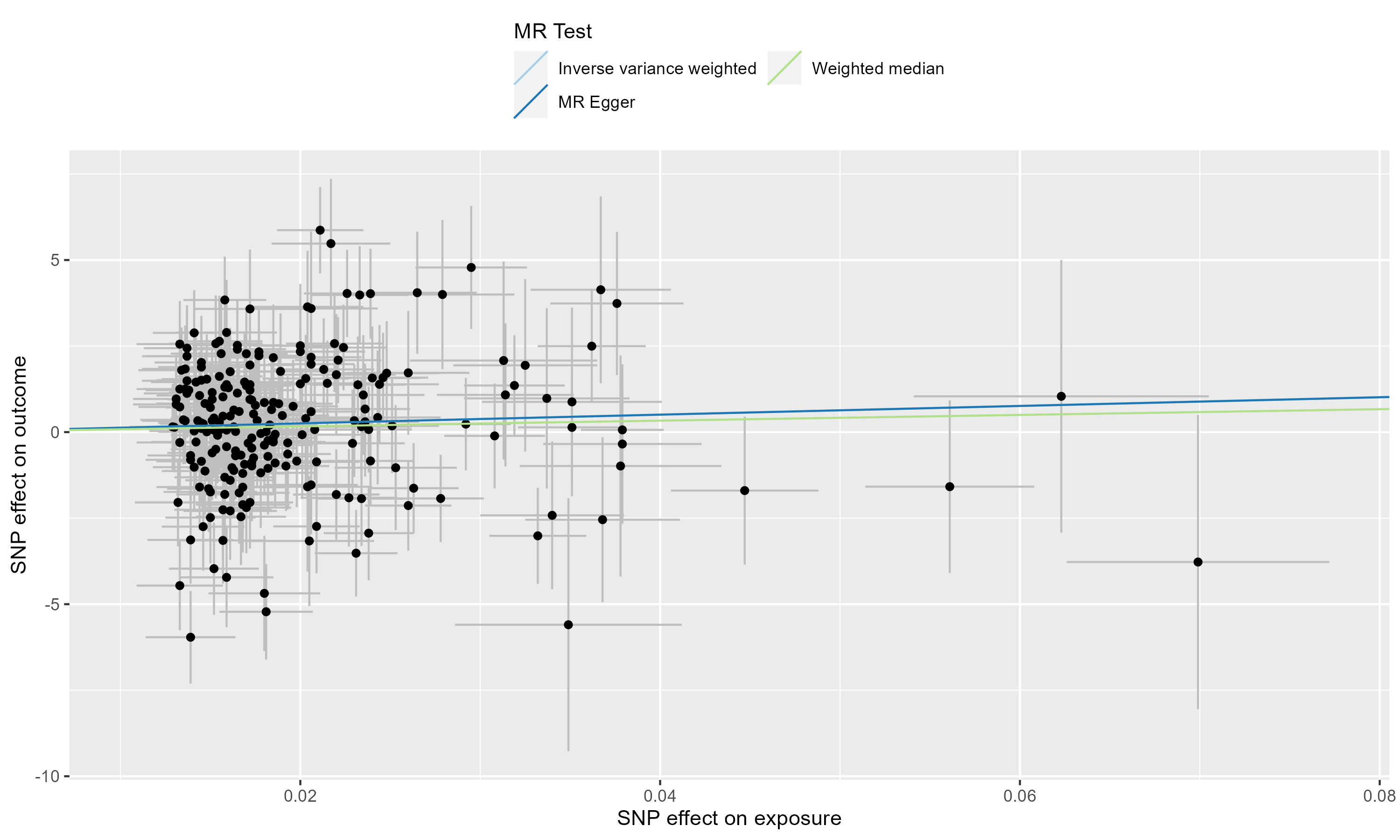

Supplement: Supplementary file 12 — Supplementary Material 12. [file 12890_2024_3150_MOESM12_ESM.zip › Supplementary Figure/scatter plot/Cortex Surface area/scatter_plotFVC_insula_surfavg_noGC.png]

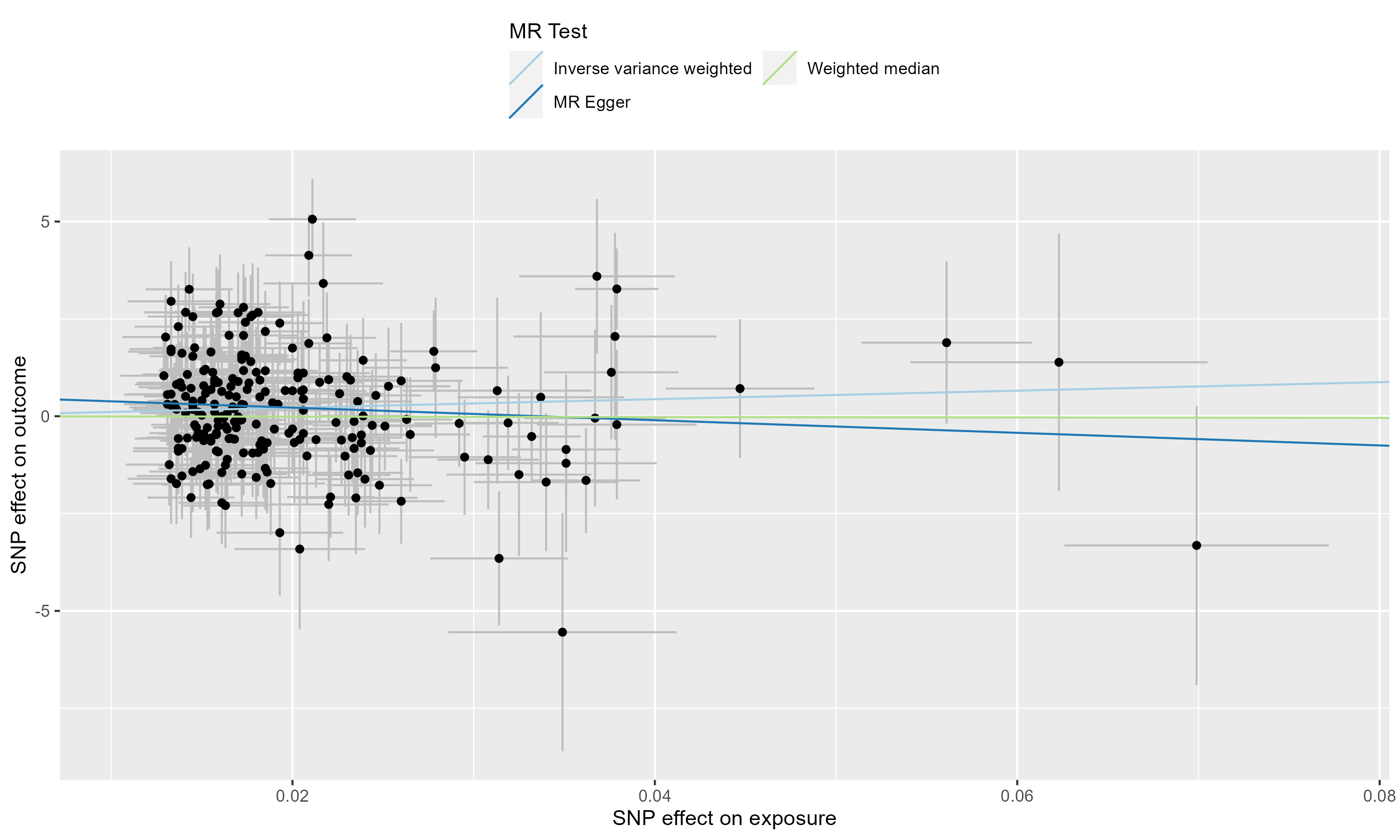

Supplement: Supplementary file 12 — Supplementary Material 12. [file 12890_2024_3150_MOESM12_ESM.zip › Supplementary Figure/scatter plot/Cortex Surface area/scatter_plotFVC_medialorbitofrontal_surfavg.png]

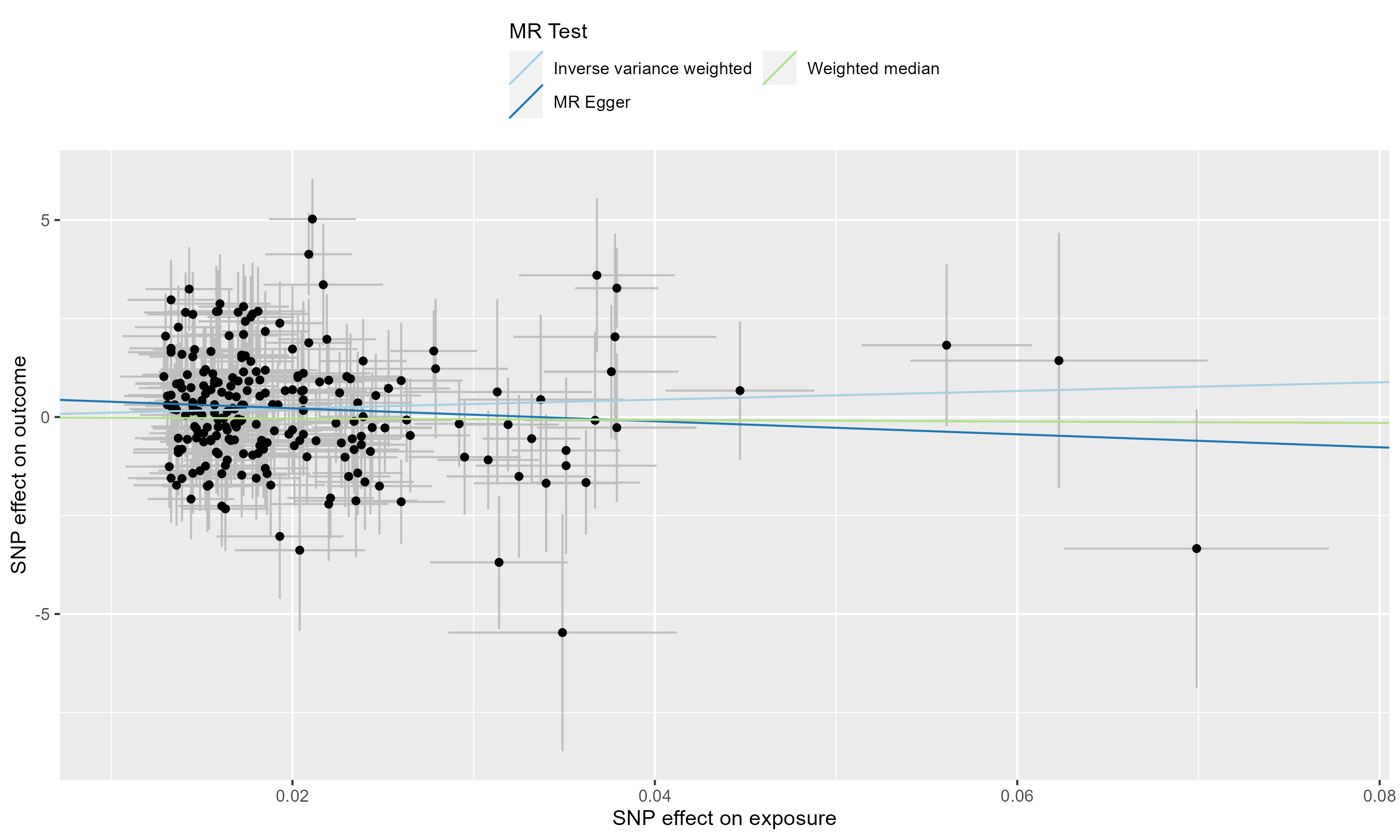

Supplement: Supplementary file 12 — Supplementary Material 12. [file 12890_2024_3150_MOESM12_ESM.zip › Supplementary Figure/scatter plot/Cortex Surface area/scatter_plotFVC_medialorbitofrontal_surfavg_noGC.png]

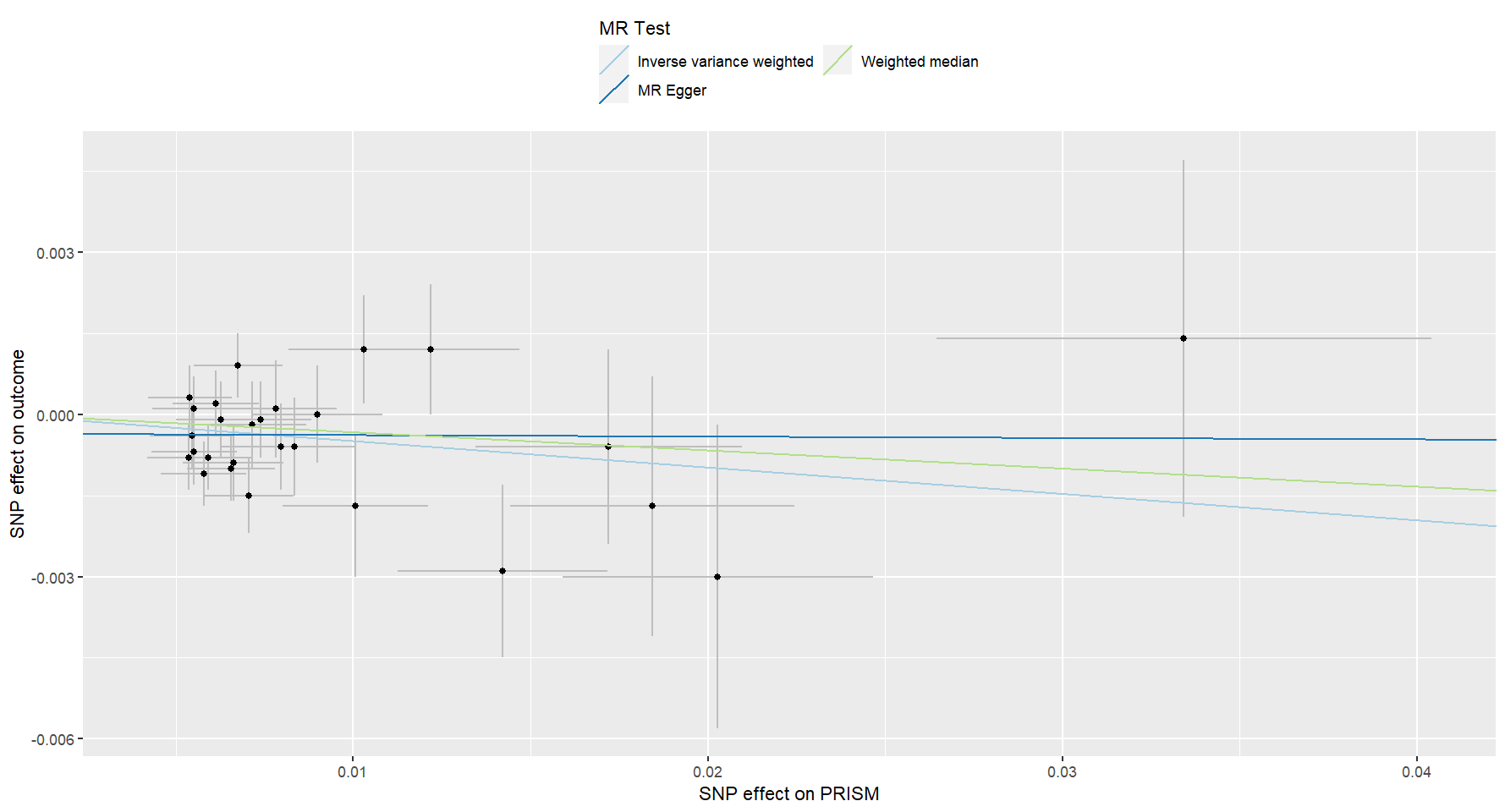

Supplement: Supplementary file 12 — Supplementary Material 12. [file 12890_2024_3150_MOESM12_ESM.zip › Supplementary Figure/scatter plot/Cortex Thickness/scatter_plot_PRISM_inferioparietal_thickavg_GC.png]

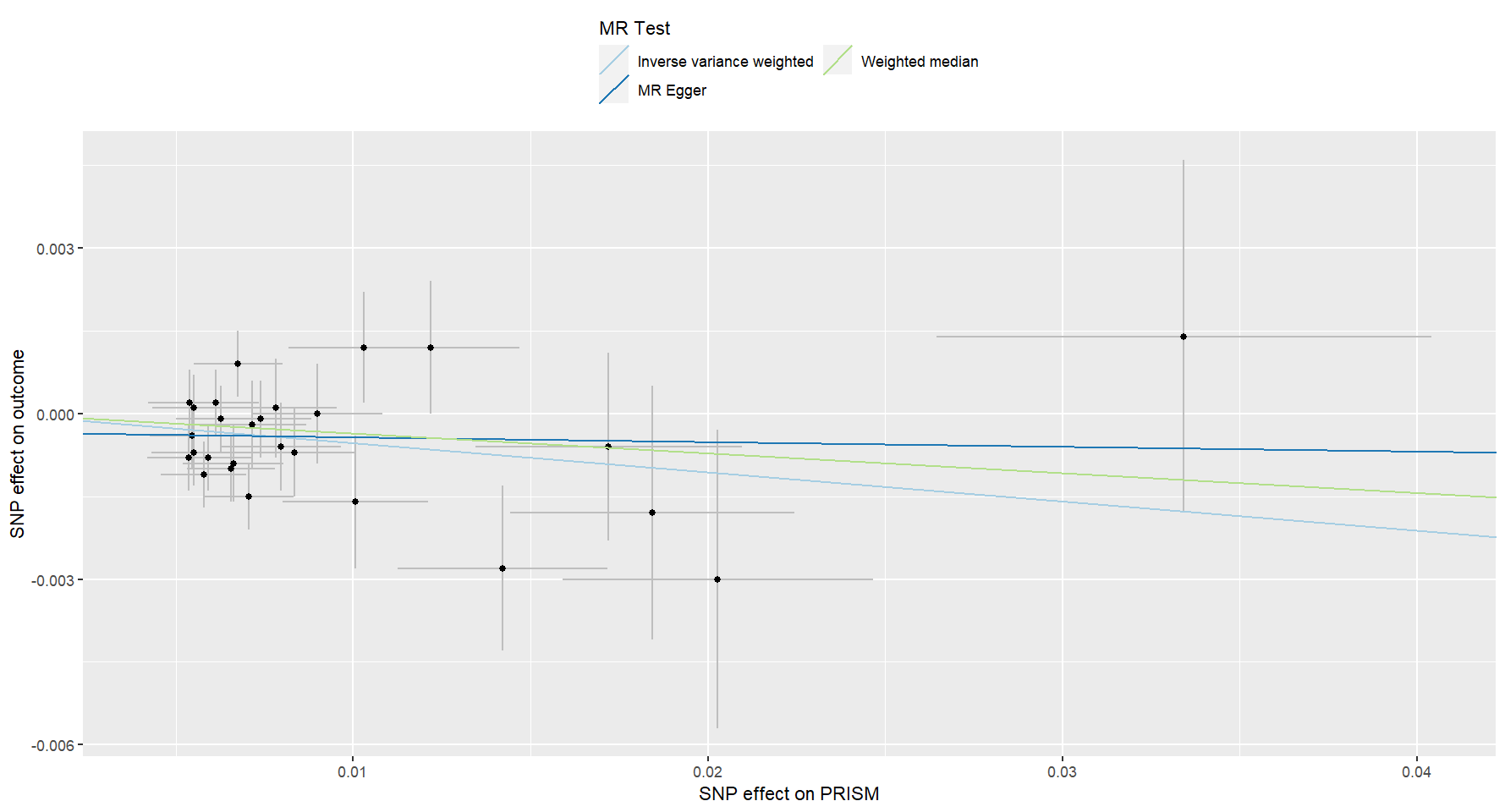

Supplement: Supplementary file 12 — Supplementary Material 12. [file 12890_2024_3150_MOESM12_ESM.zip › Supplementary Figure/scatter plot/Cortex Thickness/scatter_plot_PRISM_inferiorparietal_thickavg_noGC.png]

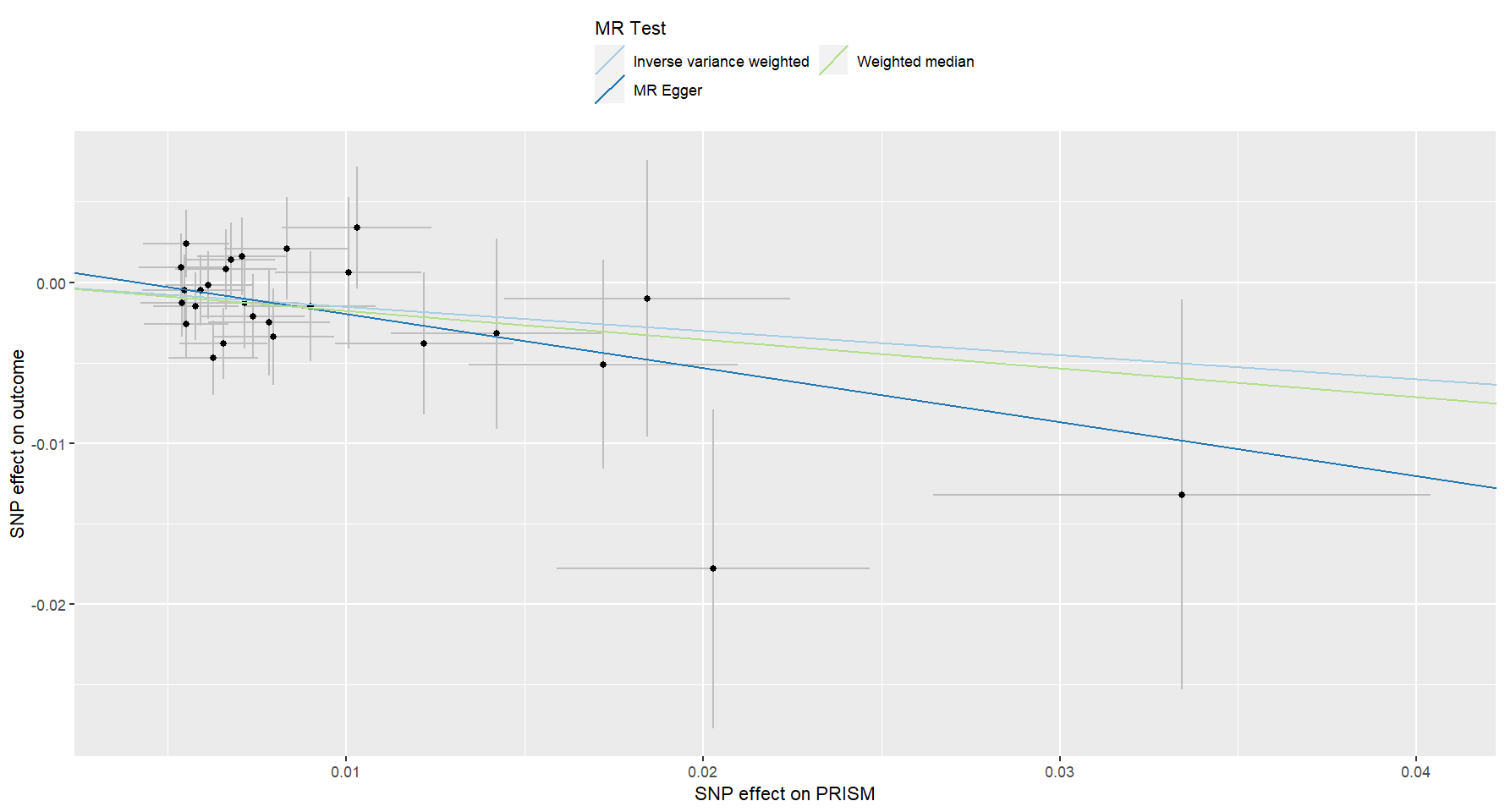

Supplement: Supplementary file 12 — Supplementary Material 12. [file 12890_2024_3150_MOESM12_ESM.zip › Supplementary Figure/scatter plot/Cortex Thickness/scatter_plot_PRISM_temporalpole_thickavg_GC.png]

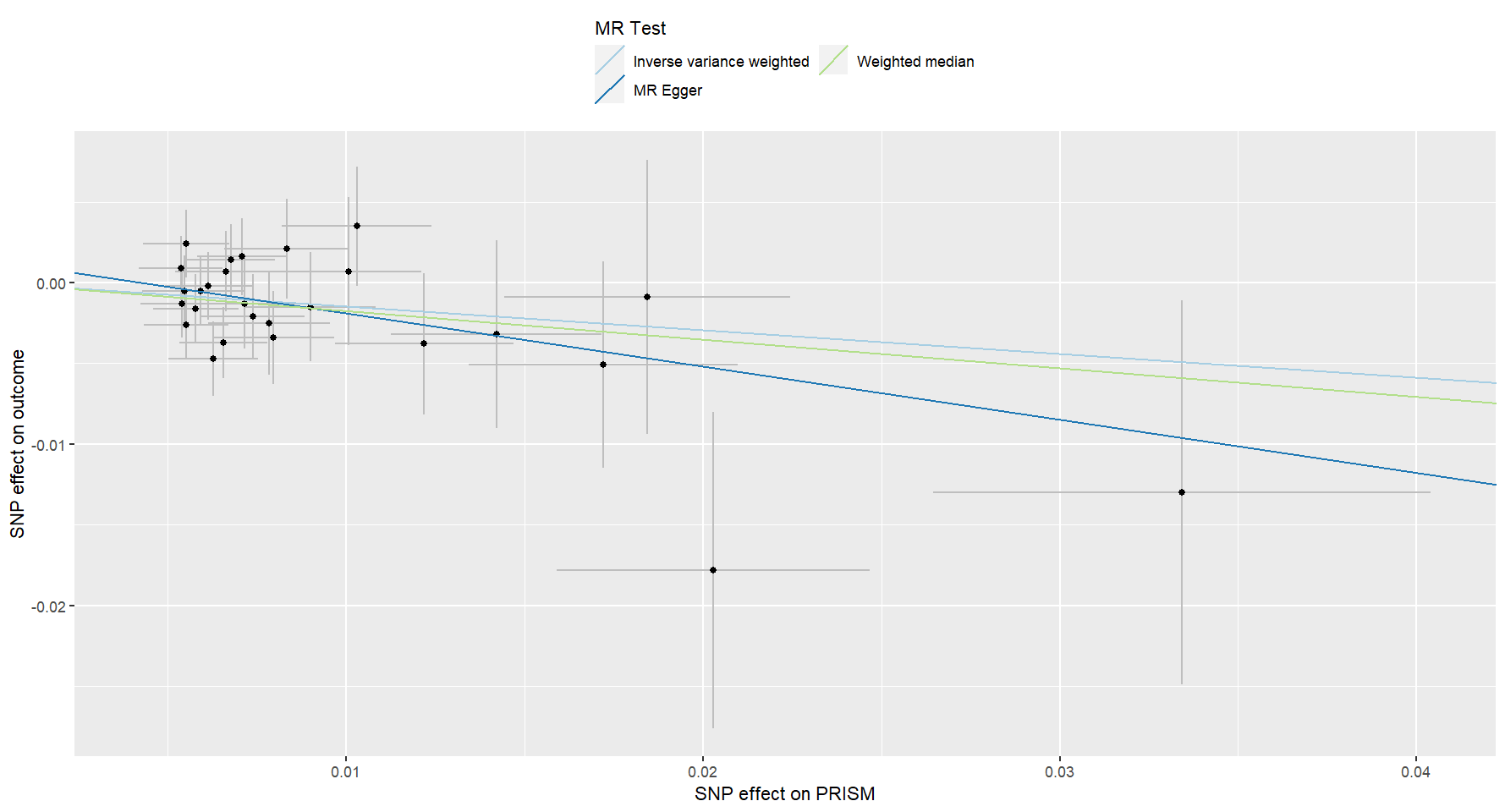

Supplement: Supplementary file 12 — Supplementary Material 12. [file 12890_2024_3150_MOESM12_ESM.zip › Supplementary Figure/scatter plot/Cortex Thickness/scatter_plot_PRISM_temporalpole_thickavg_noGC.png]

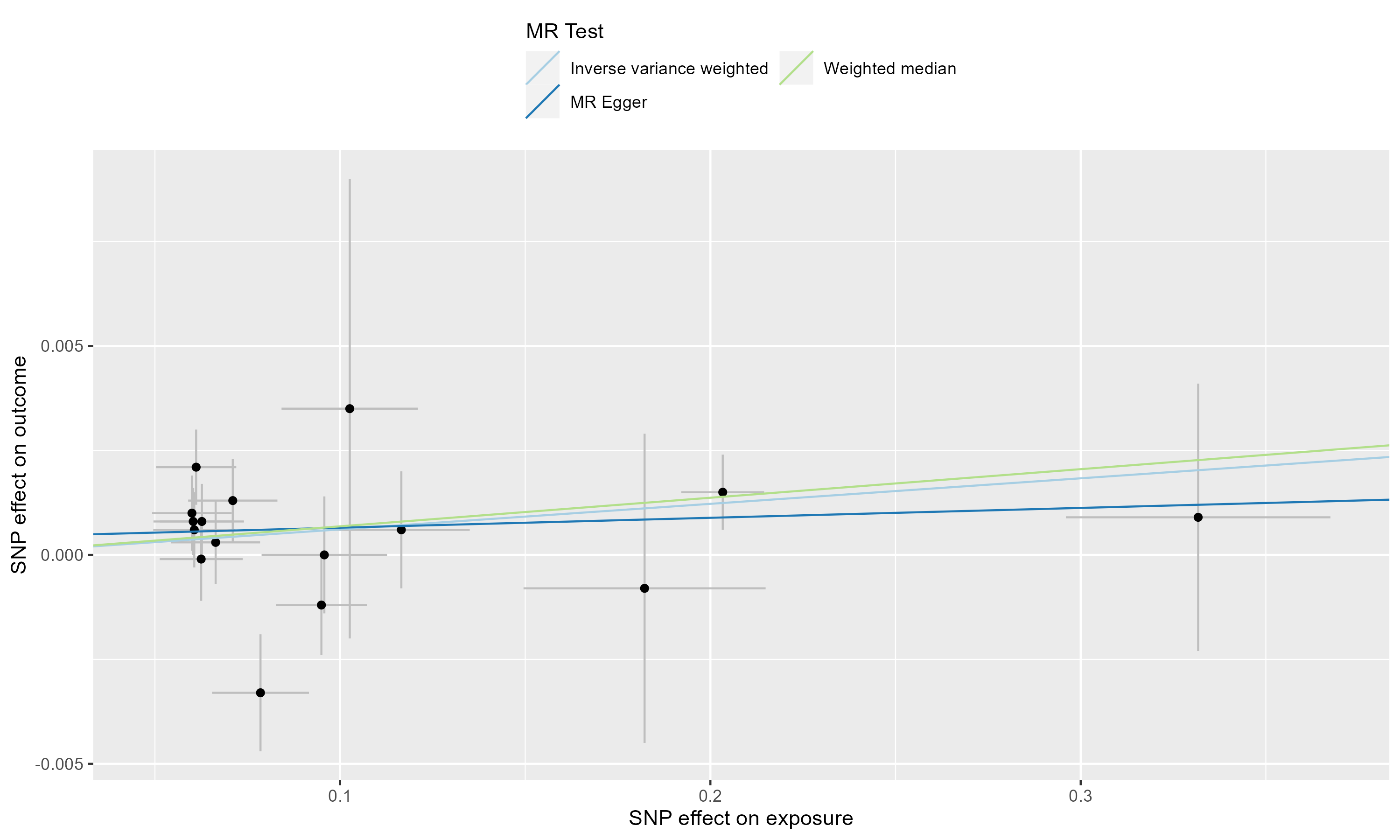

Supplement: Supplementary file 12 — Supplementary Material 12. [file 12890_2024_3150_MOESM12_ESM.zip › Supplementary Figure/scatter plot/Cortex Thickness/scatter_plotCOPD_cuneus_thickavg.png]

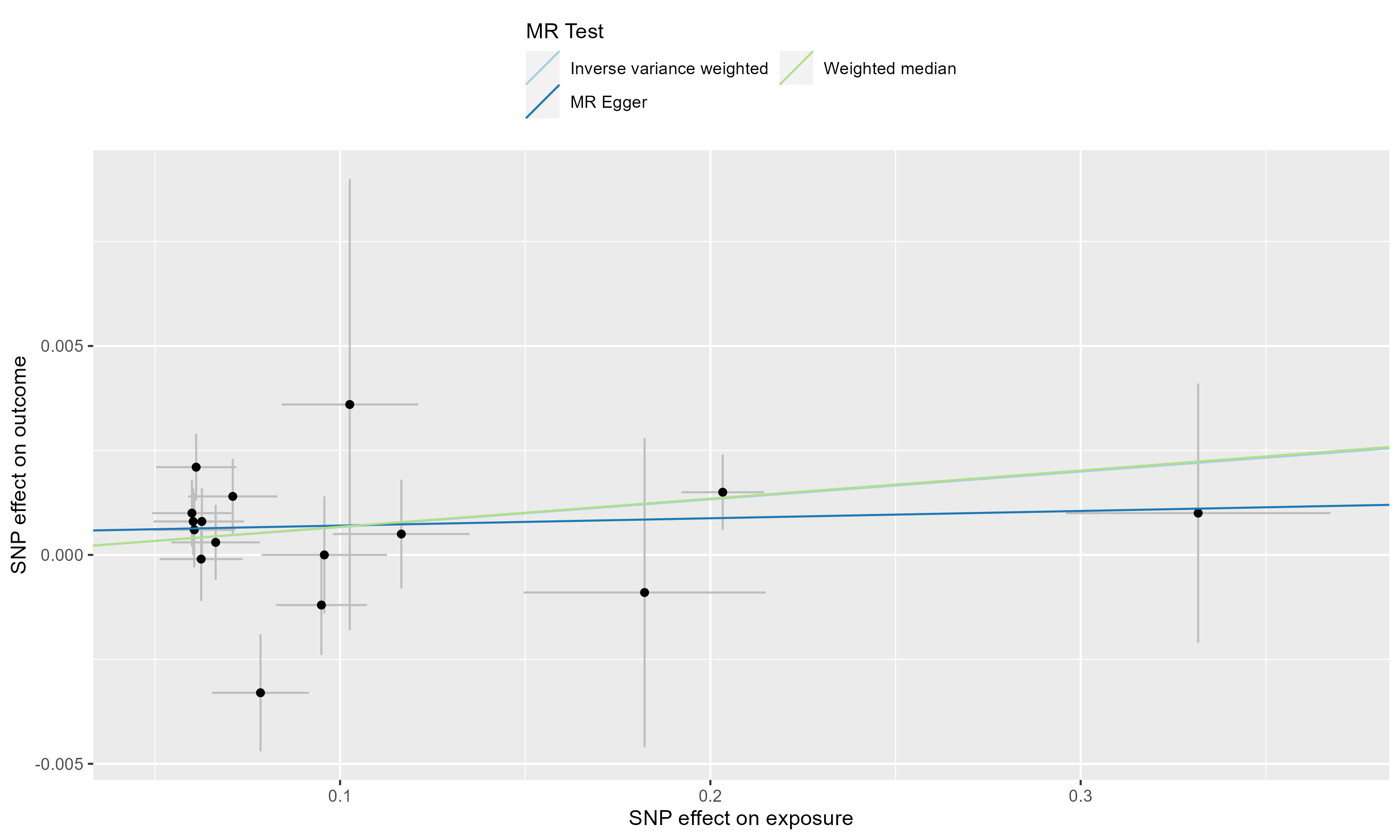

Supplement: Supplementary file 12 — Supplementary Material 12. [file 12890_2024_3150_MOESM12_ESM.zip › Supplementary Figure/scatter plot/Cortex Thickness/scatter_plotCOPD_cuneus_thickavg_noGC.png]

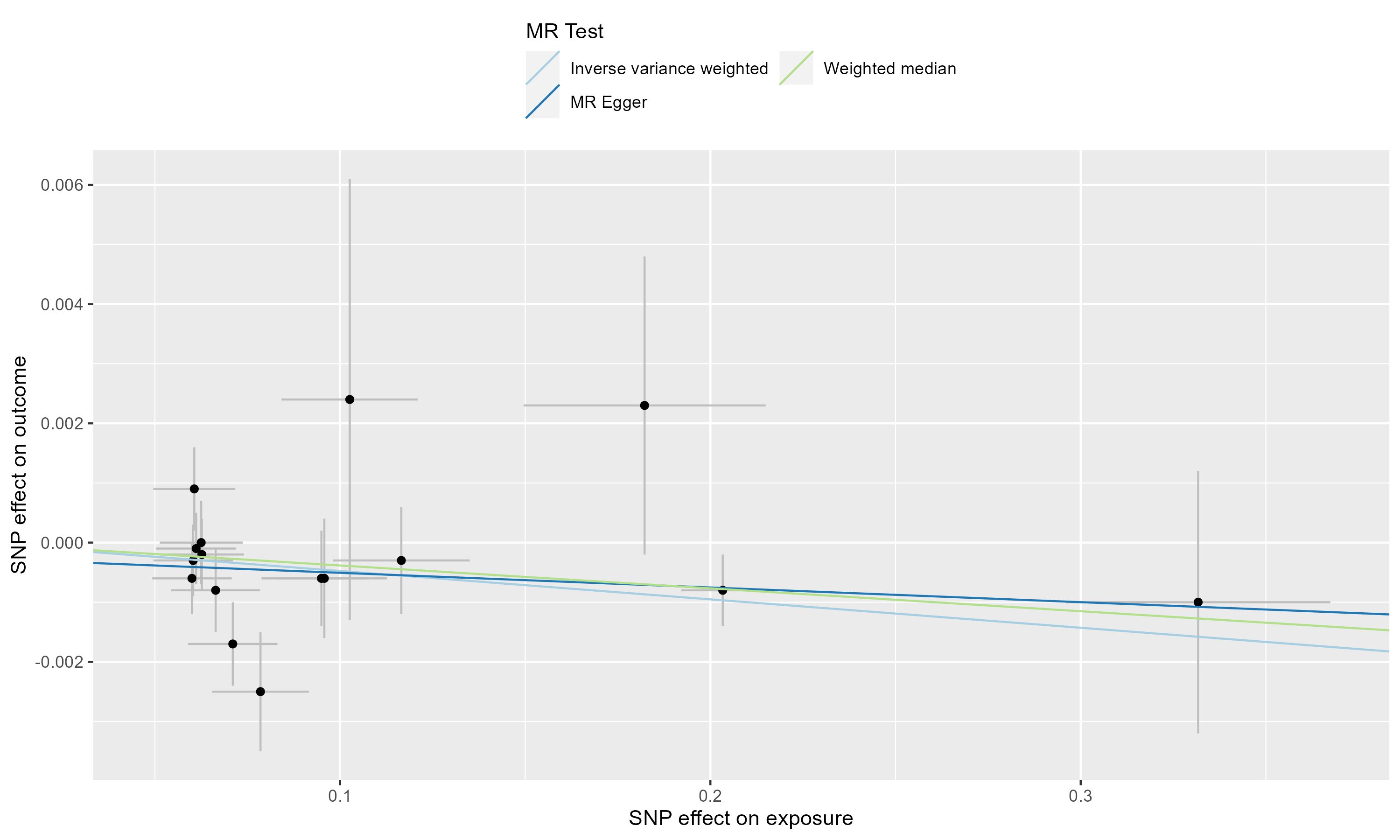

Supplement: Supplementary file 12 — Supplementary Material 12. [file 12890_2024_3150_MOESM12_ESM.zip › Supplementary Figure/scatter plot/Cortex Thickness/scatter_plotCOPD_inferiorparietal_thickavg.png]

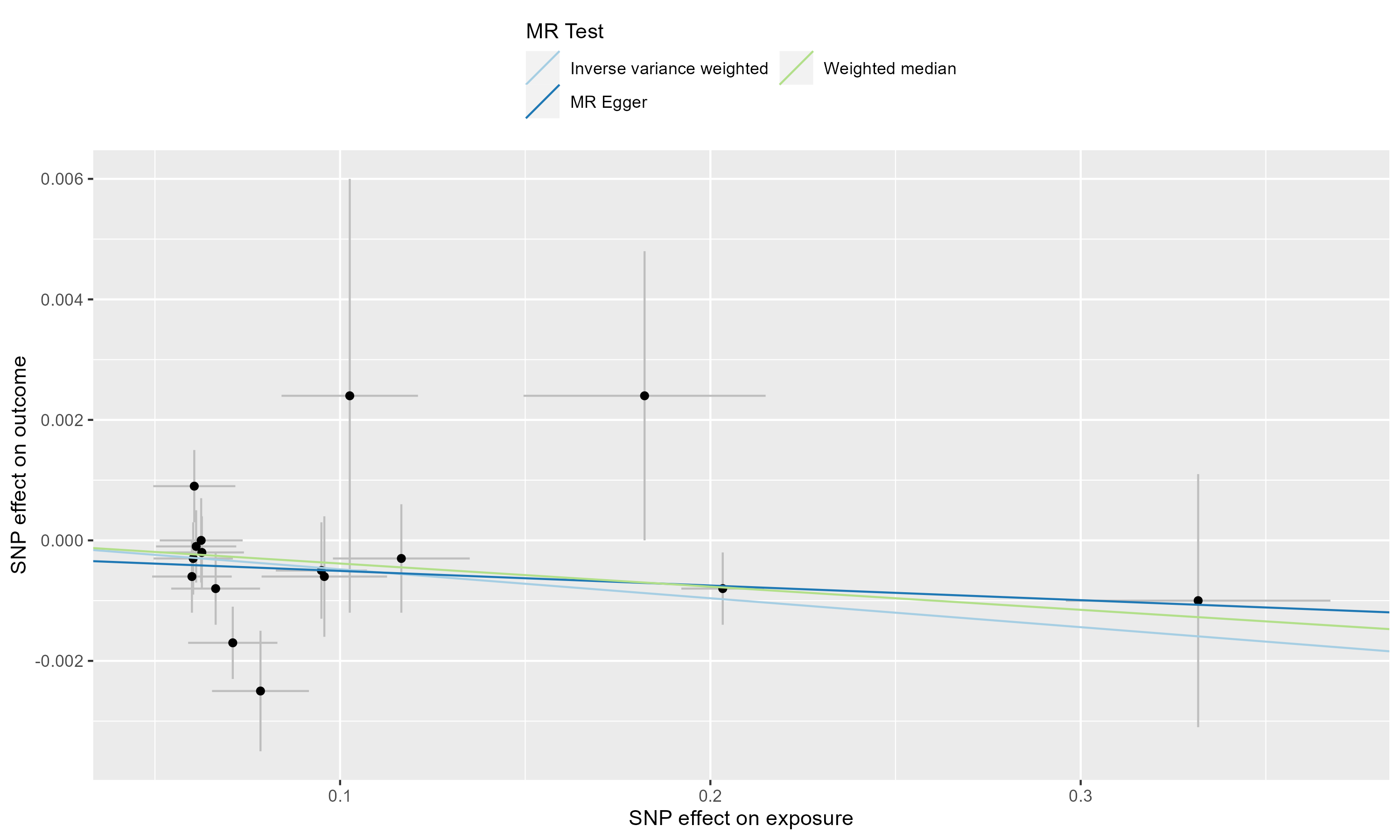

Supplement: Supplementary file 12 — Supplementary Material 12. [file 12890_2024_3150_MOESM12_ESM.zip › Supplementary Figure/scatter plot/Cortex Thickness/scatter_plotCOPD_inferiorparietal_thickavg_noGC.png]

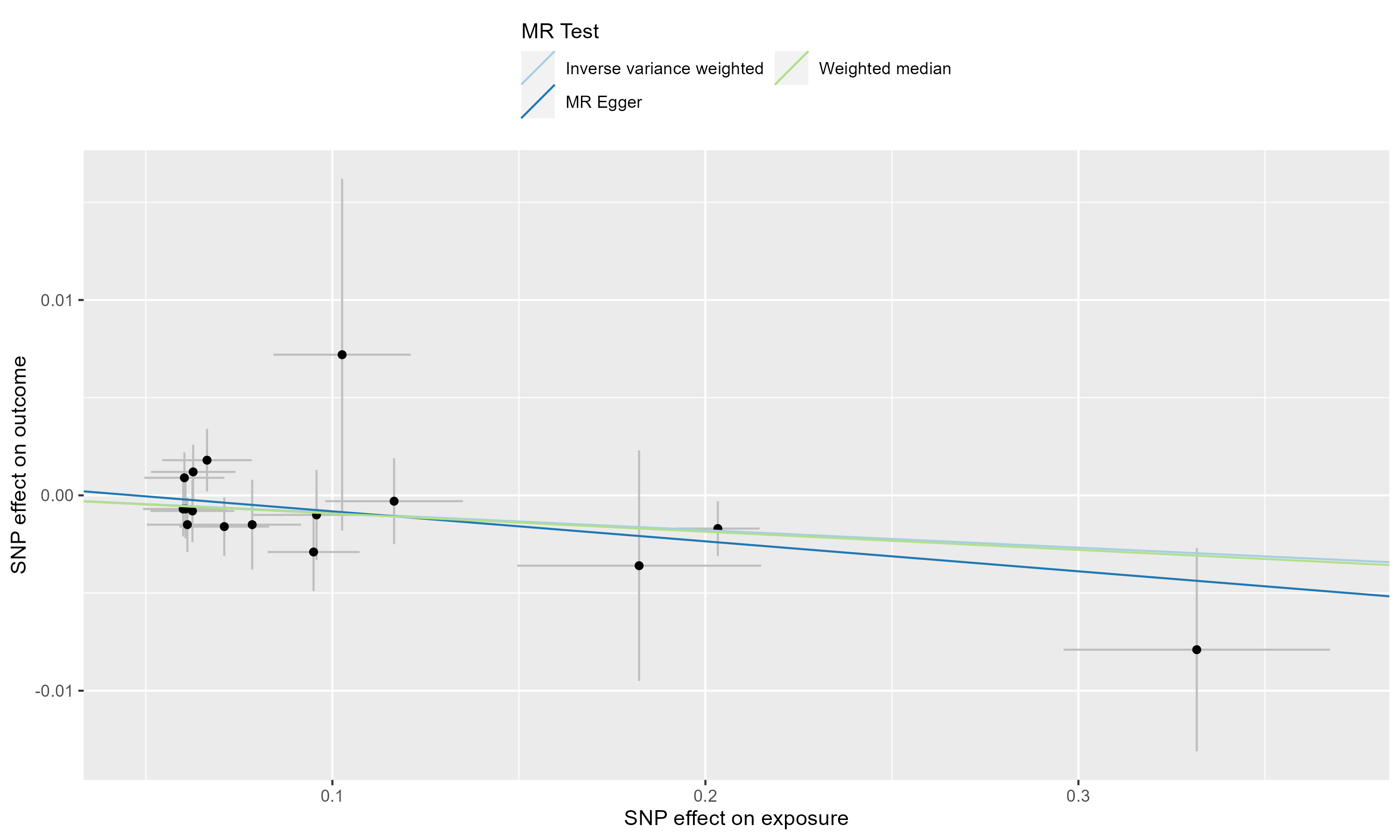

Supplement: Supplementary file 12 — Supplementary Material 12. [file 12890_2024_3150_MOESM12_ESM.zip › Supplementary Figure/scatter plot/Cortex Thickness/scatter_plotCOPD_rostralanteriorcingulate_thickavg_noGC.png]

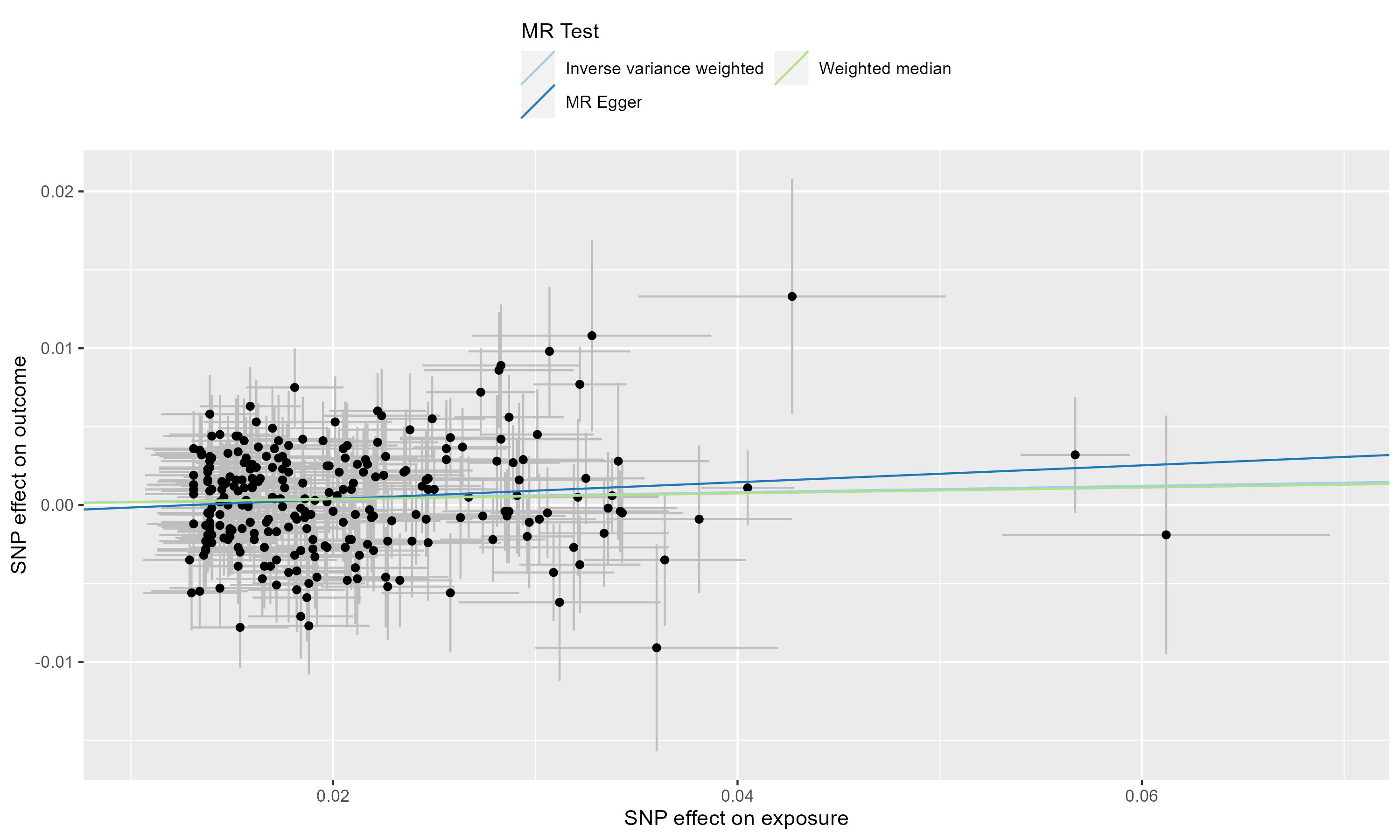

Supplement: Supplementary file 12 — Supplementary Material 12. [file 12890_2024_3150_MOESM12_ESM.zip › Supplementary Figure/scatter plot/Cortex Thickness/scatter_plotFEV1_entorhinal_thickavg.png]

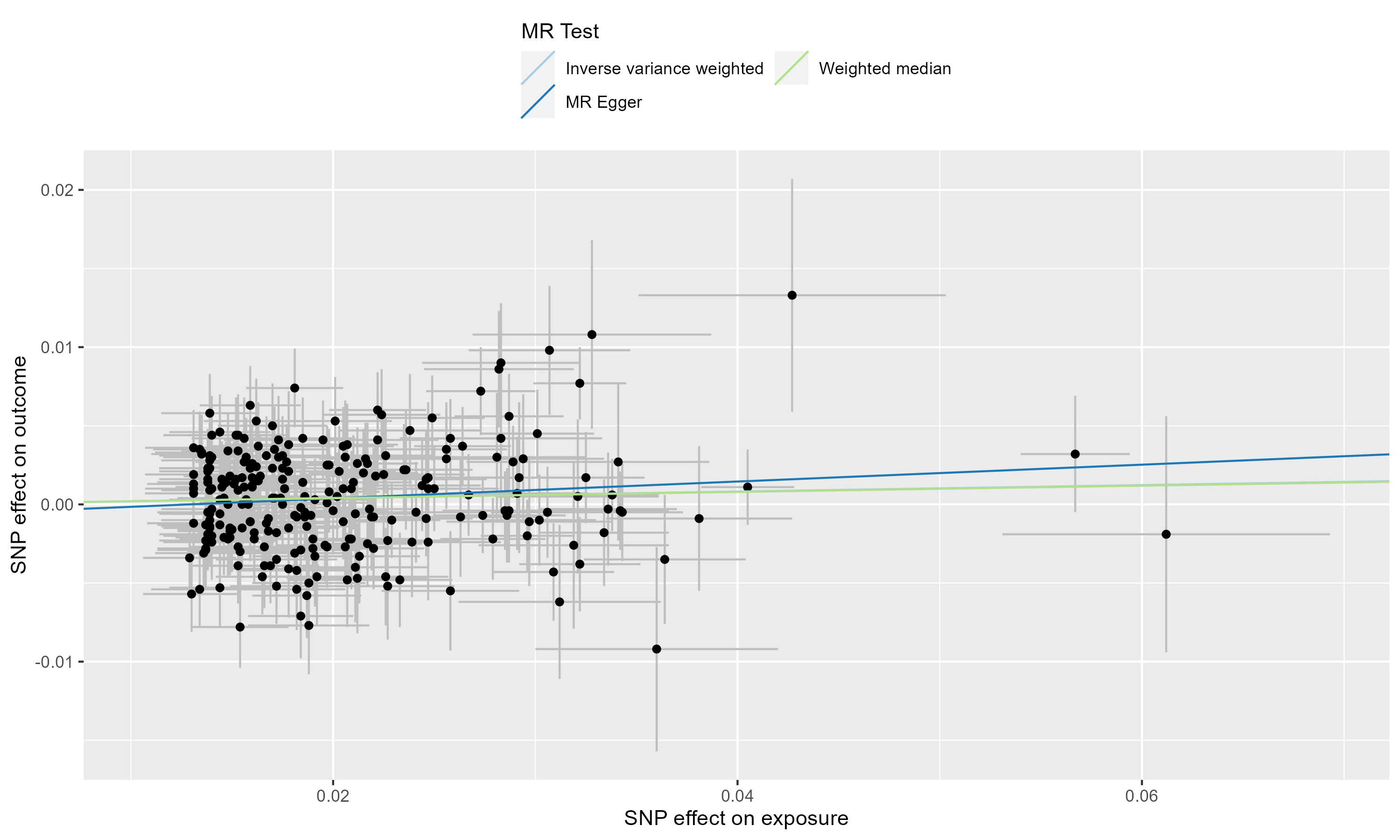

Supplement: Supplementary file 12 — Supplementary Material 12. [file 12890_2024_3150_MOESM12_ESM.zip › Supplementary Figure/scatter plot/Cortex Thickness/scatter_plotFEV1_entorhinal_thickavg_noGC.png]

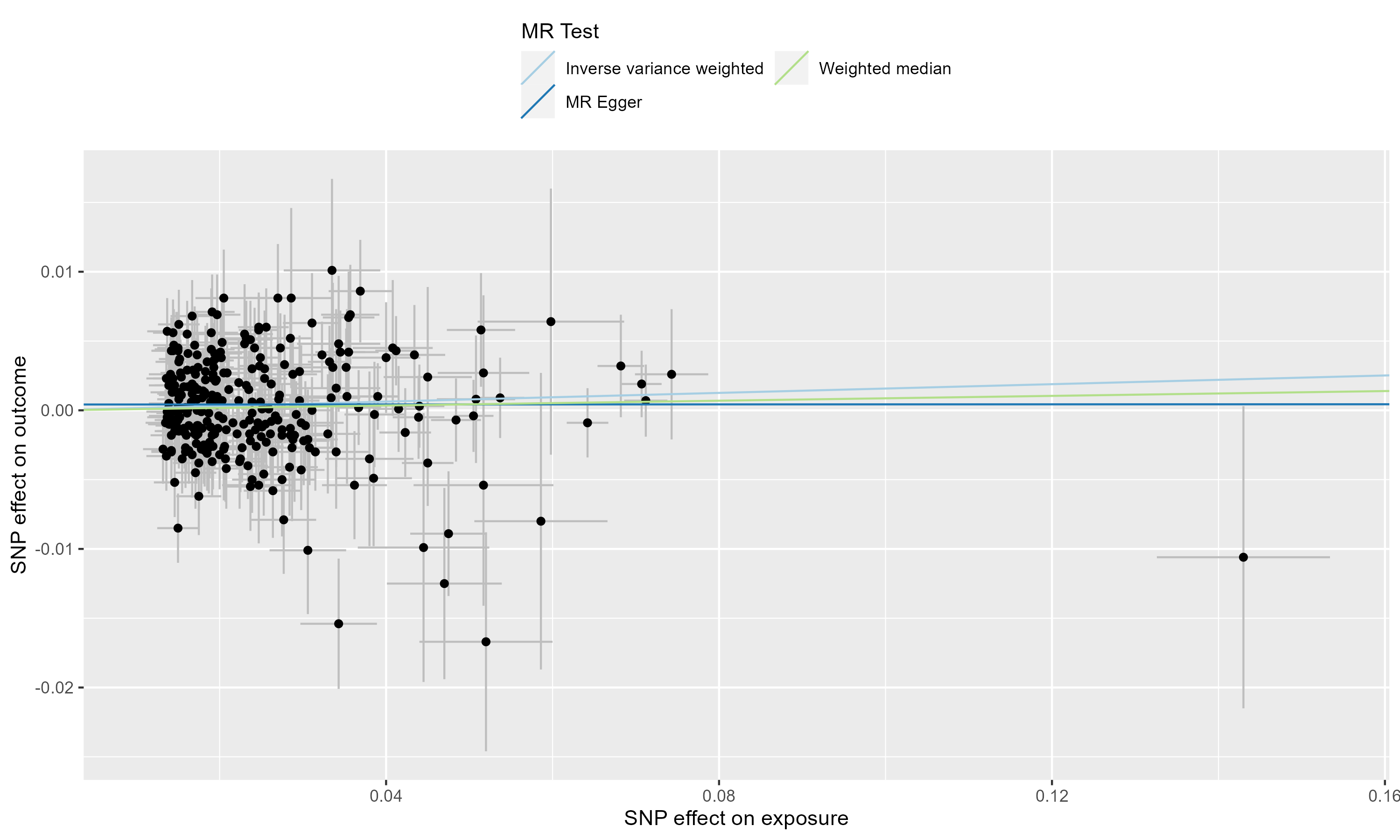

Supplement: Supplementary file 12 — Supplementary Material 12. [file 12890_2024_3150_MOESM12_ESM.zip › Supplementary Figure/scatter plot/Cortex Thickness/scatter_plotFEV1_FVC_entorhinal_thickavg.png]

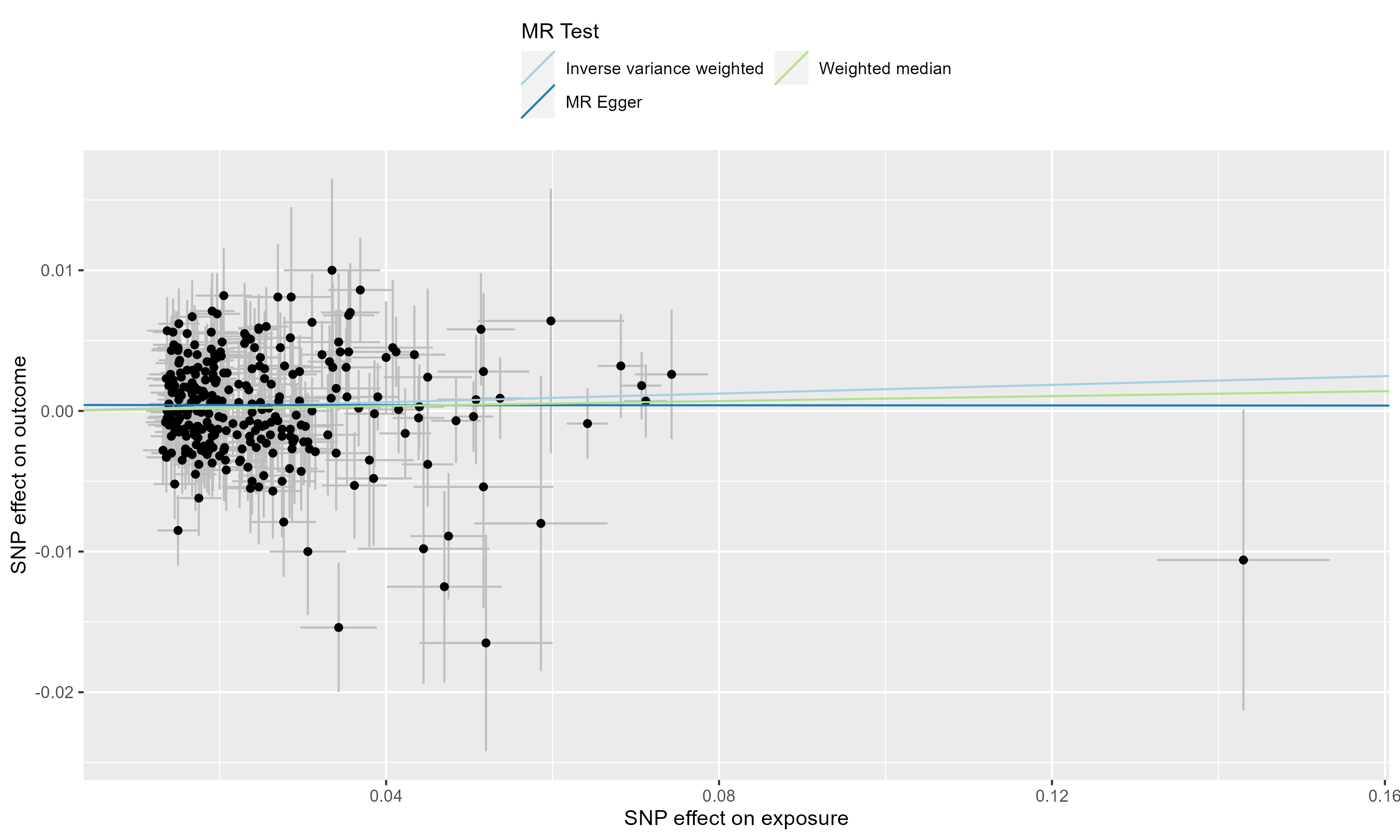

Supplement: Supplementary file 12 — Supplementary Material 12. [file 12890_2024_3150_MOESM12_ESM.zip › Supplementary Figure/scatter plot/Cortex Thickness/scatter_plotFEV1_FVC_entorhinal_thickavg_noGC.png]

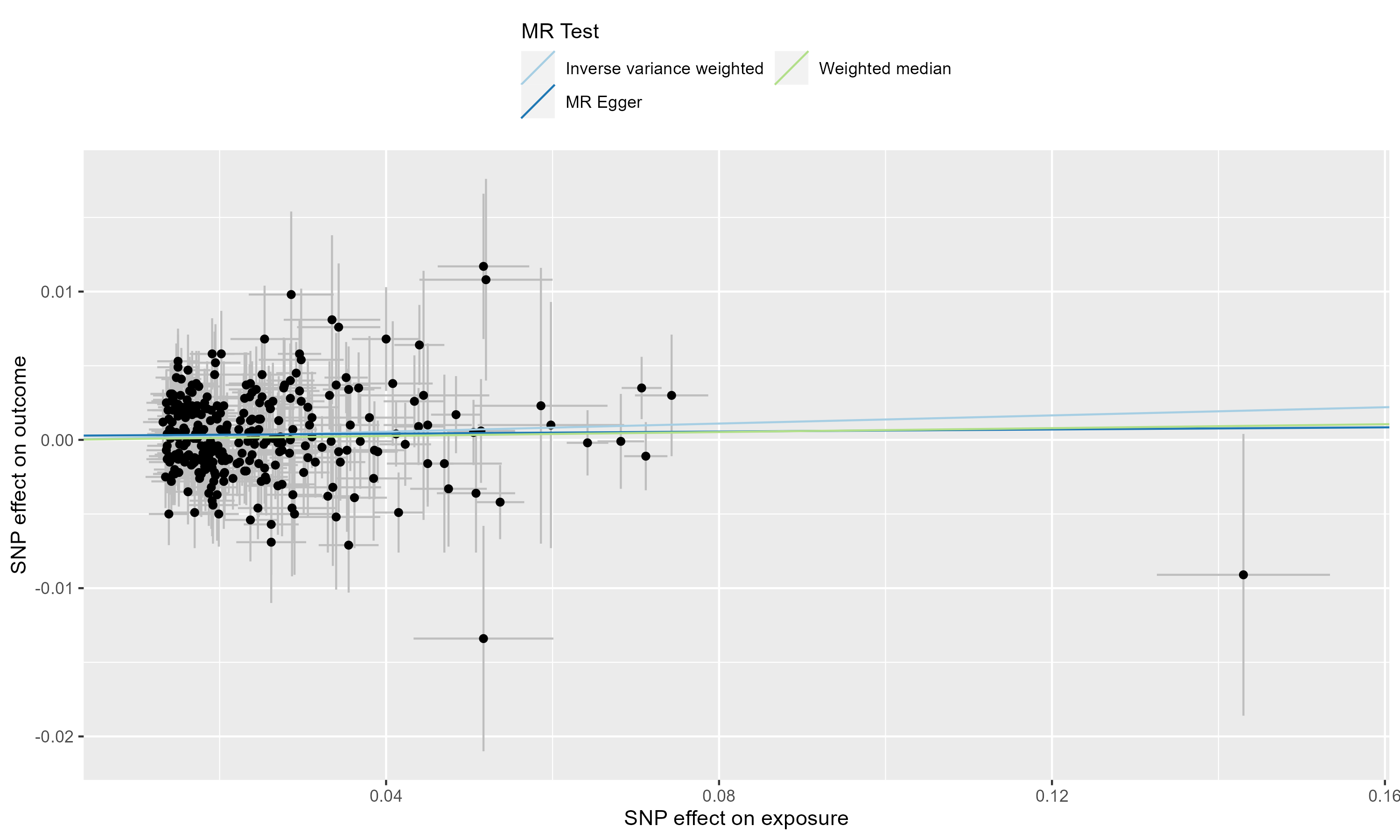

Supplement: Supplementary file 12 — Supplementary Material 12. [file 12890_2024_3150_MOESM12_ESM.zip › Supplementary Figure/scatter plot/Cortex Thickness/scatter_plotFEV1_FVC_temporalpole_thickavg.png]

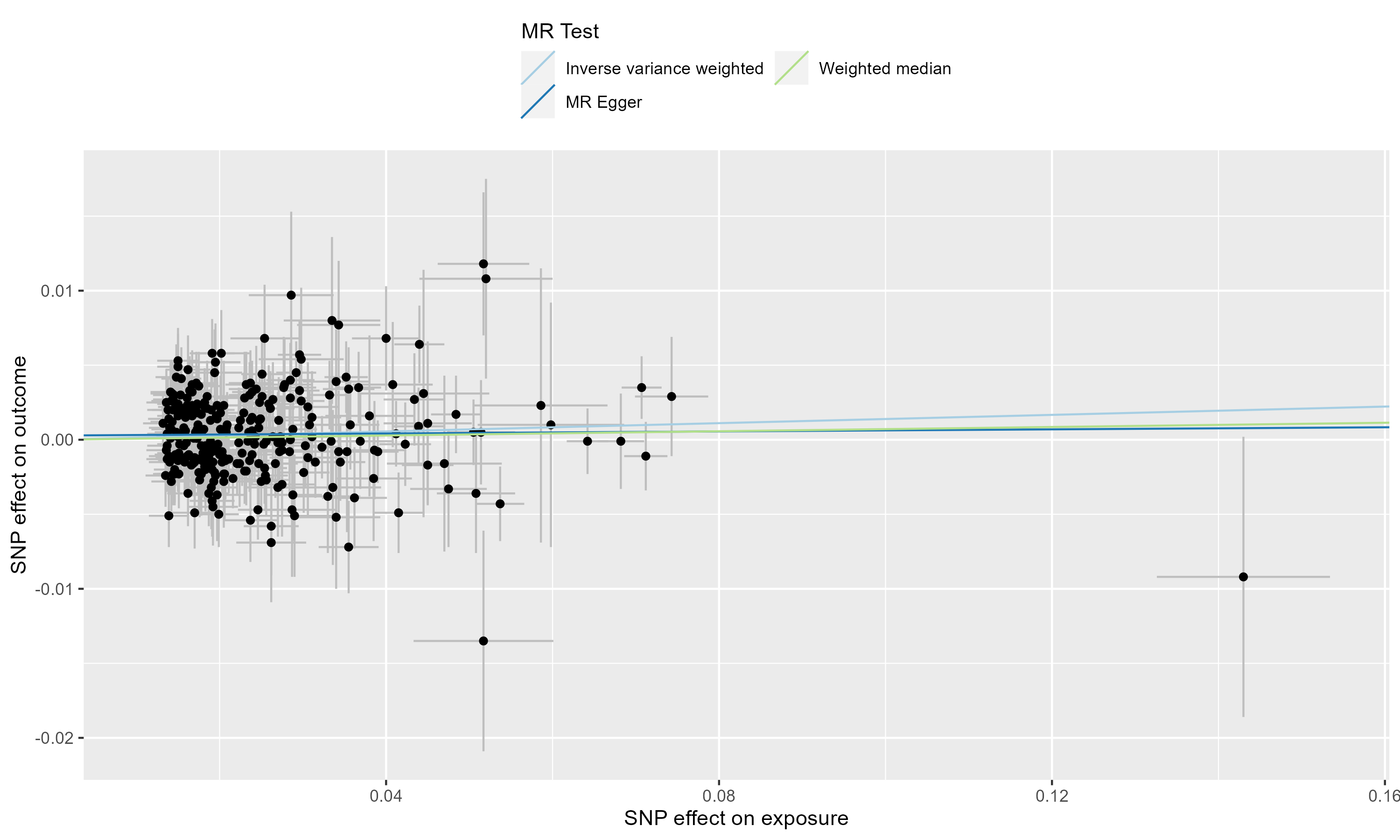

Supplement: Supplementary file 12 — Supplementary Material 12. [file 12890_2024_3150_MOESM12_ESM.zip › Supplementary Figure/scatter plot/Cortex Thickness/scatter_plotFEV1_FVC_temporalpole_thickavg_noGC.png]

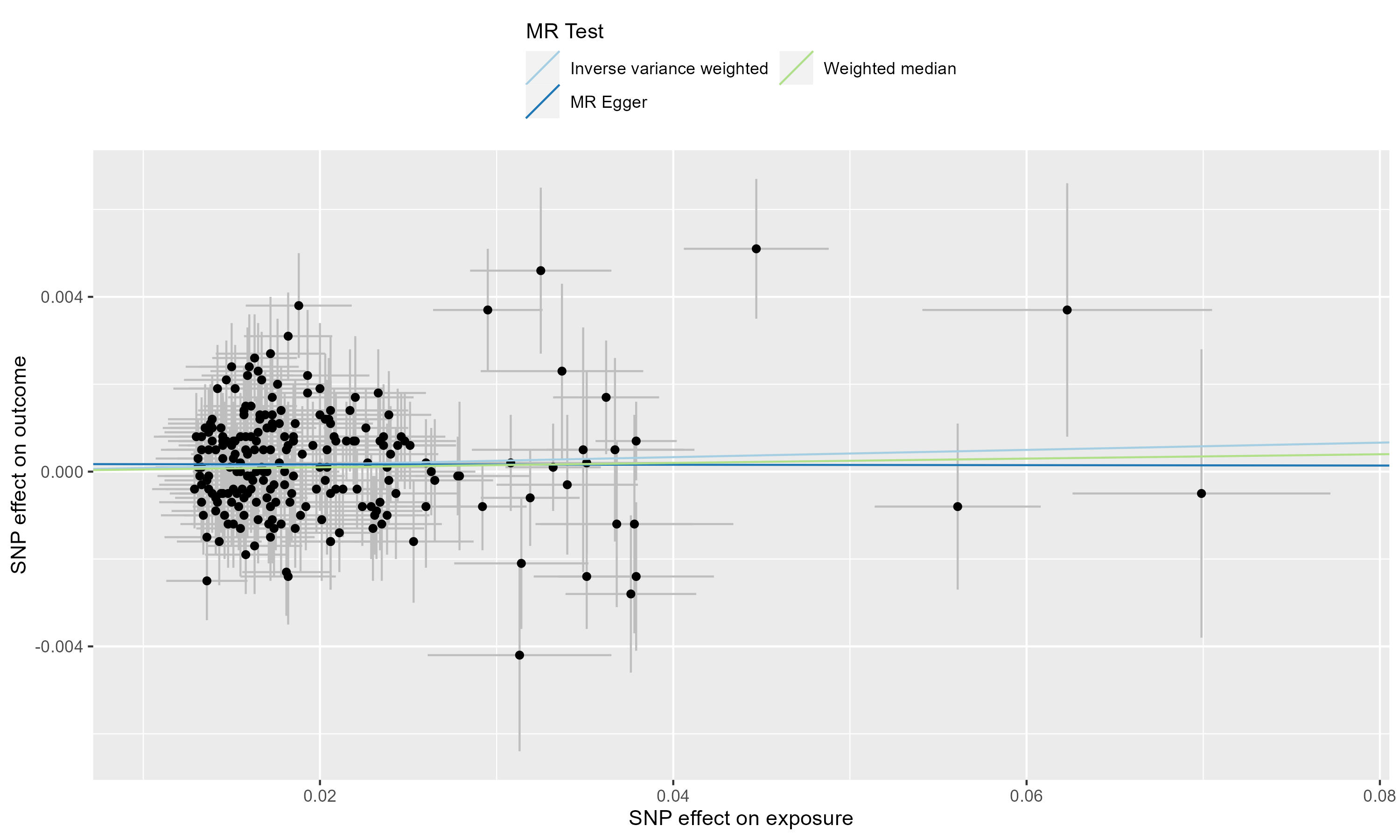

Supplement: Supplementary file 12 — Supplementary Material 12. [file 12890_2024_3150_MOESM12_ESM.zip › Supplementary Figure/scatter plot/Cortex Thickness/scatter_plotFVC_bankssts_thickavg.png]

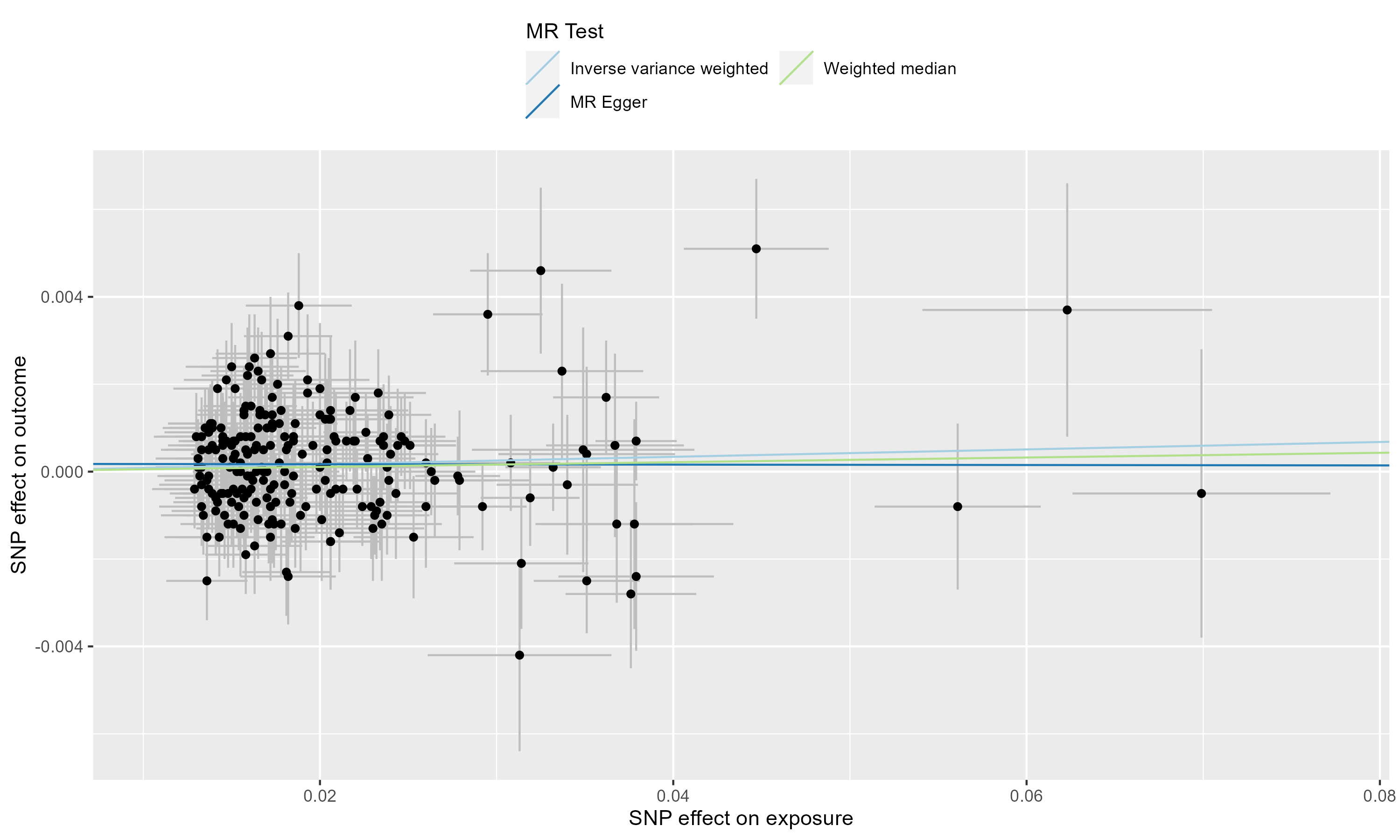

Supplement: Supplementary file 12 — Supplementary Material 12. [file 12890_2024_3150_MOESM12_ESM.zip › Supplementary Figure/scatter plot/Cortex Thickness/scatter_plotFVC_bankssts_thickavg_noGC.png]

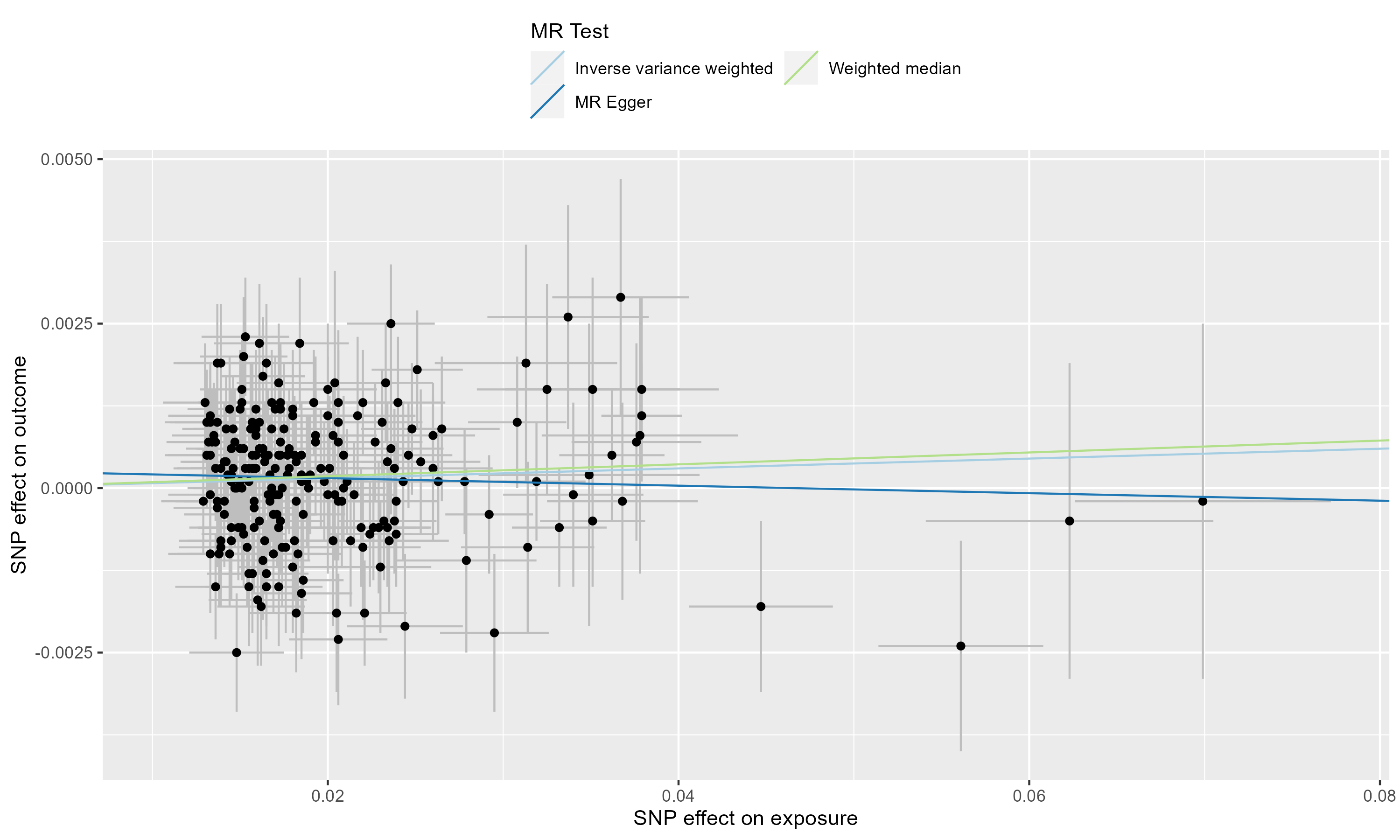

Supplement: Supplementary file 12 — Supplementary Material 12. [file 12890_2024_3150_MOESM12_ESM.zip › Supplementary Figure/scatter plot/Cortex Thickness/scatter_plotFVC_fusiform_thickavg.png]

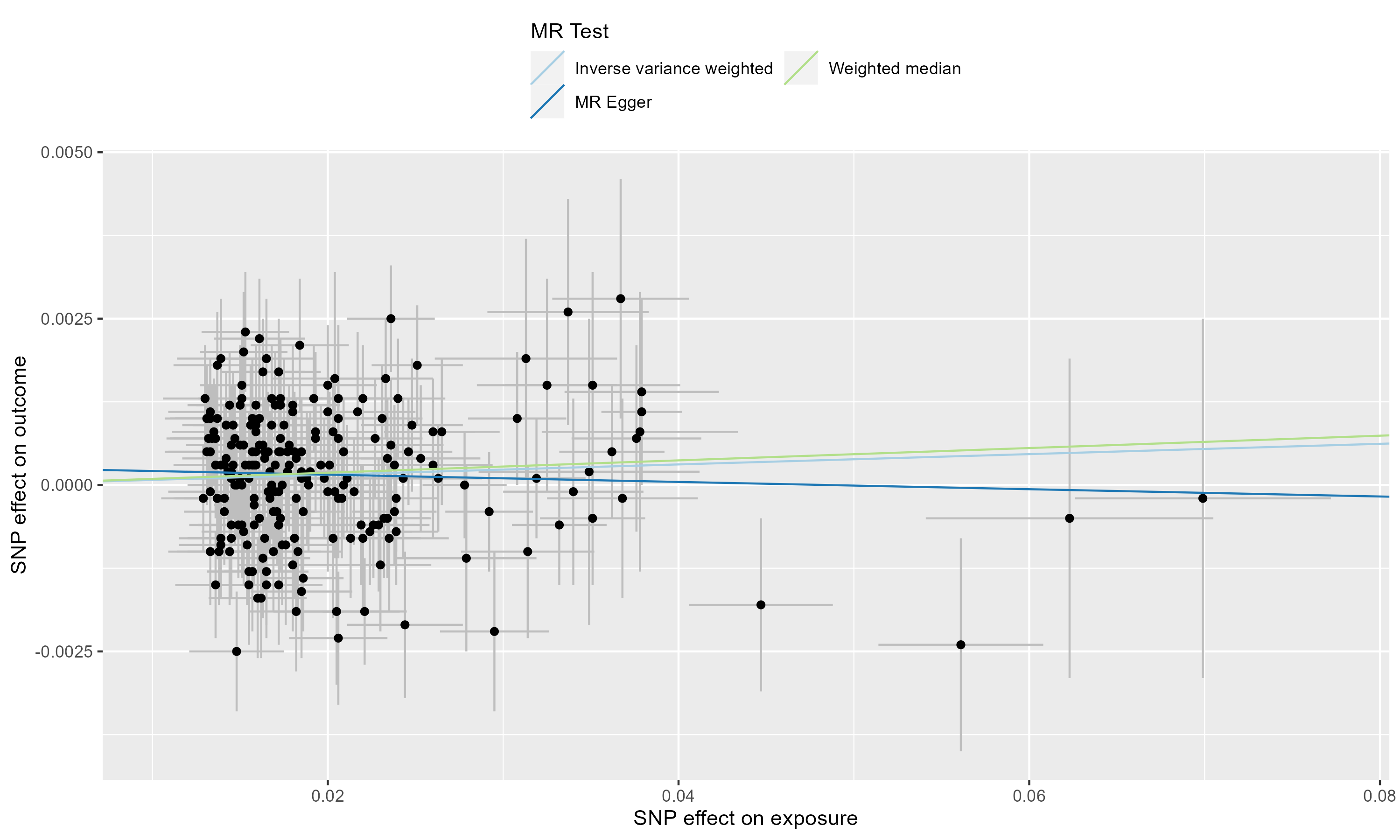

Supplement: Supplementary file 12 — Supplementary Material 12. [file 12890_2024_3150_MOESM12_ESM.zip › Supplementary Figure/scatter plot/Cortex Thickness/scatter_plotFVC_fusiform_thickavg_noGC.png]

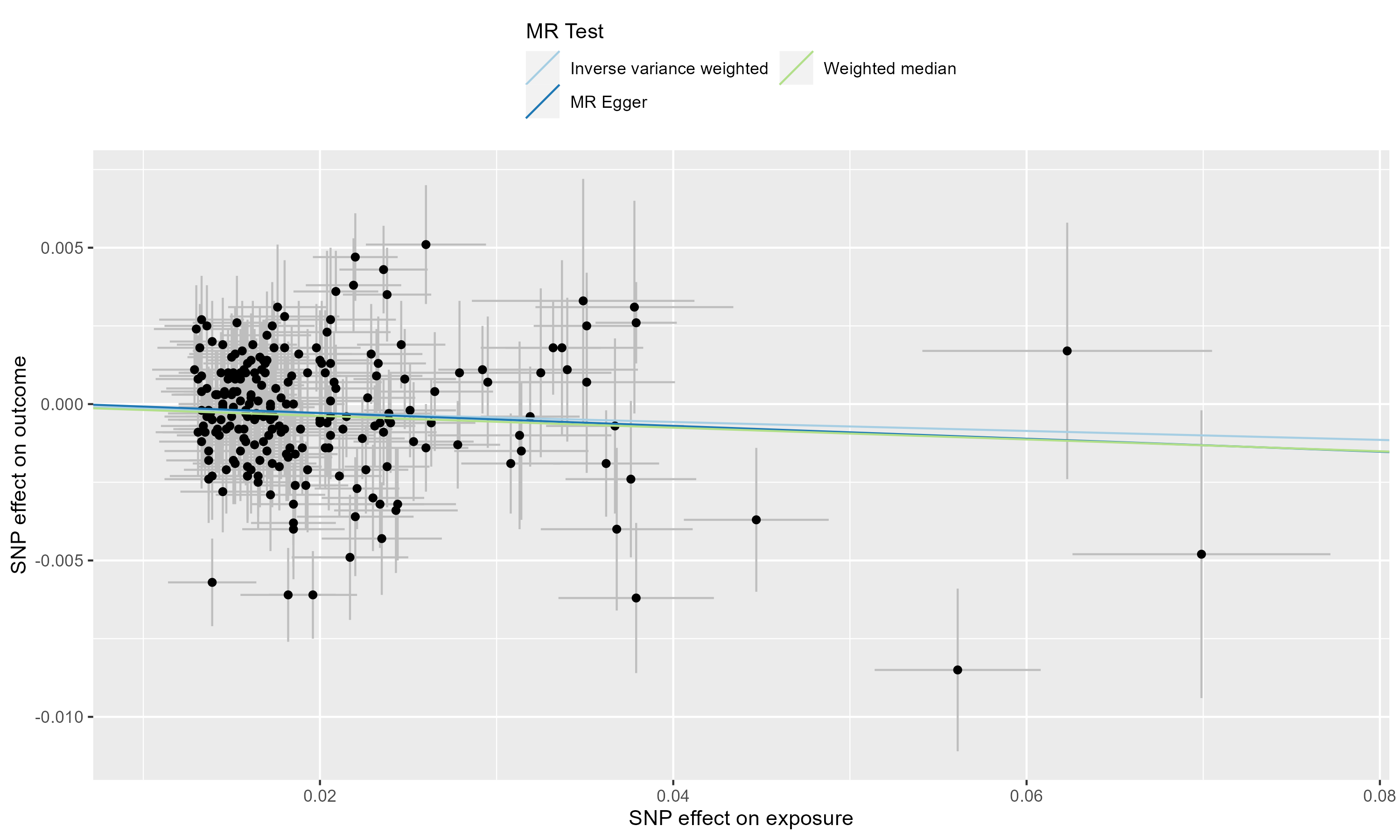

Supplement: Supplementary file 12 — Supplementary Material 12. [file 12890_2024_3150_MOESM12_ESM.zip › Supplementary Figure/scatter plot/Cortex Thickness/scatter_plotFVC_isthmuscingulate_thickavg.png]

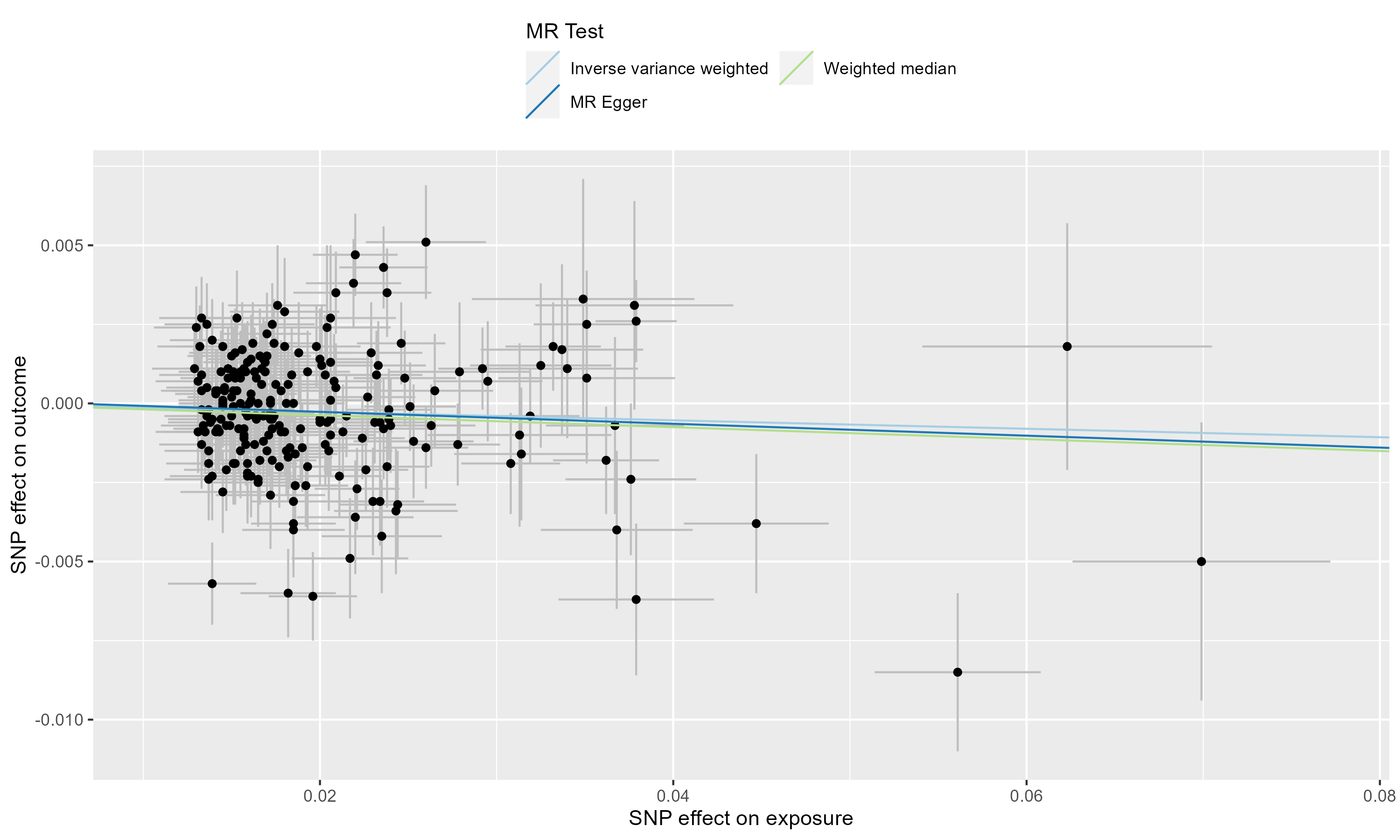

Supplement: Supplementary file 12 — Supplementary Material 12. [file 12890_2024_3150_MOESM12_ESM.zip › Supplementary Figure/scatter plot/Cortex Thickness/scatter_plotFVC_isthmuscingulate_thickavg_noGC.png]

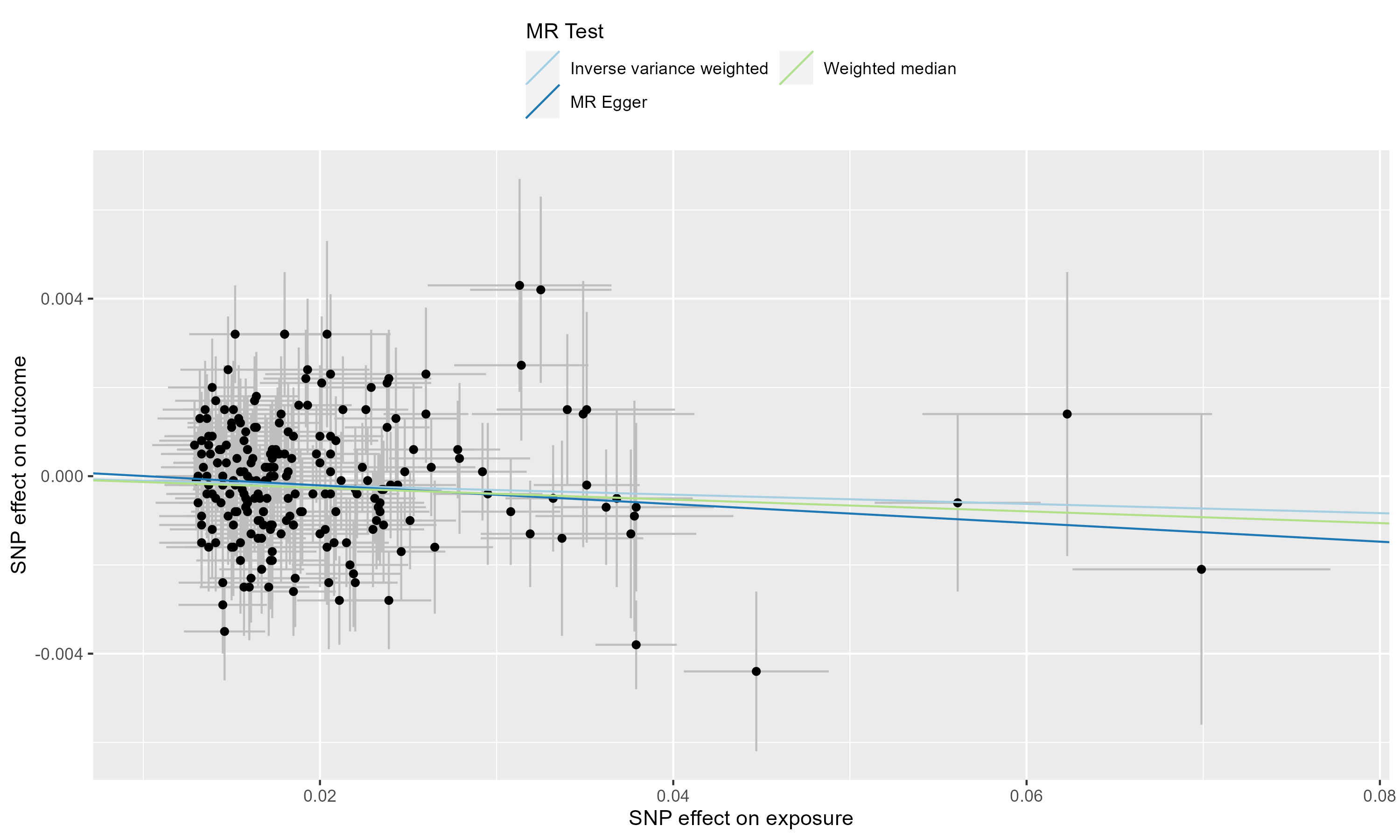

Supplement: Supplementary file 12 — Supplementary Material 12. [file 12890_2024_3150_MOESM12_ESM.zip › Supplementary Figure/scatter plot/Cortex Thickness/scatter_plotFVC_medialorbitofrontal_thickavg.png]

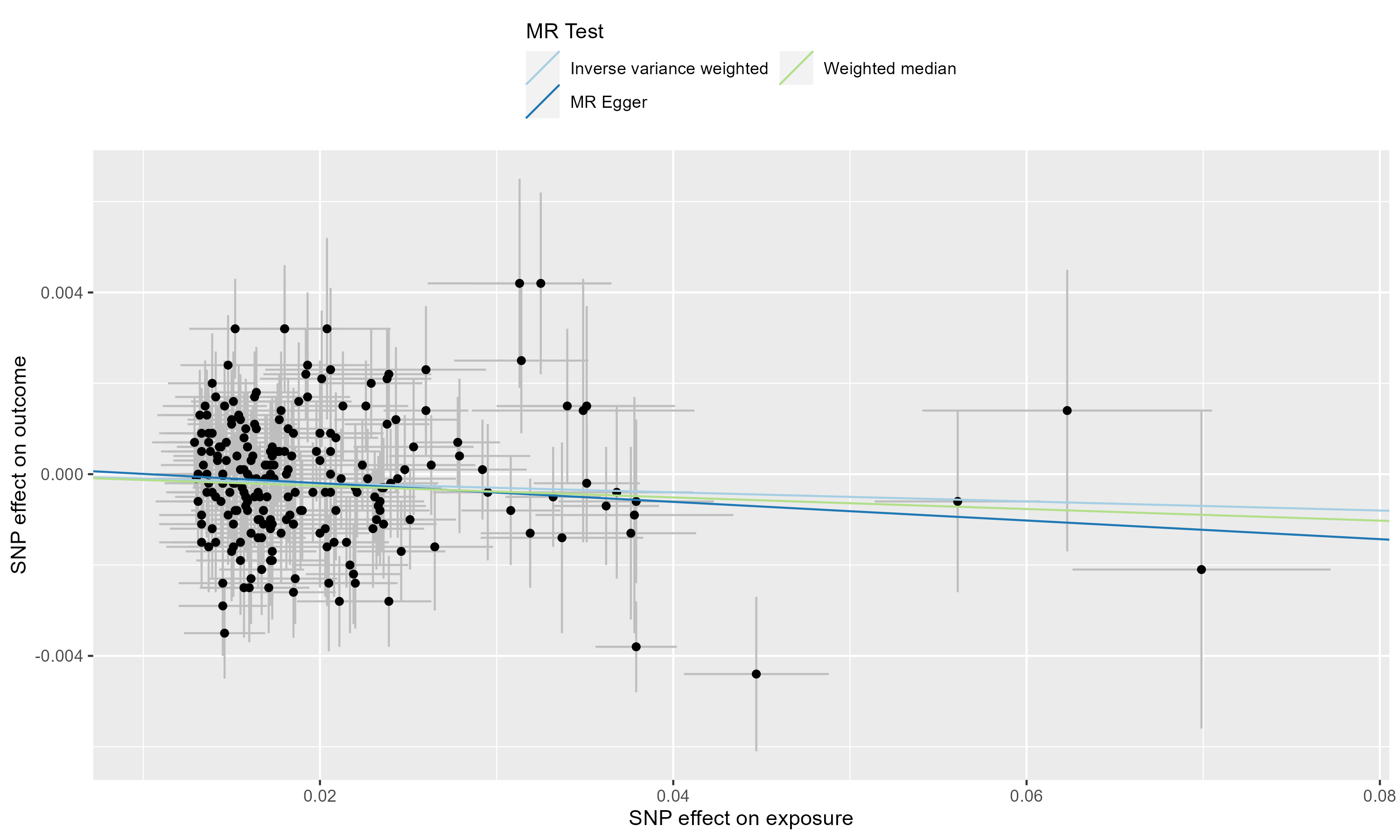

Supplement: Supplementary file 12 — Supplementary Material 12. [file 12890_2024_3150_MOESM12_ESM.zip › Supplementary Figure/scatter plot/Cortex Thickness/scatter_plotFVC_medialorbitofrontal_thickavg_noGC.png]

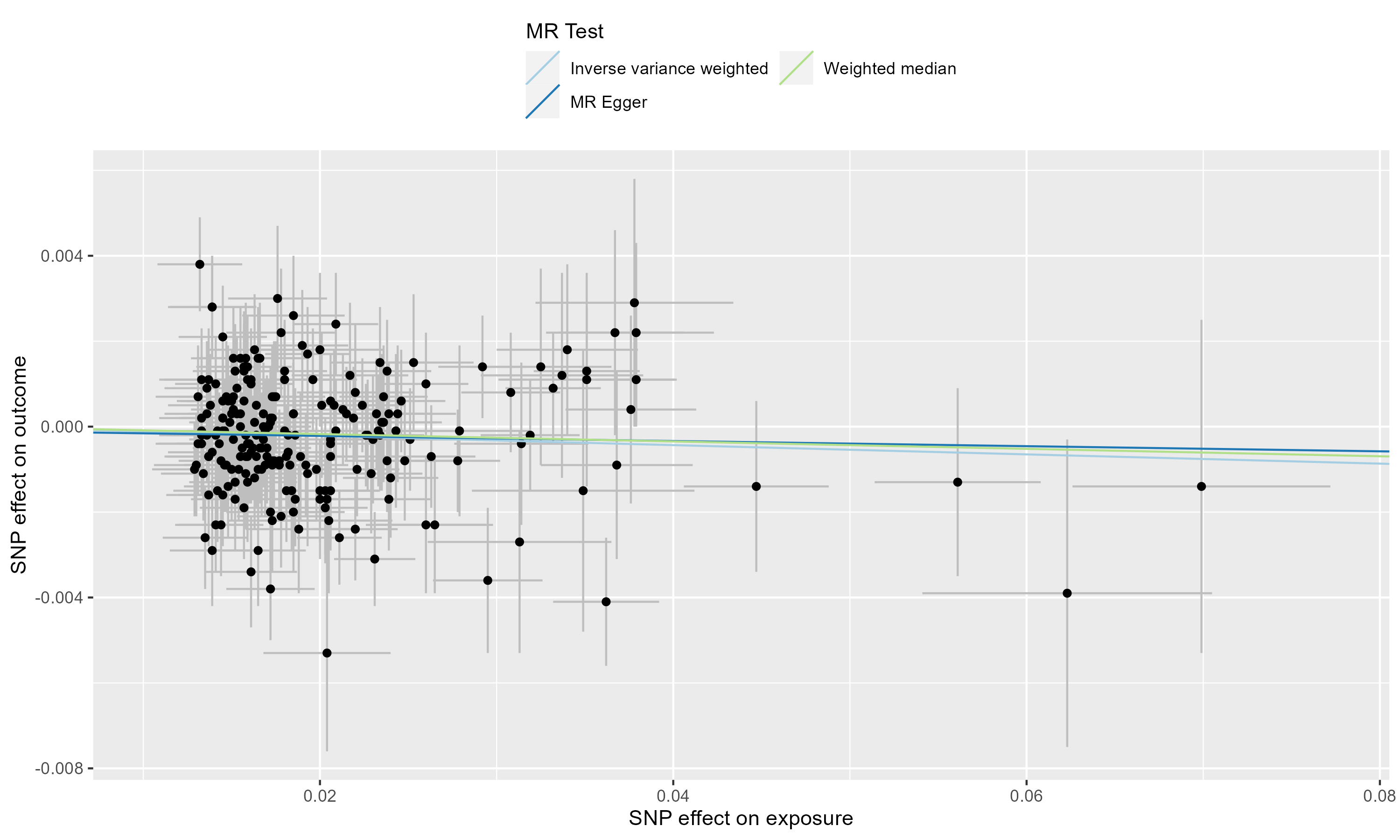

Supplement: Supplementary file 12 — Supplementary Material 12. [file 12890_2024_3150_MOESM12_ESM.zip › Supplementary Figure/scatter plot/Cortex Thickness/scatter_plotFVC_parsorbitalis_thickavg.png]

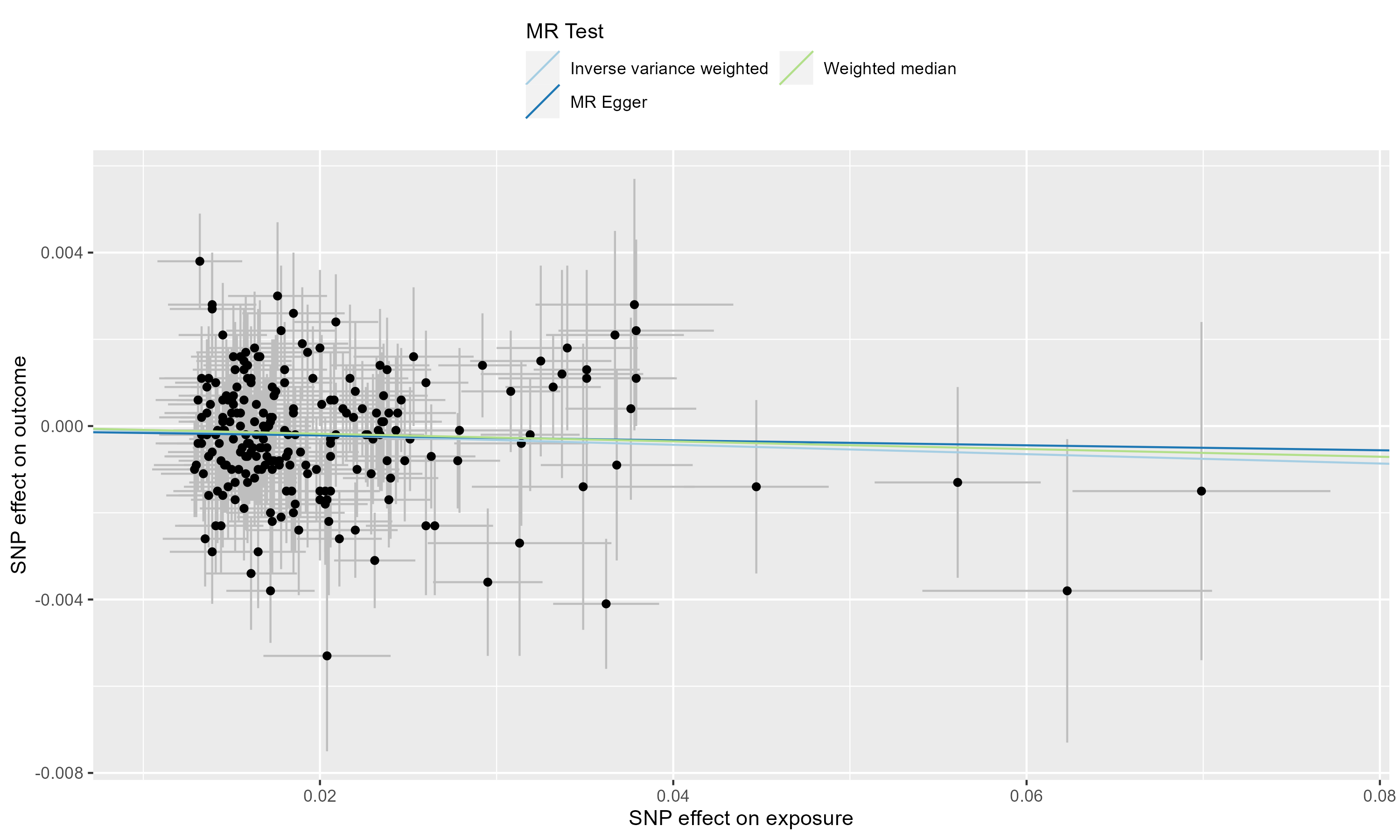

Supplement: Supplementary file 12 — Supplementary Material 12. [file 12890_2024_3150_MOESM12_ESM.zip › Supplementary Figure/scatter plot/Cortex Thickness/scatter_plotFVC_parsorbitalis_thickavg_noGC.png]
