# Supplementary figures and images for: COPD, PRISm and lung function reduction affect the brain cortical structure: a Mendelian randomization study (part 1 of 2)
Source: BMC Pulm Med. 2024 Jul 15;24:341. doi: 10.1186/s12890-024-03150-2 (PMC11251327; doi:10.1186/s12890-024-03150-2)

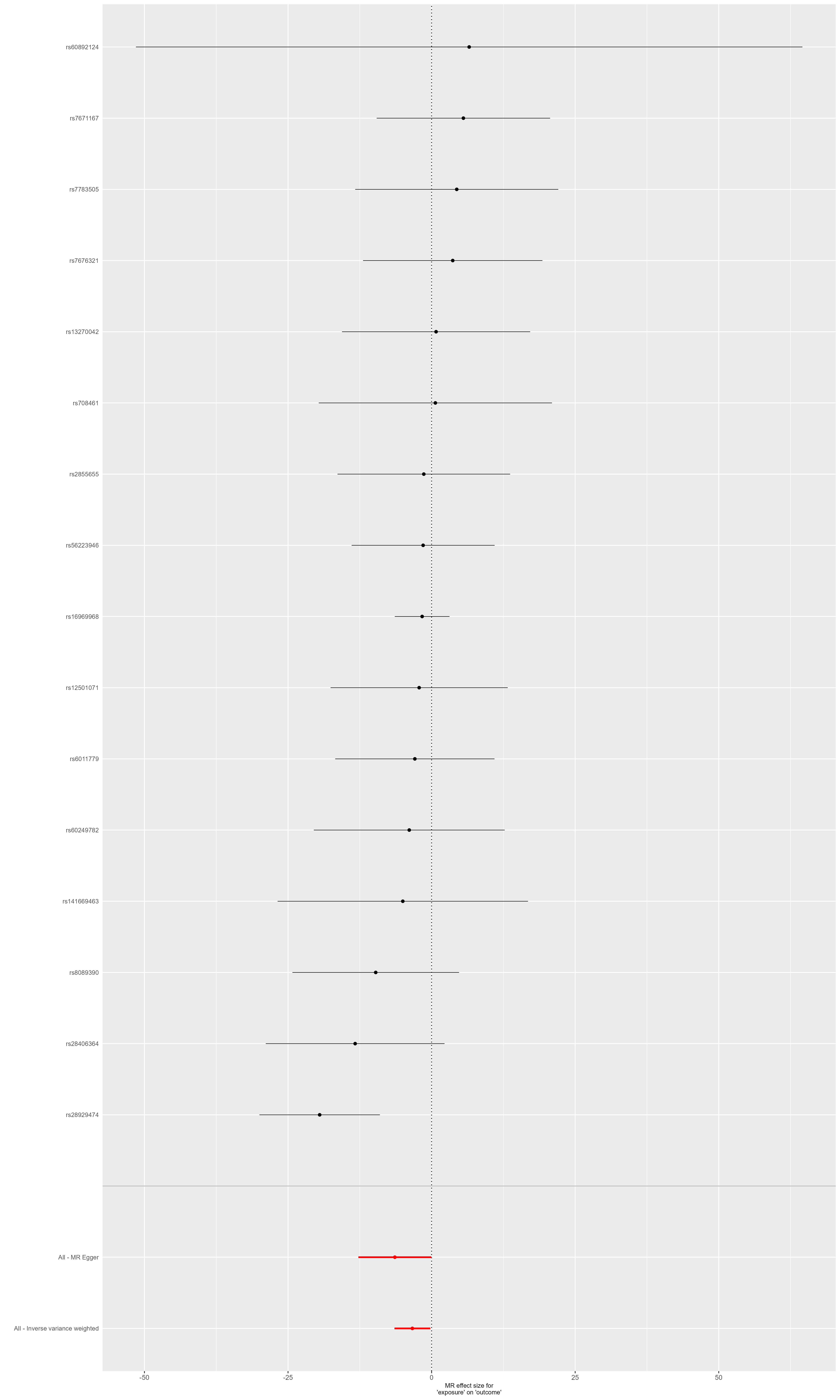

Supplement: Supplementary file 12 — Supplementary Material 12. [file 12890_2024_3150_MOESM12_ESM.zip › Supplementary Figure/Forest plot/Cortex Surface area/forest_plotCOPD_parsorbitalis_surfavg.png]

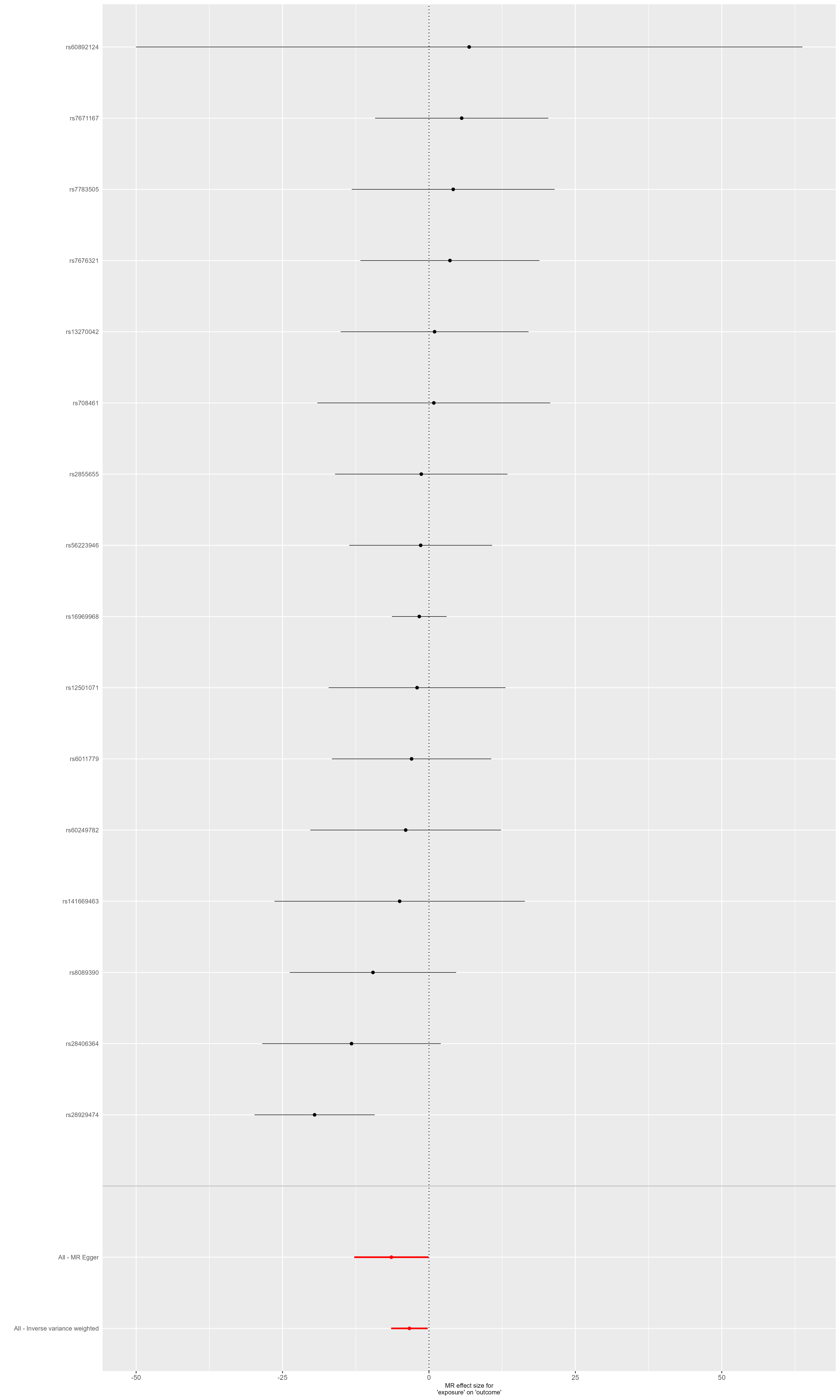

Supplement: Supplementary file 12 — Supplementary Material 12. [file 12890_2024_3150_MOESM12_ESM.zip › Supplementary Figure/Forest plot/Cortex Surface area/forest_plotCOPD_parsorbitalis_surfavg_noGC.png]

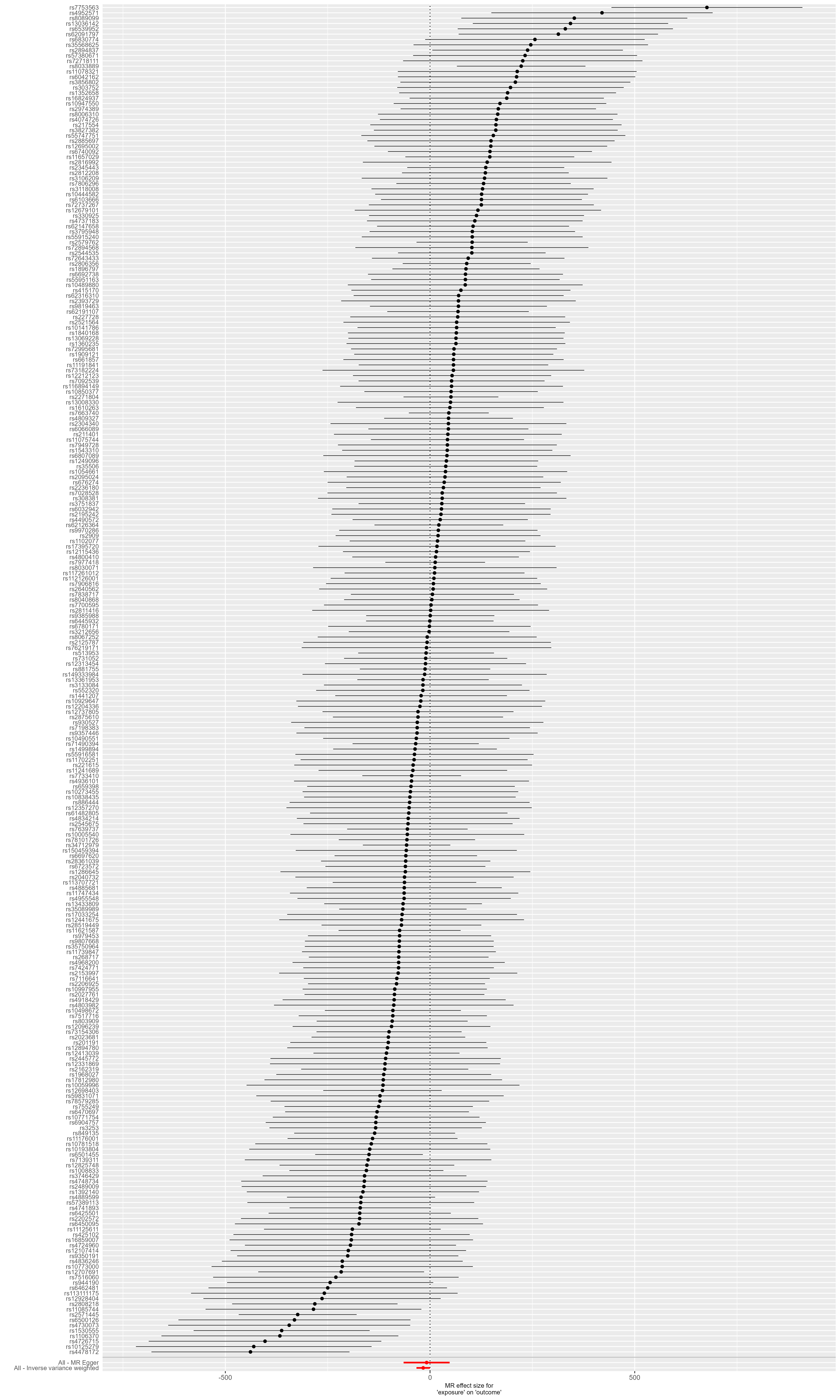

Supplement: Supplementary file 12 — Supplementary Material 12. [file 12890_2024_3150_MOESM12_ESM.zip › Supplementary Figure/Forest plot/Cortex Surface area/forest_plotFEV1_caudalmiddlefrontal_surfavg.png]

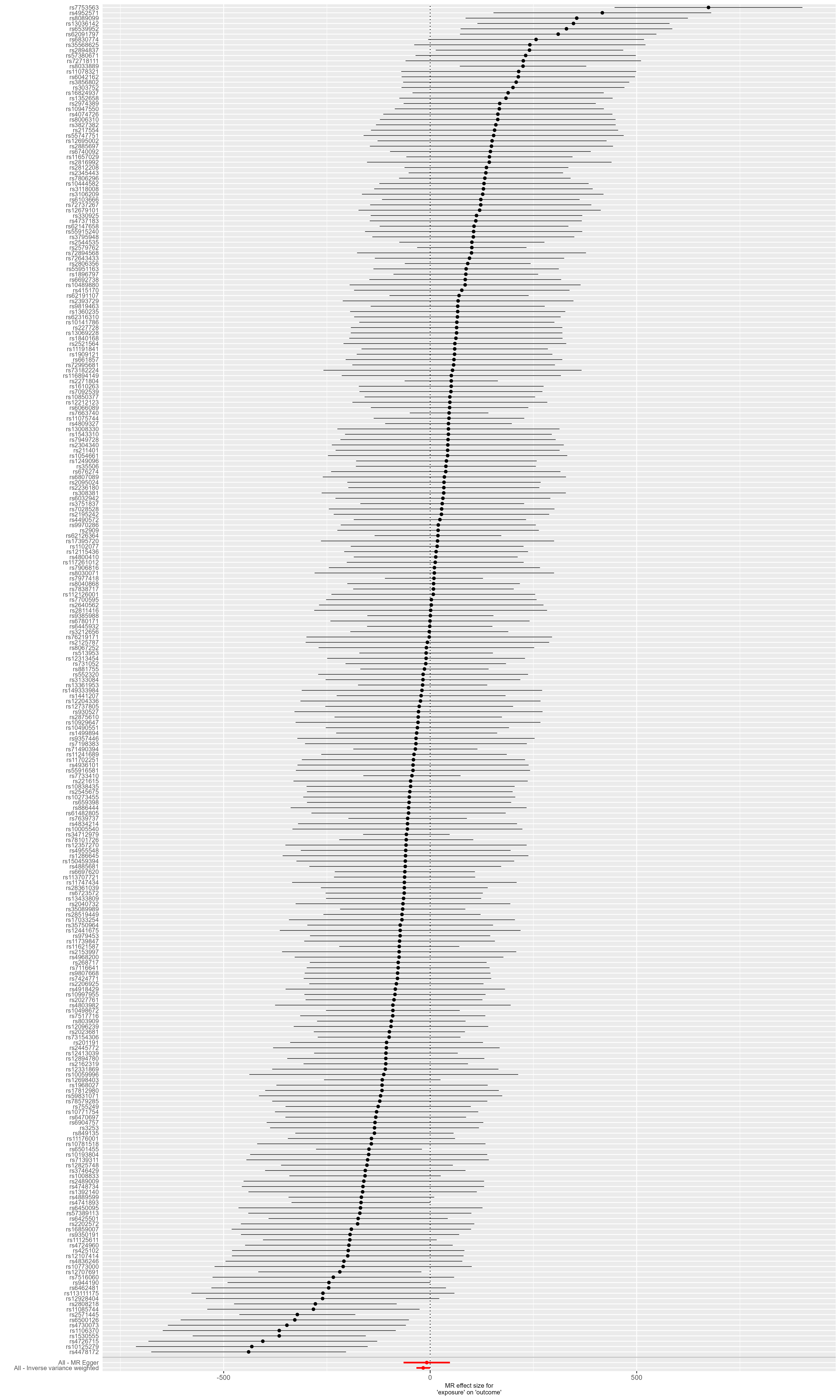

Supplement: Supplementary file 12 — Supplementary Material 12. [file 12890_2024_3150_MOESM12_ESM.zip › Supplementary Figure/Forest plot/Cortex Surface area/forest_plotFEV1_caudalmiddlefrontal_surfavg_noGC.png]

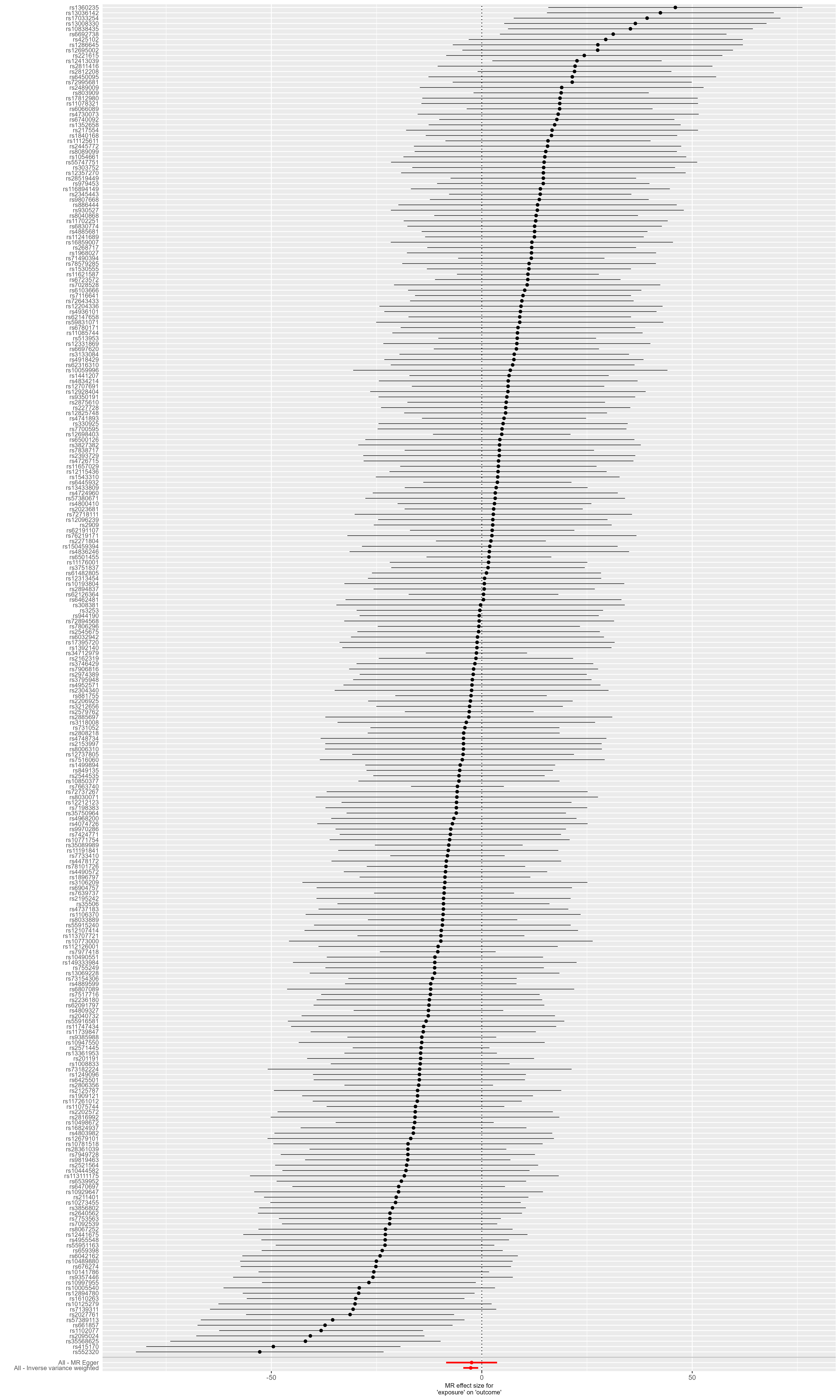

Supplement: Supplementary file 12 — Supplementary Material 12. [file 12890_2024_3150_MOESM12_ESM.zip › Supplementary Figure/Forest plot/Cortex Surface area/forest_plotFEV1_frontalpole_surfavg.png]

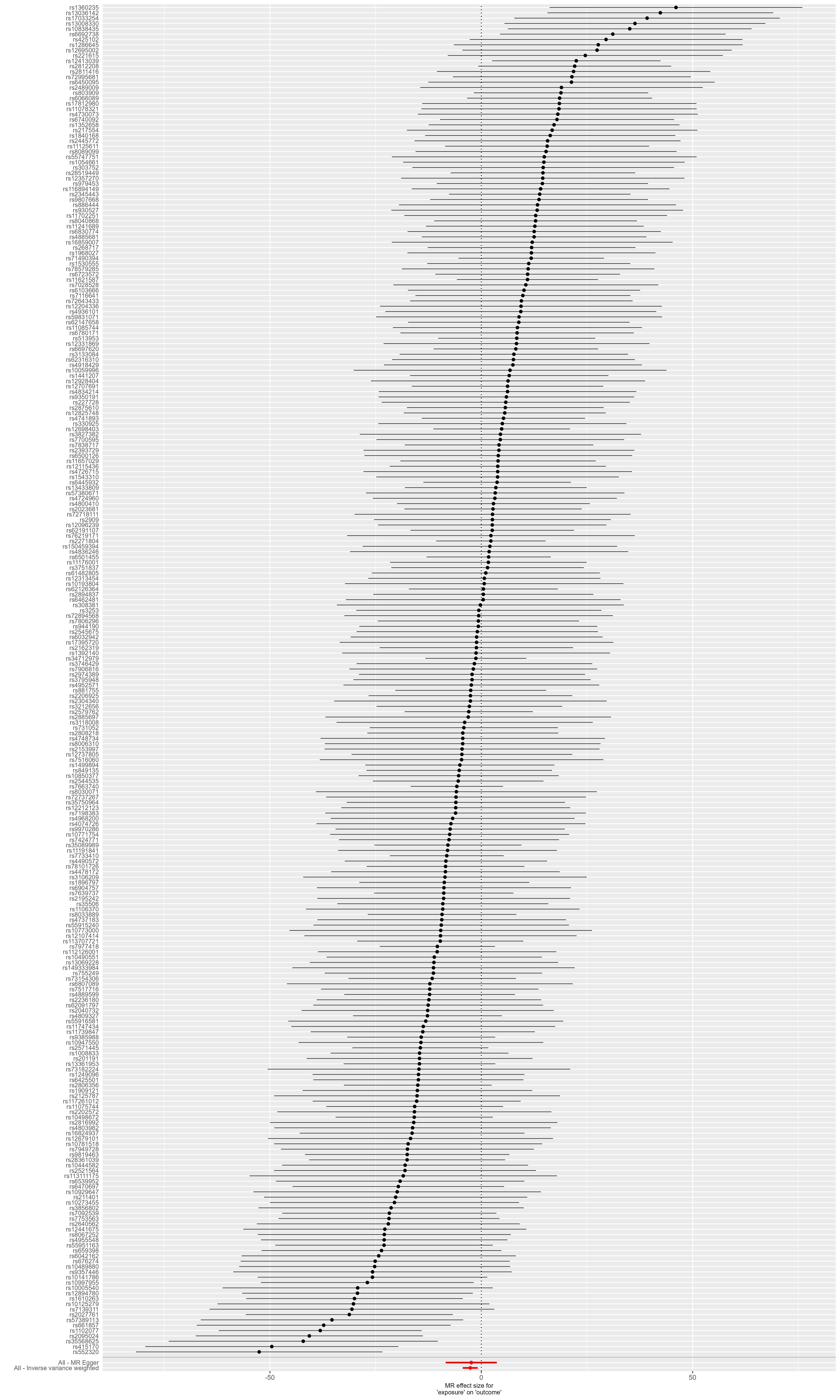

Supplement: Supplementary file 12 — Supplementary Material 12. [file 12890_2024_3150_MOESM12_ESM.zip › Supplementary Figure/Forest plot/Cortex Surface area/forest_plotFEV1_frontalpole_surfavg_noGC.png]

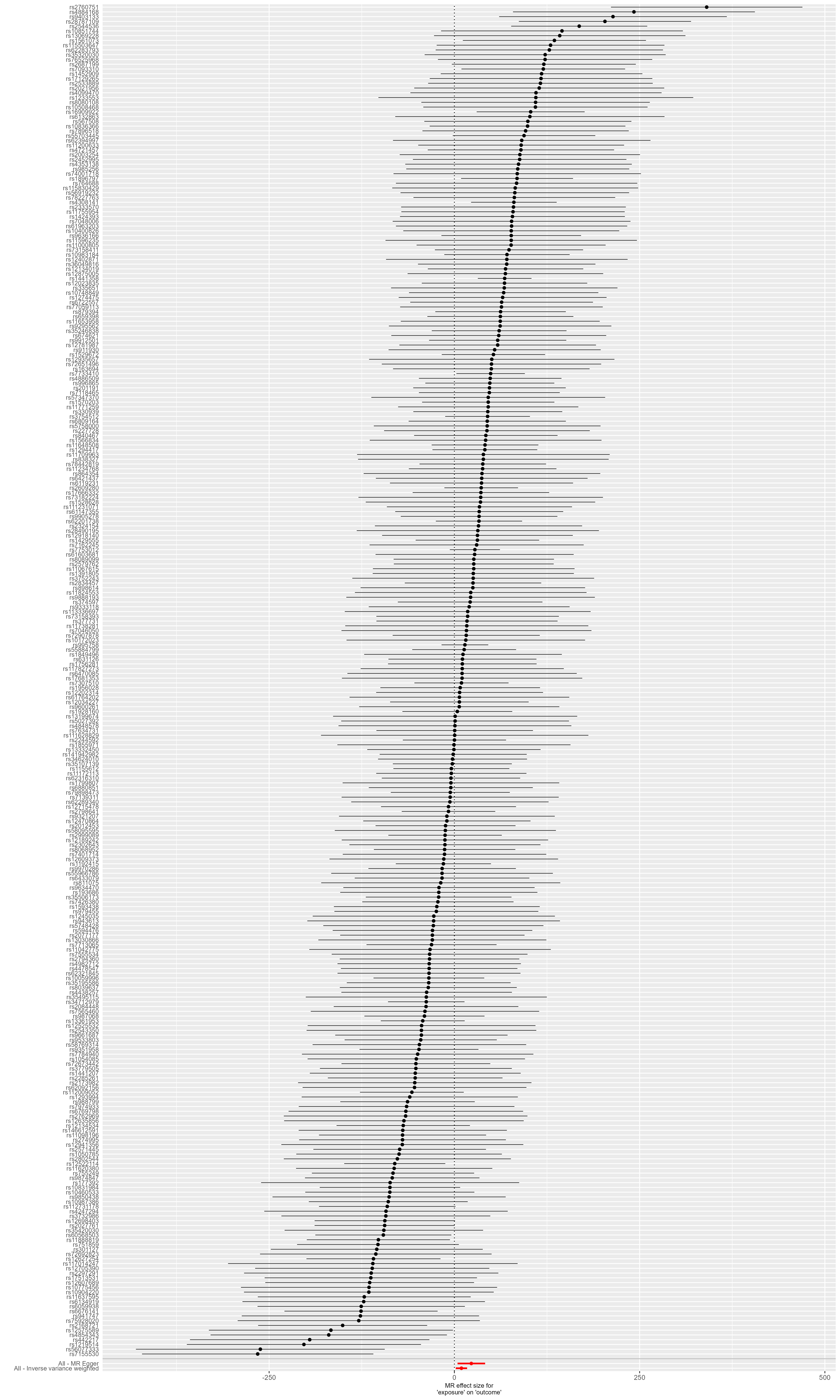

Supplement: Supplementary file 12 — Supplementary Material 12. [file 12890_2024_3150_MOESM12_ESM.zip › Supplementary Figure/Forest plot/Cortex Surface area/forest_plotFEV1_FVC_paracentral_surfavg.png]

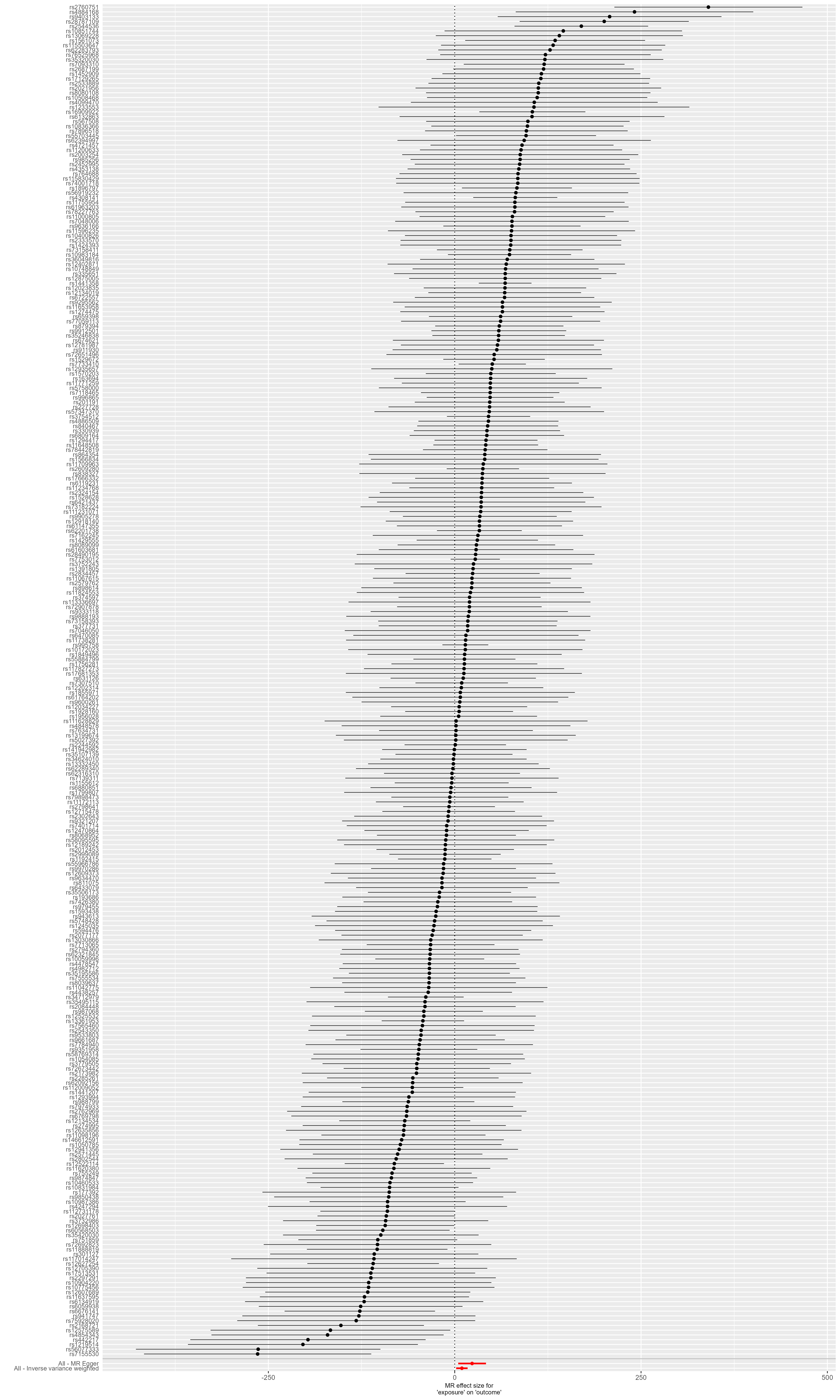

Supplement: Supplementary file 12 — Supplementary Material 12. [file 12890_2024_3150_MOESM12_ESM.zip › Supplementary Figure/Forest plot/Cortex Surface area/forest_plotFEV1_FVC_paracentral_surfavg_noGC.png]

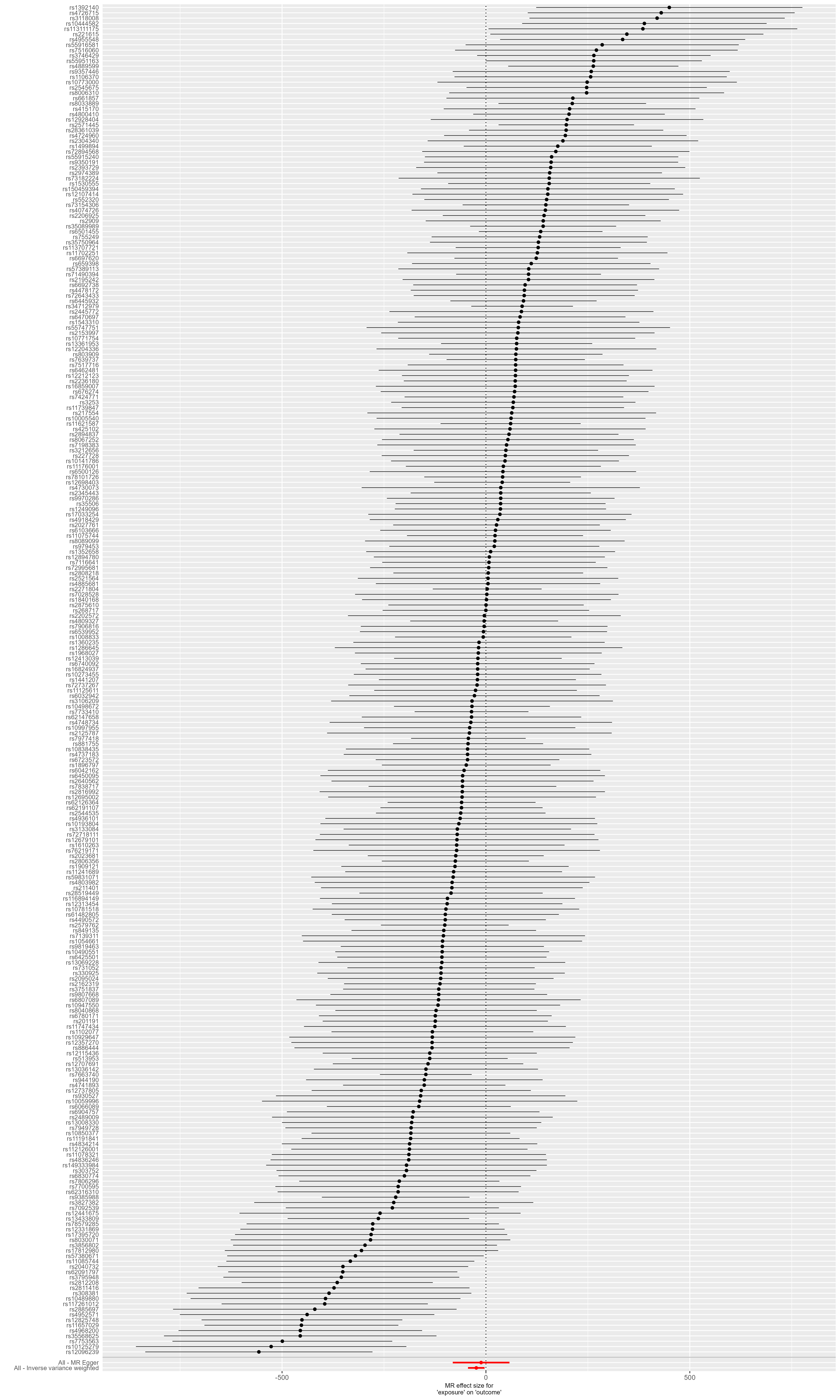

Supplement: Supplementary file 12 — Supplementary Material 12. [file 12890_2024_3150_MOESM12_ESM.zip › Supplementary Figure/Forest plot/Cortex Surface area/forest_plotFEV1_lingual_surfavg.png]

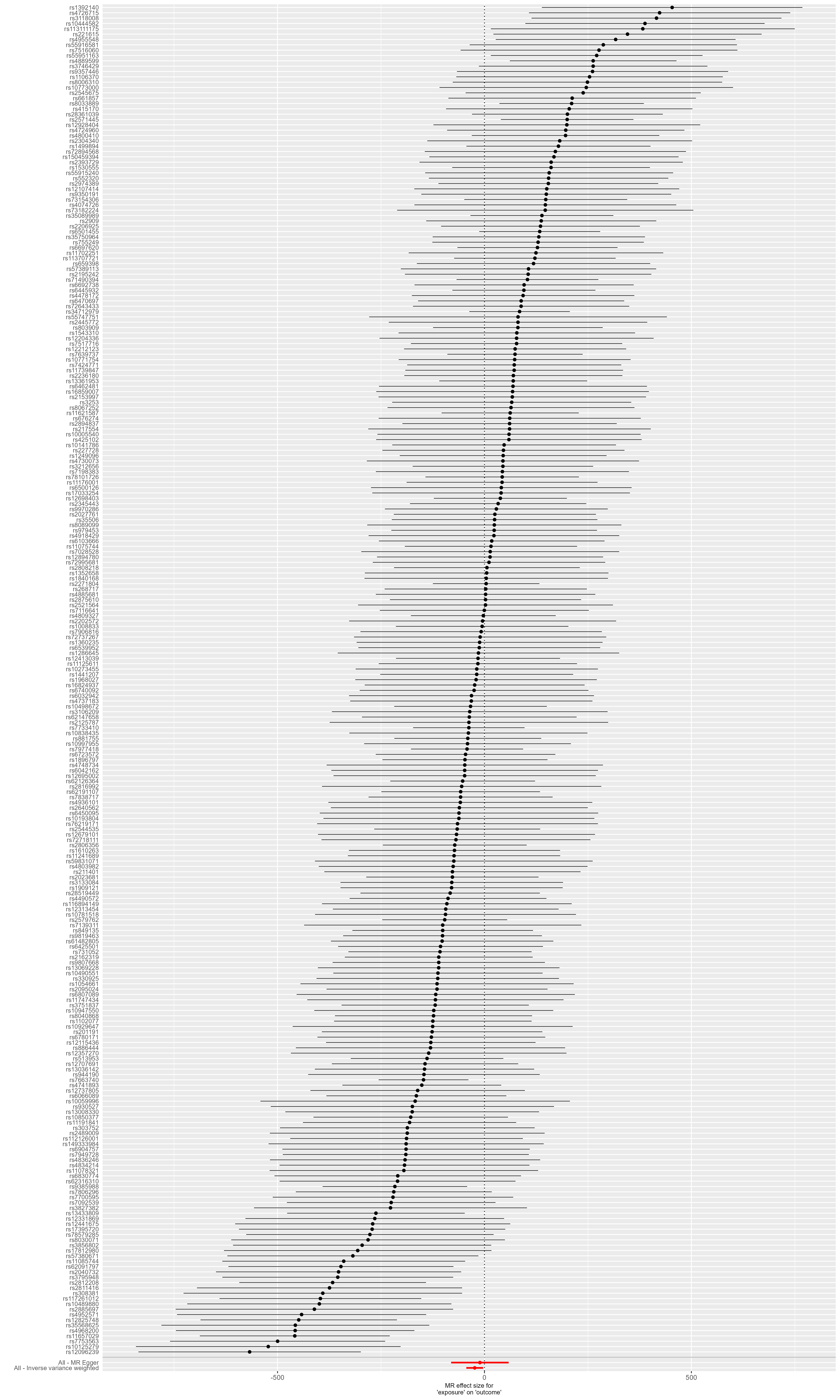

Supplement: Supplementary file 12 — Supplementary Material 12. [file 12890_2024_3150_MOESM12_ESM.zip › Supplementary Figure/Forest plot/Cortex Surface area/forest_plotFEV1_lingual_surfavg_noGC.png]

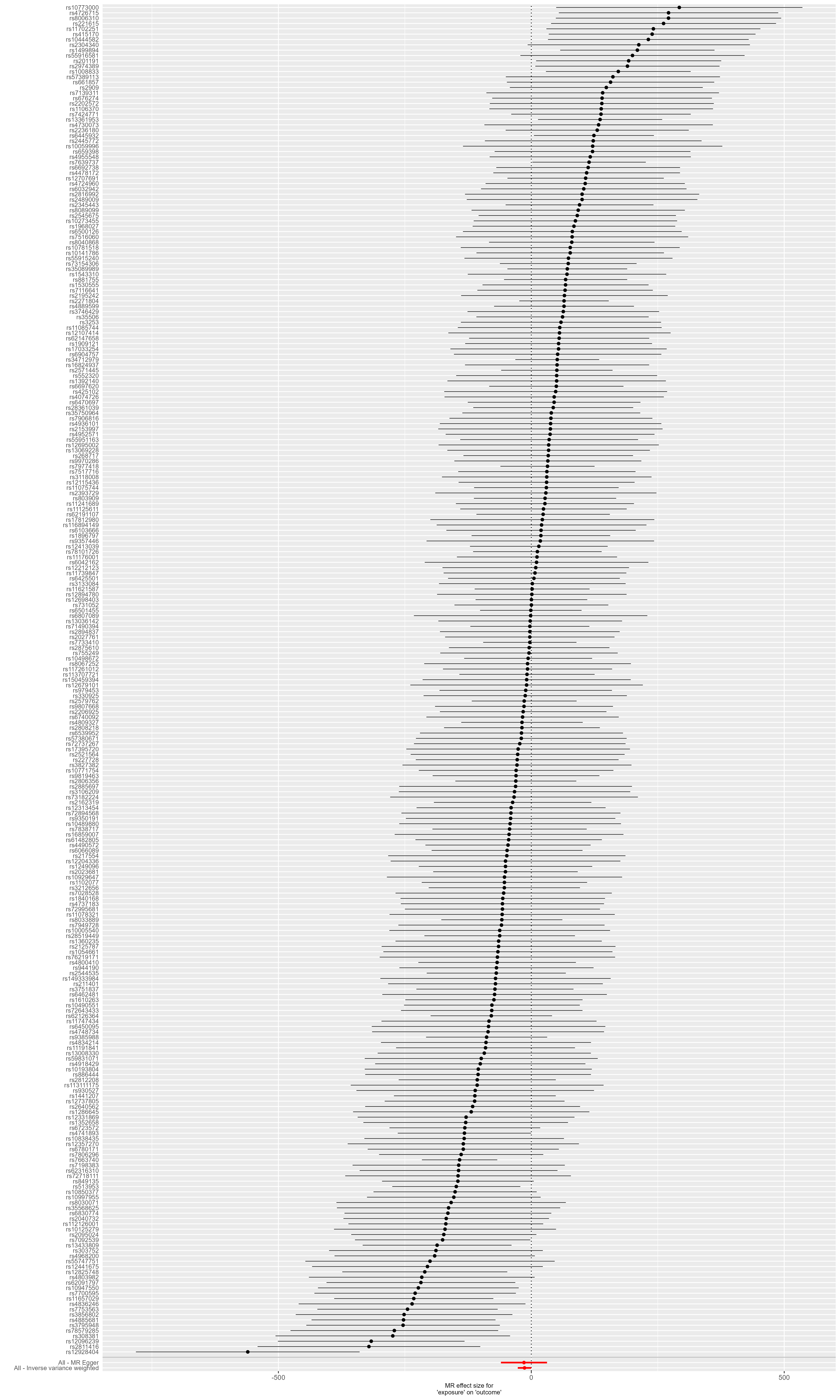

Supplement: Supplementary file 12 — Supplementary Material 12. [file 12890_2024_3150_MOESM12_ESM.zip › Supplementary Figure/Forest plot/Cortex Surface area/forest_plotFEV1_pericalcarine_surfavg.png]

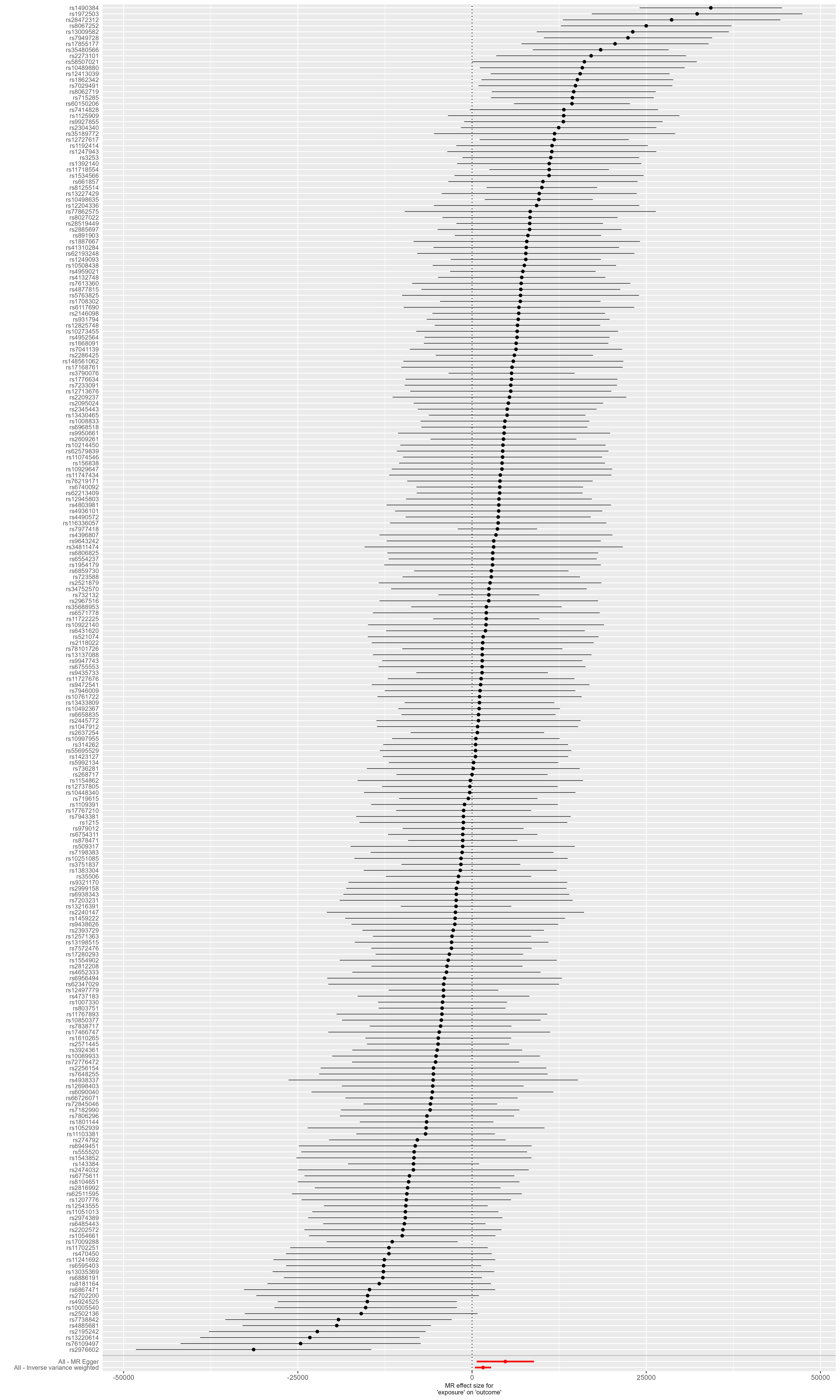

Supplement: Supplementary file 12 — Supplementary Material 12. [file 12890_2024_3150_MOESM12_ESM.zip › Supplementary Figure/Forest plot/Cortex Surface area/forest_plotFVC_Full_SurfArea.png]

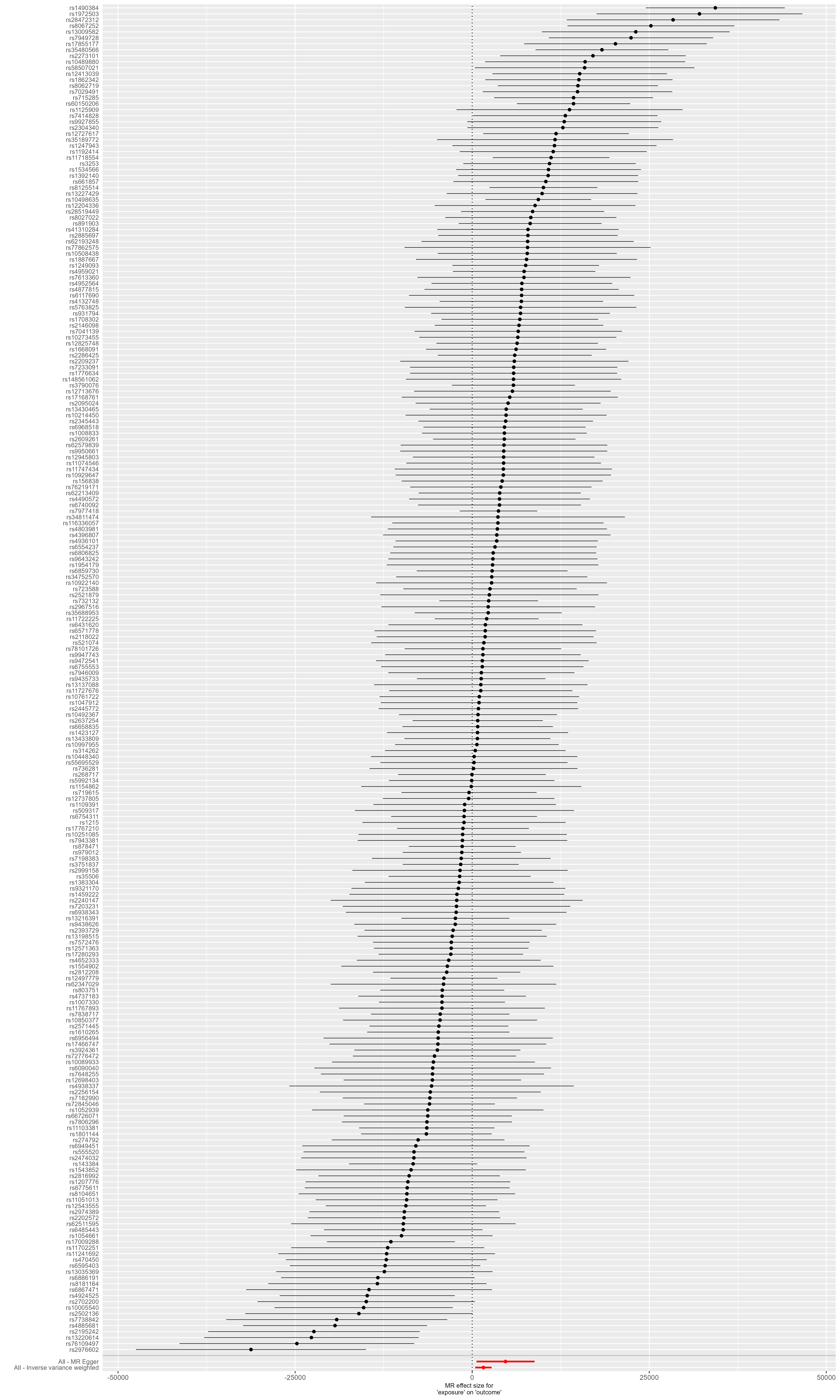

Supplement: Supplementary file 12 — Supplementary Material 12. [file 12890_2024_3150_MOESM12_ESM.zip › Supplementary Figure/Forest plot/Cortex Surface area/forest_plotFVC_Full_SurfArea_noGC.png]

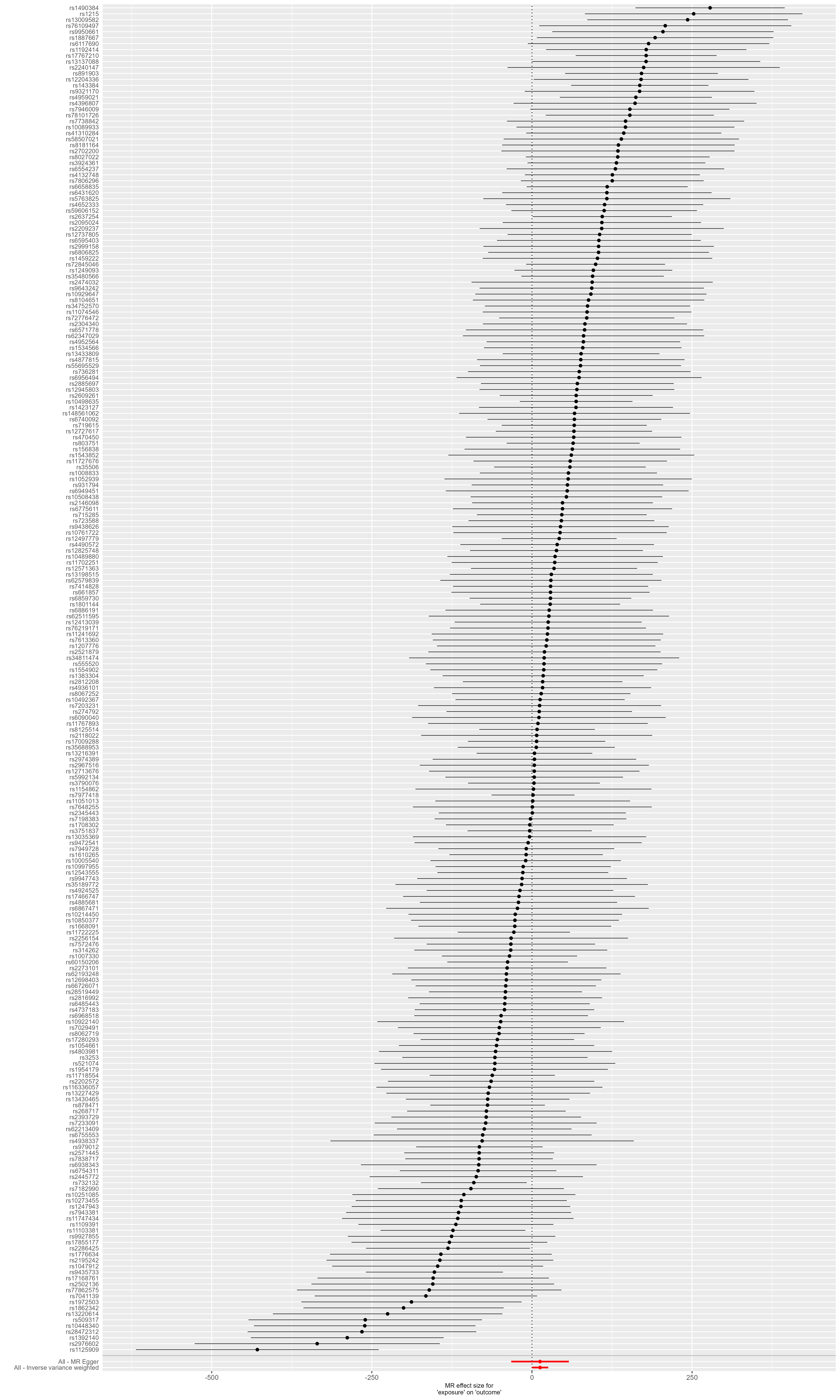

Supplement: Supplementary file 12 — Supplementary Material 12. [file 12890_2024_3150_MOESM12_ESM.zip › Supplementary Figure/Forest plot/Cortex Surface area/forest_plotFVC_insula_surfavg_noGC.png]

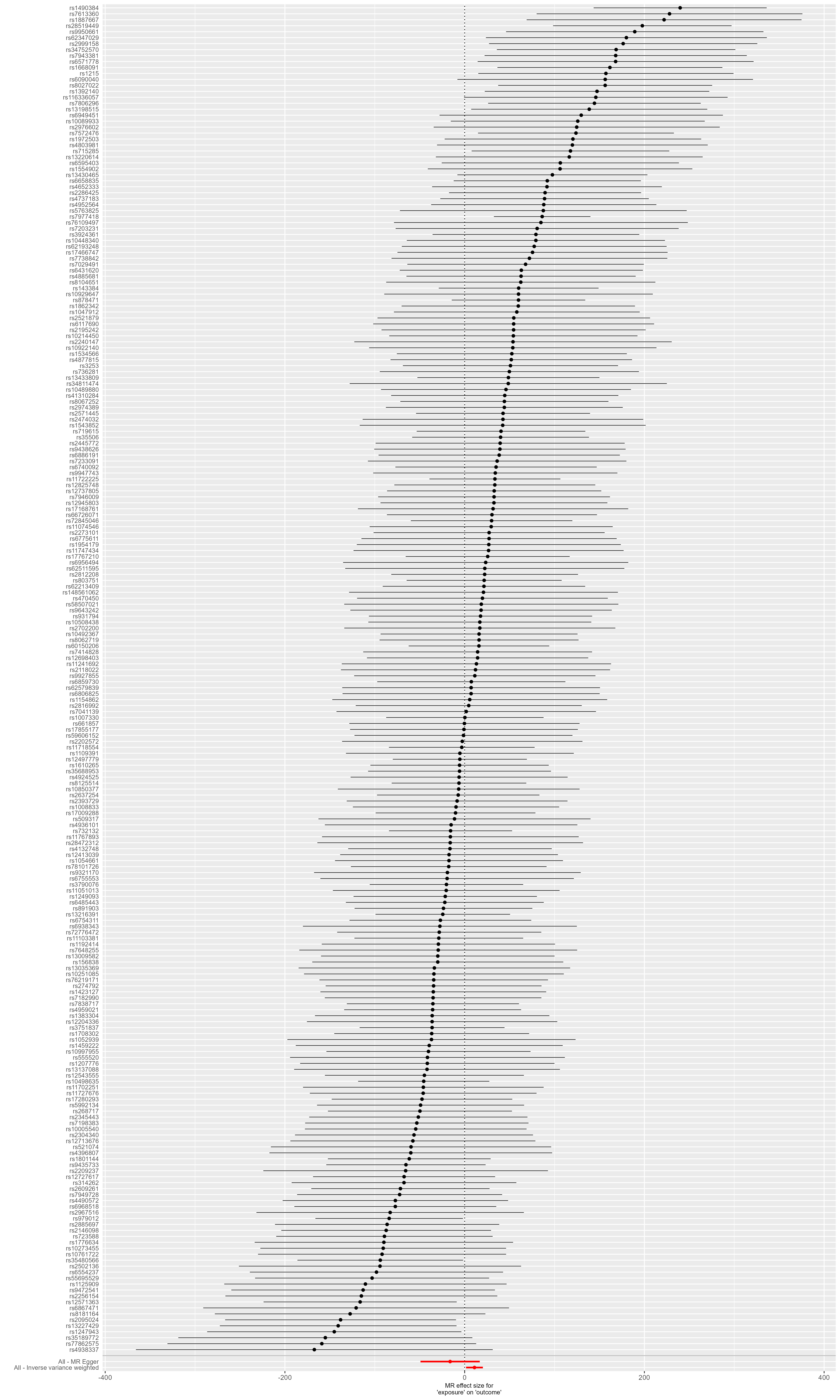

Supplement: Supplementary file 12 — Supplementary Material 12. [file 12890_2024_3150_MOESM12_ESM.zip › Supplementary Figure/Forest plot/Cortex Surface area/forest_plotFVC_medialorbitofrontal_surfavg.png]

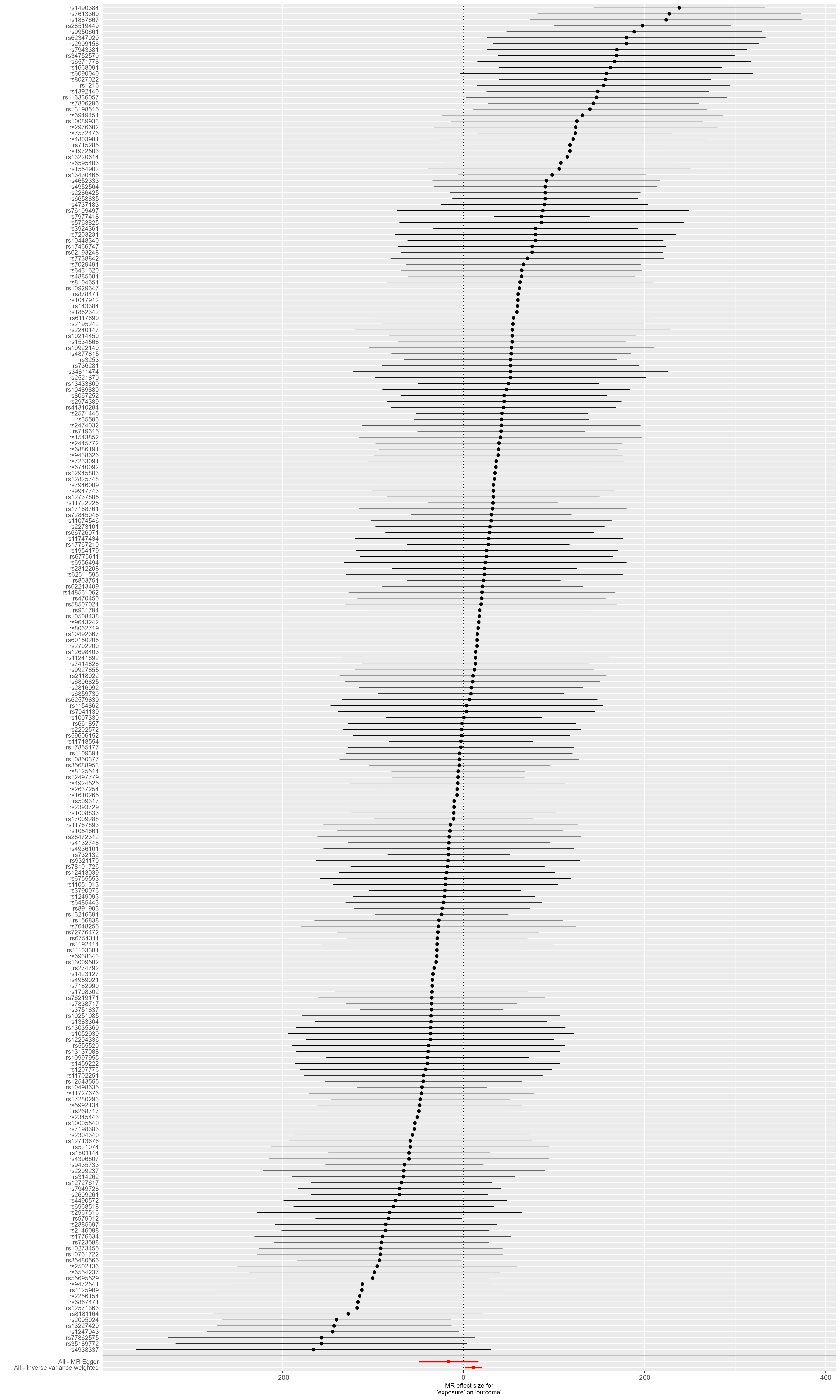

Supplement: Supplementary file 12 — Supplementary Material 12. [file 12890_2024_3150_MOESM12_ESM.zip › Supplementary Figure/Forest plot/Cortex Surface area/forest_plotFVC_medialorbitofrontal_surfavg_noGC.png]

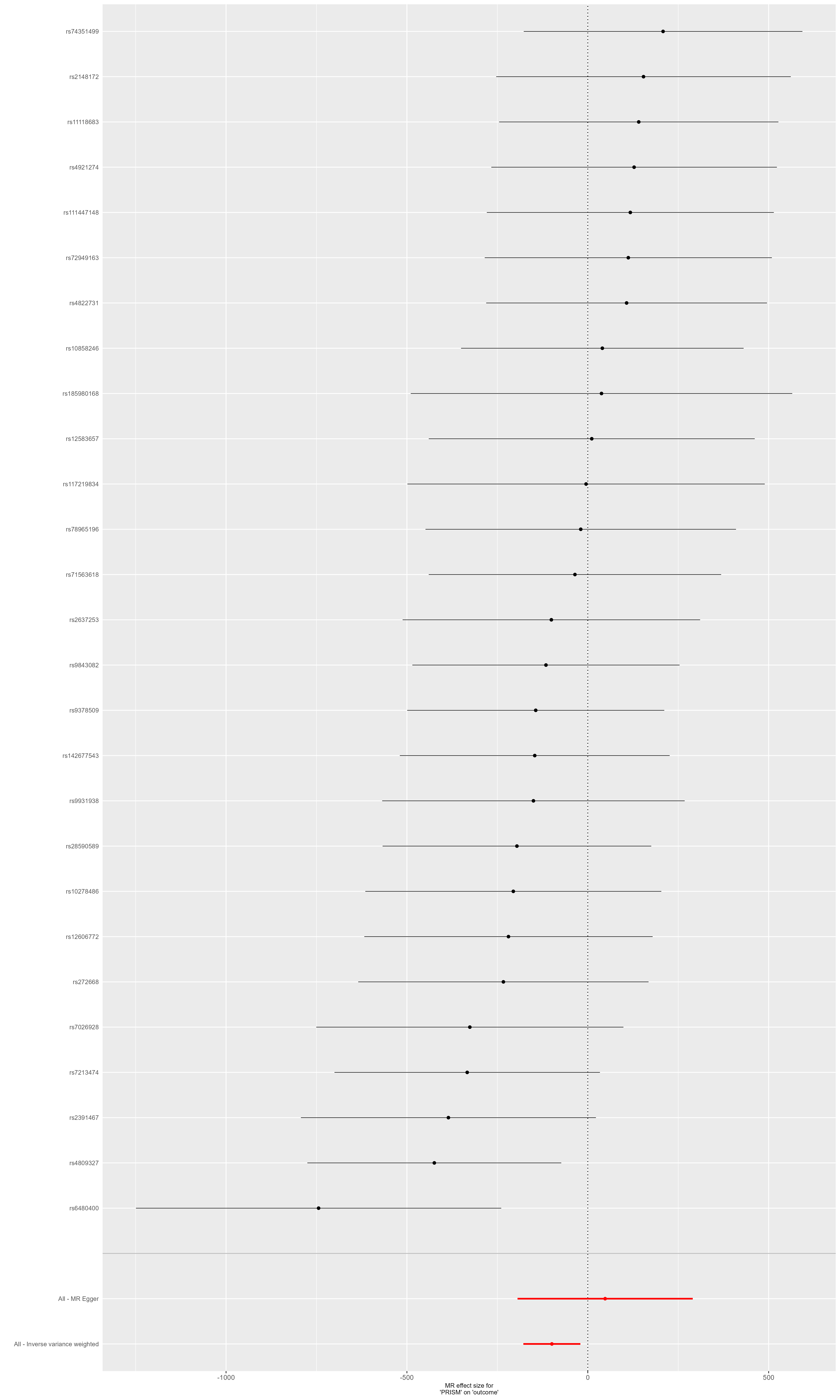

Supplement: Supplementary file 12 — Supplementary Material 12. [file 12890_2024_3150_MOESM12_ESM.zip › Supplementary Figure/Forest plot/Cortex Surface area/forest_plotPRISM_paracentral_surfavg_GC.png]

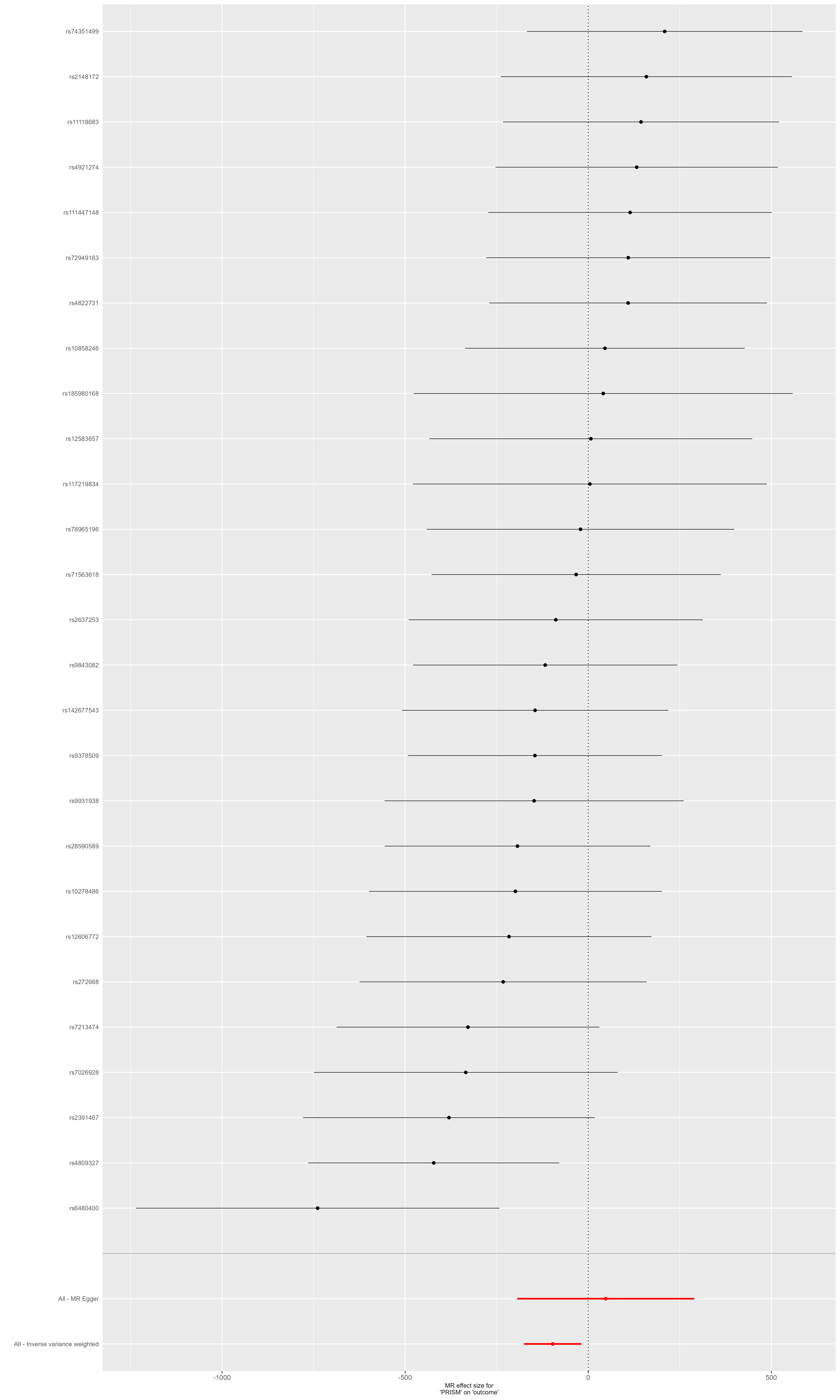

Supplement: Supplementary file 12 — Supplementary Material 12. [file 12890_2024_3150_MOESM12_ESM.zip › Supplementary Figure/Forest plot/Cortex Surface area/forest_plotPRISM_paracentral_surfavg_noGC.png]

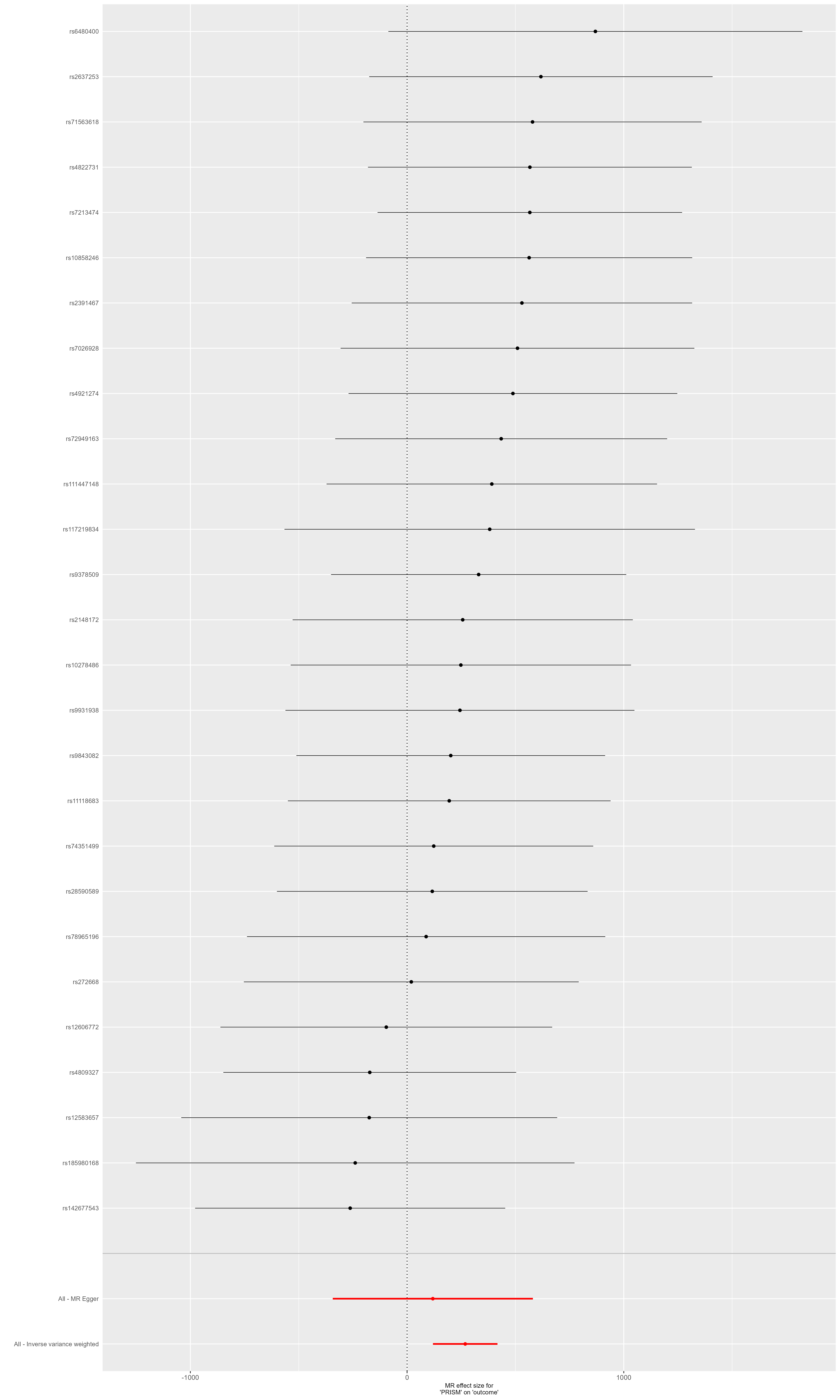

Supplement: Supplementary file 12 — Supplementary Material 12. [file 12890_2024_3150_MOESM12_ESM.zip › Supplementary Figure/Forest plot/Cortex Surface area/forest_plotPRISM_precuneus_surfavg_GC.png]

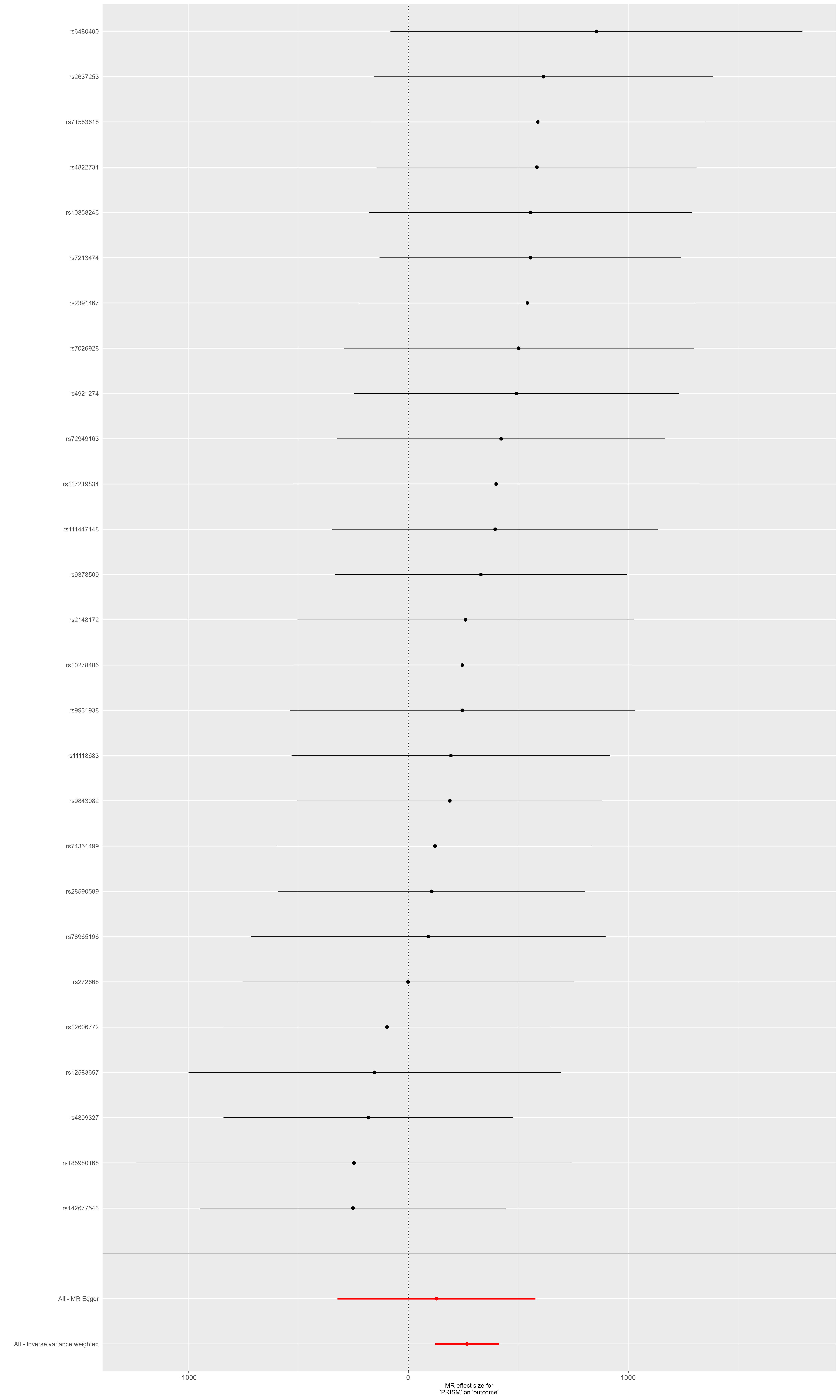

Supplement: Supplementary file 12 — Supplementary Material 12. [file 12890_2024_3150_MOESM12_ESM.zip › Supplementary Figure/Forest plot/Cortex Surface area/forest_plotPRISM_precuneus_surfavg_noGC.png]

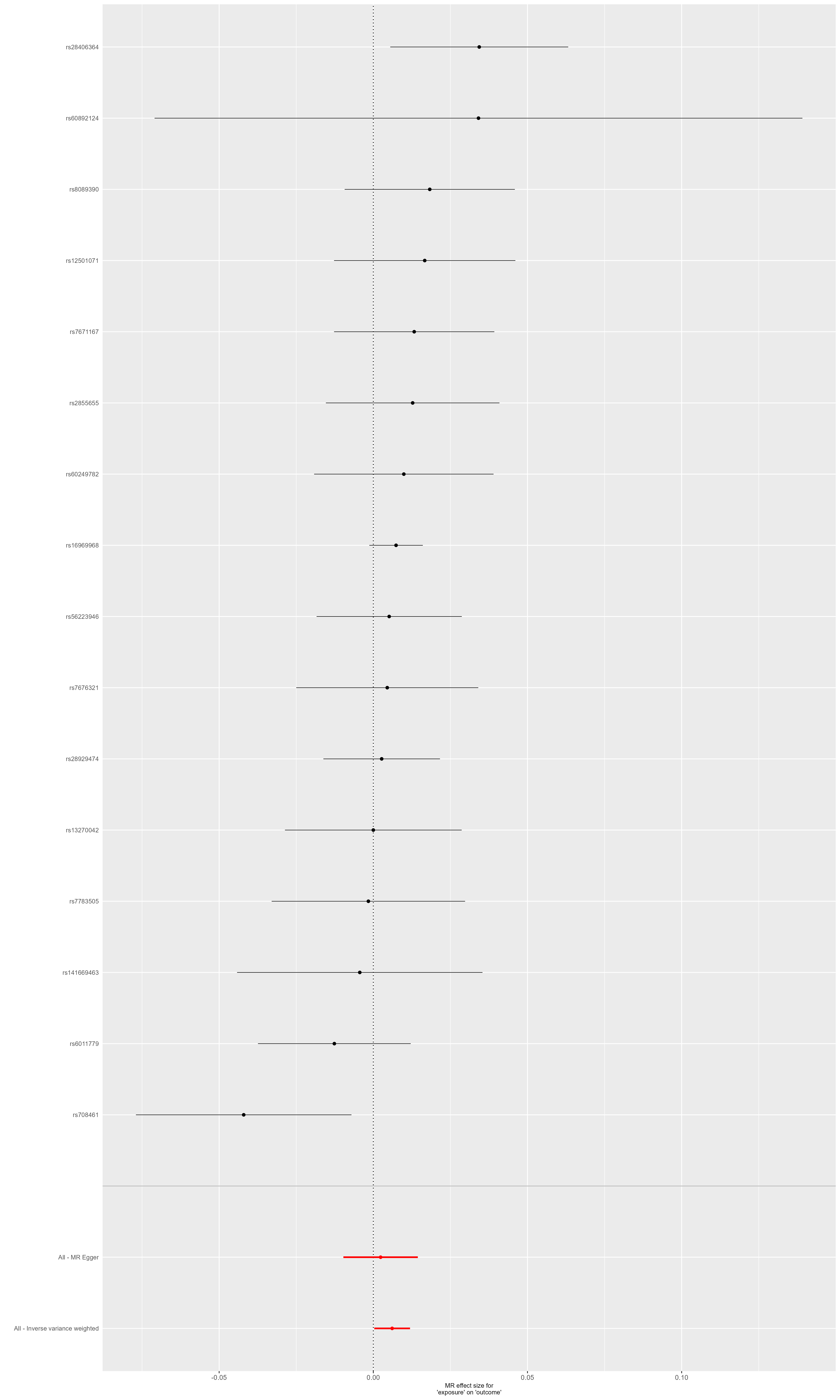

Supplement: Supplementary file 12 — Supplementary Material 12. [file 12890_2024_3150_MOESM12_ESM.zip › Supplementary Figure/Forest plot/Cortex Thickness/forest_plotCOPD_cuneus_thickavg.png]

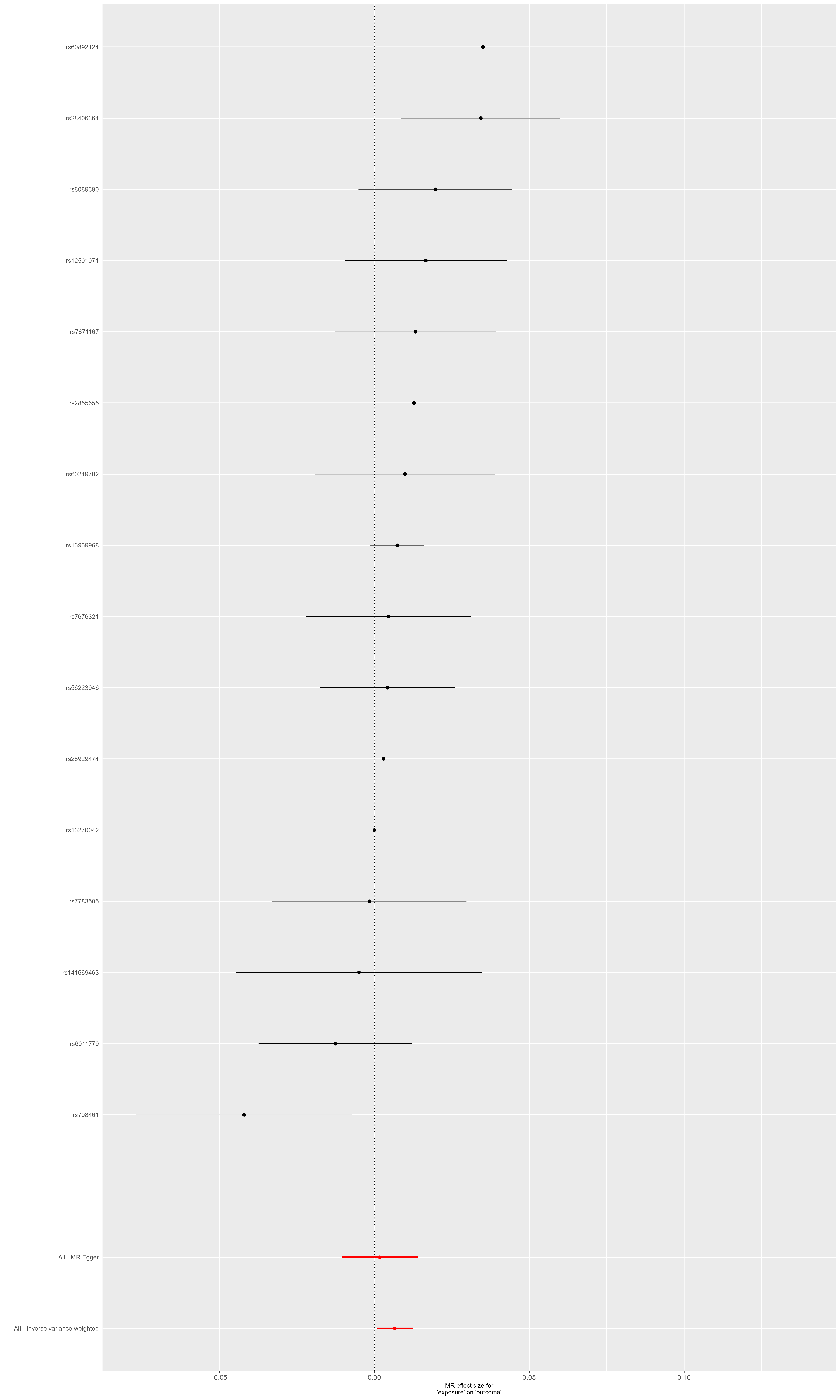

Supplement: Supplementary file 12 — Supplementary Material 12. [file 12890_2024_3150_MOESM12_ESM.zip › Supplementary Figure/Forest plot/Cortex Thickness/forest_plotCOPD_cuneus_thickavg_noGC.png]

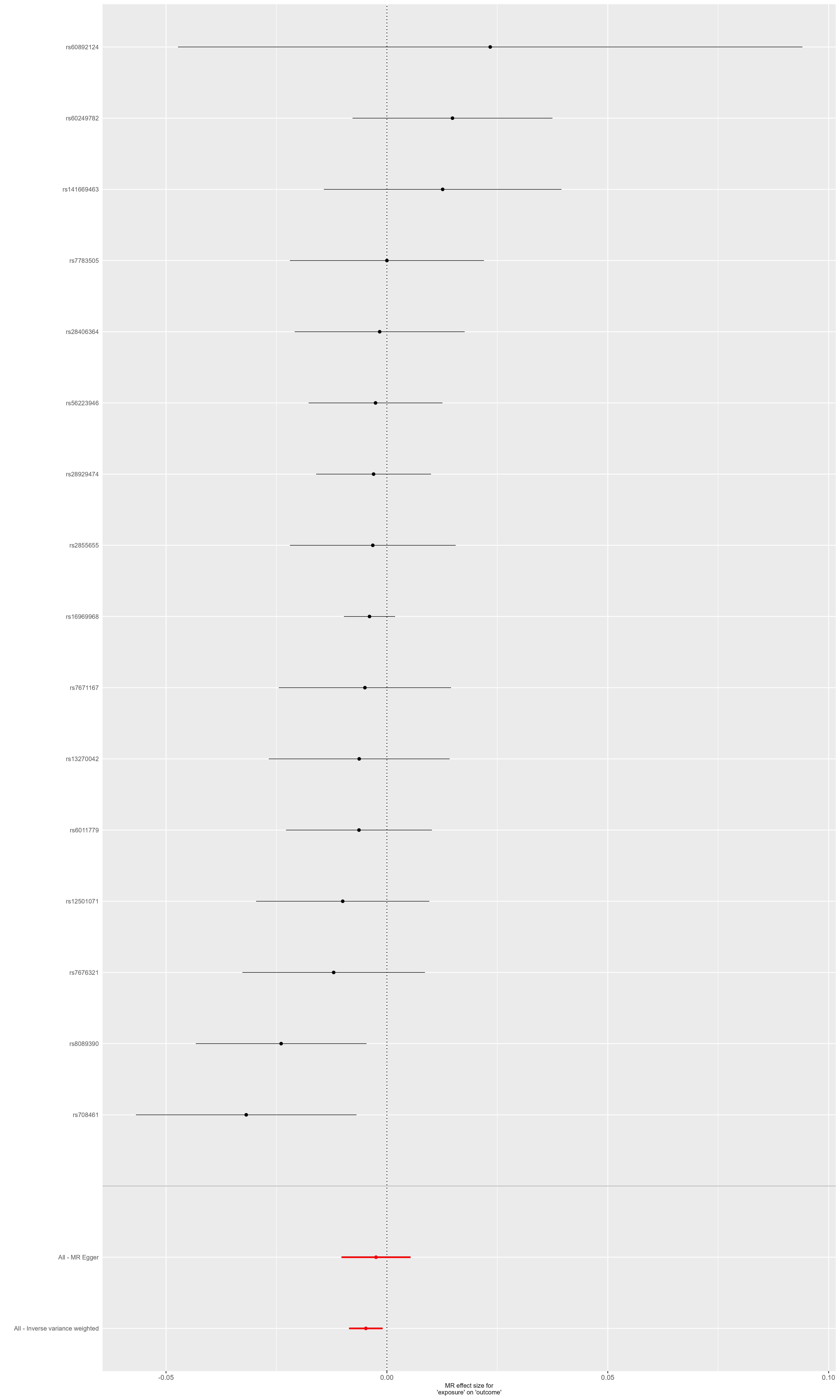

Supplement: Supplementary file 12 — Supplementary Material 12. [file 12890_2024_3150_MOESM12_ESM.zip › Supplementary Figure/Forest plot/Cortex Thickness/forest_plotCOPD_inferiorparietal_thickavg.png]

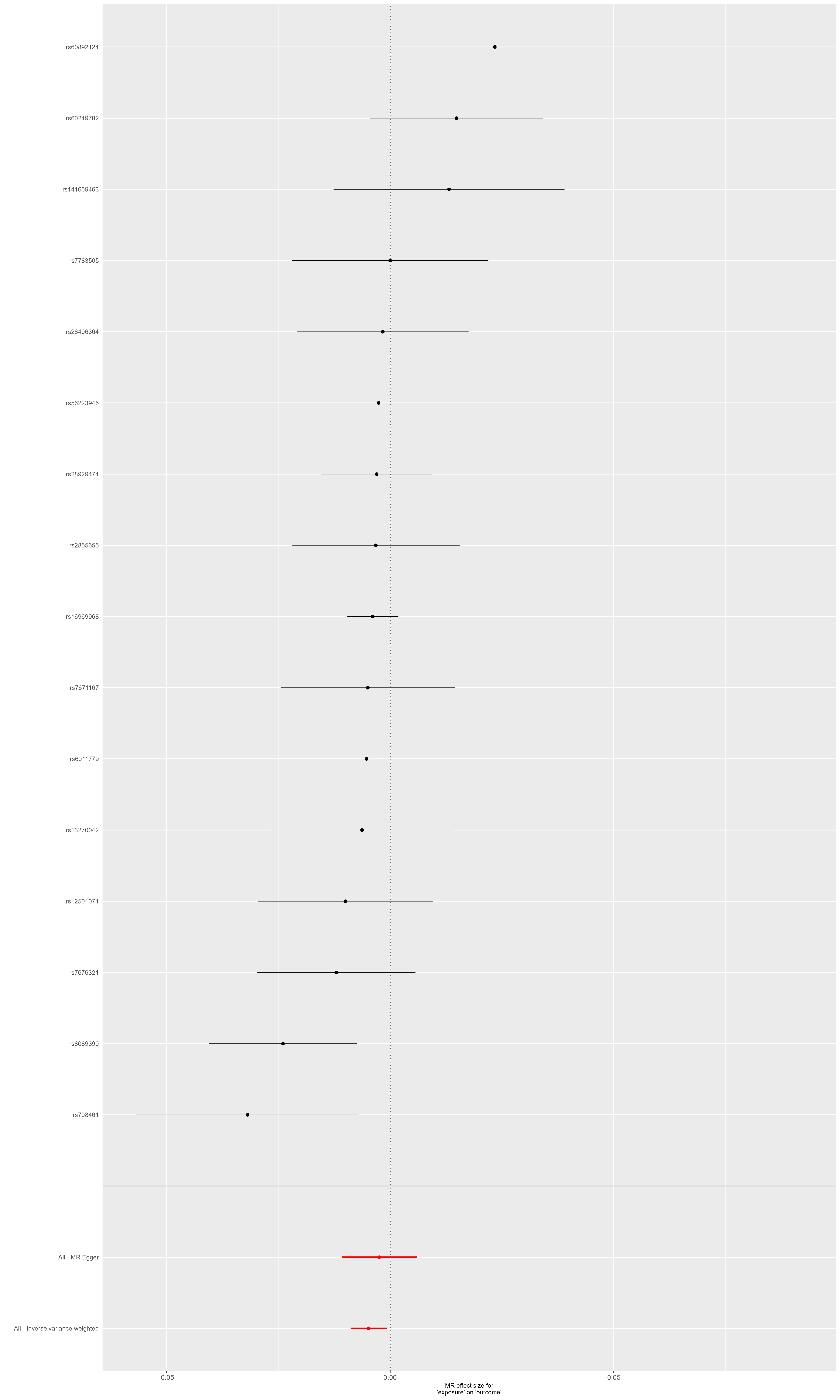

Supplement: Supplementary file 12 — Supplementary Material 12. [file 12890_2024_3150_MOESM12_ESM.zip › Supplementary Figure/Forest plot/Cortex Thickness/forest_plotCOPD_inferiorparietal_thickavg_noGC.png]

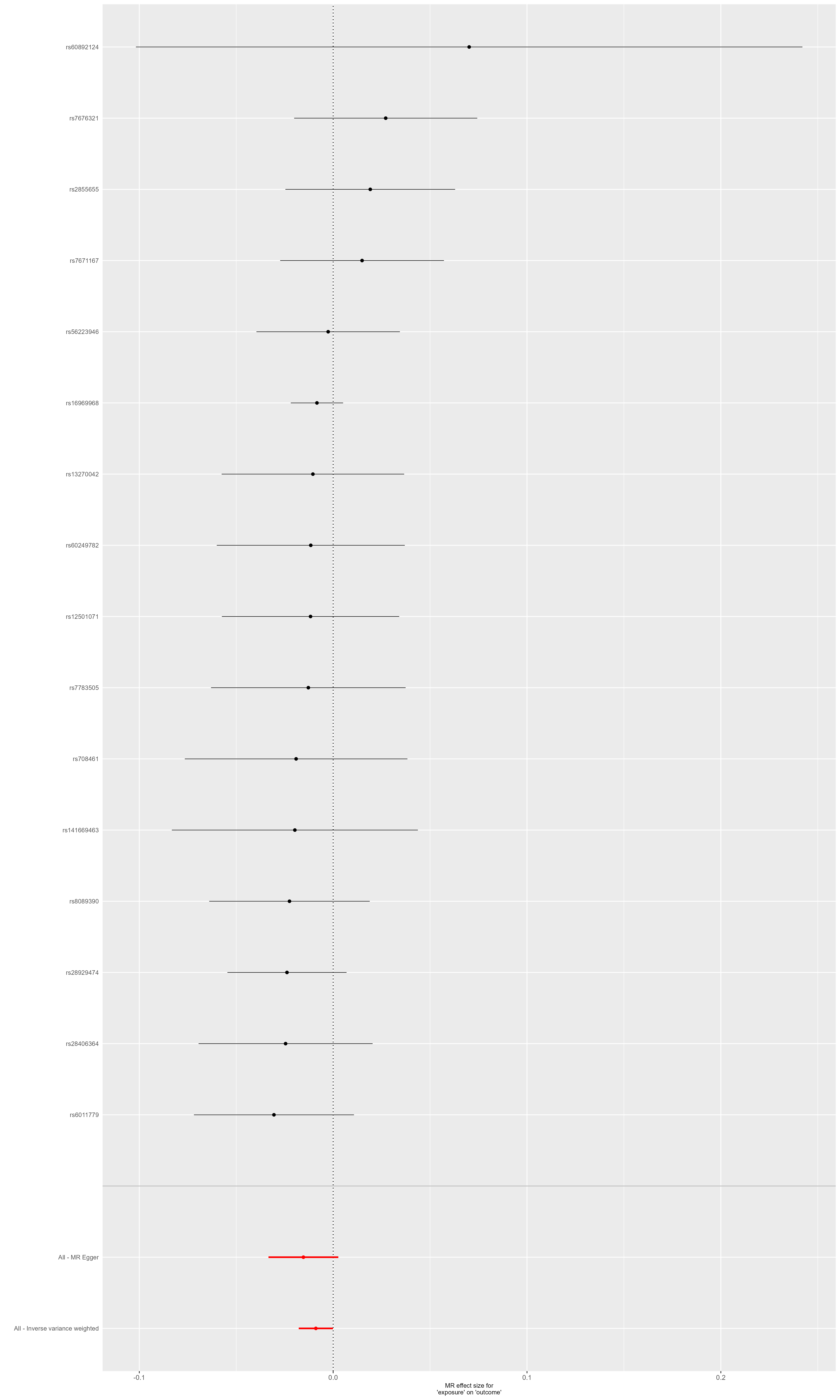

Supplement: Supplementary file 12 — Supplementary Material 12. [file 12890_2024_3150_MOESM12_ESM.zip › Supplementary Figure/Forest plot/Cortex Thickness/forest_plotCOPD_rostralanteriorcingulate_thickavg_noGC.png]

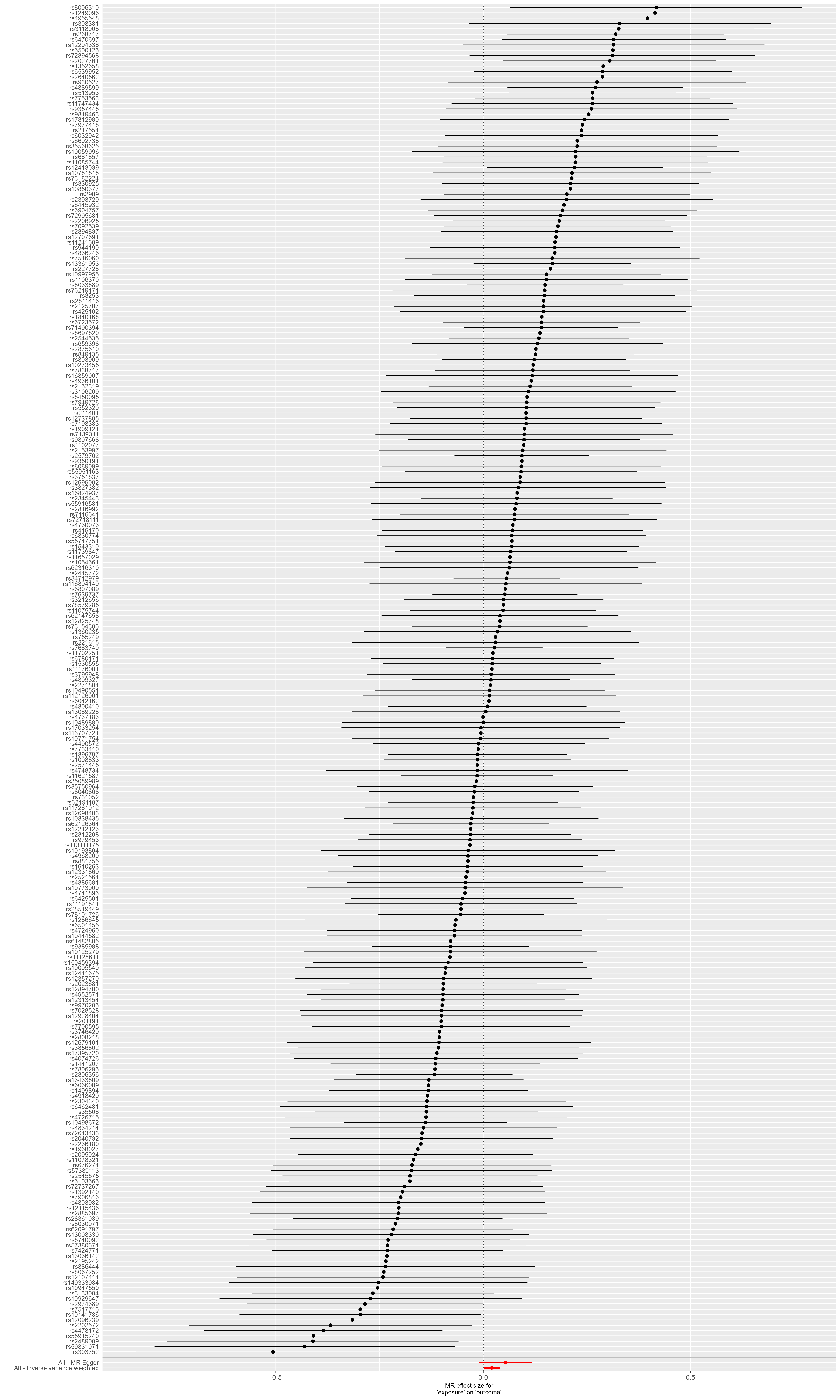

Supplement: Supplementary file 12 — Supplementary Material 12. [file 12890_2024_3150_MOESM12_ESM.zip › Supplementary Figure/Forest plot/Cortex Thickness/forest_plotFEV1_entorhinal_thickavg.png]

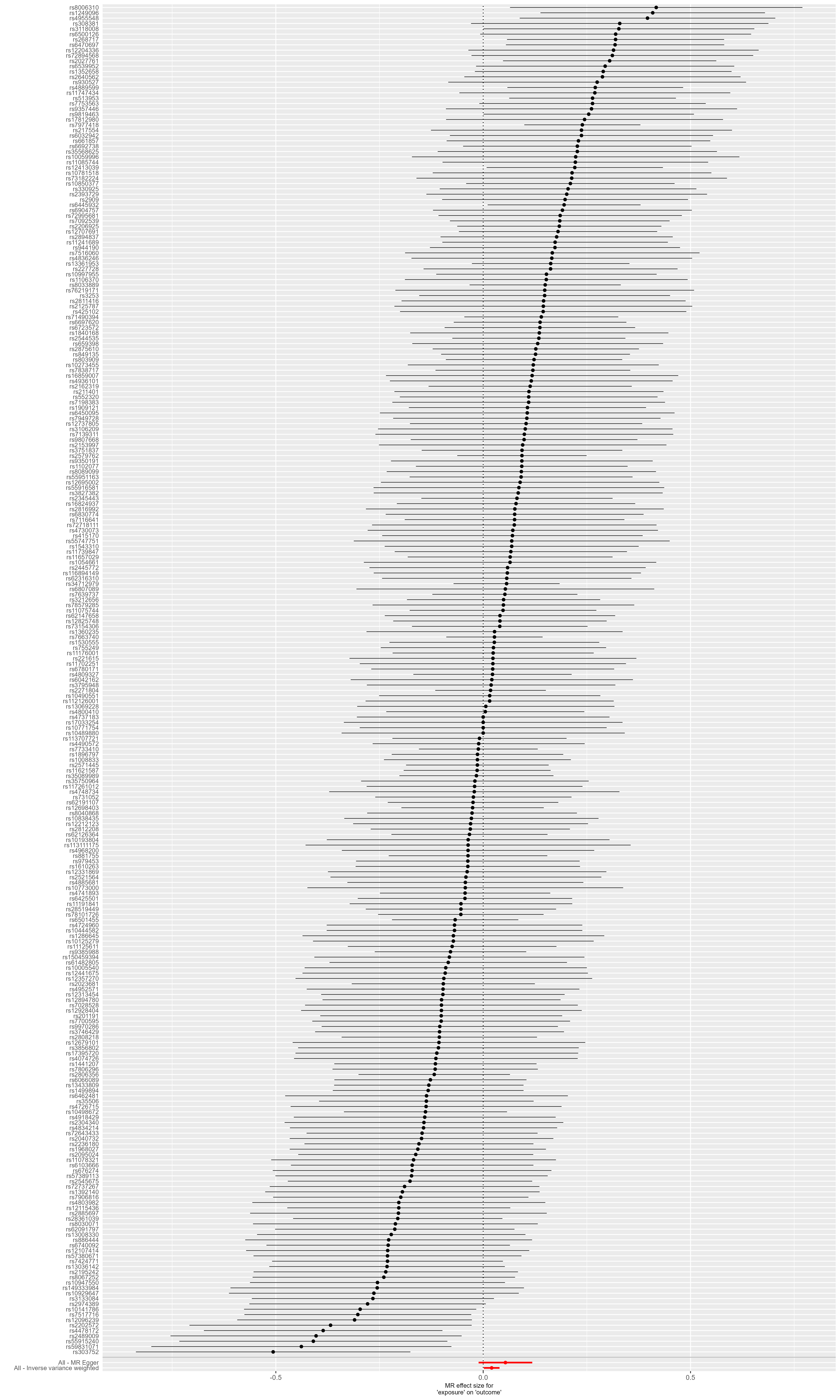

Supplement: Supplementary file 12 — Supplementary Material 12. [file 12890_2024_3150_MOESM12_ESM.zip › Supplementary Figure/Forest plot/Cortex Thickness/forest_plotFEV1_entorhinal_thickavg_noGC.png]

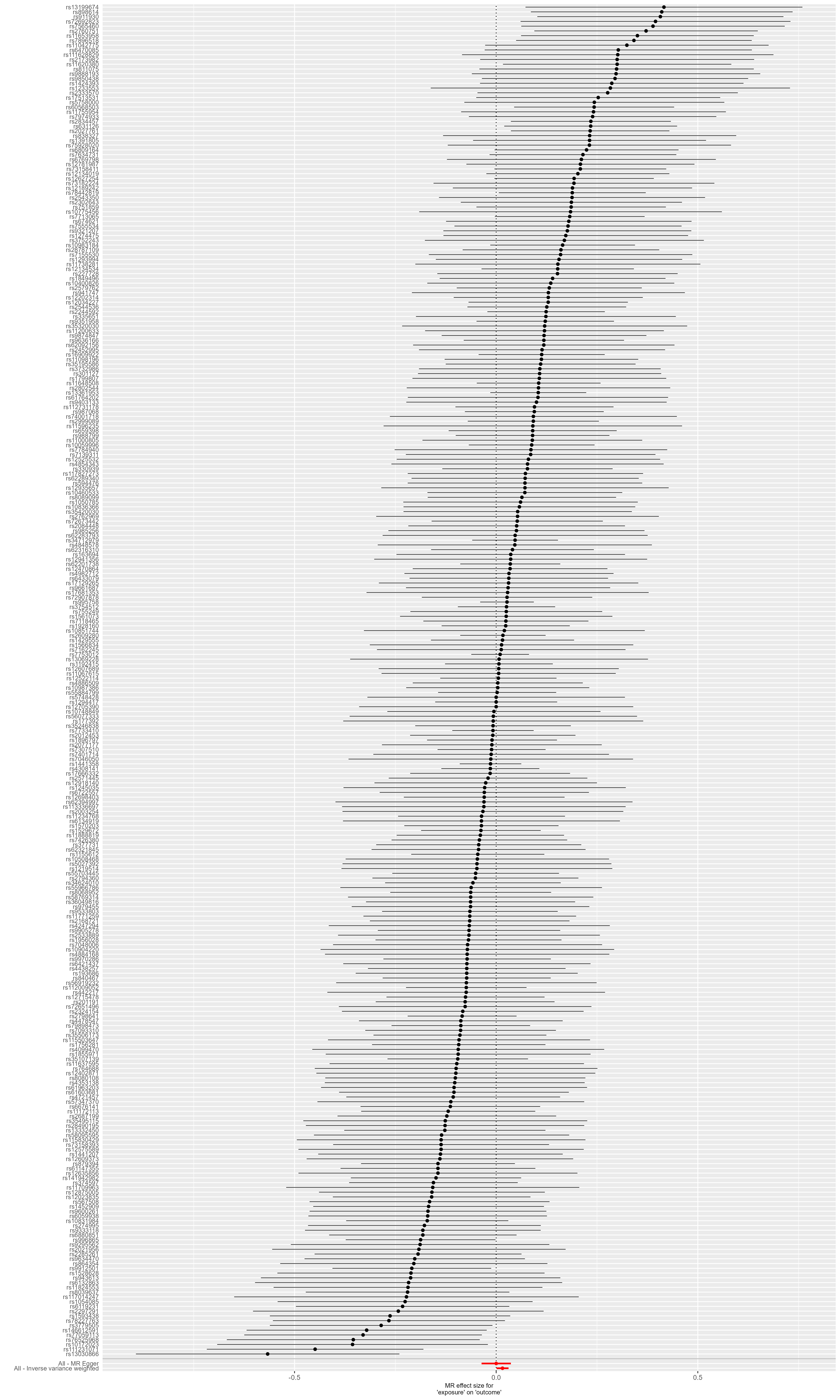

Supplement: Supplementary file 12 — Supplementary Material 12. [file 12890_2024_3150_MOESM12_ESM.zip › Supplementary Figure/Forest plot/Cortex Thickness/forest_plotFEV1_FVC_entorhinal_thickavg.png]

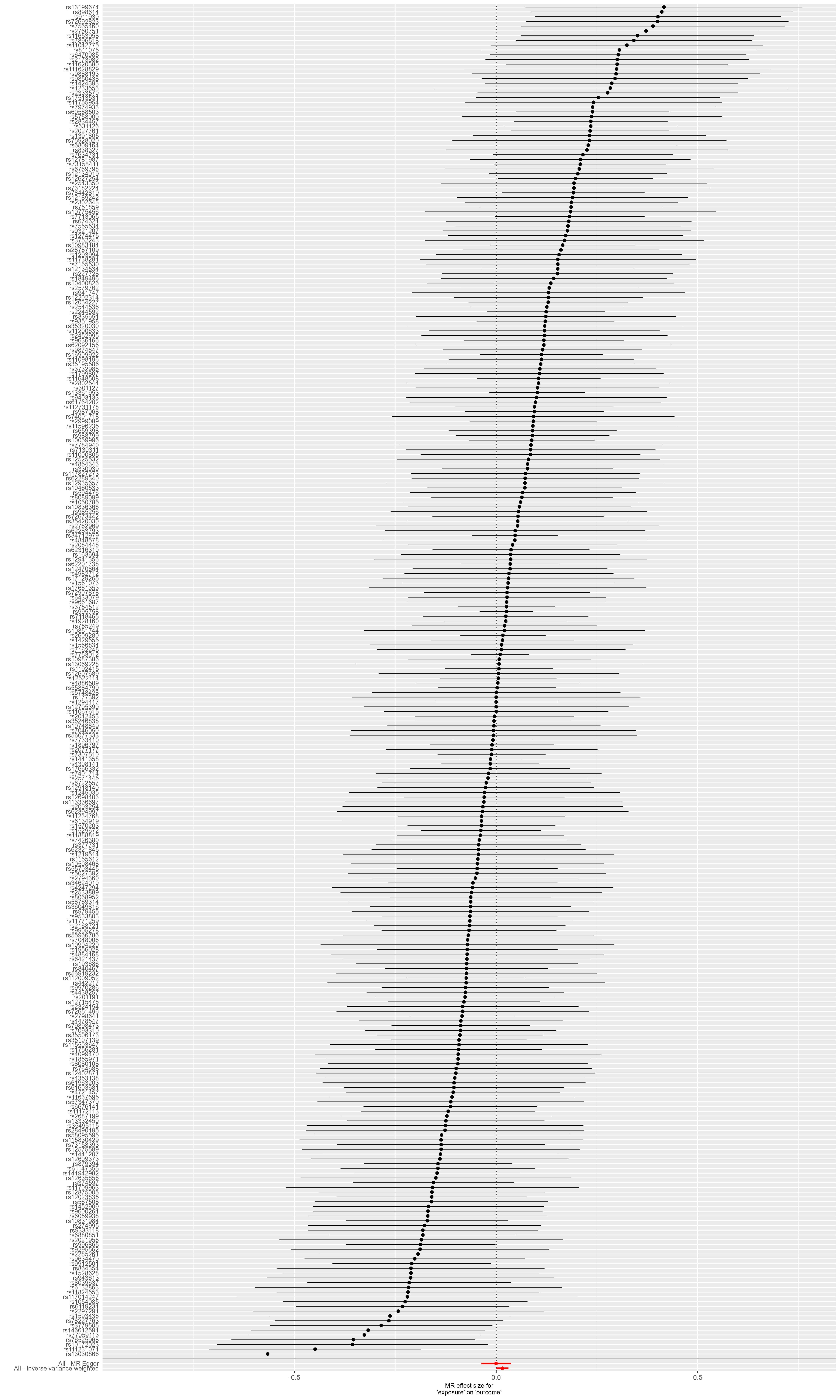

Supplement: Supplementary file 12 — Supplementary Material 12. [file 12890_2024_3150_MOESM12_ESM.zip › Supplementary Figure/Forest plot/Cortex Thickness/forest_plotFEV1_FVC_entorhinal_thickavg_noGC.png]

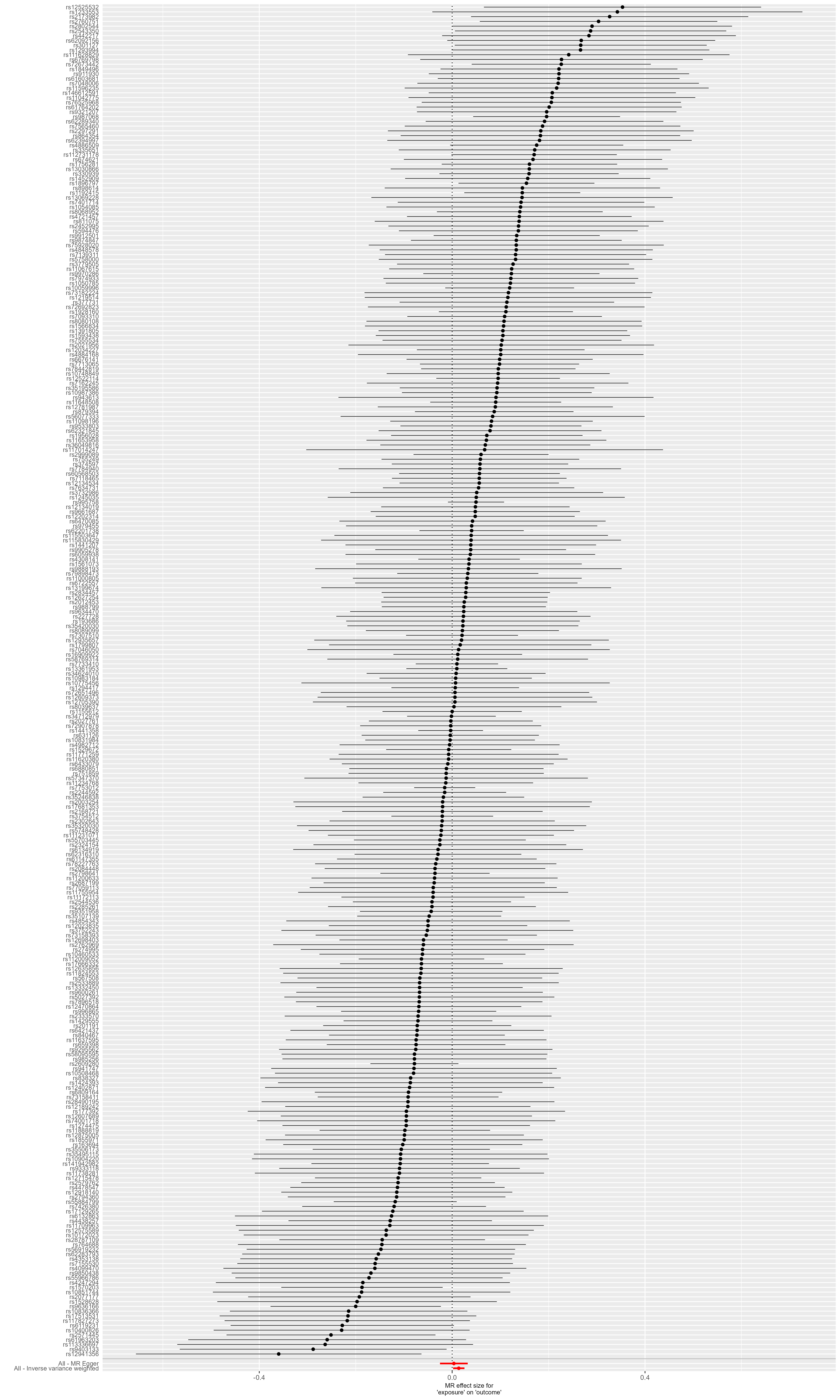

Supplement: Supplementary file 12 — Supplementary Material 12. [file 12890_2024_3150_MOESM12_ESM.zip › Supplementary Figure/Forest plot/Cortex Thickness/forest_plotFEV1_FVC_temporalpole_thickavg.png]

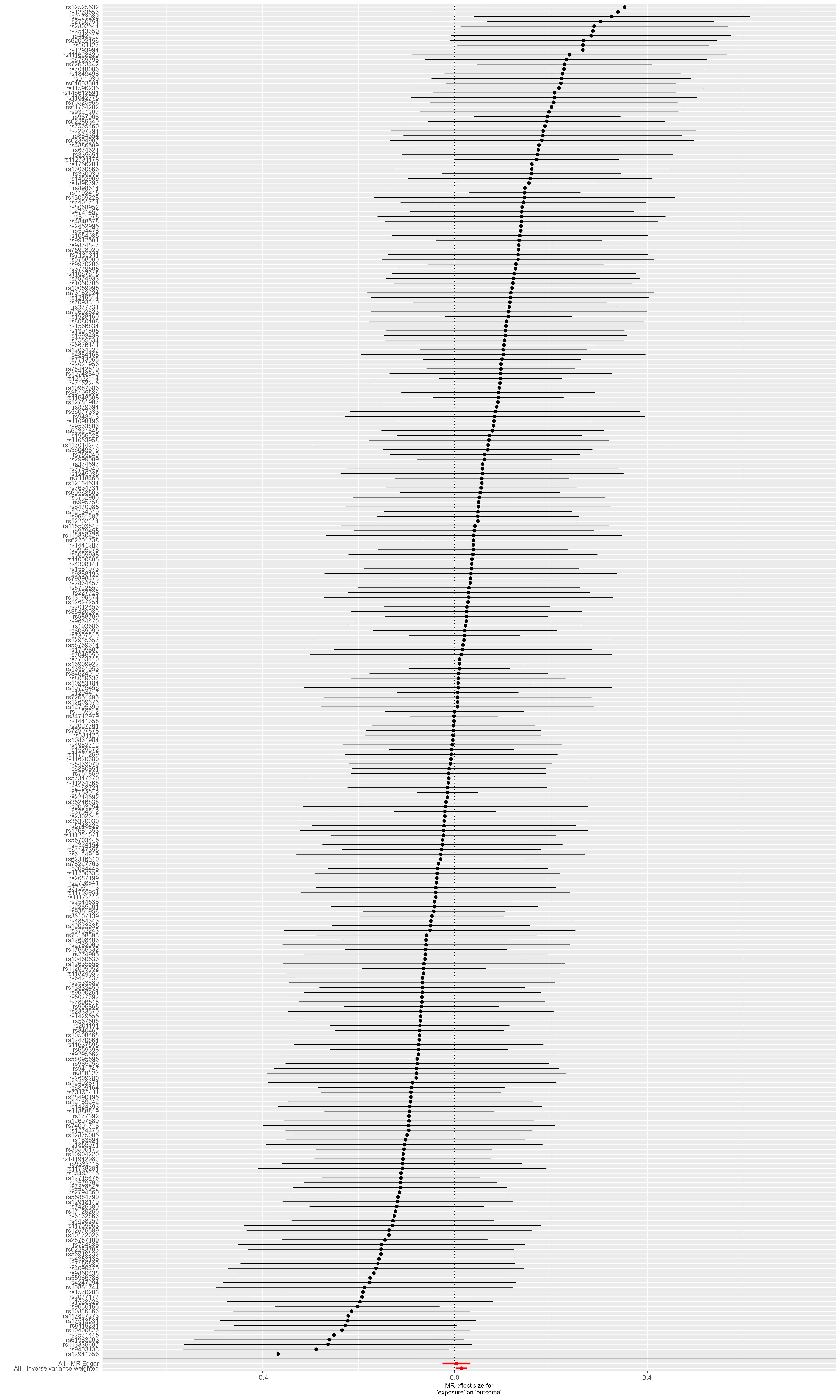

Supplement: Supplementary file 12 — Supplementary Material 12. [file 12890_2024_3150_MOESM12_ESM.zip › Supplementary Figure/Forest plot/Cortex Thickness/forest_plotFEV1_FVC_temporalpole_thickavg_noGC.png]

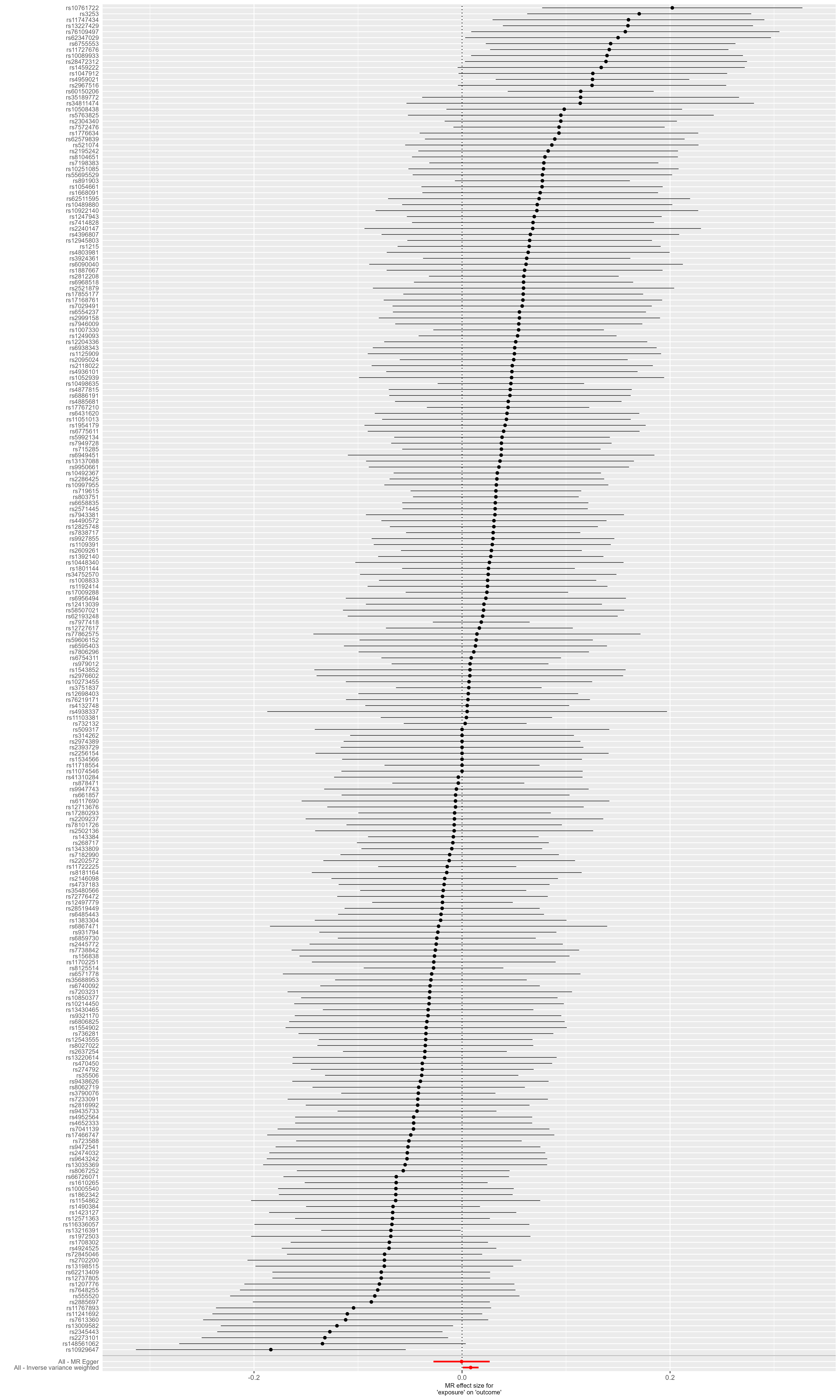

Supplement: Supplementary file 12 — Supplementary Material 12. [file 12890_2024_3150_MOESM12_ESM.zip › Supplementary Figure/Forest plot/Cortex Thickness/forest_plotFVC_bankssts_thickavg.png]

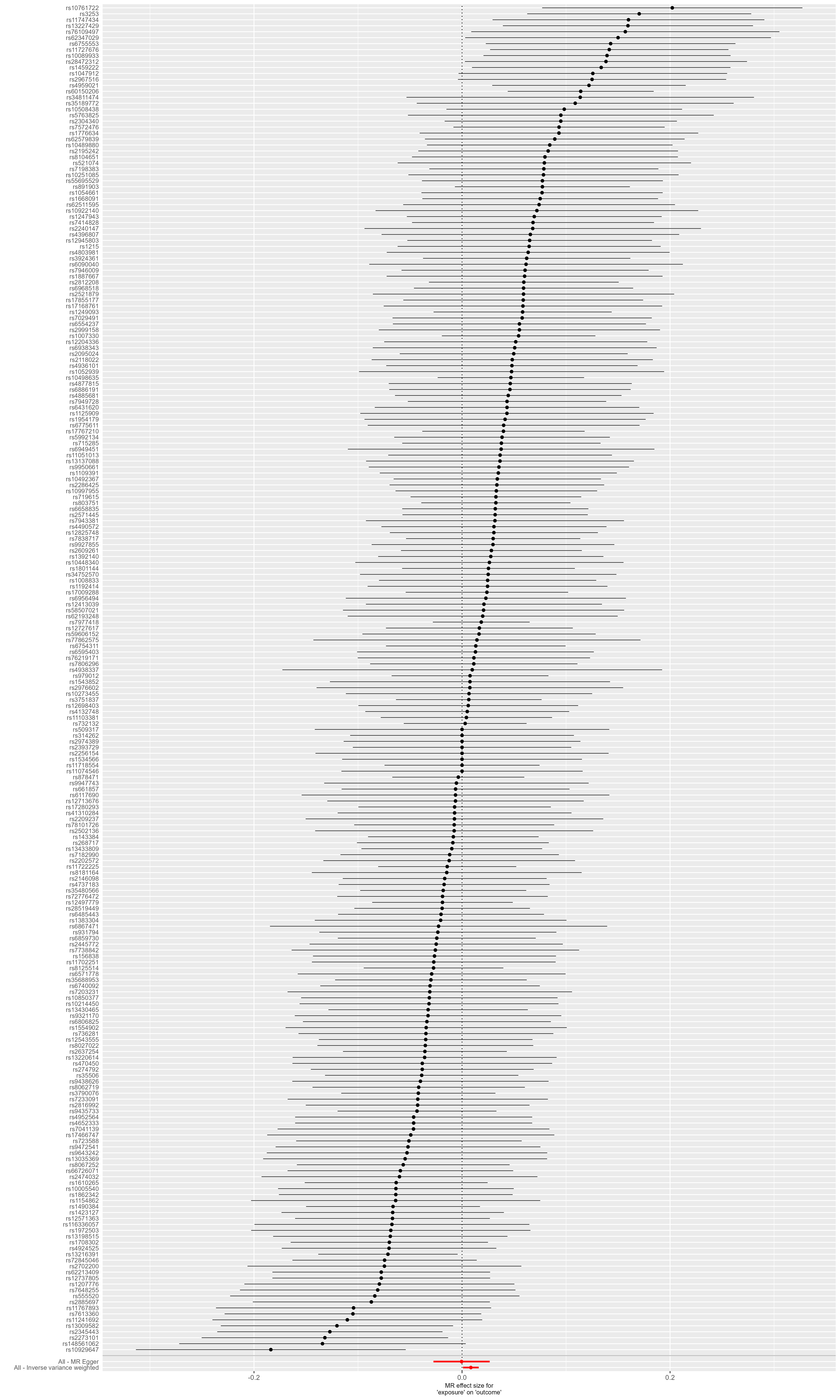

Supplement: Supplementary file 12 — Supplementary Material 12. [file 12890_2024_3150_MOESM12_ESM.zip › Supplementary Figure/Forest plot/Cortex Thickness/forest_plotFVC_bankssts_thickavg_noGC.png]

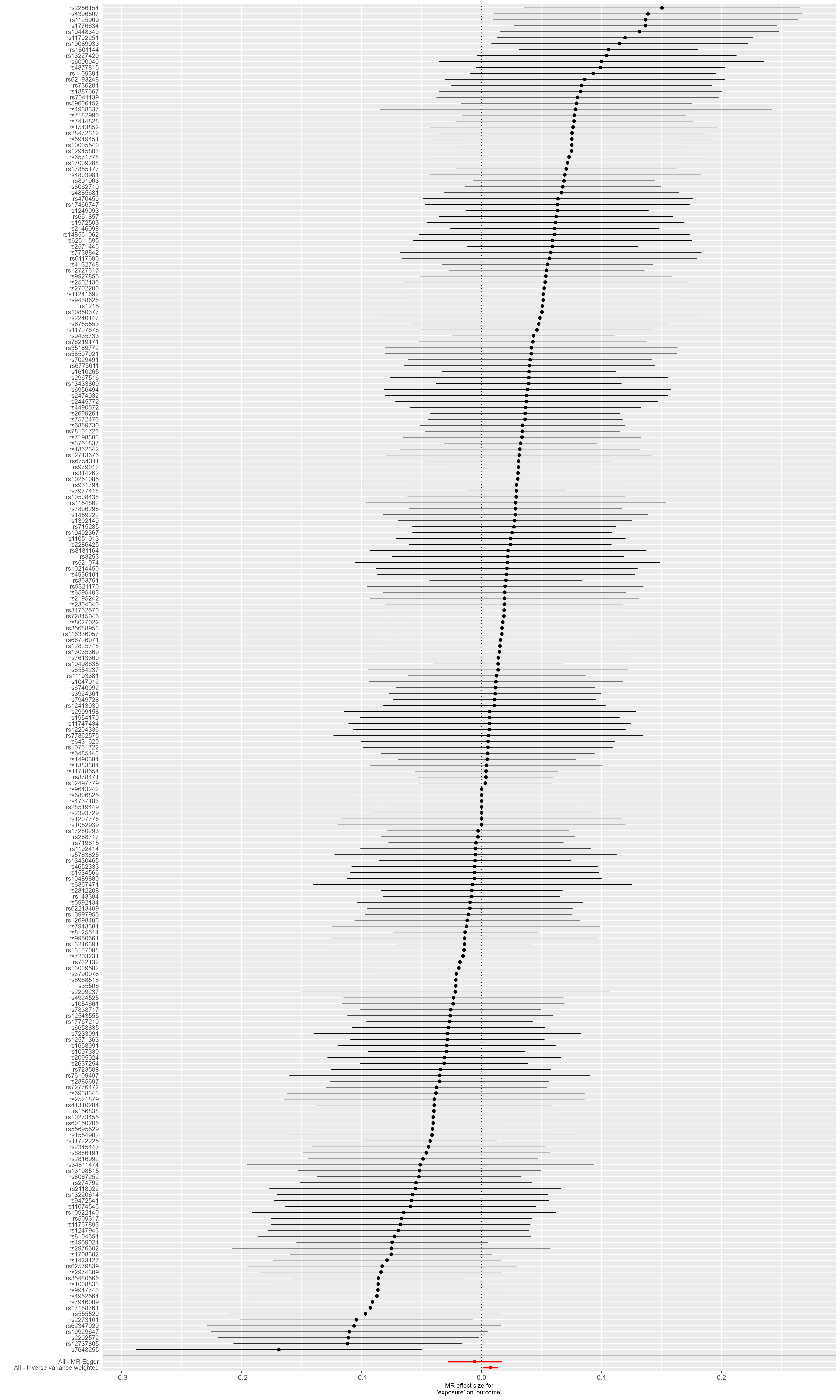

Supplement: Supplementary file 12 — Supplementary Material 12. [file 12890_2024_3150_MOESM12_ESM.zip › Supplementary Figure/Forest plot/Cortex Thickness/forest_plotFVC_fusiform_thickavg.png]

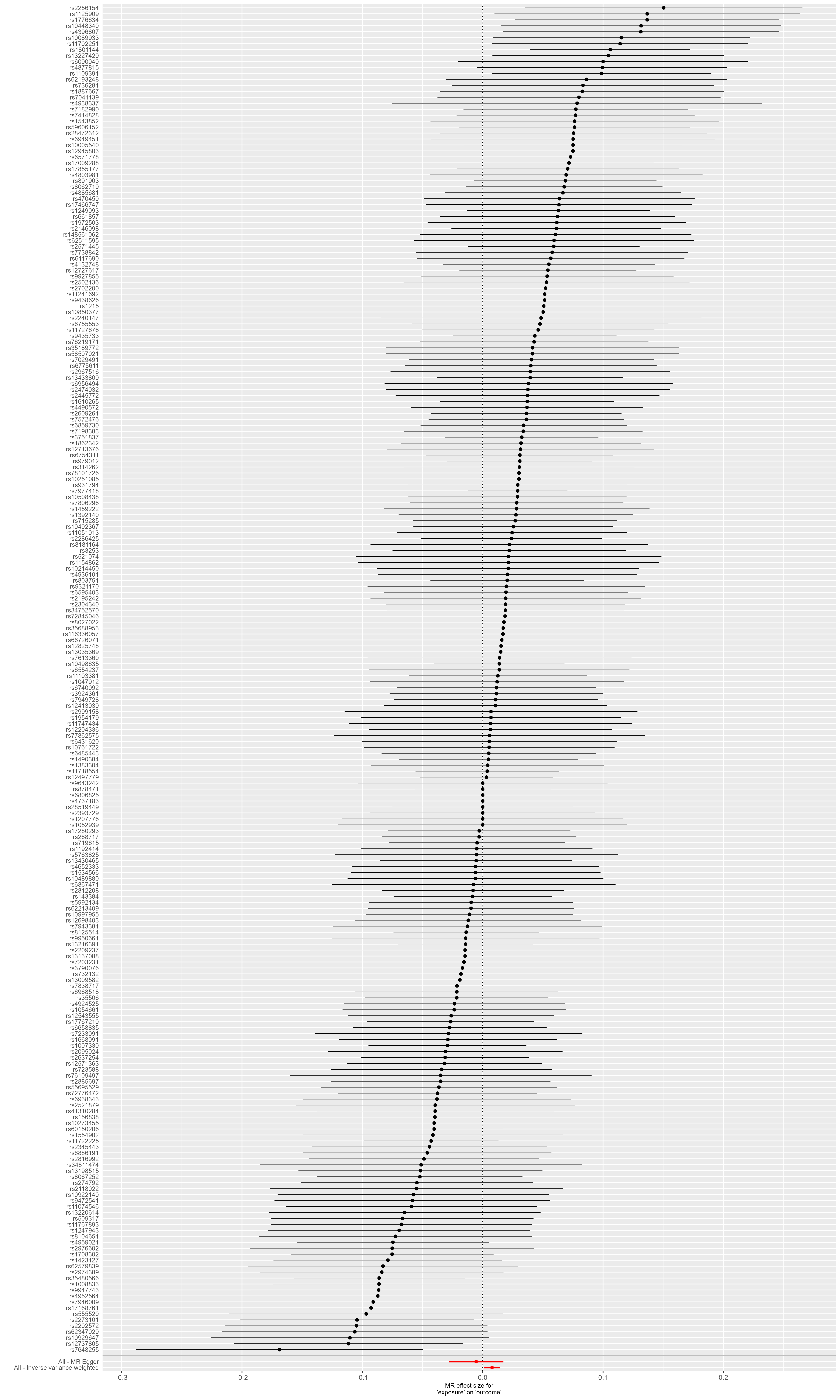

Supplement: Supplementary file 12 — Supplementary Material 12. [file 12890_2024_3150_MOESM12_ESM.zip › Supplementary Figure/Forest plot/Cortex Thickness/forest_plotFVC_fusiform_thickavg_noGC.png]

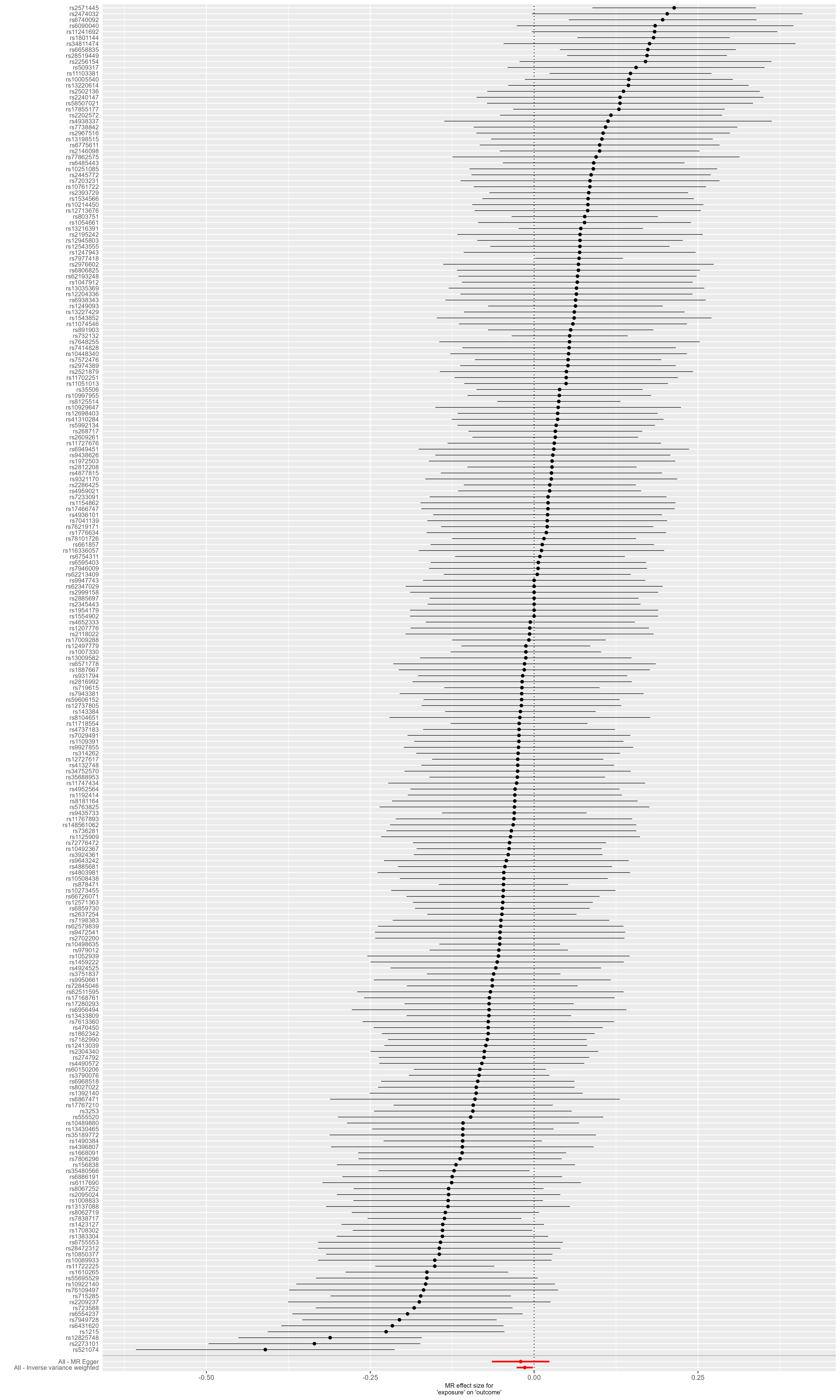

Supplement: Supplementary file 12 — Supplementary Material 12. [file 12890_2024_3150_MOESM12_ESM.zip › Supplementary Figure/Forest plot/Cortex Thickness/forest_plotFVC_isthmuscingulate_thickavg.png]

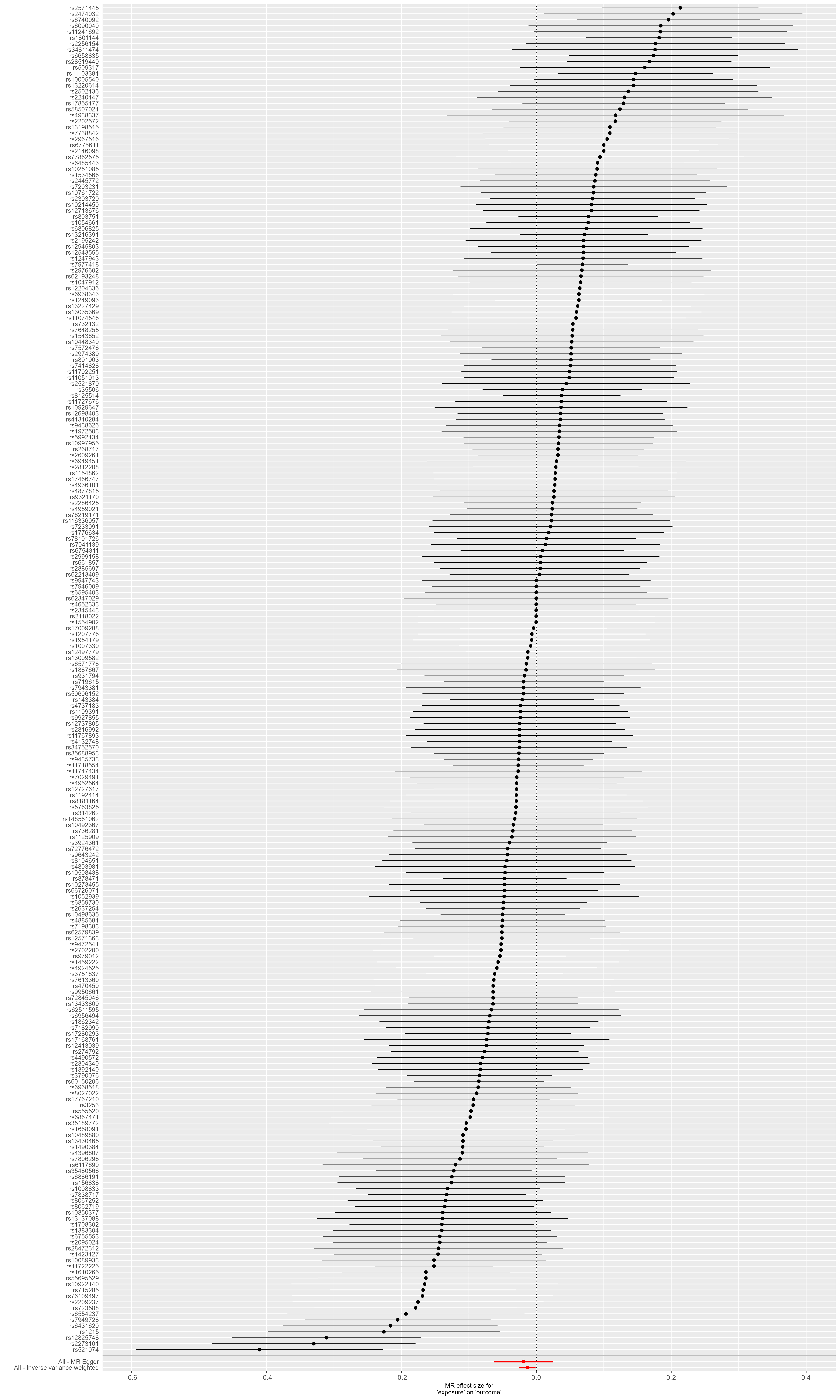

Supplement: Supplementary file 12 — Supplementary Material 12. [file 12890_2024_3150_MOESM12_ESM.zip › Supplementary Figure/Forest plot/Cortex Thickness/forest_plotFVC_isthmuscingulate_thickavg_noGC.png]

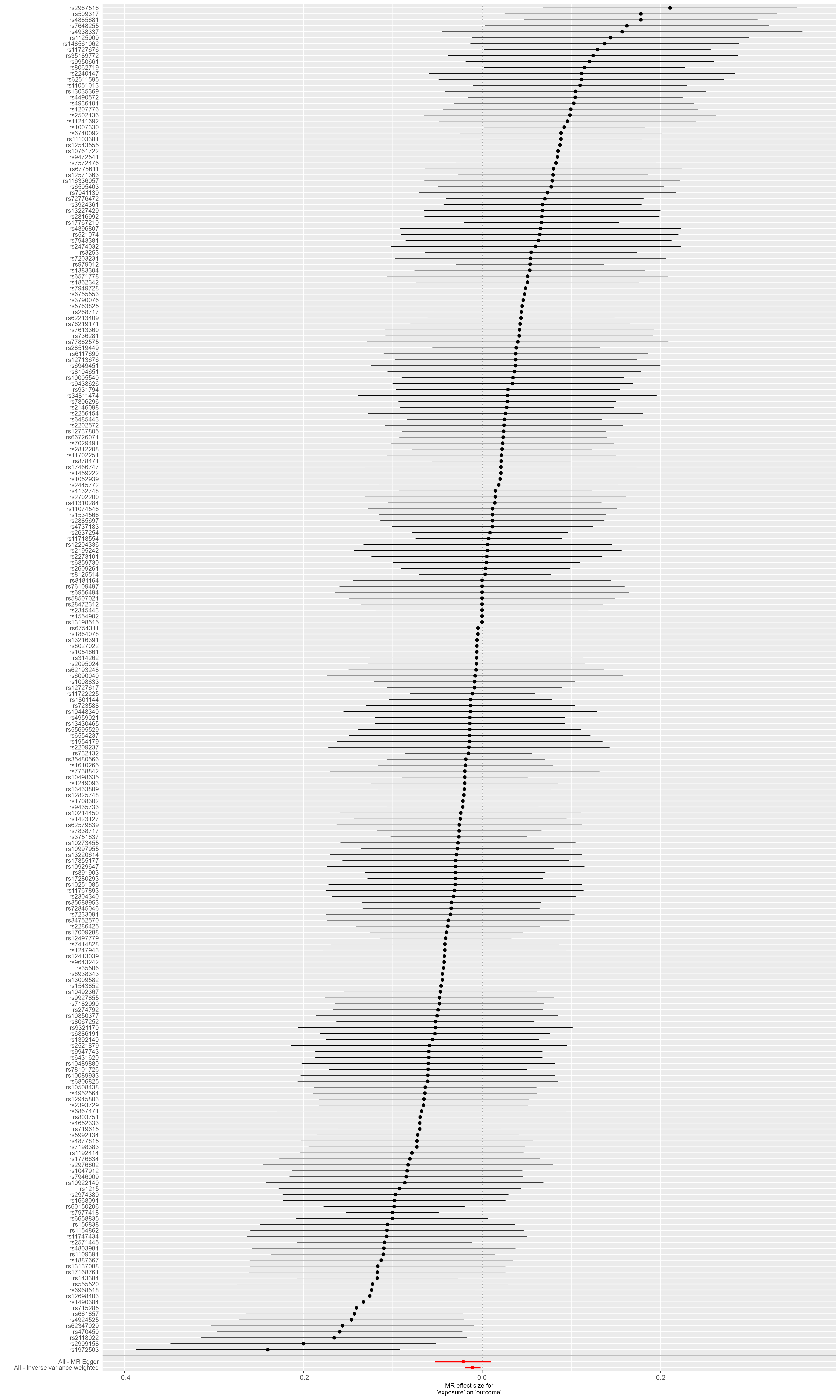

Supplement: Supplementary file 12 — Supplementary Material 12. [file 12890_2024_3150_MOESM12_ESM.zip › Supplementary Figure/Forest plot/Cortex Thickness/forest_plotFVC_medialorbitofrontal_thickavg.png]

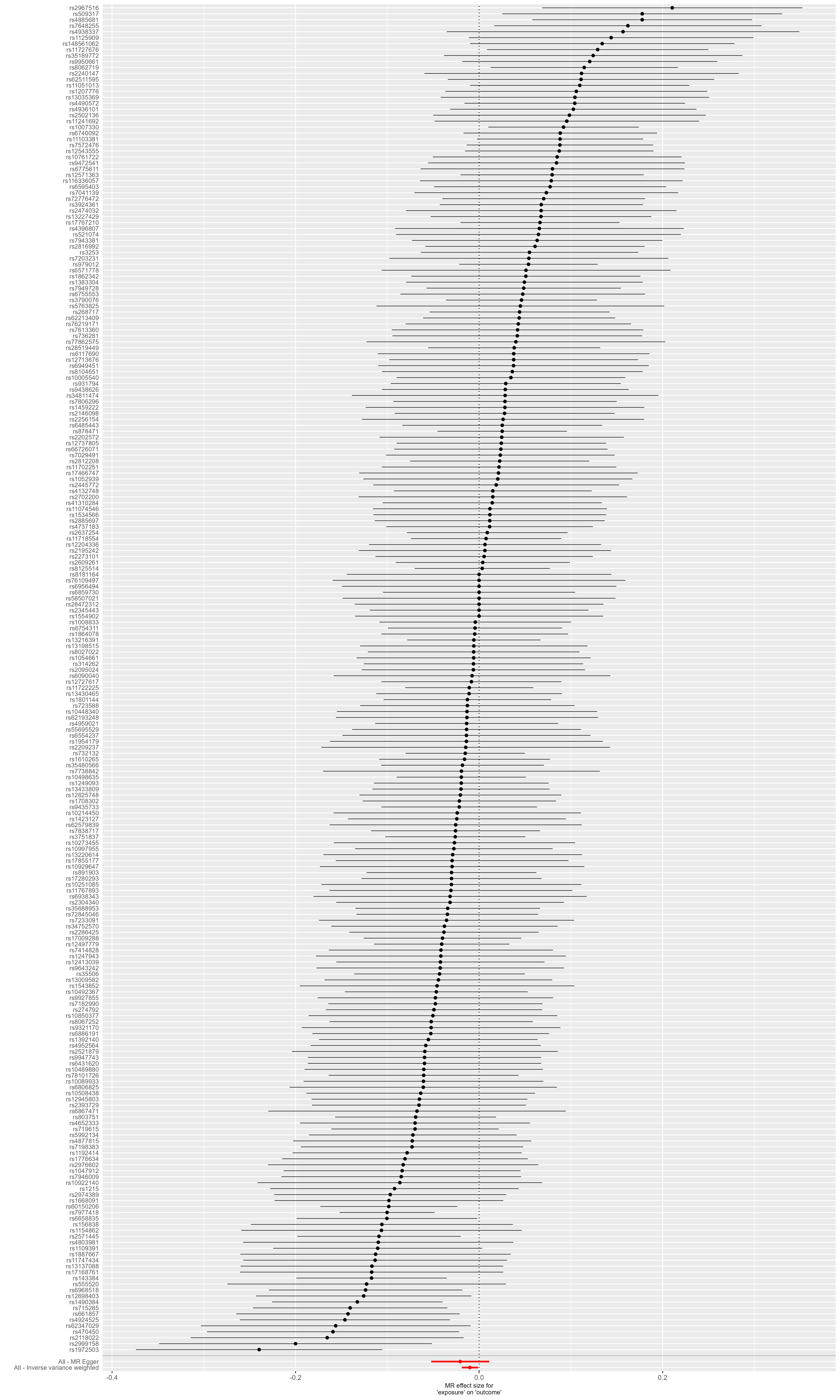

Supplement: Supplementary file 12 — Supplementary Material 12. [file 12890_2024_3150_MOESM12_ESM.zip › Supplementary Figure/Forest plot/Cortex Thickness/forest_plotFVC_medialorbitofrontal_thickavg_noGC.png]

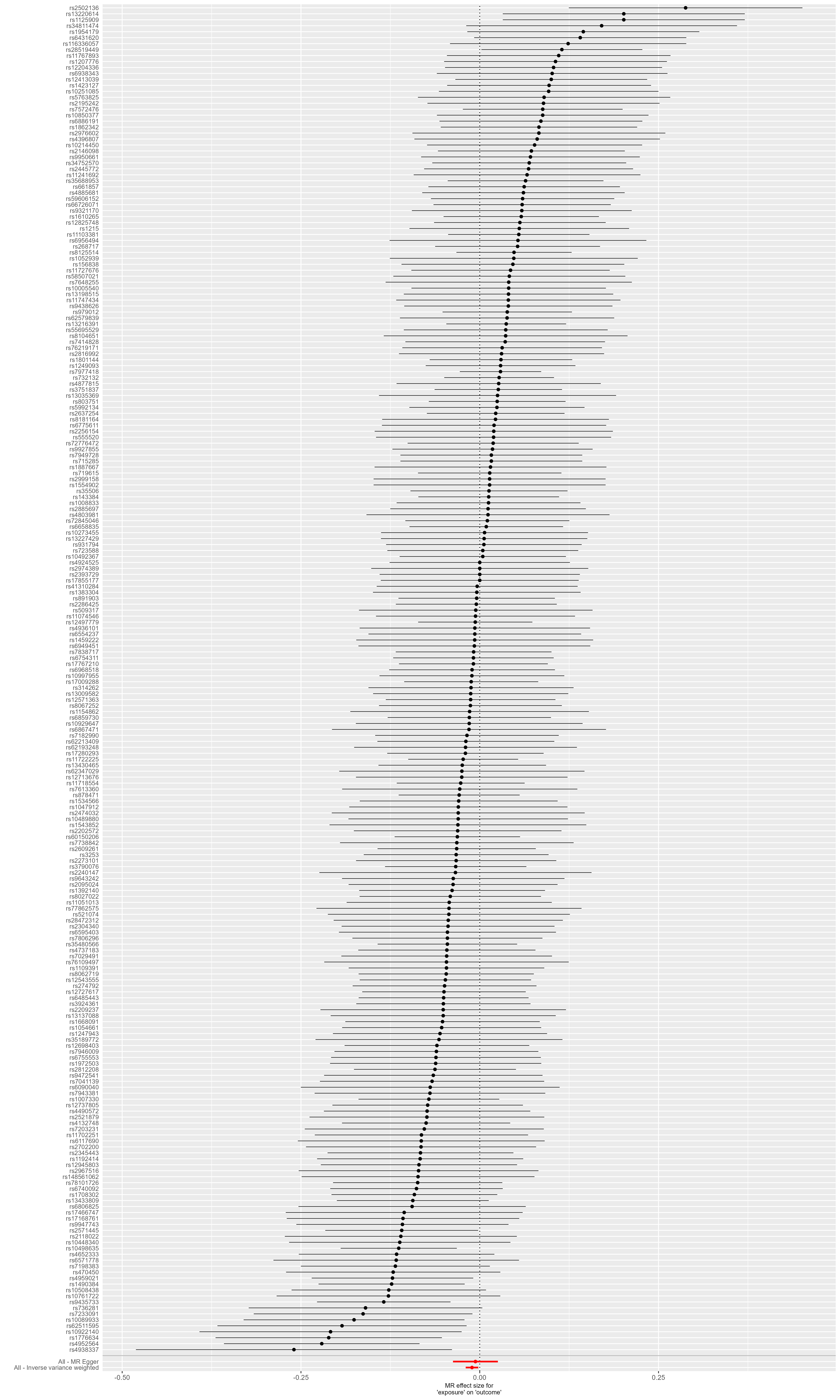

Supplement: Supplementary file 12 — Supplementary Material 12. [file 12890_2024_3150_MOESM12_ESM.zip › Supplementary Figure/Forest plot/Cortex Thickness/forest_plotFVC_parsorbitalis_thickavg.png]

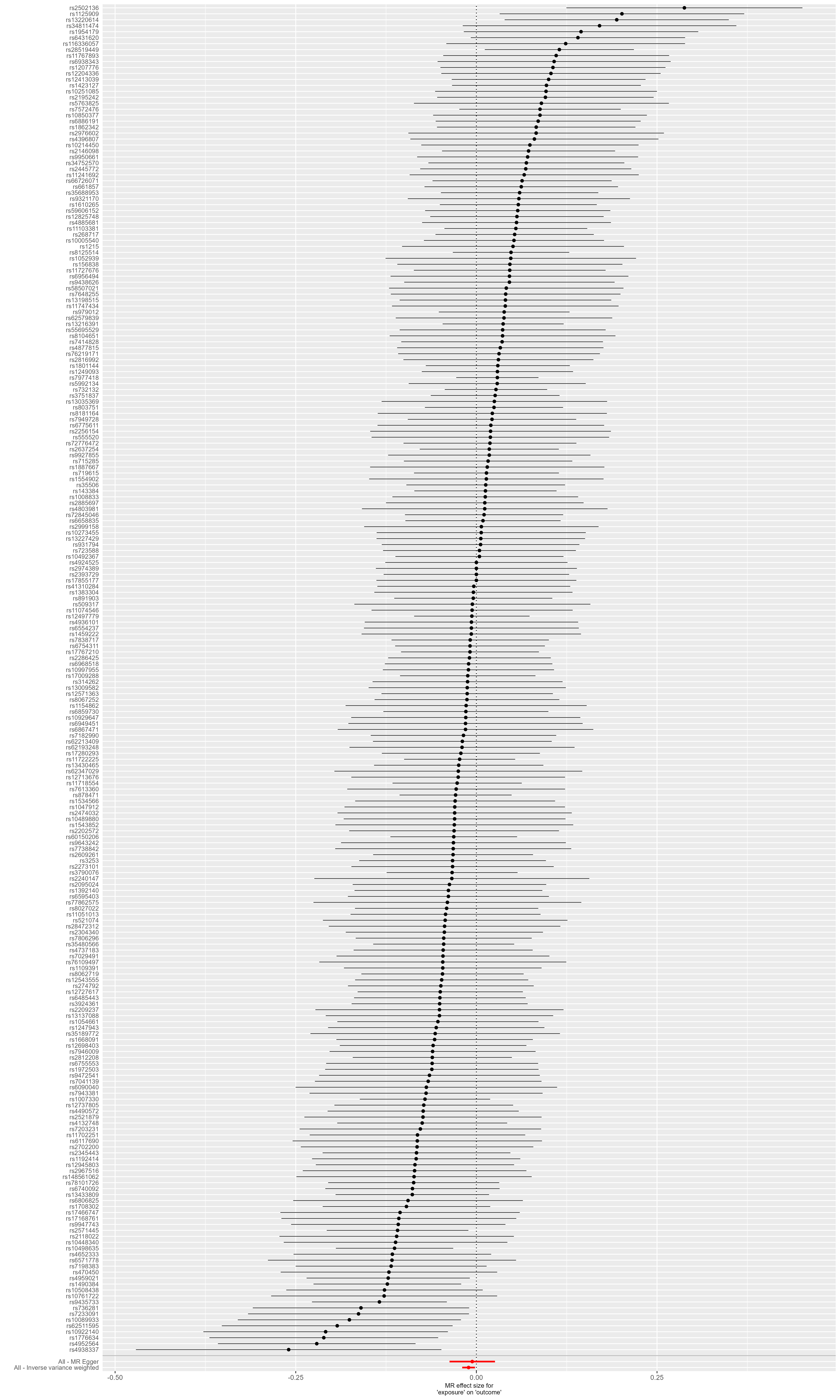

Supplement: Supplementary file 12 — Supplementary Material 12. [file 12890_2024_3150_MOESM12_ESM.zip › Supplementary Figure/Forest plot/Cortex Thickness/forest_plotFVC_parsorbitalis_thickavg_noGC.png]

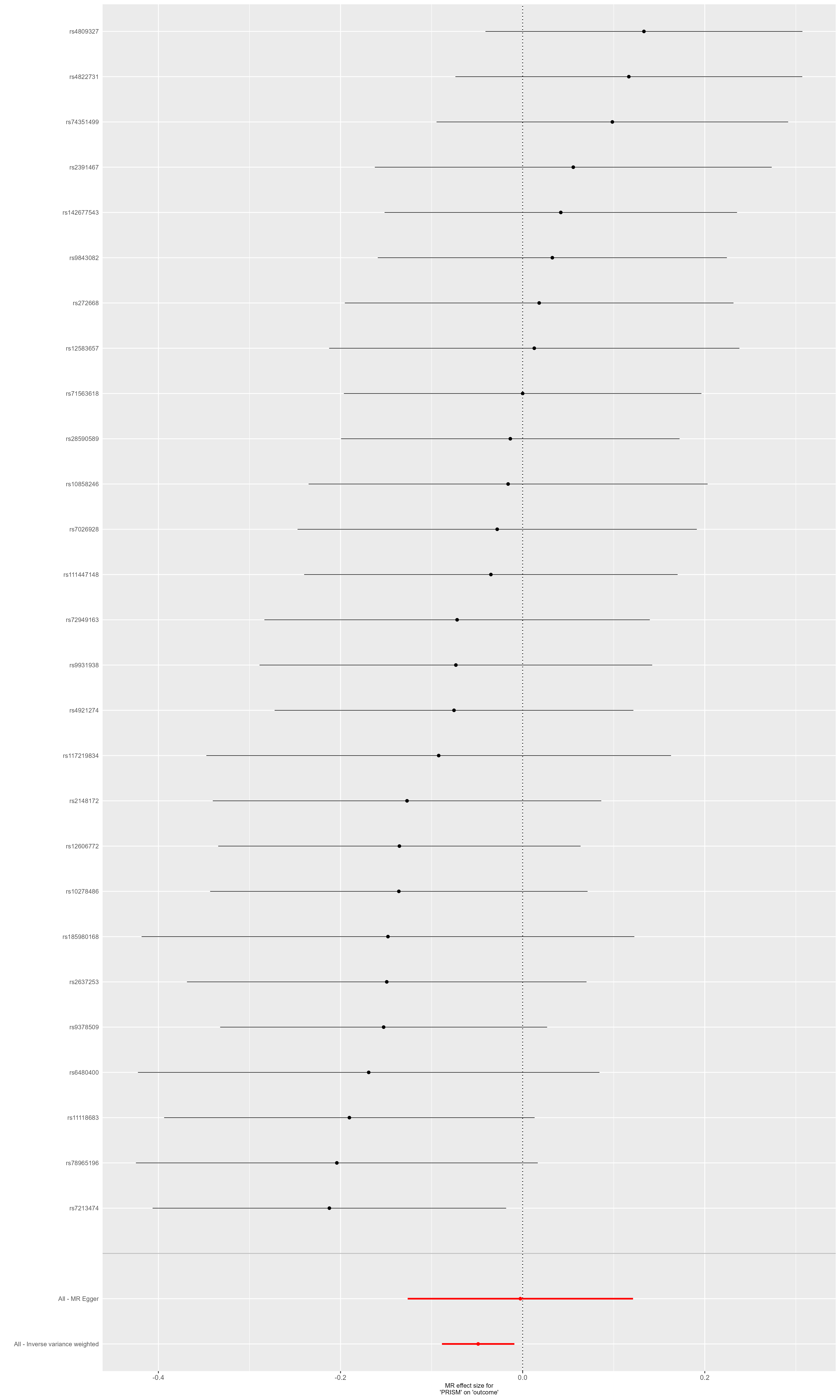

Supplement: Supplementary file 12 — Supplementary Material 12. [file 12890_2024_3150_MOESM12_ESM.zip › Supplementary Figure/Forest plot/Cortex Thickness/forest_plotPRISM_inferiorparietal_thickavg_GC.png]

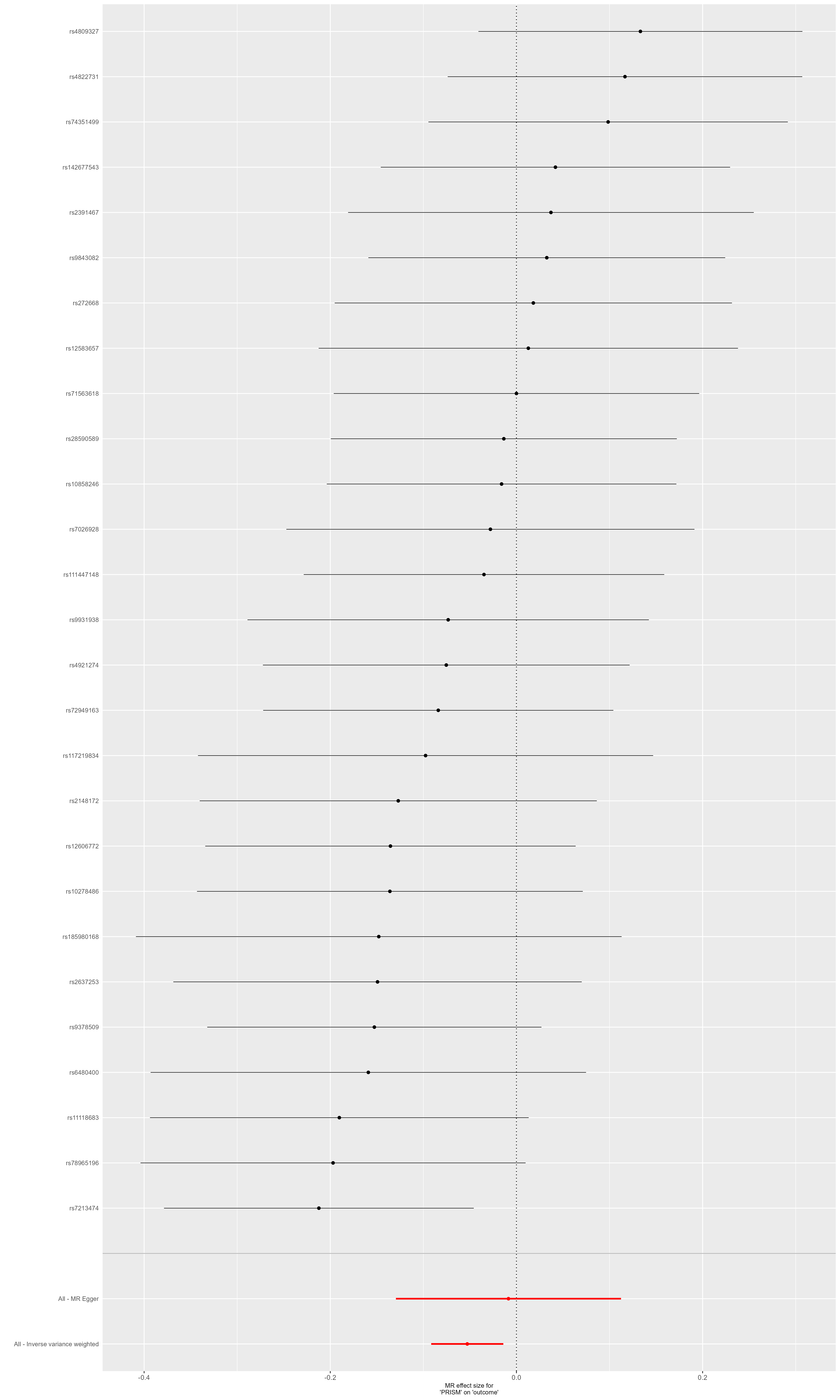

Supplement: Supplementary file 12 — Supplementary Material 12. [file 12890_2024_3150_MOESM12_ESM.zip › Supplementary Figure/Forest plot/Cortex Thickness/forest_plotPRISM_inferiorparietal_thickavg_noGC.png]

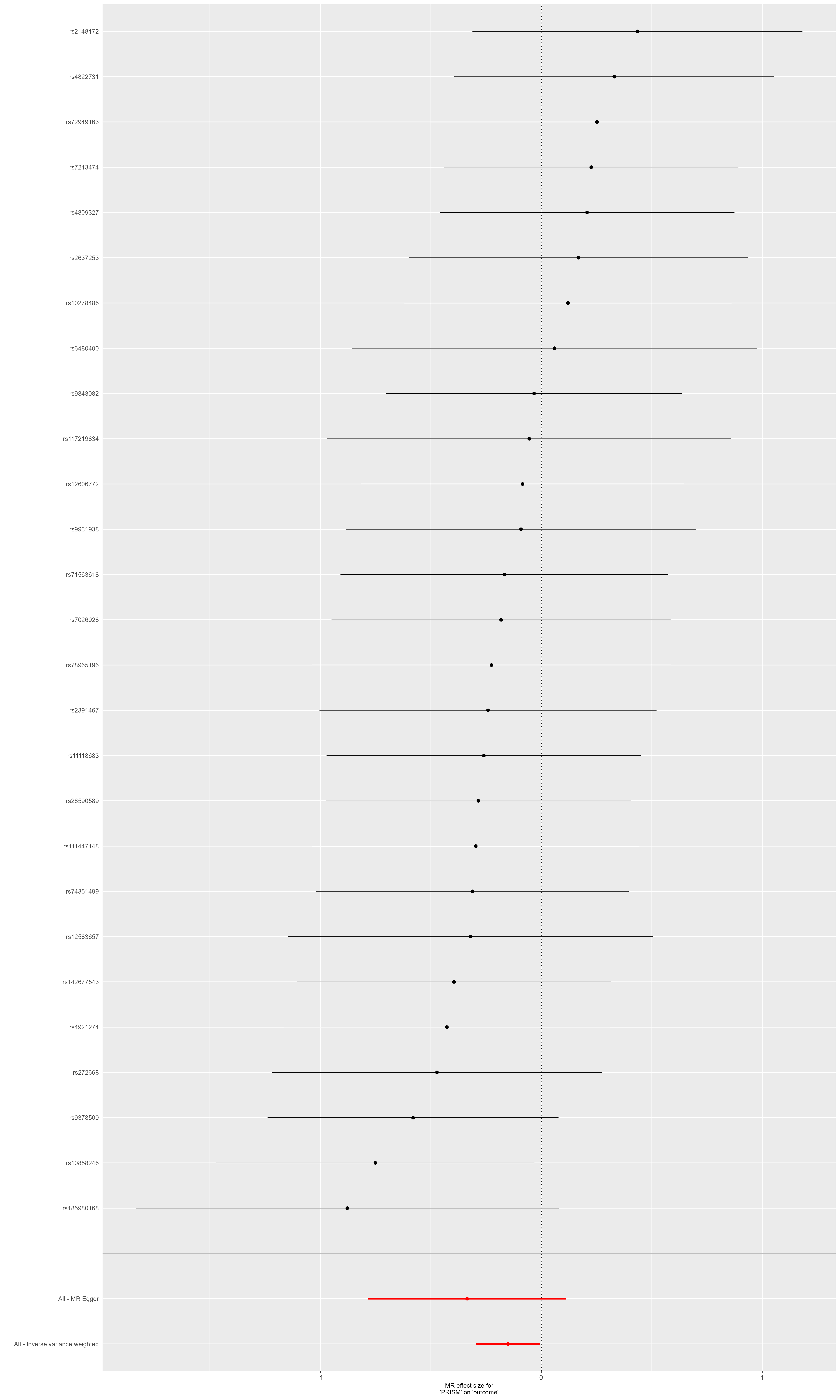

Supplement: Supplementary file 12 — Supplementary Material 12. [file 12890_2024_3150_MOESM12_ESM.zip › Supplementary Figure/Forest plot/Cortex Thickness/forest_plotPRISM_temporalpole_thickavg_GC.png]

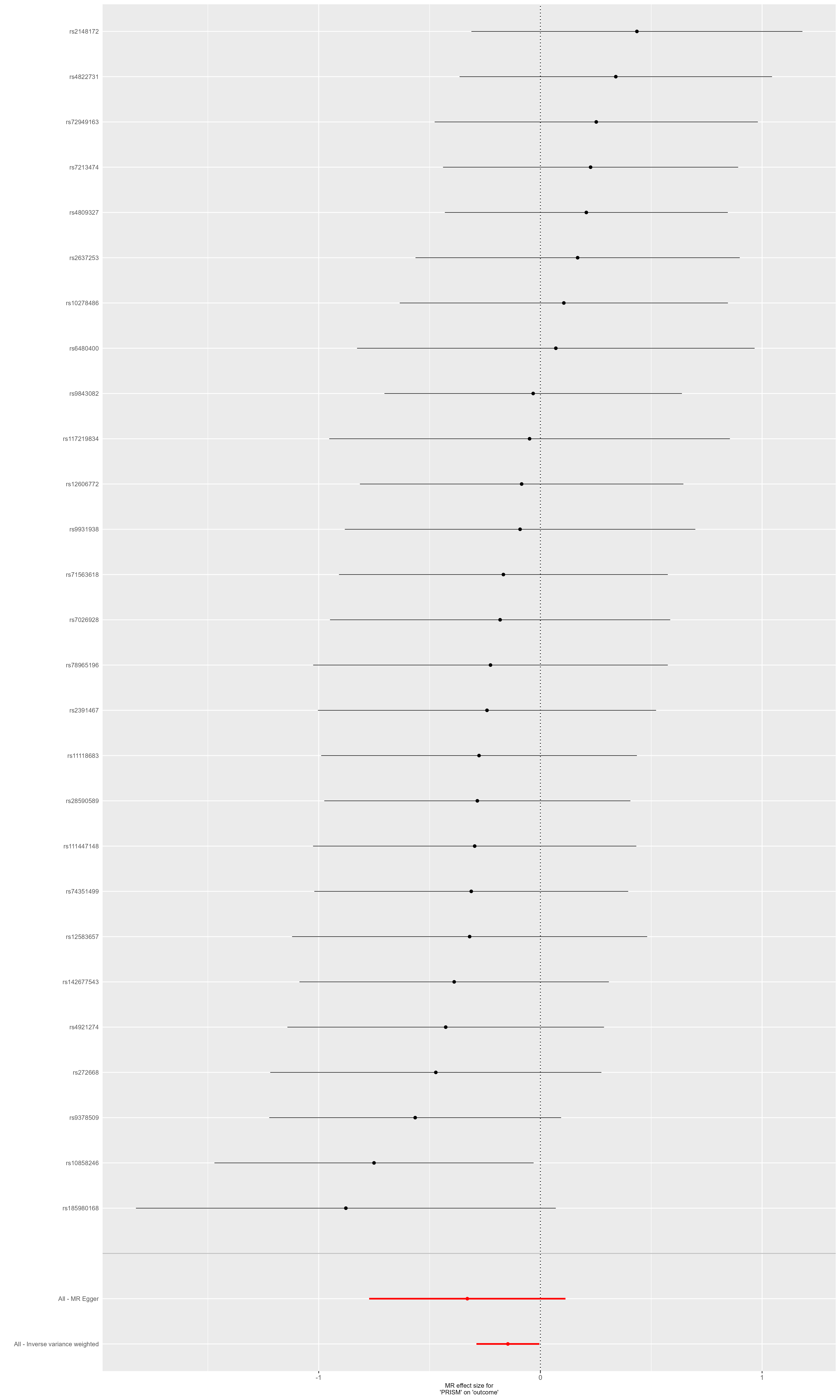

Supplement: Supplementary file 12 — Supplementary Material 12. [file 12890_2024_3150_MOESM12_ESM.zip › Supplementary Figure/Forest plot/Cortex Thickness/forest_plotPRISM_temporalpole_thickavg_noGC.png]

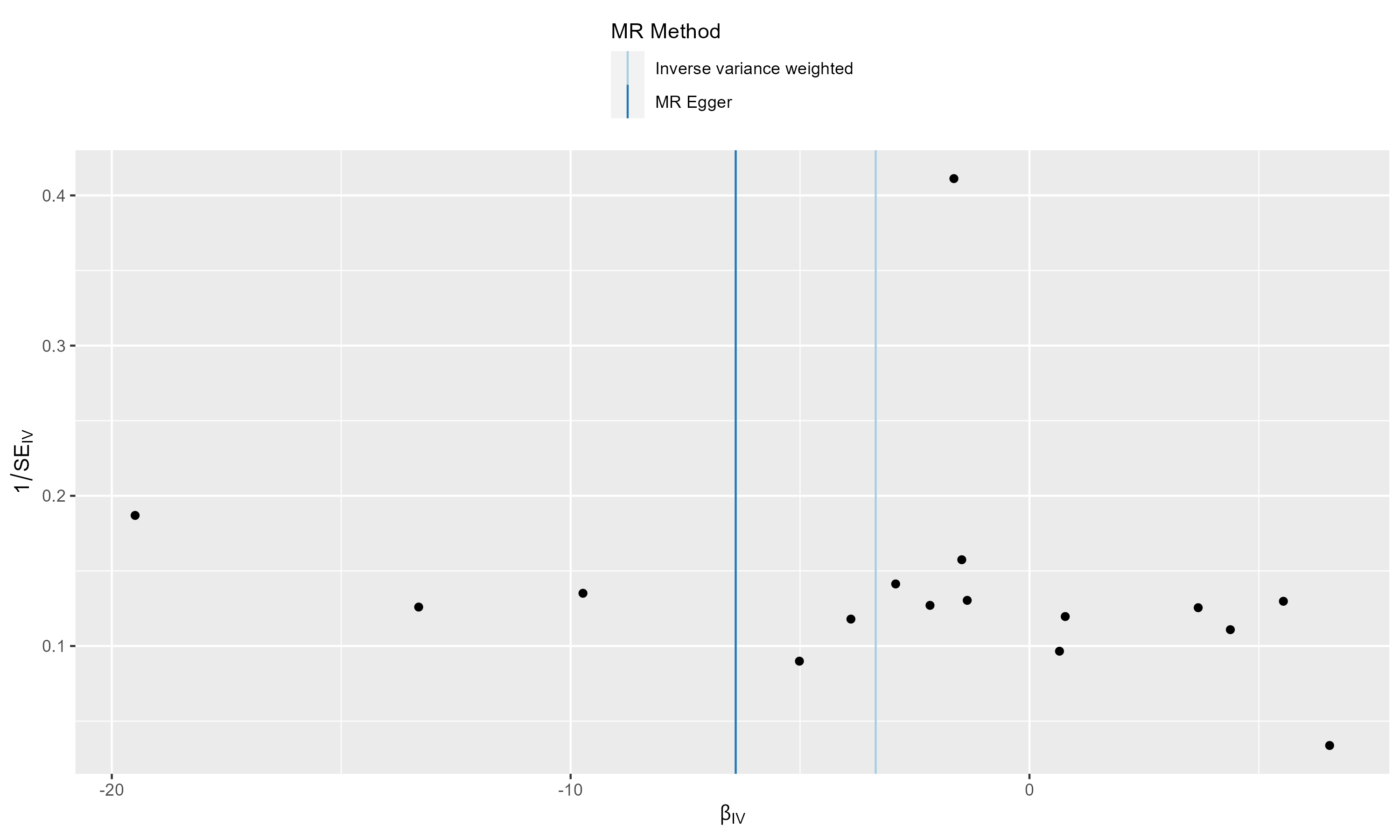

Supplement: Supplementary file 12 — Supplementary Material 12. [file 12890_2024_3150_MOESM12_ESM.zip › Supplementary Figure/funnel plot/Cortex Surface area/funnel_plotCOPD_parsorbitalis_surfavg.png]

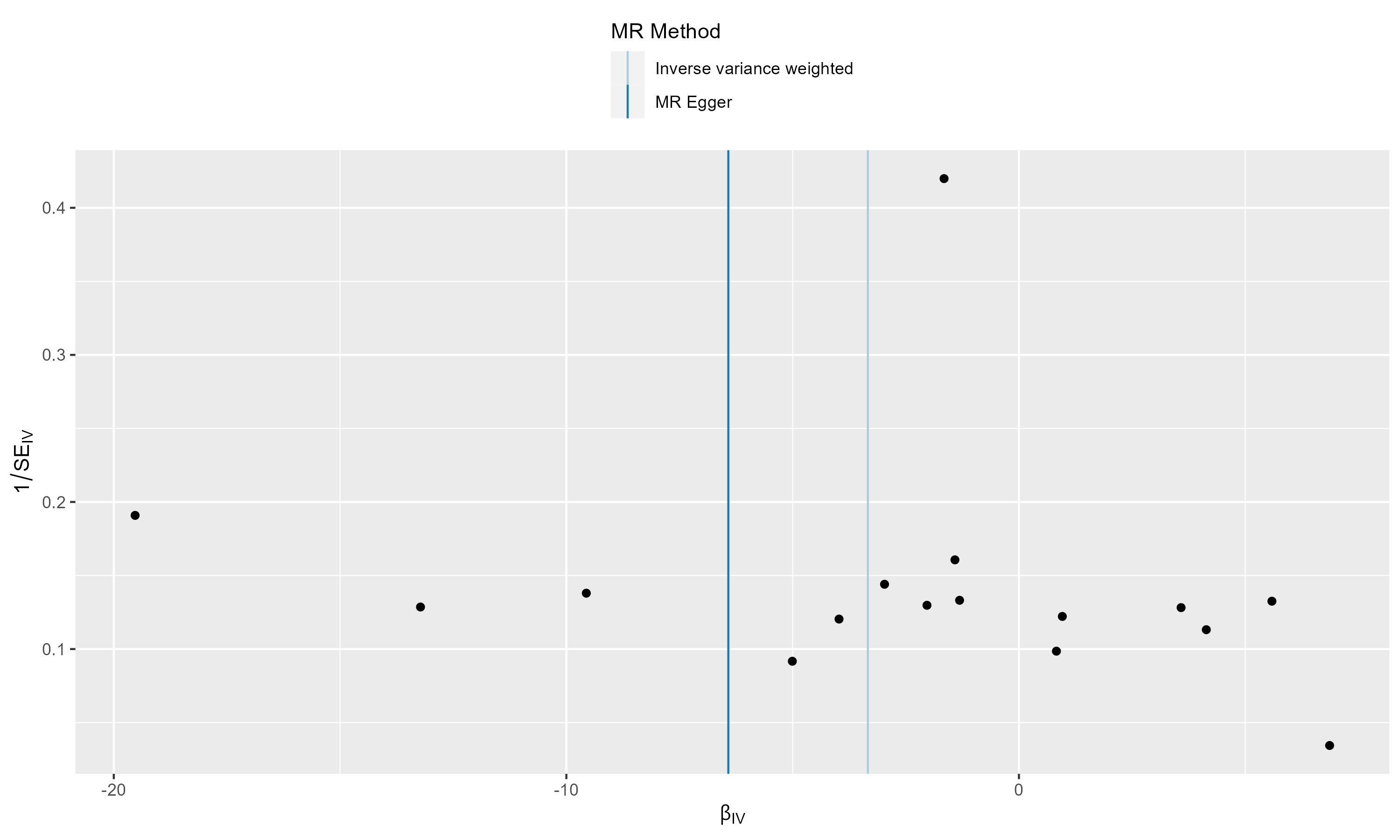

Supplement: Supplementary file 12 — Supplementary Material 12. [file 12890_2024_3150_MOESM12_ESM.zip › Supplementary Figure/funnel plot/Cortex Surface area/funnel_plotCOPD_parsorbitalis_surfavg_noGC.png]

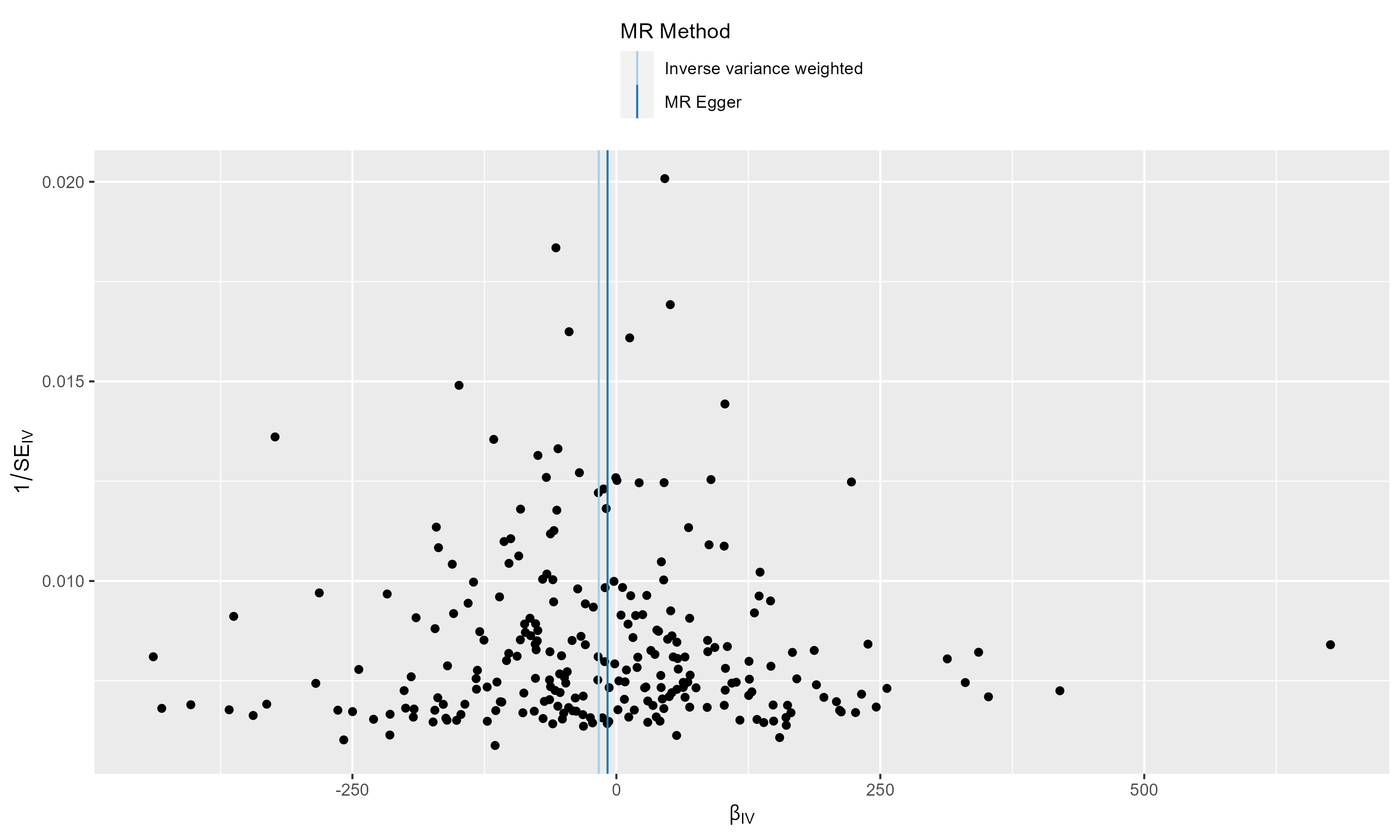

Supplement: Supplementary file 12 — Supplementary Material 12. [file 12890_2024_3150_MOESM12_ESM.zip › Supplementary Figure/funnel plot/Cortex Surface area/funnel_plotFEV1_caudalmiddlefrontal_surfavg.png]

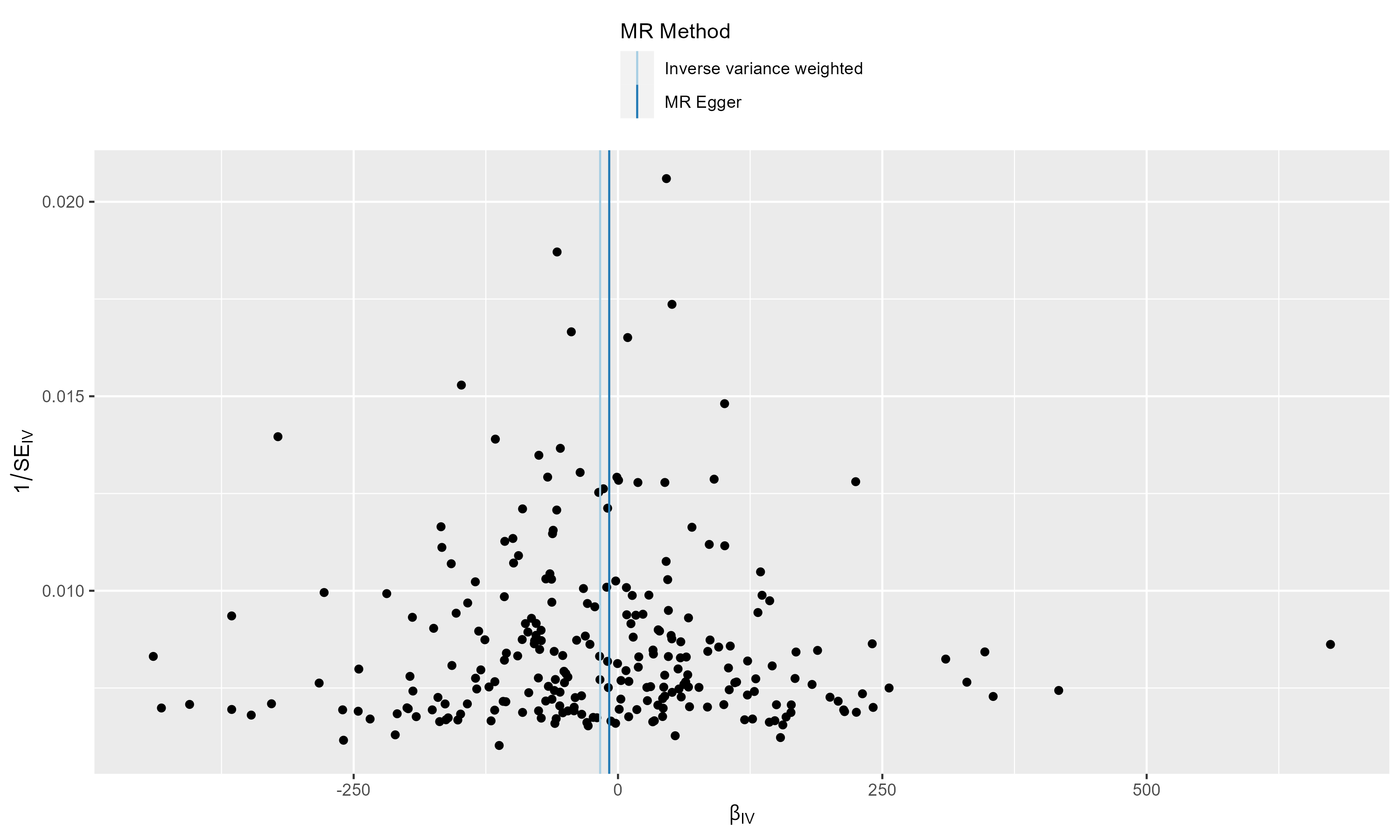

Supplement: Supplementary file 12 — Supplementary Material 12. [file 12890_2024_3150_MOESM12_ESM.zip › Supplementary Figure/funnel plot/Cortex Surface area/funnel_plotFEV1_caudalmiddlefrontal_surfavg_noGC.png]

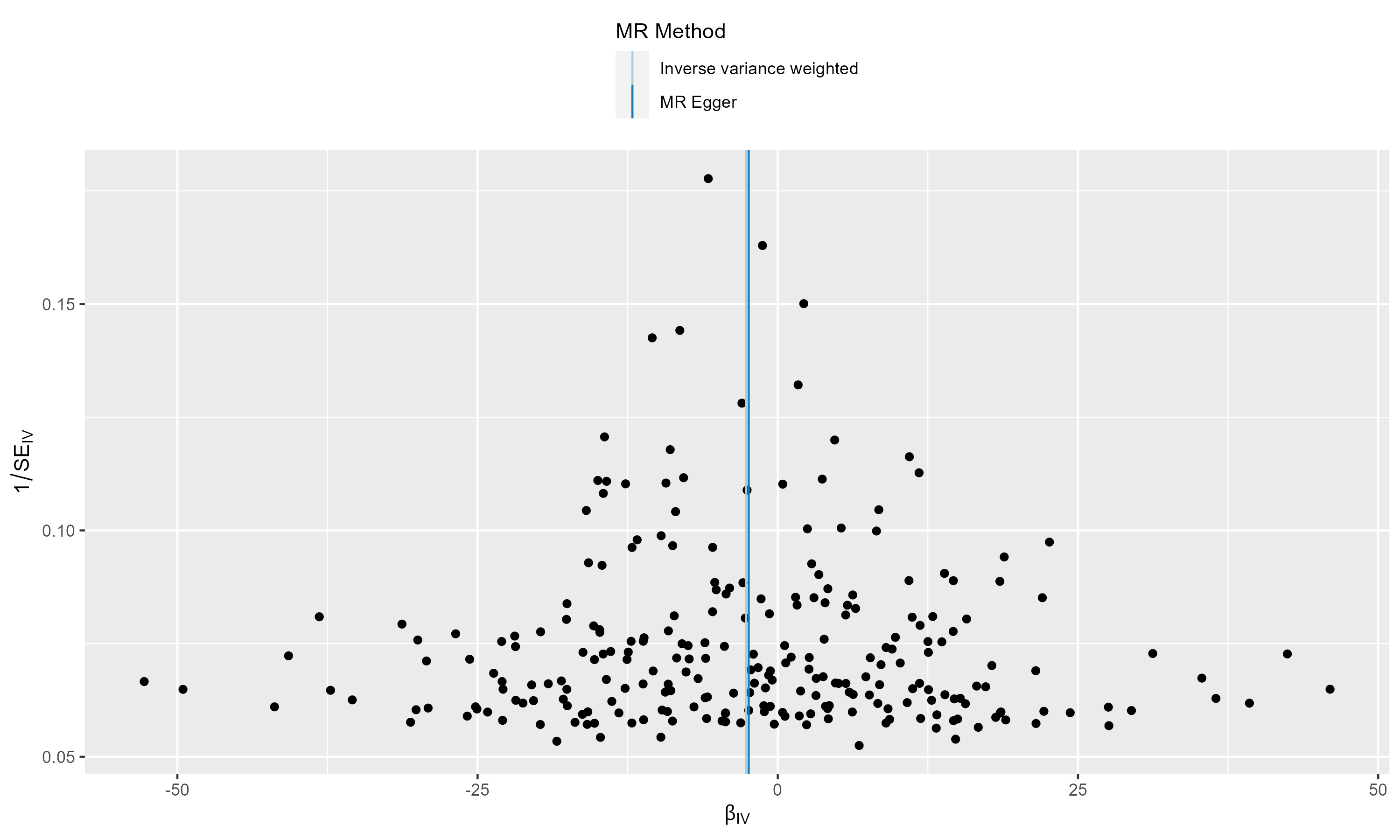

Supplement: Supplementary file 12 — Supplementary Material 12. [file 12890_2024_3150_MOESM12_ESM.zip › Supplementary Figure/funnel plot/Cortex Surface area/funnel_plotFEV1_frontalpole_surfavg.png]

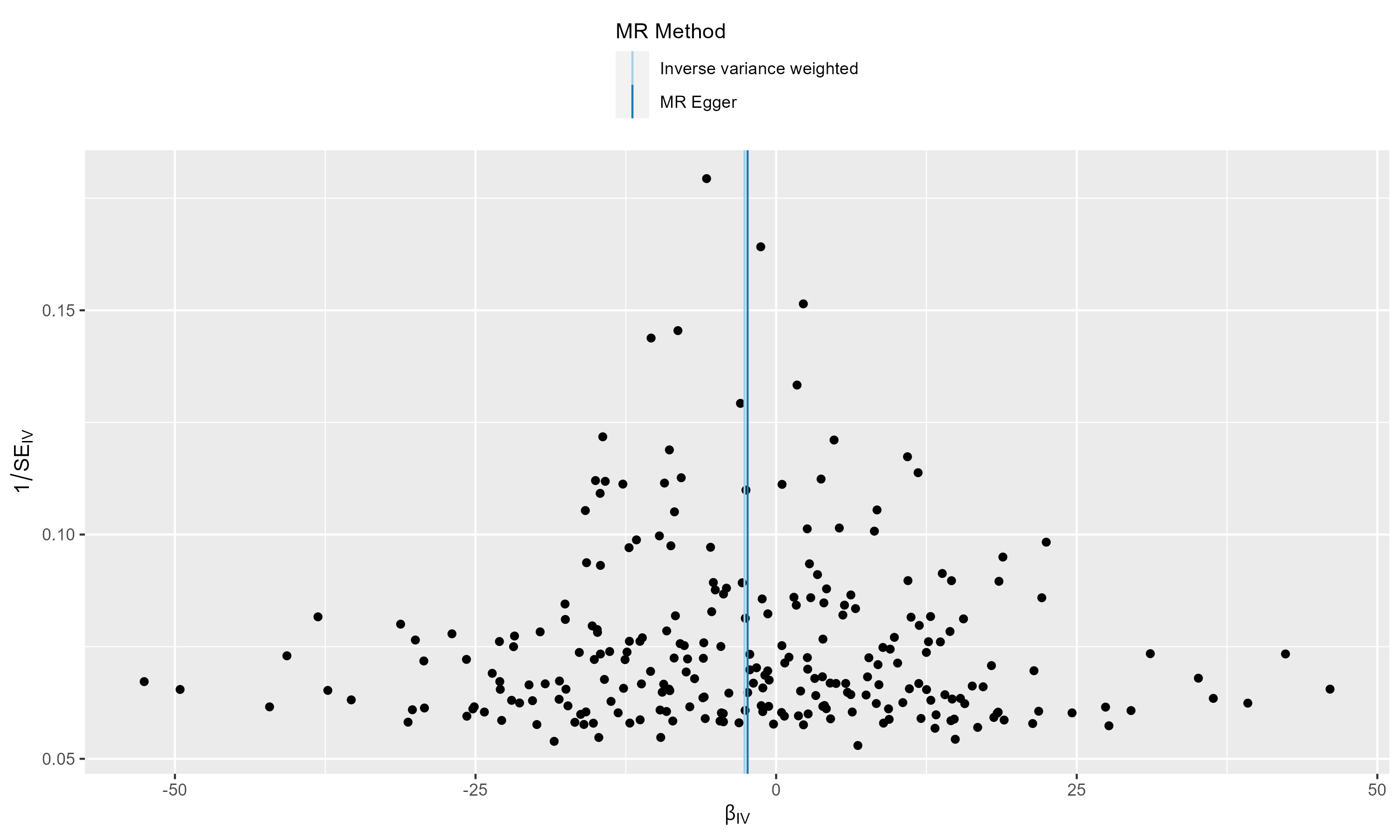

Supplement: Supplementary file 12 — Supplementary Material 12. [file 12890_2024_3150_MOESM12_ESM.zip › Supplementary Figure/funnel plot/Cortex Surface area/funnel_plotFEV1_frontalpole_surfavg_noGC.png]

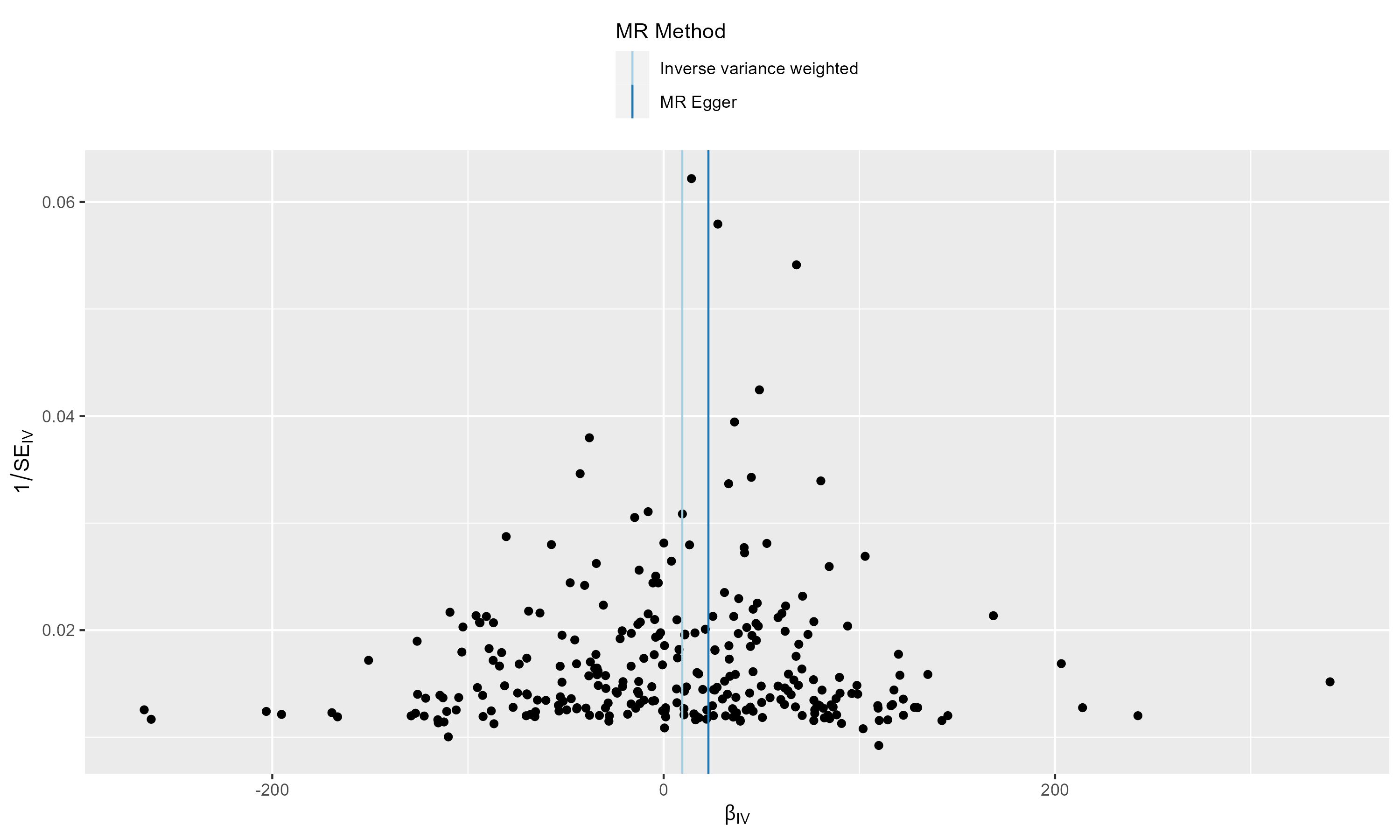

Supplement: Supplementary file 12 — Supplementary Material 12. [file 12890_2024_3150_MOESM12_ESM.zip › Supplementary Figure/funnel plot/Cortex Surface area/funnel_plotFEV1_FVC_paracentral_surfavg.png]

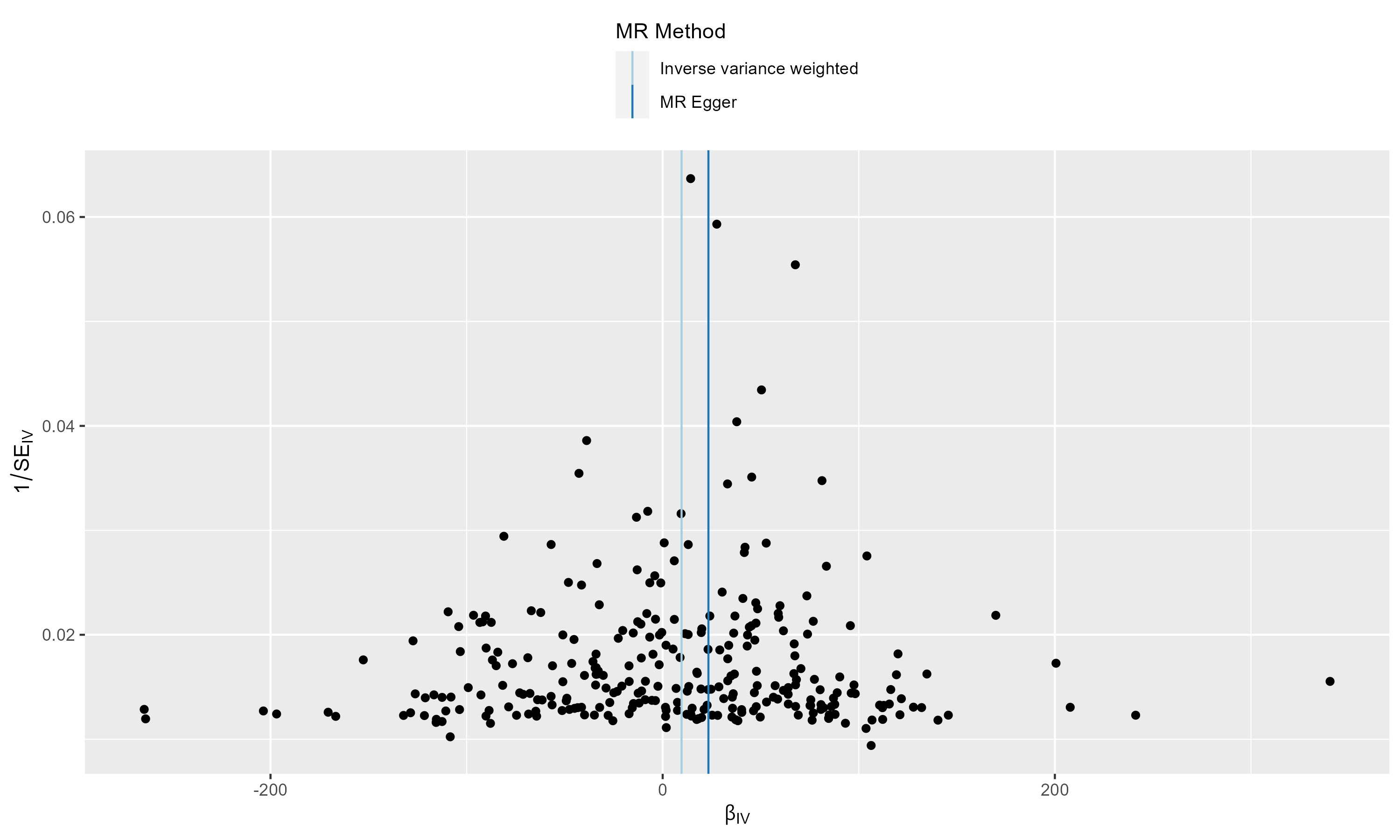

Supplement: Supplementary file 12 — Supplementary Material 12. [file 12890_2024_3150_MOESM12_ESM.zip › Supplementary Figure/funnel plot/Cortex Surface area/funnel_plotFEV1_FVC_paracentral_surfavg_noGC.png]

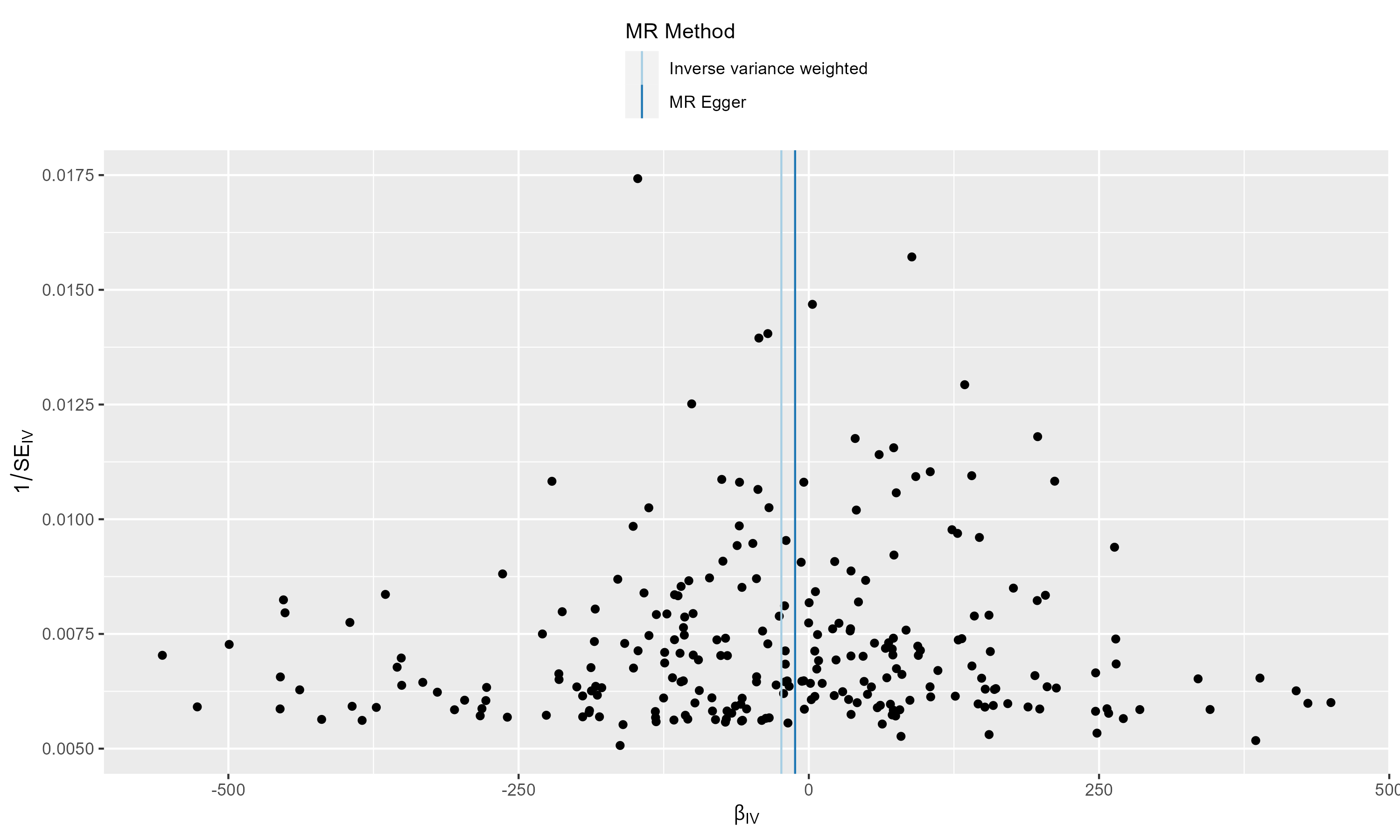

Supplement: Supplementary file 12 — Supplementary Material 12. [file 12890_2024_3150_MOESM12_ESM.zip › Supplementary Figure/funnel plot/Cortex Surface area/funnel_plotFEV1_lingual_surfavg.png]

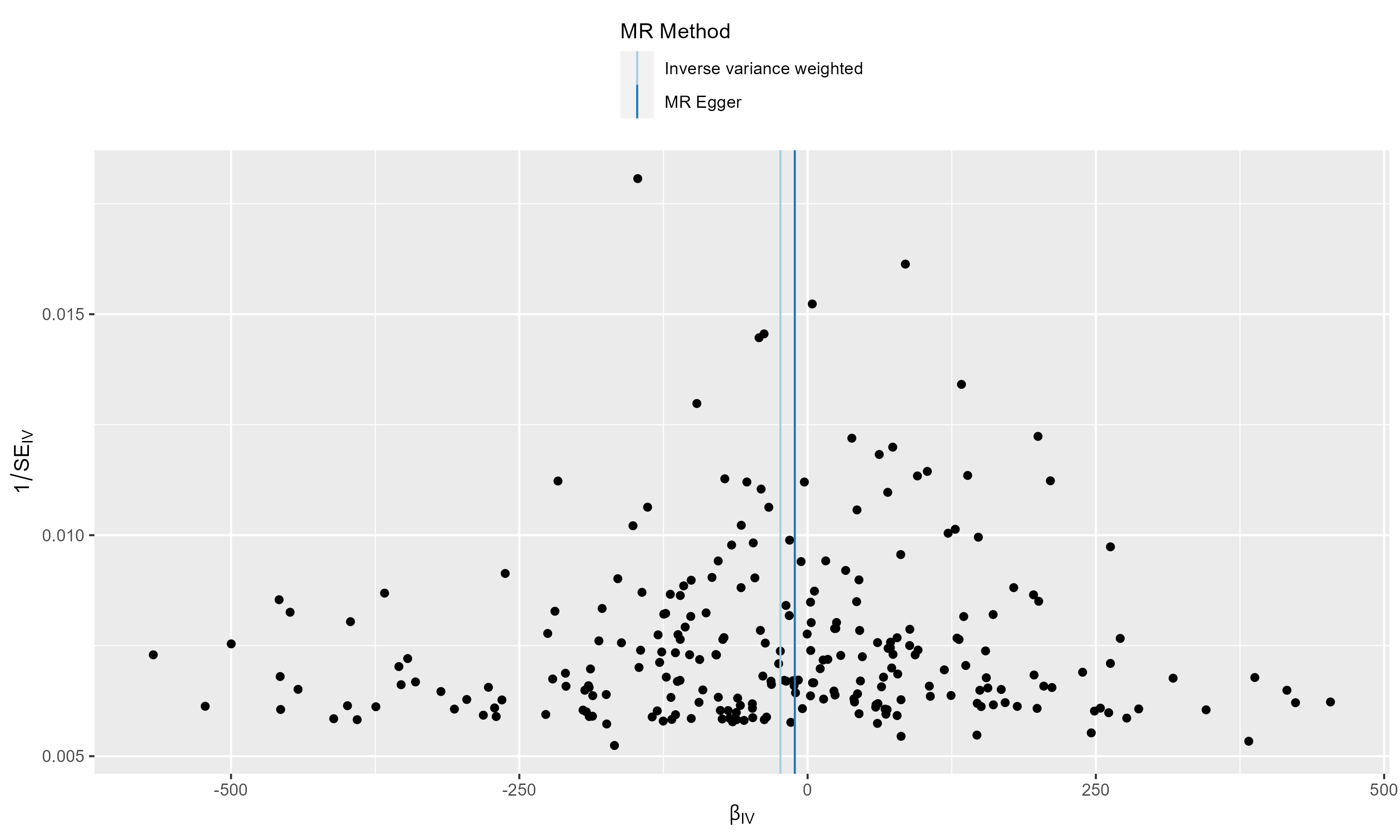

Supplement: Supplementary file 12 — Supplementary Material 12. [file 12890_2024_3150_MOESM12_ESM.zip › Supplementary Figure/funnel plot/Cortex Surface area/funnel_plotFEV1_lingual_surfavg_noGC.png]

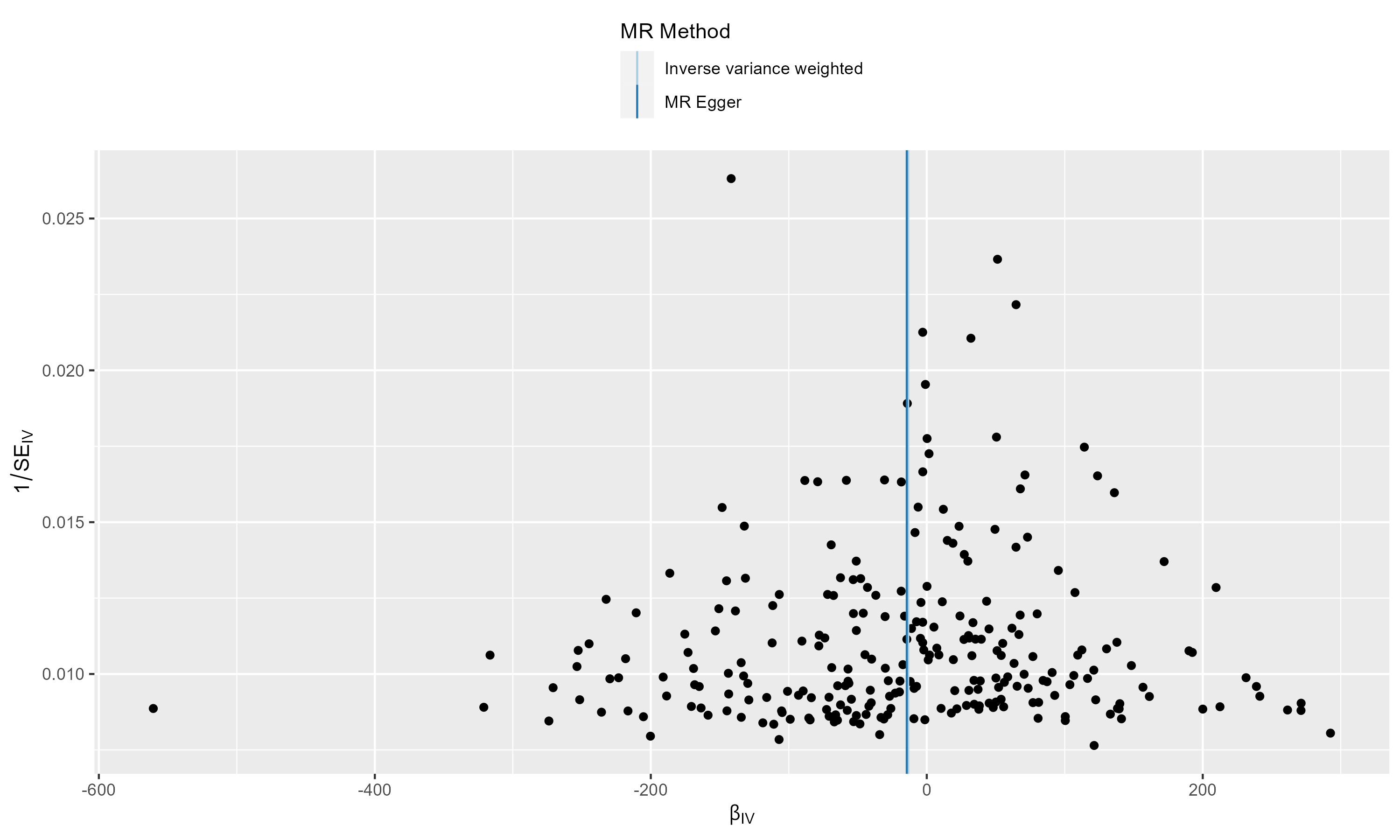

Supplement: Supplementary file 12 — Supplementary Material 12. [file 12890_2024_3150_MOESM12_ESM.zip › Supplementary Figure/funnel plot/Cortex Surface area/funnel_plotFEV1_pericalcarine_surfavg.png]

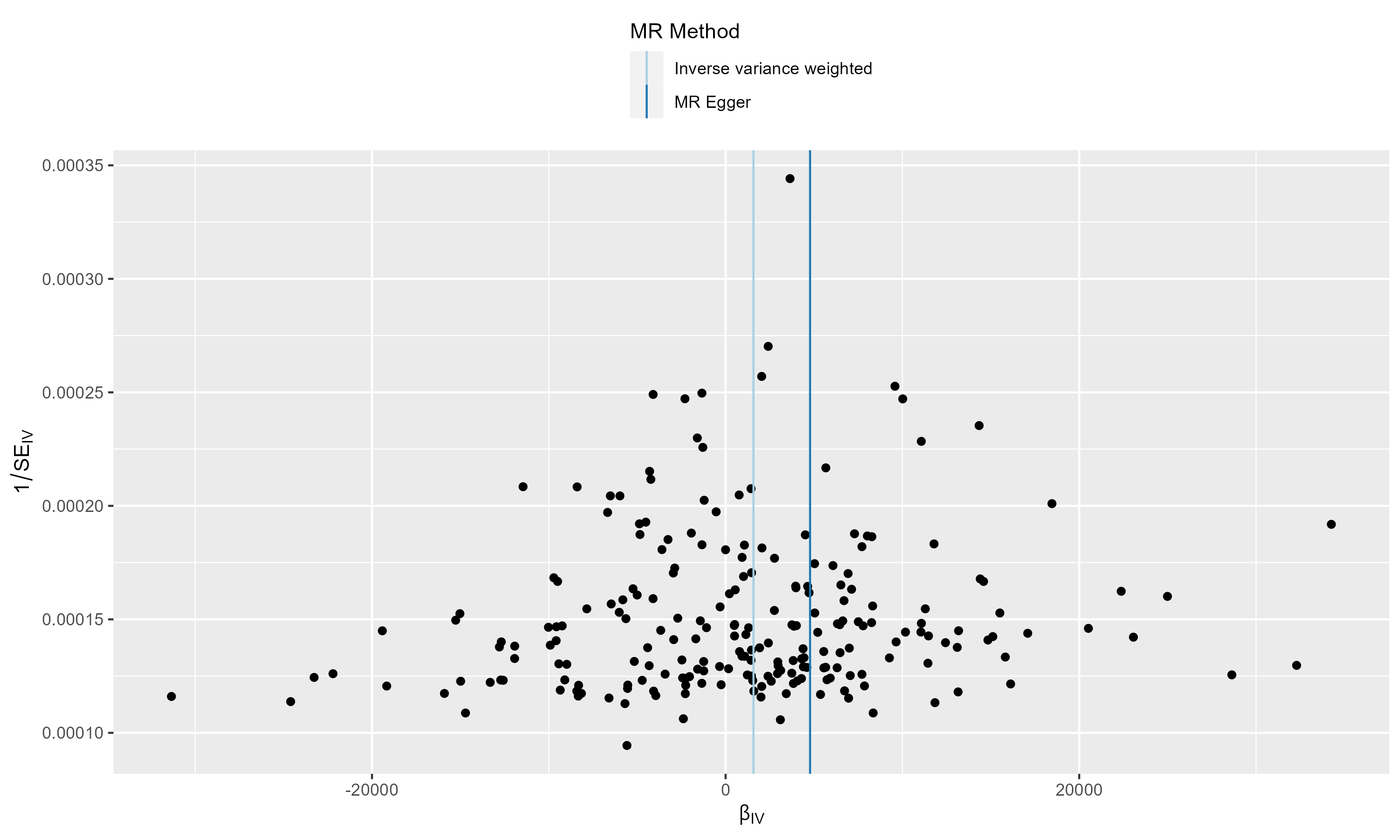

Supplement: Supplementary file 12 — Supplementary Material 12. [file 12890_2024_3150_MOESM12_ESM.zip › Supplementary Figure/funnel plot/Cortex Surface area/funnel_plotFVC_Full_SurfArea.png]

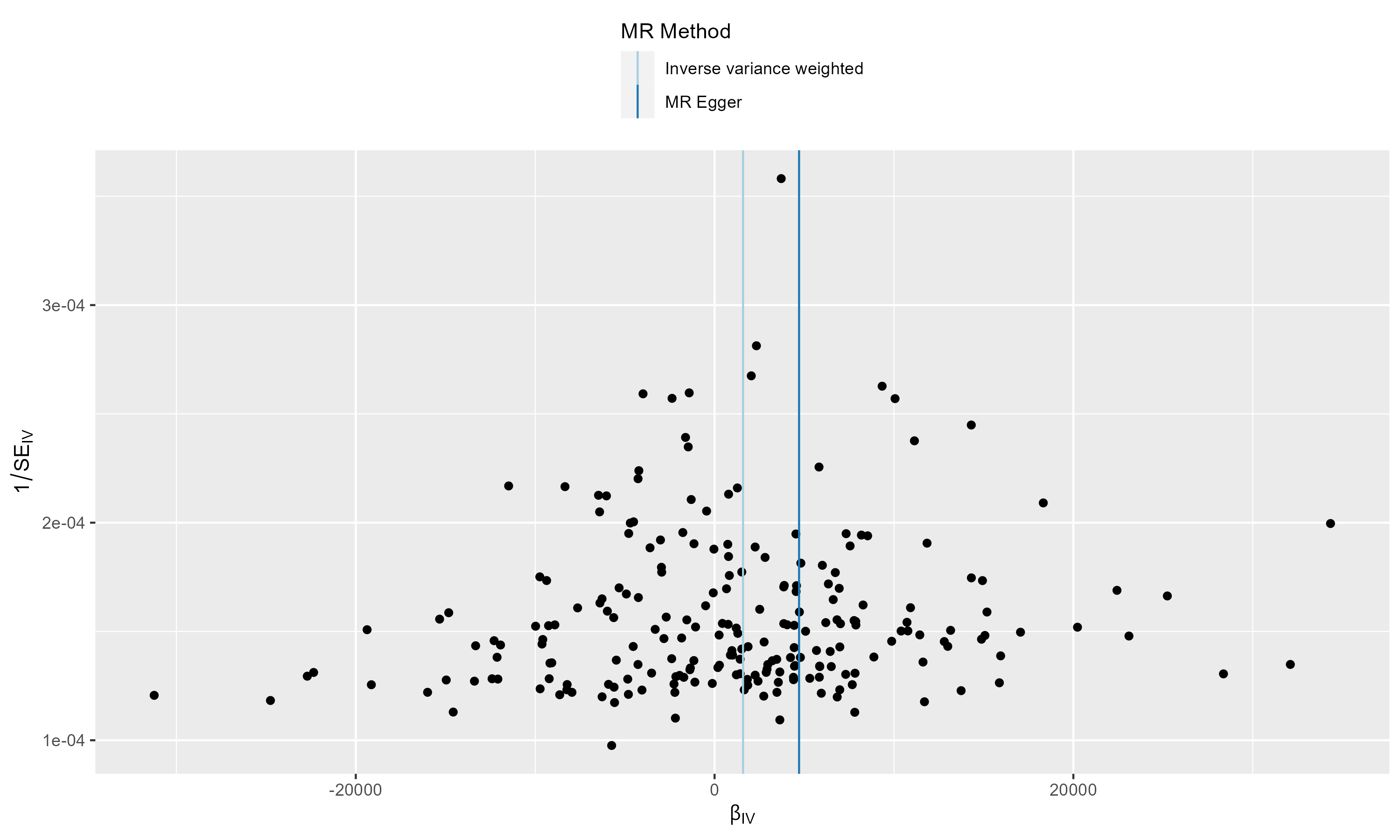

Supplement: Supplementary file 12 — Supplementary Material 12. [file 12890_2024_3150_MOESM12_ESM.zip › Supplementary Figure/funnel plot/Cortex Surface area/funnel_plotFVC_Full_SurfArea_noGC.png]

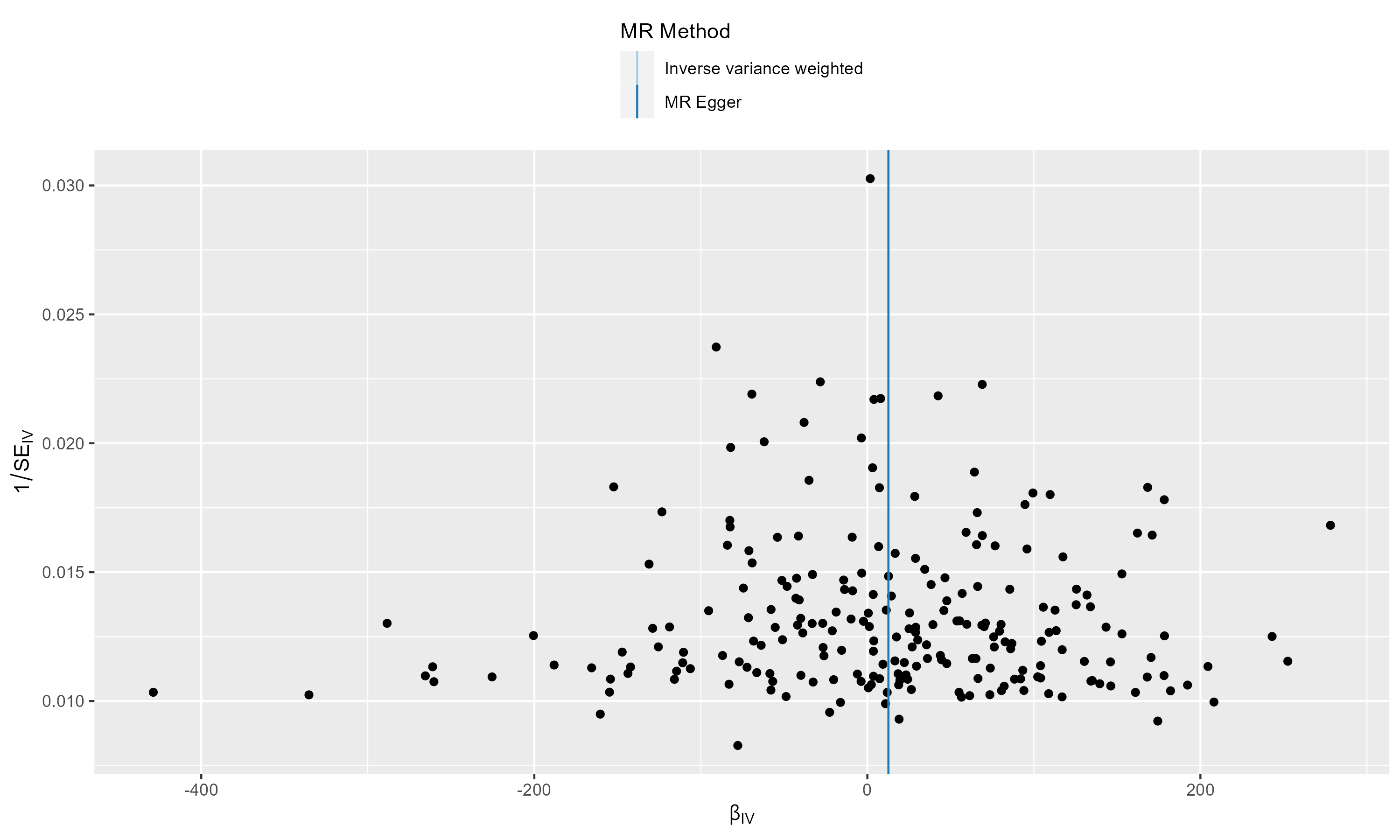

Supplement: Supplementary file 12 — Supplementary Material 12. [file 12890_2024_3150_MOESM12_ESM.zip › Supplementary Figure/funnel plot/Cortex Surface area/funnel_plotFVC_insula_surfavg_noGC.png]

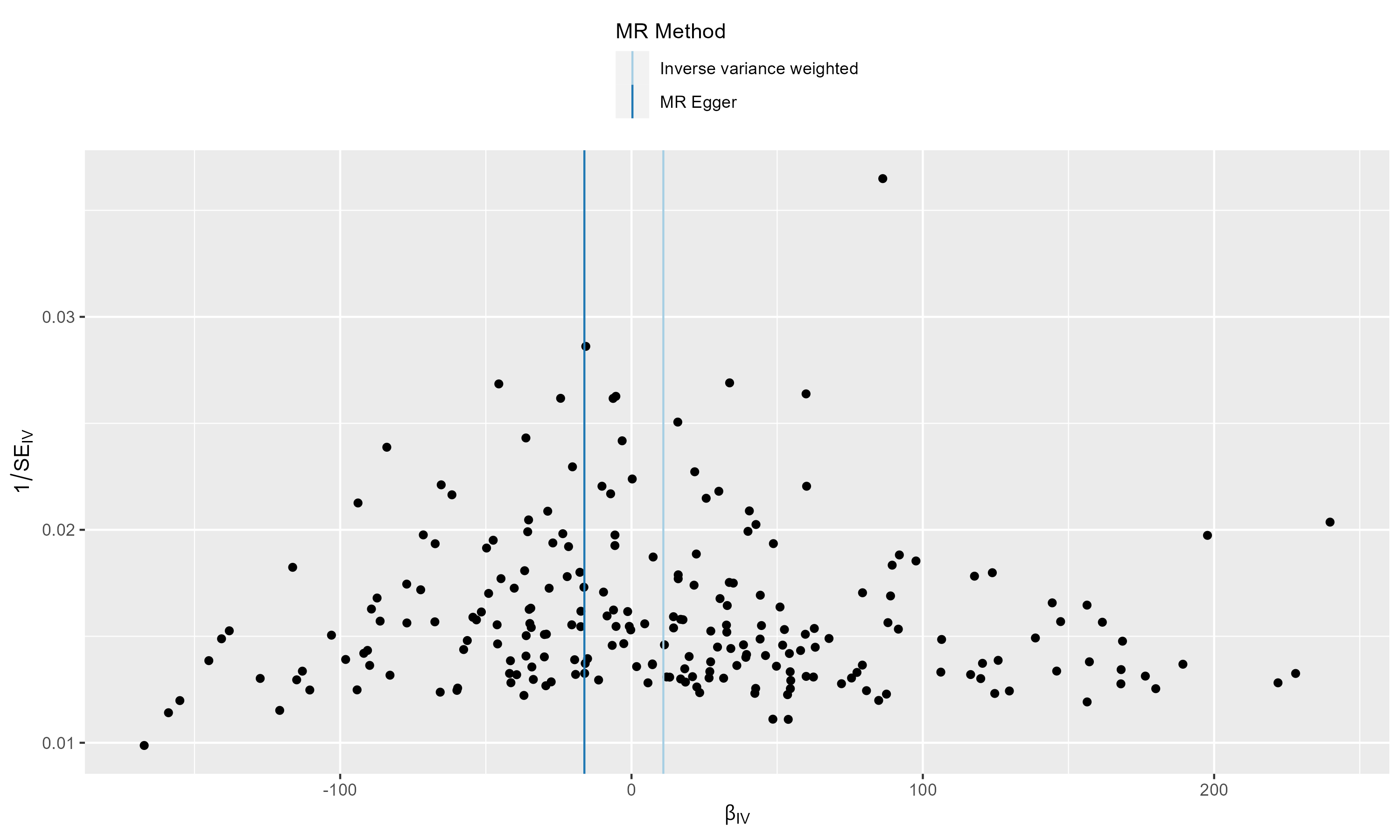

Supplement: Supplementary file 12 — Supplementary Material 12. [file 12890_2024_3150_MOESM12_ESM.zip › Supplementary Figure/funnel plot/Cortex Surface area/funnel_plotFVC_medialorbitofrontal_surfavg.png]

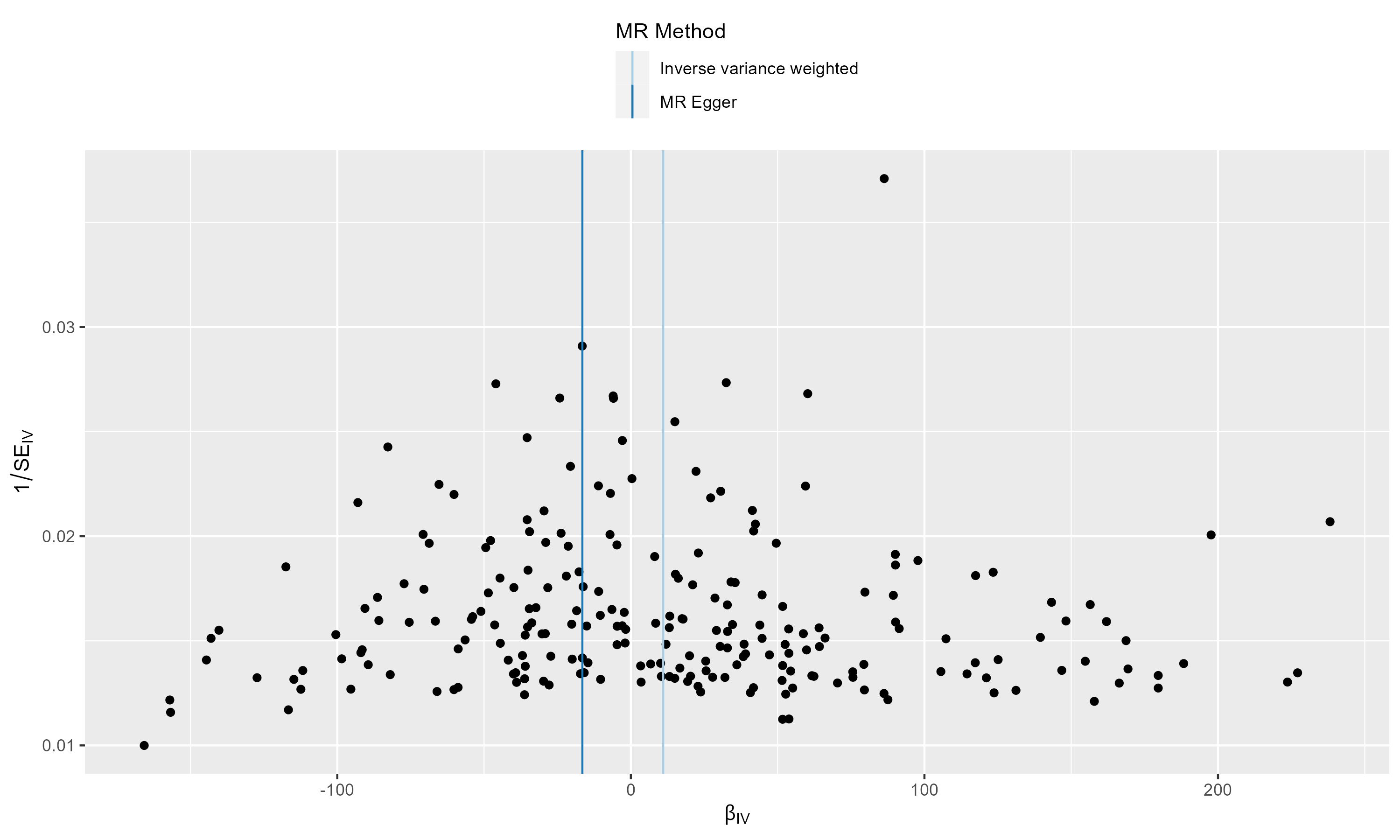

Supplement: Supplementary file 12 — Supplementary Material 12. [file 12890_2024_3150_MOESM12_ESM.zip › Supplementary Figure/funnel plot/Cortex Surface area/funnel_plotFVC_medialorbitofrontal_surfavg_noGC.png]

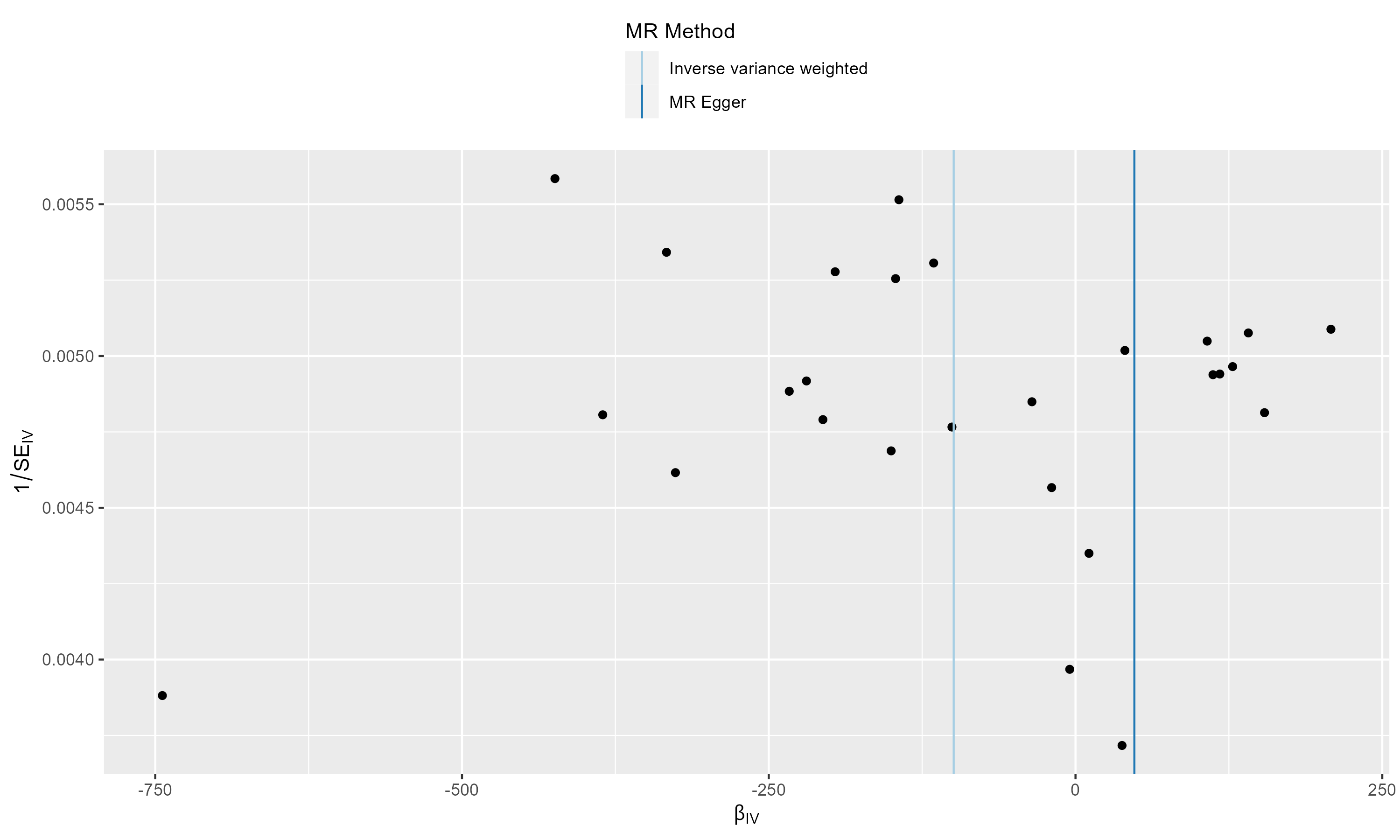

Supplement: Supplementary file 12 — Supplementary Material 12. [file 12890_2024_3150_MOESM12_ESM.zip › Supplementary Figure/funnel plot/Cortex Surface area/funnel_plotPRISM_paracentral_surfavg_GC.png]

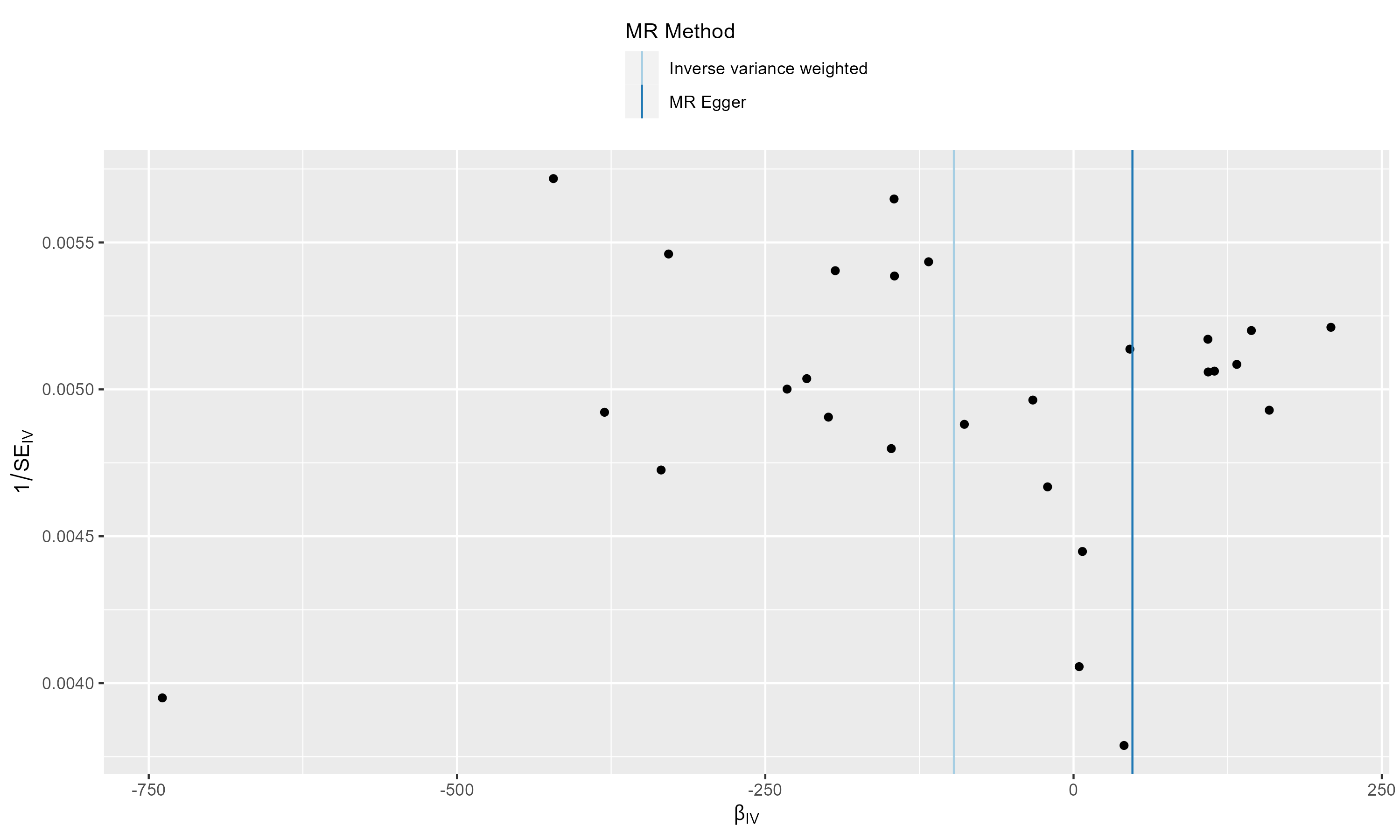

Supplement: Supplementary file 12 — Supplementary Material 12. [file 12890_2024_3150_MOESM12_ESM.zip › Supplementary Figure/funnel plot/Cortex Surface area/funnel_plotPRISM_paracentral_surfavg_noGC.png]

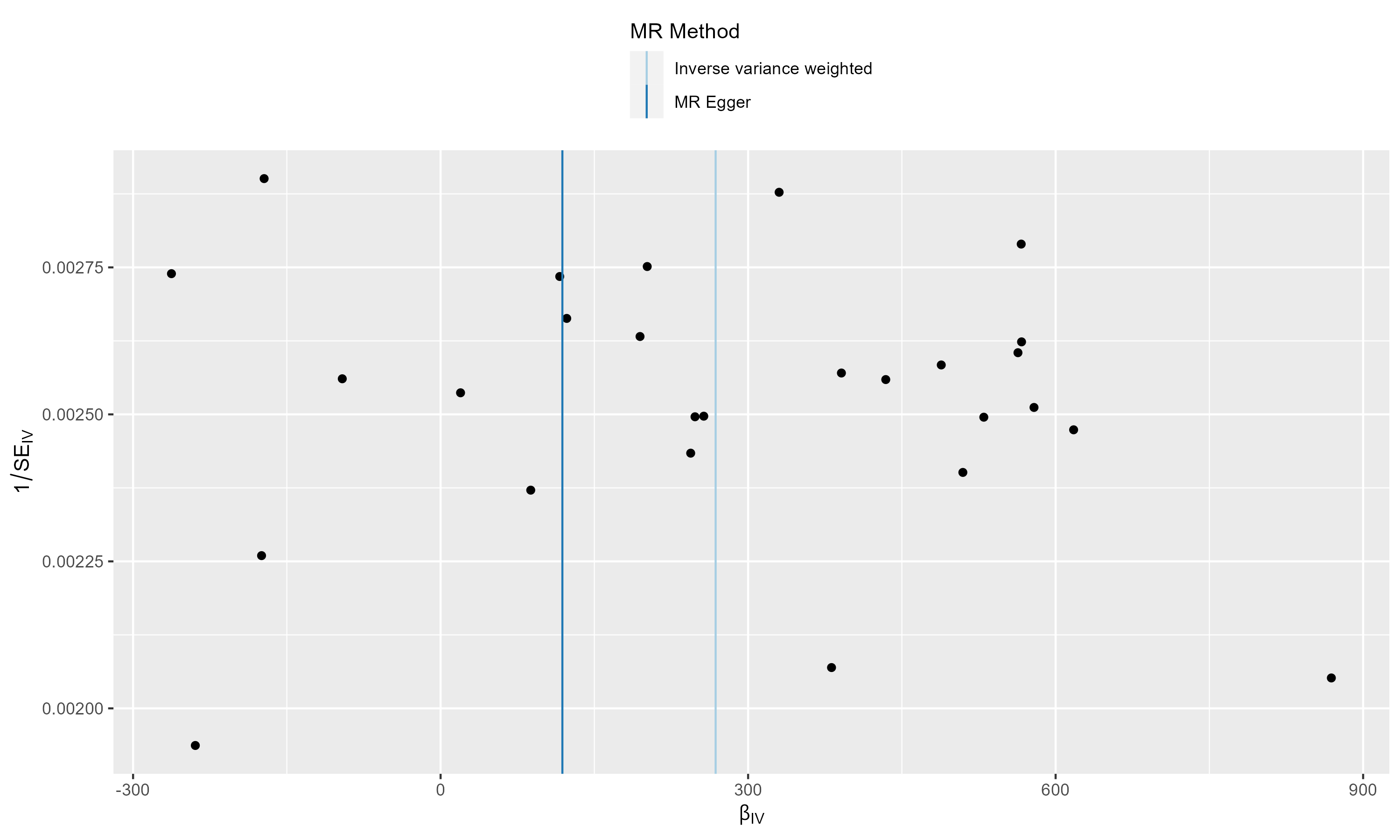

Supplement: Supplementary file 12 — Supplementary Material 12. [file 12890_2024_3150_MOESM12_ESM.zip › Supplementary Figure/funnel plot/Cortex Surface area/funnel_plotPRISM_precuneus_surfavg_GC.png]

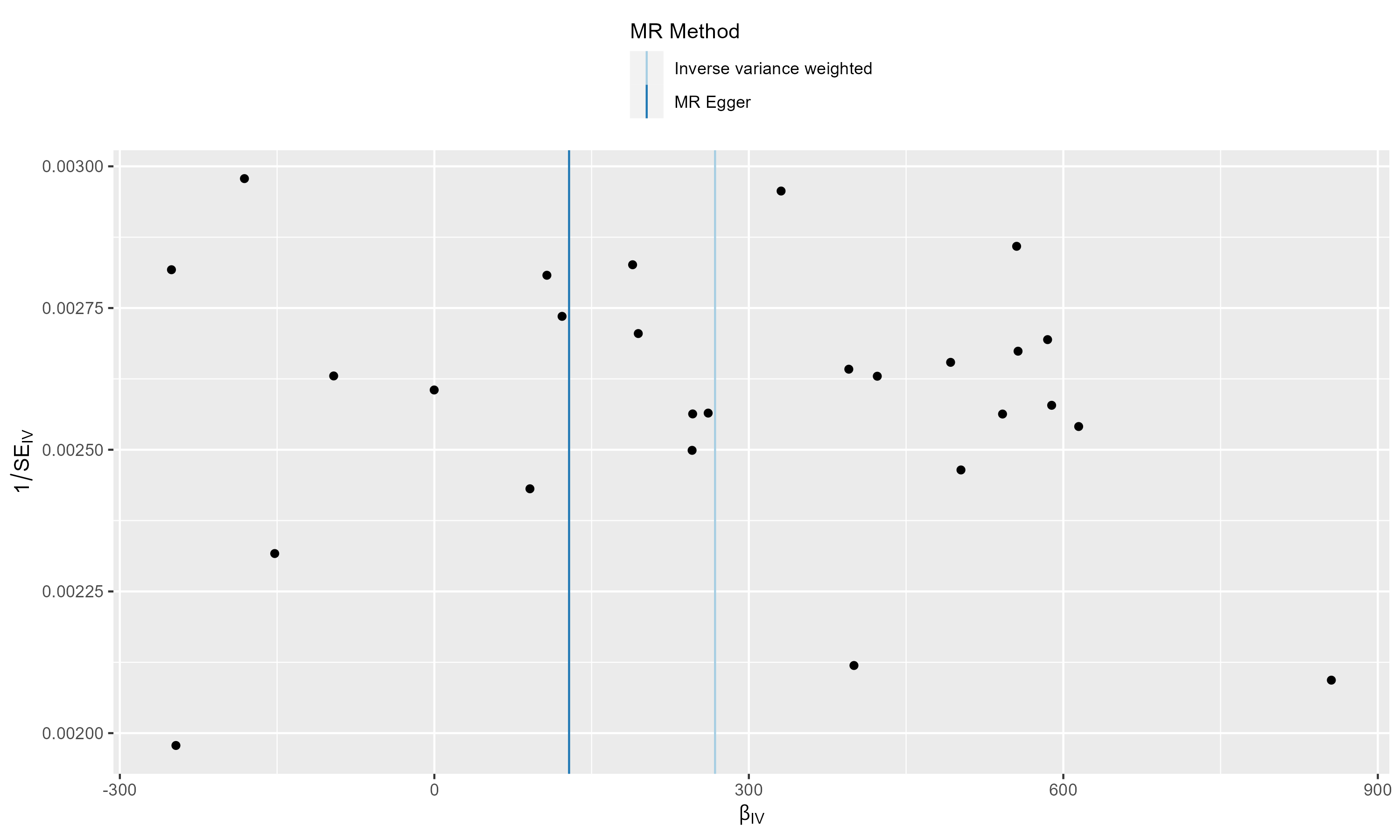

Supplement: Supplementary file 12 — Supplementary Material 12. [file 12890_2024_3150_MOESM12_ESM.zip › Supplementary Figure/funnel plot/Cortex Surface area/funnel_plotPRISM_precuneus_surfavg_noGC.png]

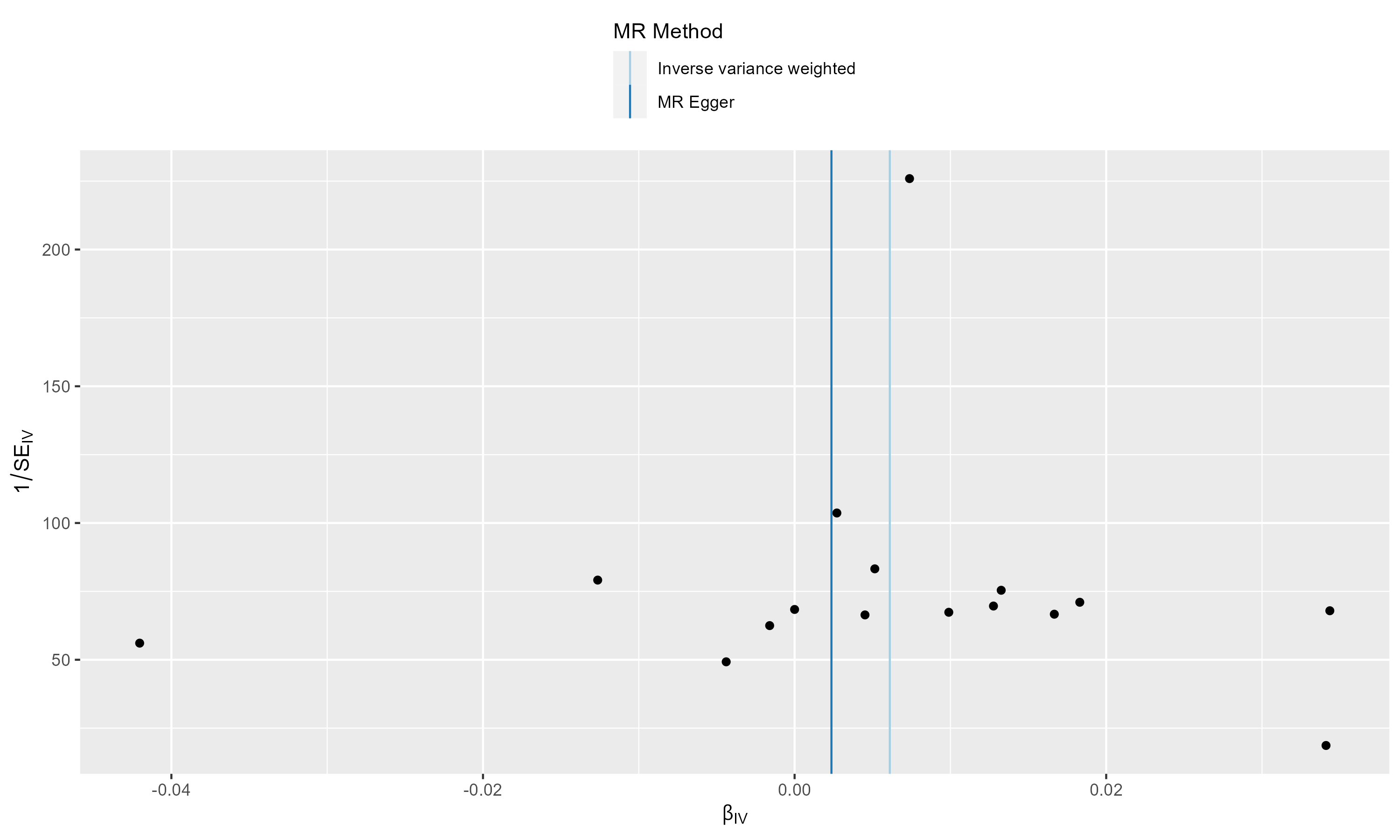

Supplement: Supplementary file 12 — Supplementary Material 12. [file 12890_2024_3150_MOESM12_ESM.zip › Supplementary Figure/funnel plot/Cortex Thickness/funnel_plotCOPD_cuneus_thickavg.png]

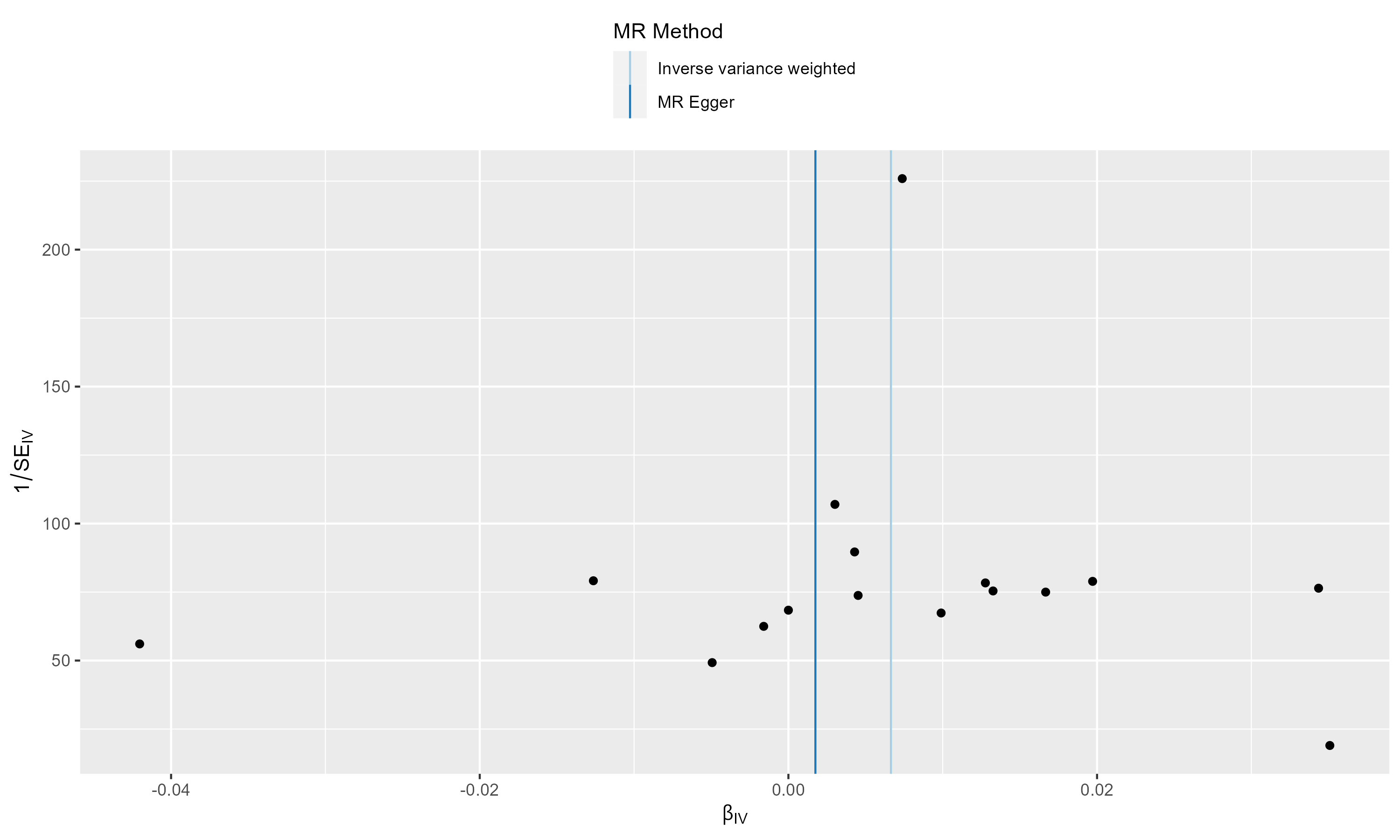

Supplement: Supplementary file 12 — Supplementary Material 12. [file 12890_2024_3150_MOESM12_ESM.zip › Supplementary Figure/funnel plot/Cortex Thickness/funnel_plotCOPD_cuneus_thickavg_noGC.png]

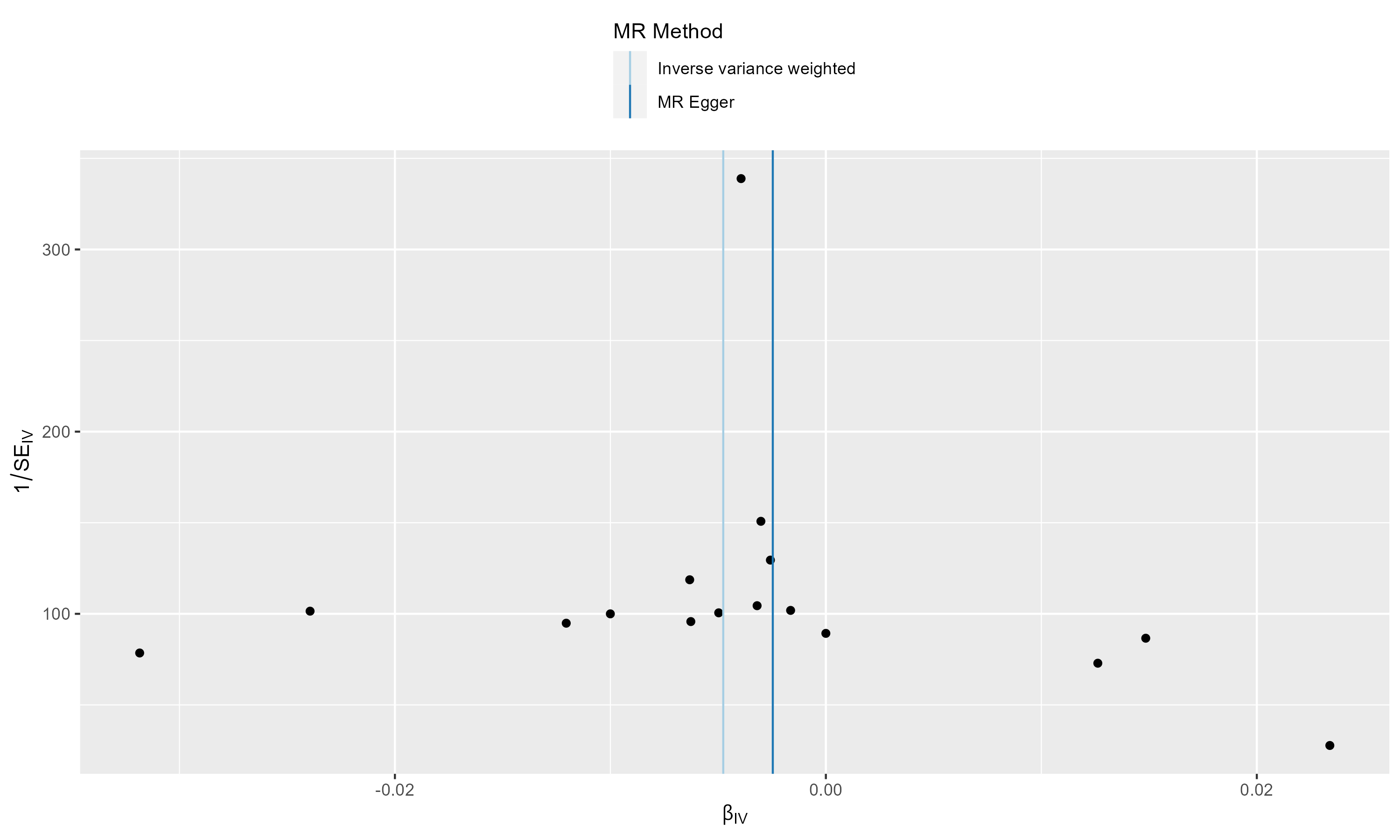

Supplement: Supplementary file 12 — Supplementary Material 12. [file 12890_2024_3150_MOESM12_ESM.zip › Supplementary Figure/funnel plot/Cortex Thickness/funnel_plotCOPD_inferiorparietal_thickavg.png]

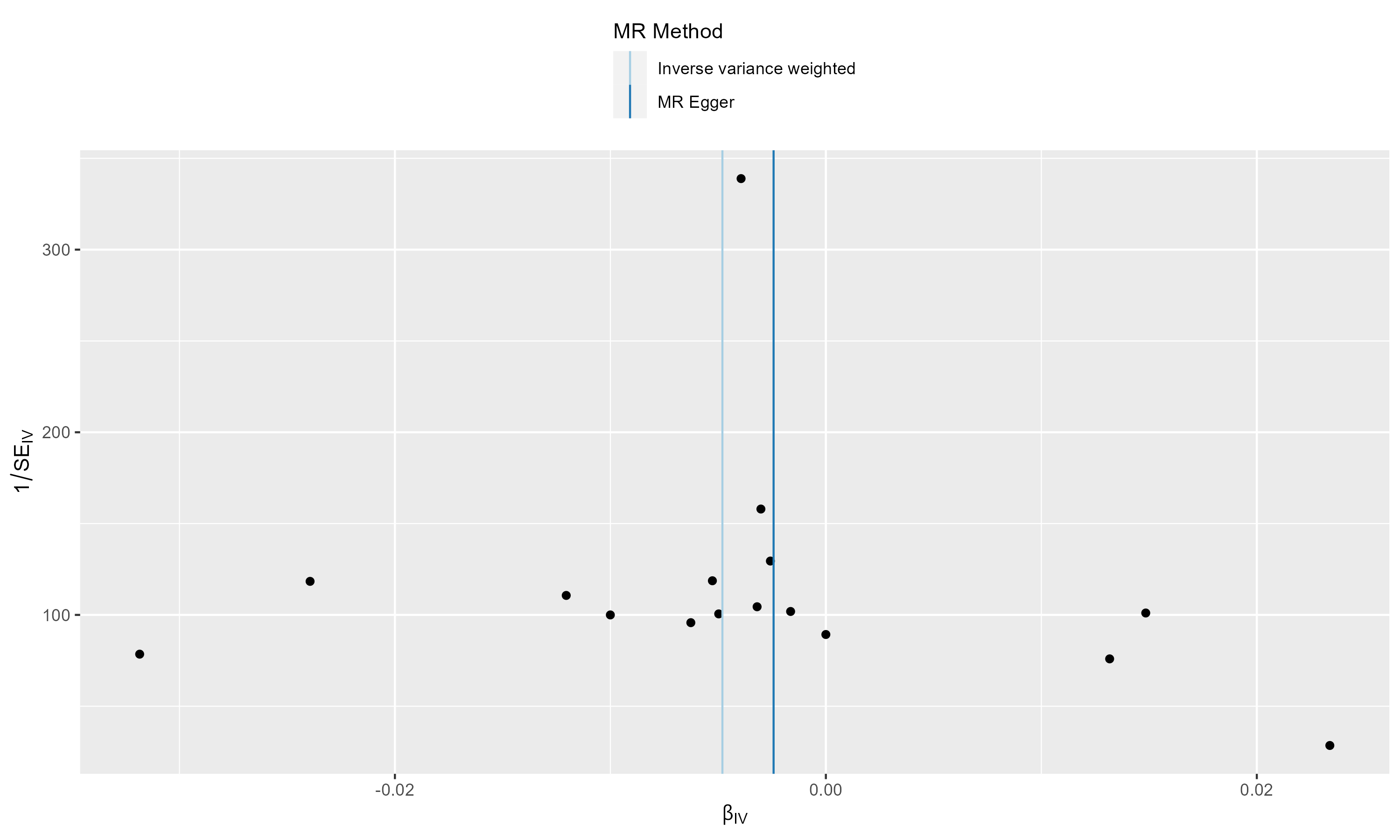

Supplement: Supplementary file 12 — Supplementary Material 12. [file 12890_2024_3150_MOESM12_ESM.zip › Supplementary Figure/funnel plot/Cortex Thickness/funnel_plotCOPD_inferiorparietal_thickavg_noGC.png]

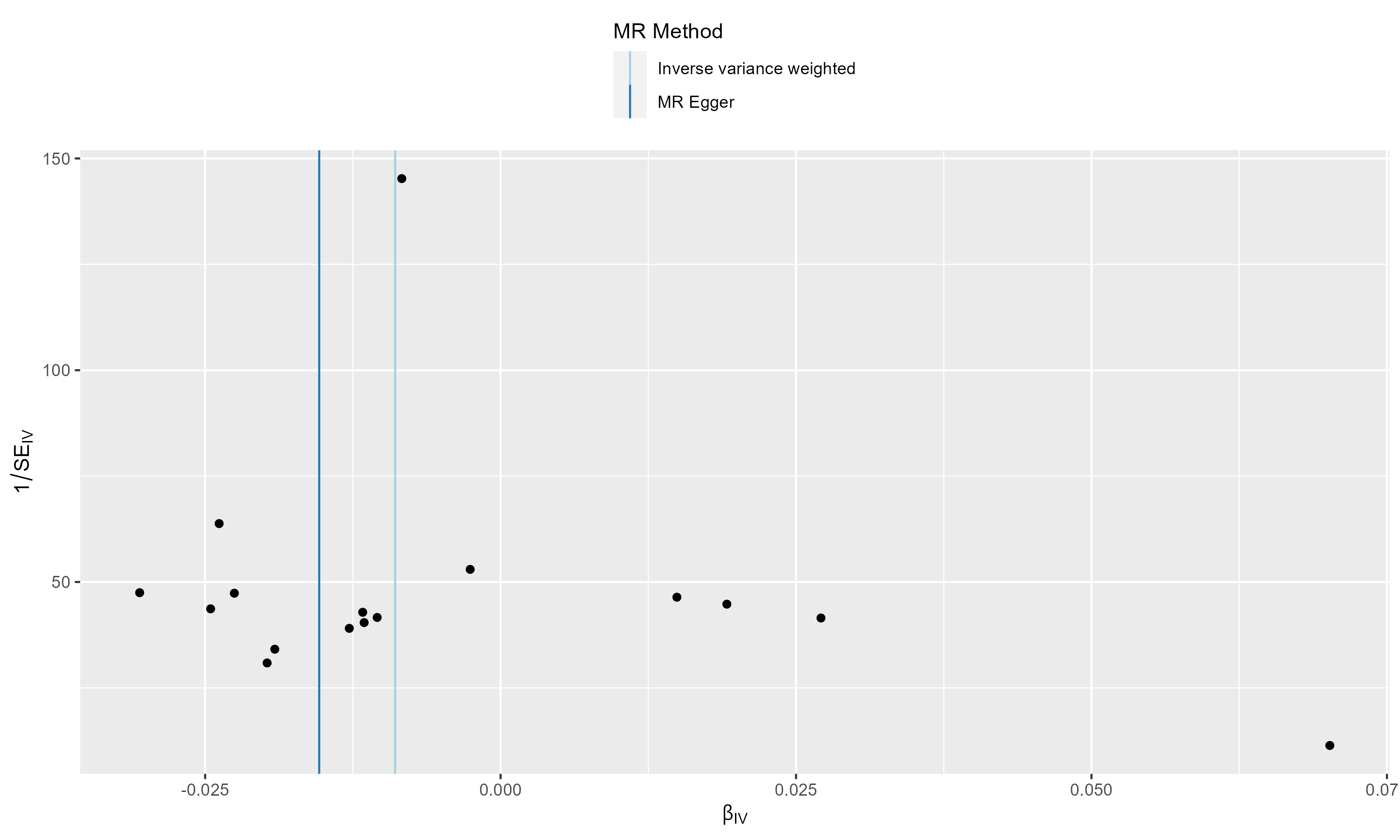

Supplement: Supplementary file 12 — Supplementary Material 12. [file 12890_2024_3150_MOESM12_ESM.zip › Supplementary Figure/funnel plot/Cortex Thickness/funnel_plotCOPD_rostralanteriorcingulate_thickavg_noGC.png]

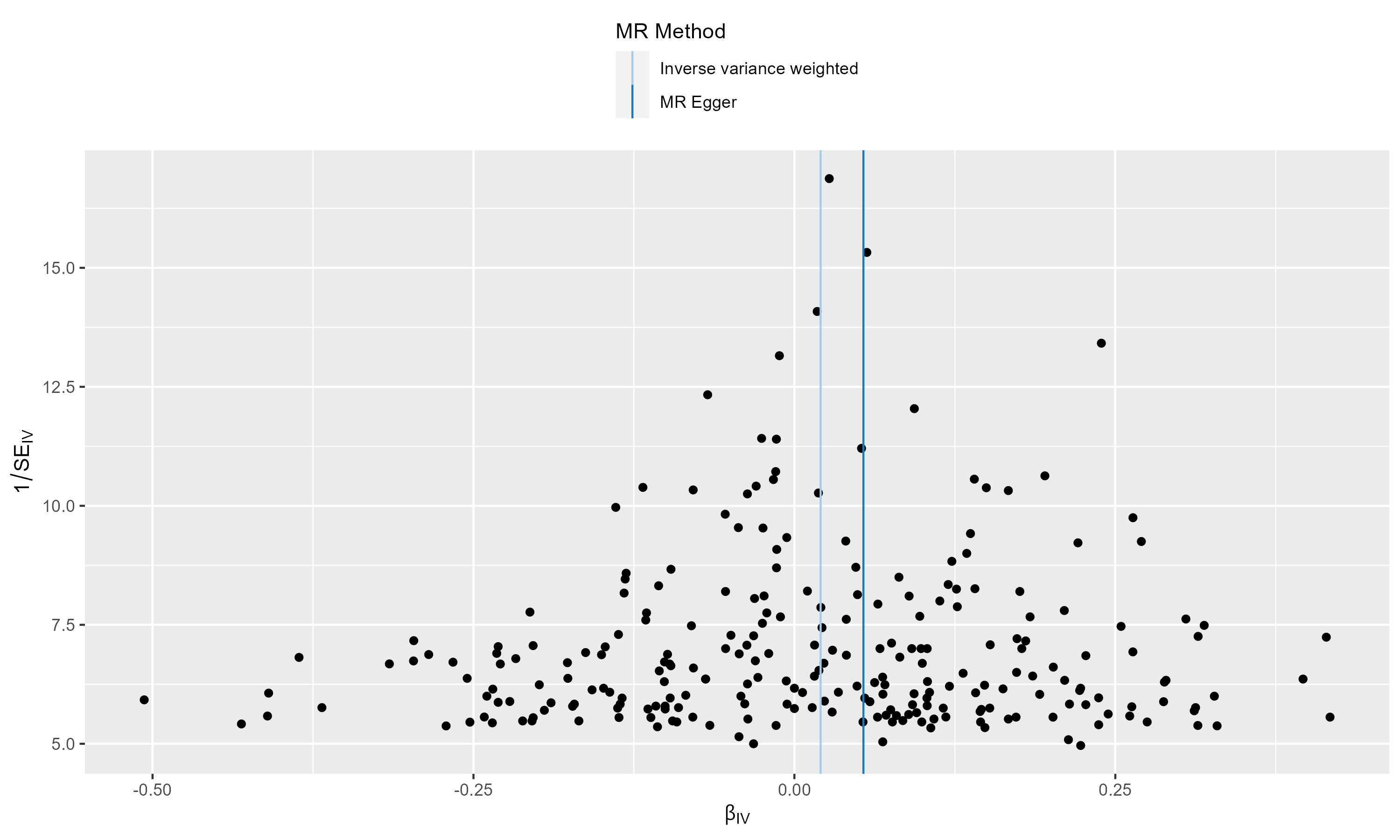

Supplement: Supplementary file 12 — Supplementary Material 12. [file 12890_2024_3150_MOESM12_ESM.zip › Supplementary Figure/funnel plot/Cortex Thickness/funnel_plotFEV1_entorhinal_thickavg.png]

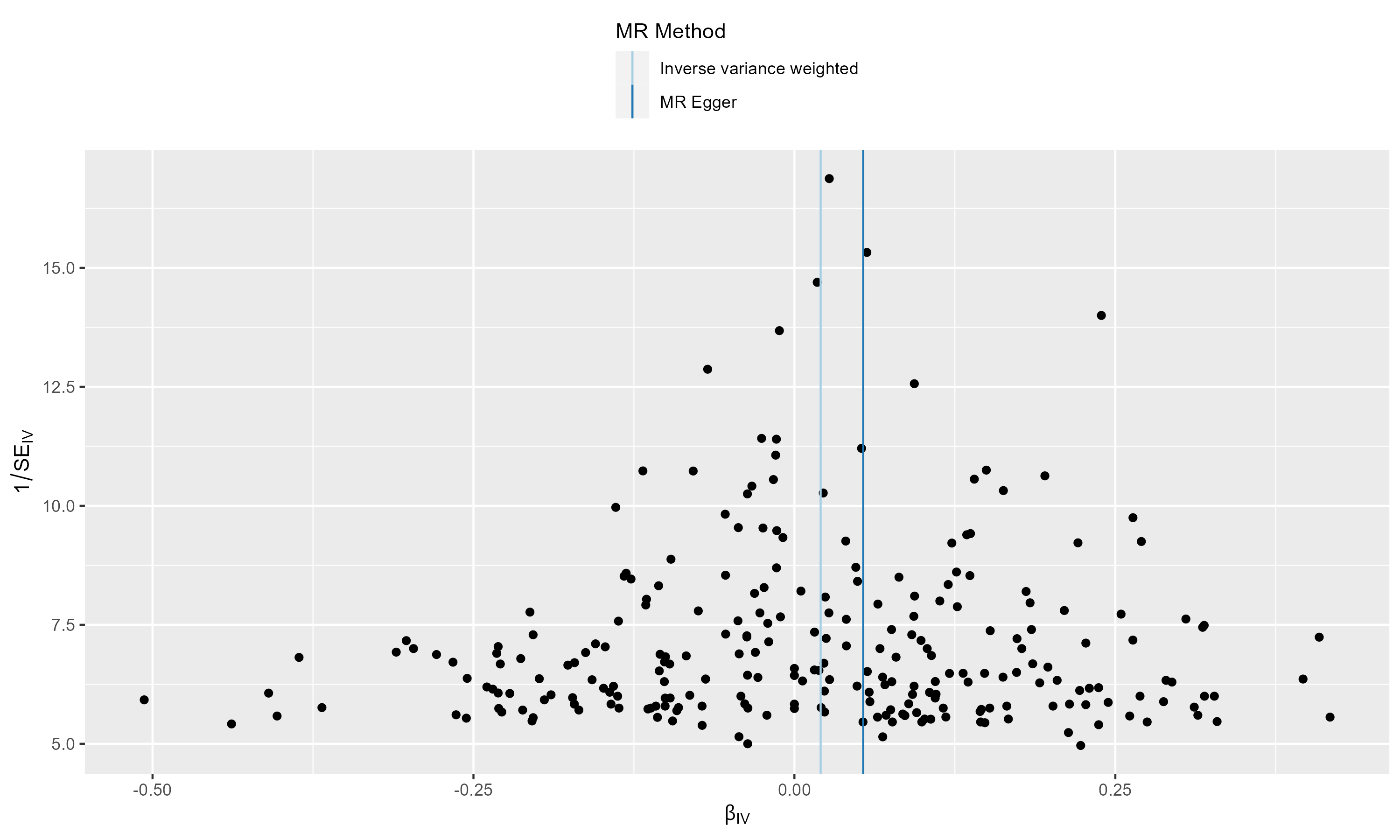

Supplement: Supplementary file 12 — Supplementary Material 12. [file 12890_2024_3150_MOESM12_ESM.zip › Supplementary Figure/funnel plot/Cortex Thickness/funnel_plotFEV1_entorhinal_thickavg_noGC.png]

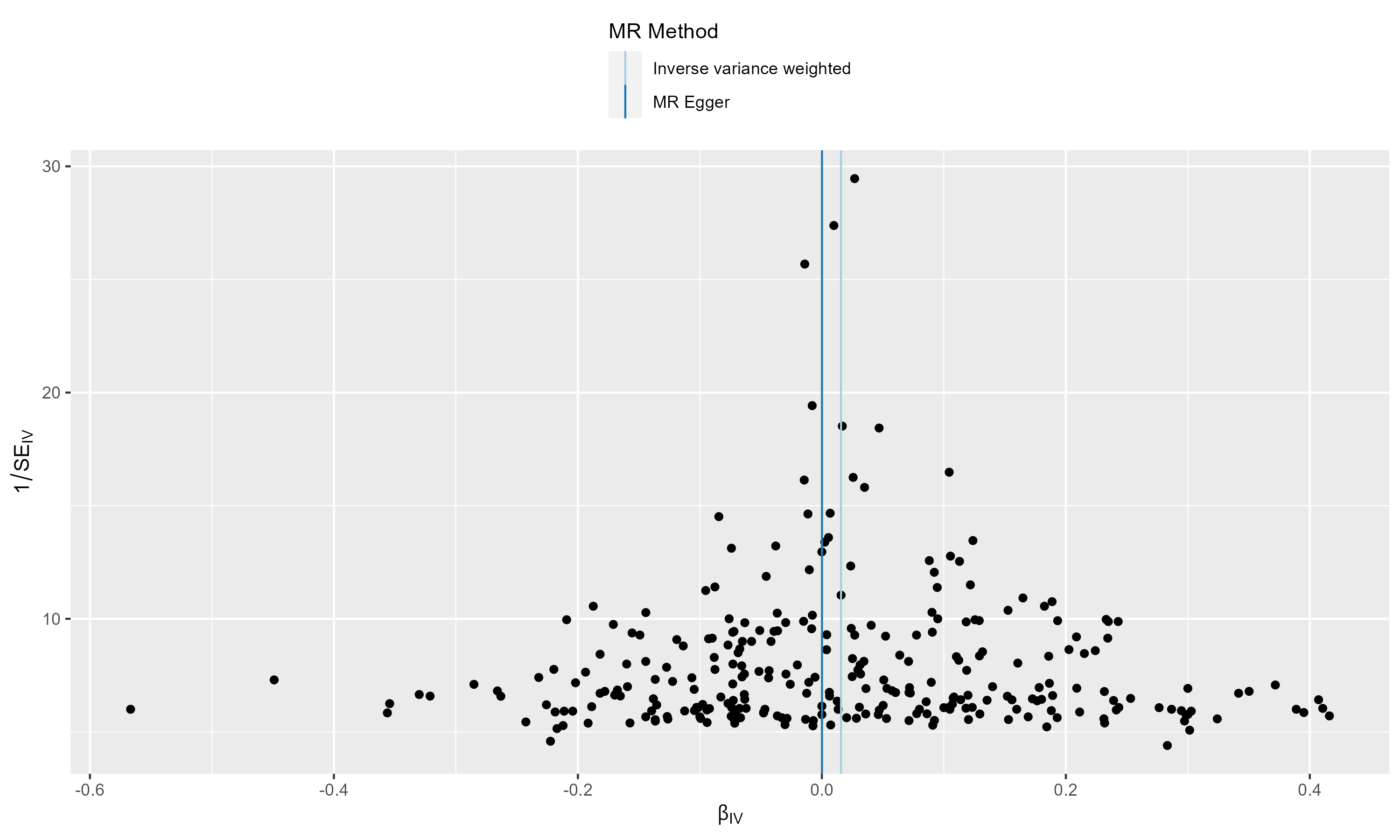

Supplement: Supplementary file 12 — Supplementary Material 12. [file 12890_2024_3150_MOESM12_ESM.zip › Supplementary Figure/funnel plot/Cortex Thickness/funnel_plotFEV1_FVC_entorhinal_thickavg.png]

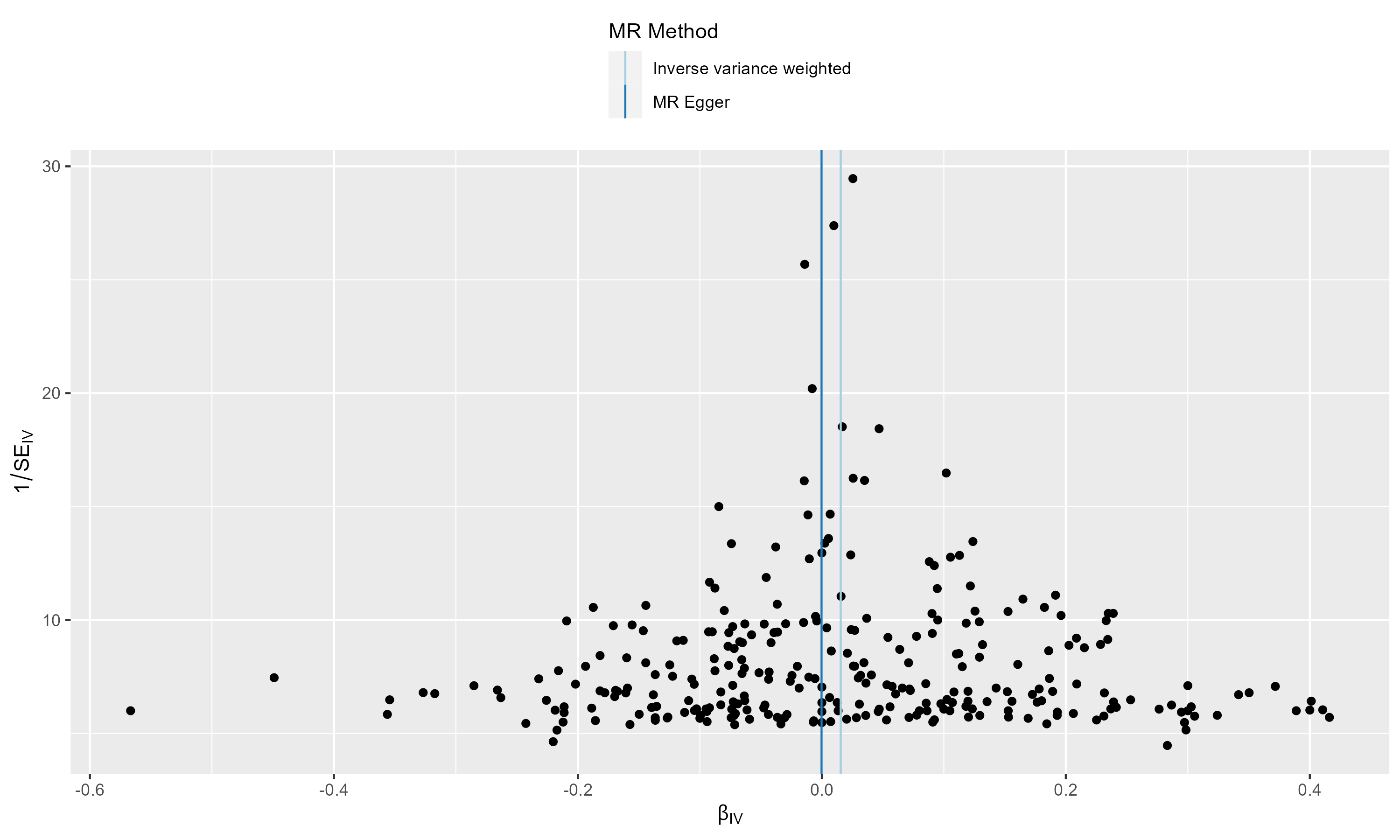

Supplement: Supplementary file 12 — Supplementary Material 12. [file 12890_2024_3150_MOESM12_ESM.zip › Supplementary Figure/funnel plot/Cortex Thickness/funnel_plotFEV1_FVC_entorhinal_thickavg_noGC.png]

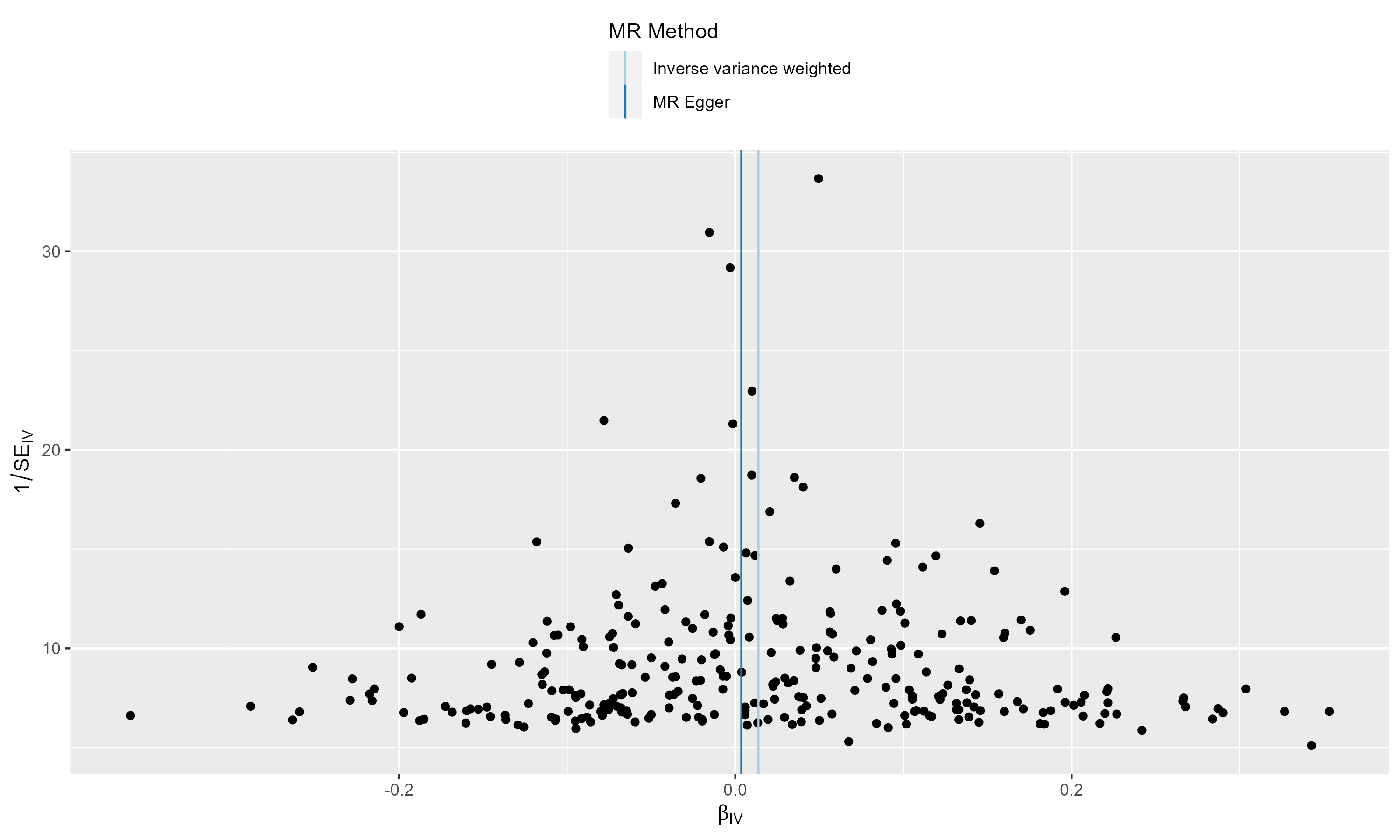

Supplement: Supplementary file 12 — Supplementary Material 12. [file 12890_2024_3150_MOESM12_ESM.zip › Supplementary Figure/funnel plot/Cortex Thickness/funnel_plotFEV1_FVC_temporalpole_thickavg.png]

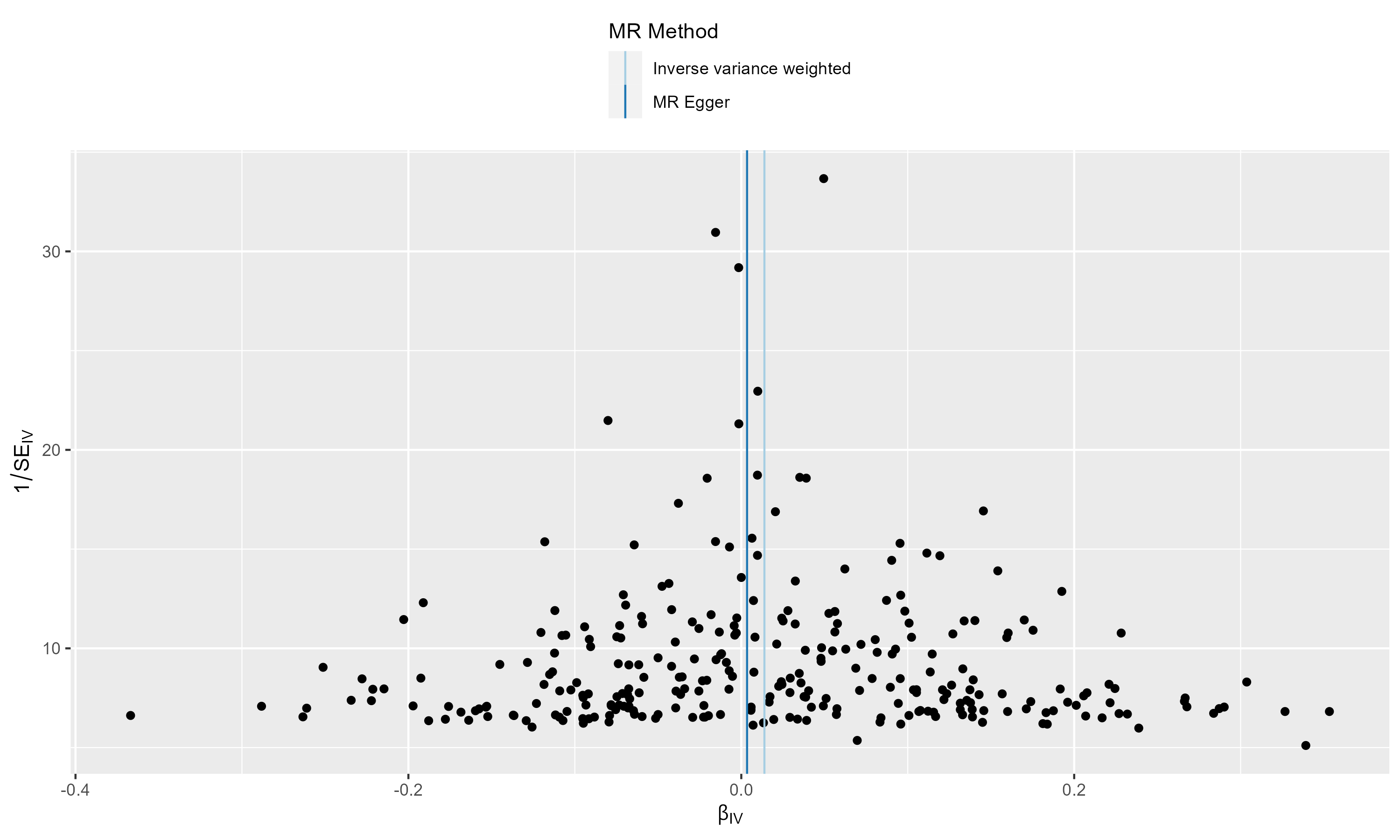

Supplement: Supplementary file 12 — Supplementary Material 12. [file 12890_2024_3150_MOESM12_ESM.zip › Supplementary Figure/funnel plot/Cortex Thickness/funnel_plotFEV1_FVC_temporalpole_thickavg_noGC.png]

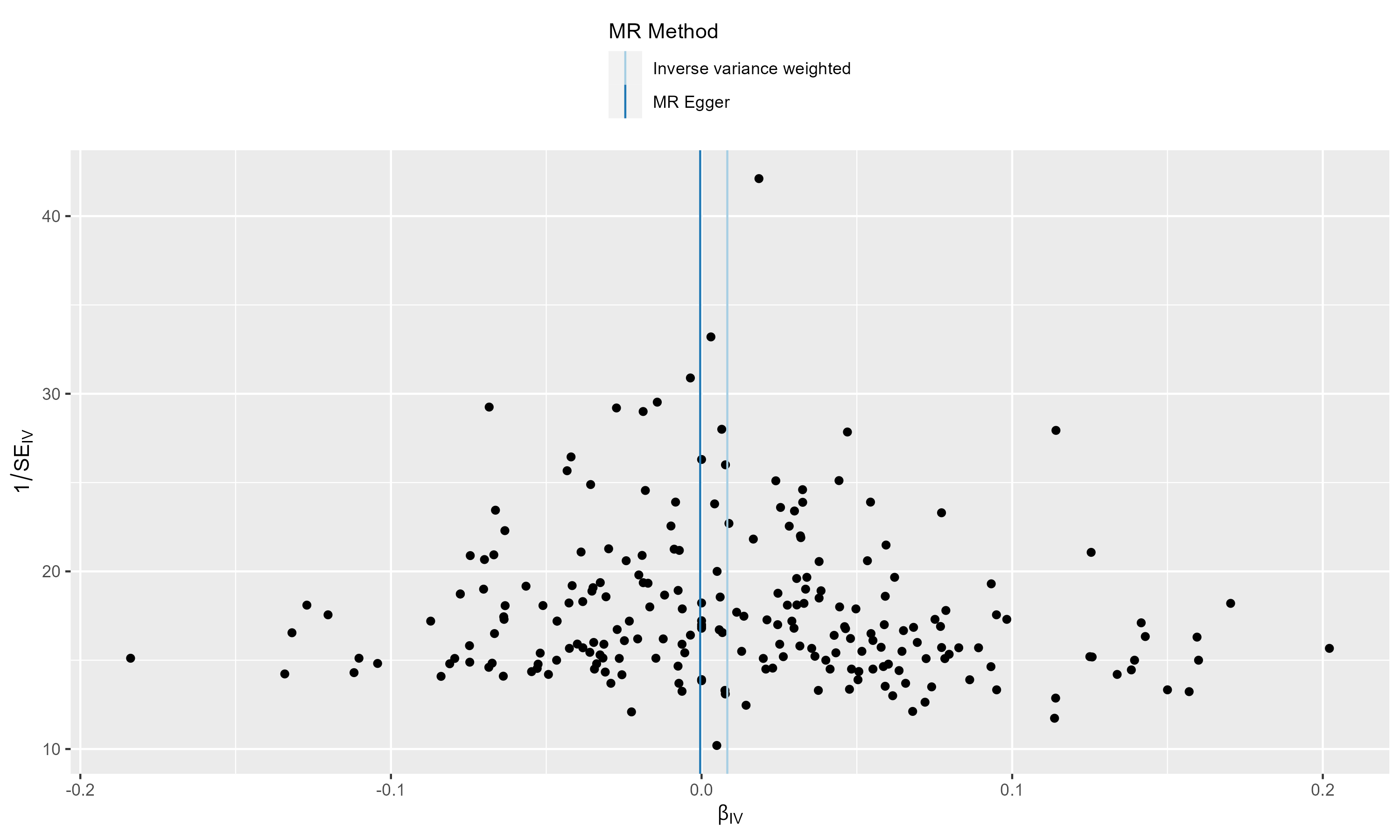

Supplement: Supplementary file 12 — Supplementary Material 12. [file 12890_2024_3150_MOESM12_ESM.zip › Supplementary Figure/funnel plot/Cortex Thickness/funnel_plotFVC_bankssts_thickavg.png]

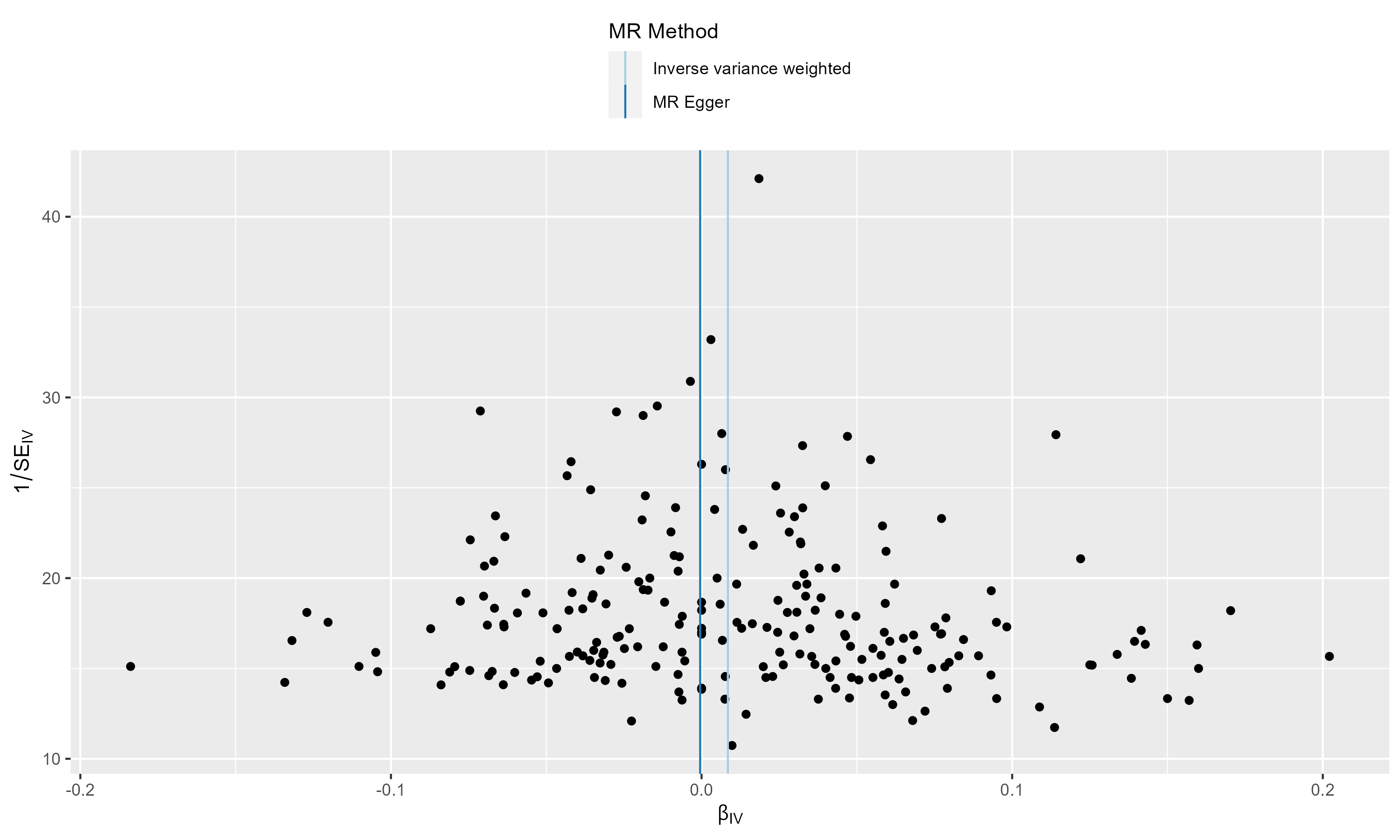

Supplement: Supplementary file 12 — Supplementary Material 12. [file 12890_2024_3150_MOESM12_ESM.zip › Supplementary Figure/funnel plot/Cortex Thickness/funnel_plotFVC_bankssts_thickavg_noGC.png]

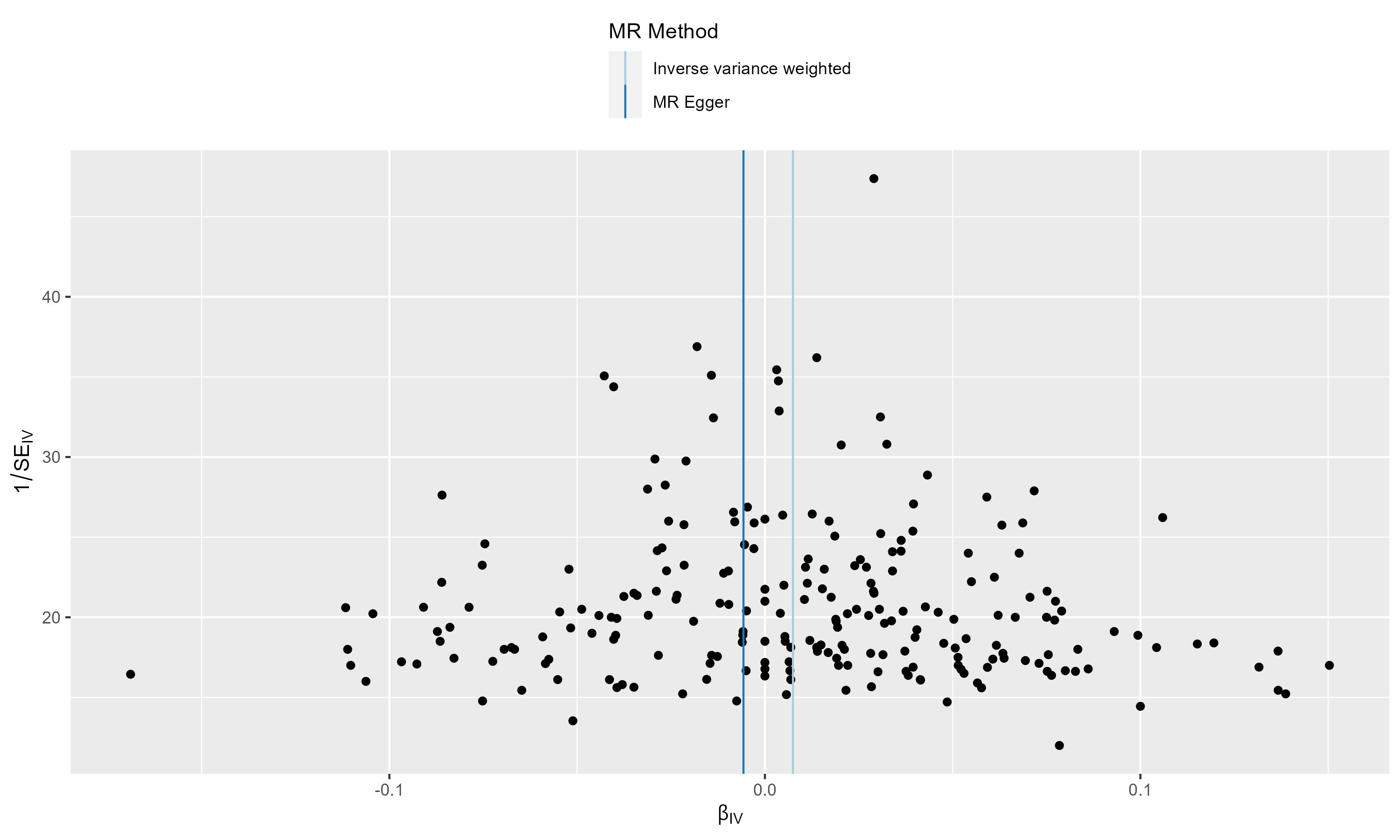

Supplement: Supplementary file 12 — Supplementary Material 12. [file 12890_2024_3150_MOESM12_ESM.zip › Supplementary Figure/funnel plot/Cortex Thickness/funnel_plotFVC_fusiform_thickavg.png]

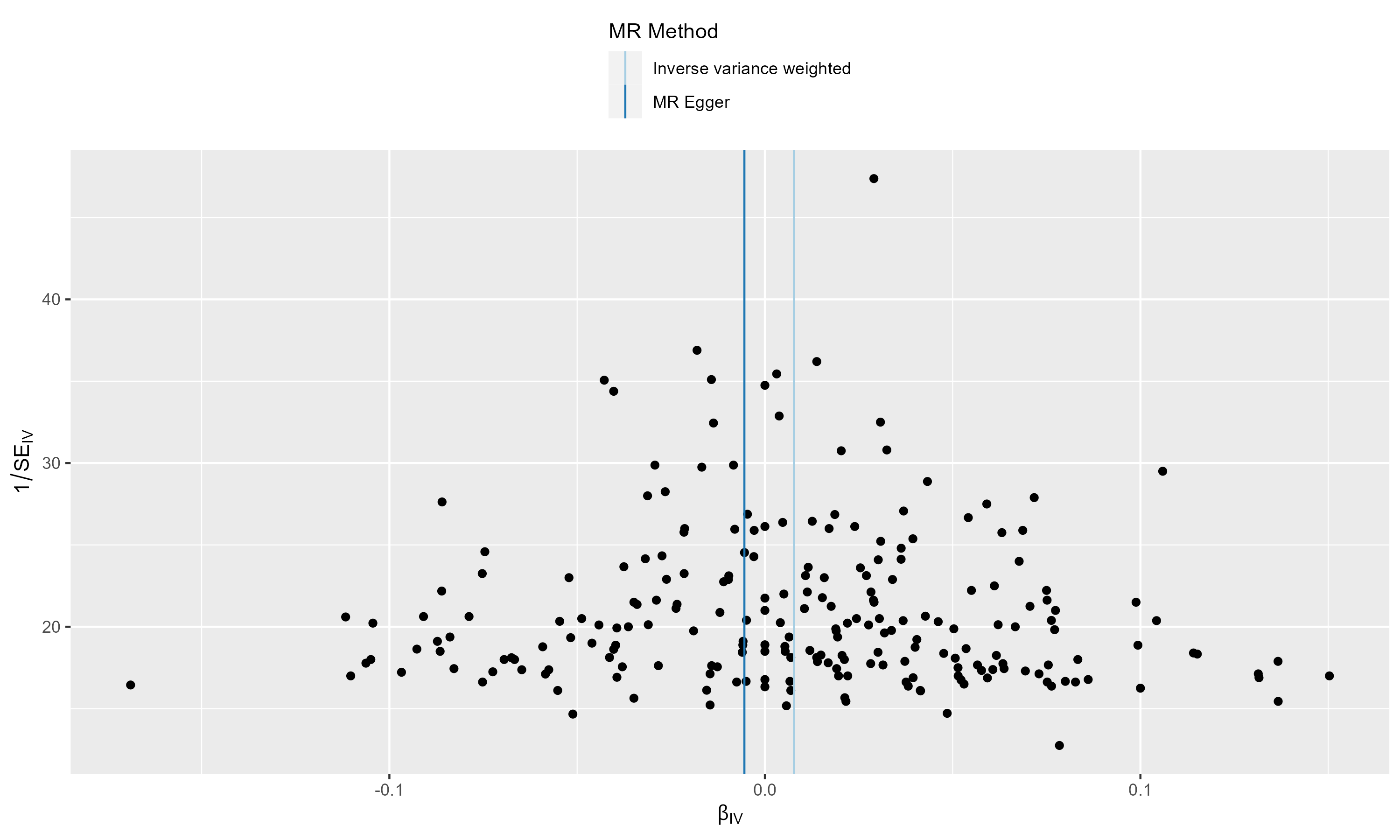

Supplement: Supplementary file 12 — Supplementary Material 12. [file 12890_2024_3150_MOESM12_ESM.zip › Supplementary Figure/funnel plot/Cortex Thickness/funnel_plotFVC_fusiform_thickavg_noGC.png]

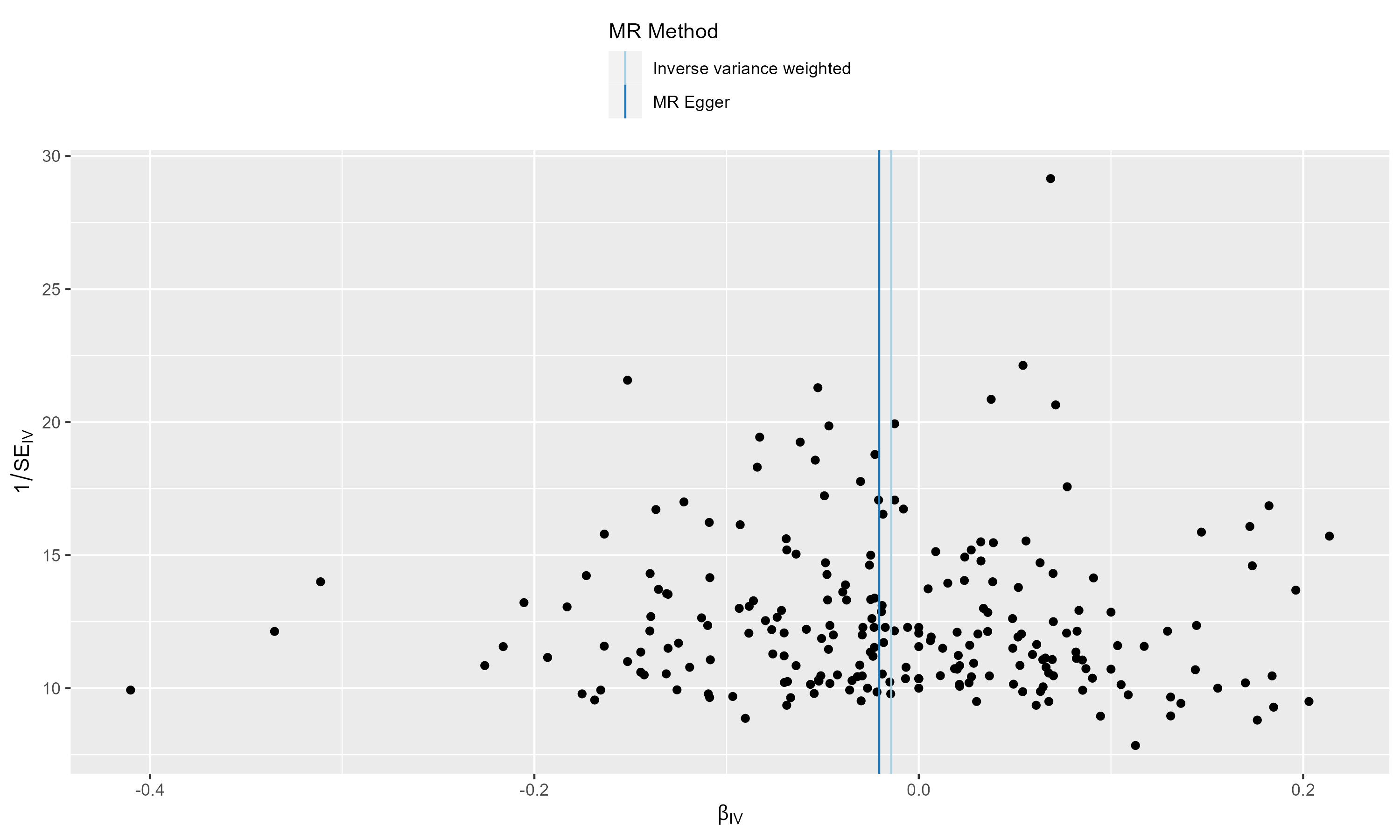

Supplement: Supplementary file 12 — Supplementary Material 12. [file 12890_2024_3150_MOESM12_ESM.zip › Supplementary Figure/funnel plot/Cortex Thickness/funnel_plotFVC_isthmuscingulate_thickavg.png]

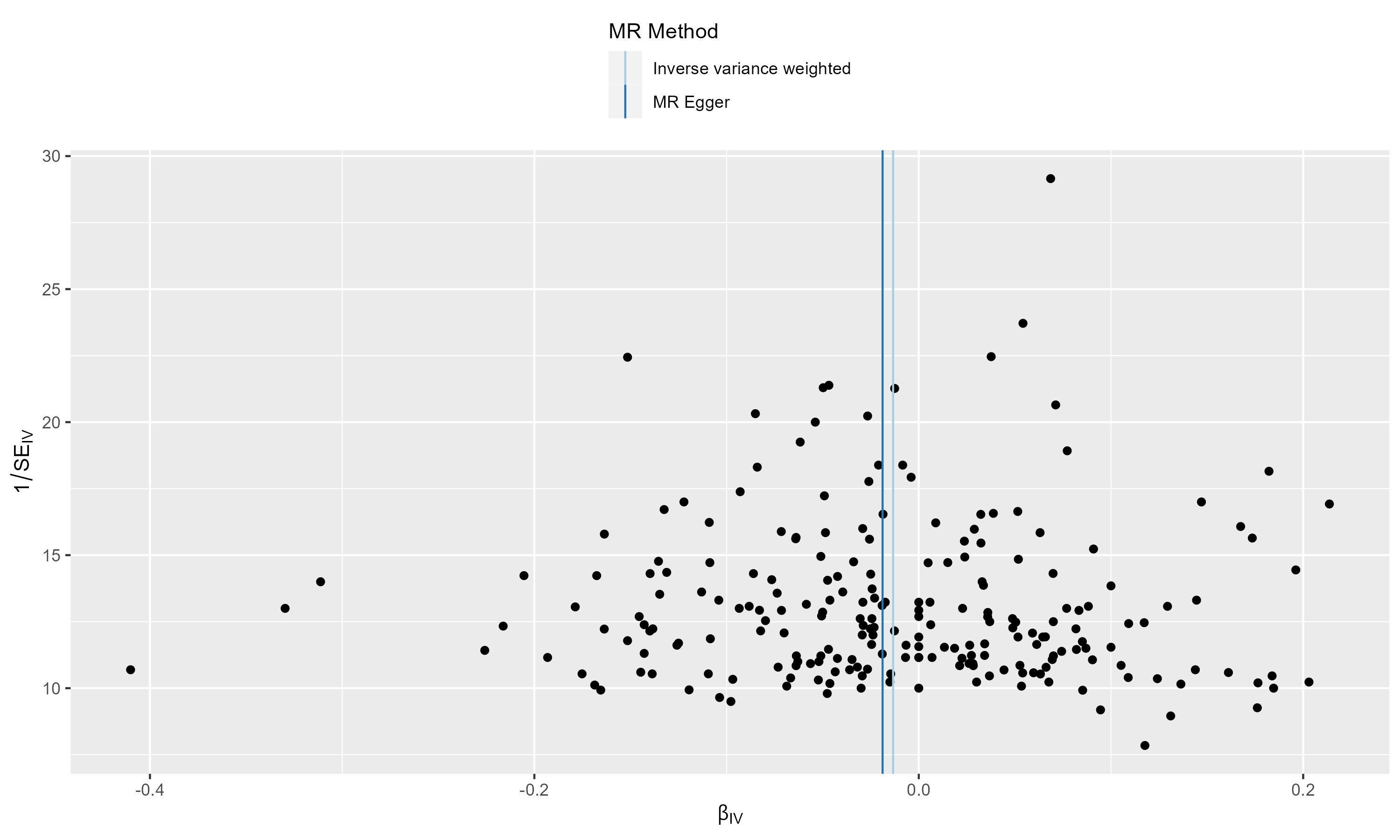

Supplement: Supplementary file 12 — Supplementary Material 12. [file 12890_2024_3150_MOESM12_ESM.zip › Supplementary Figure/funnel plot/Cortex Thickness/funnel_plotFVC_isthmuscingulate_thickavg_noGC.png]

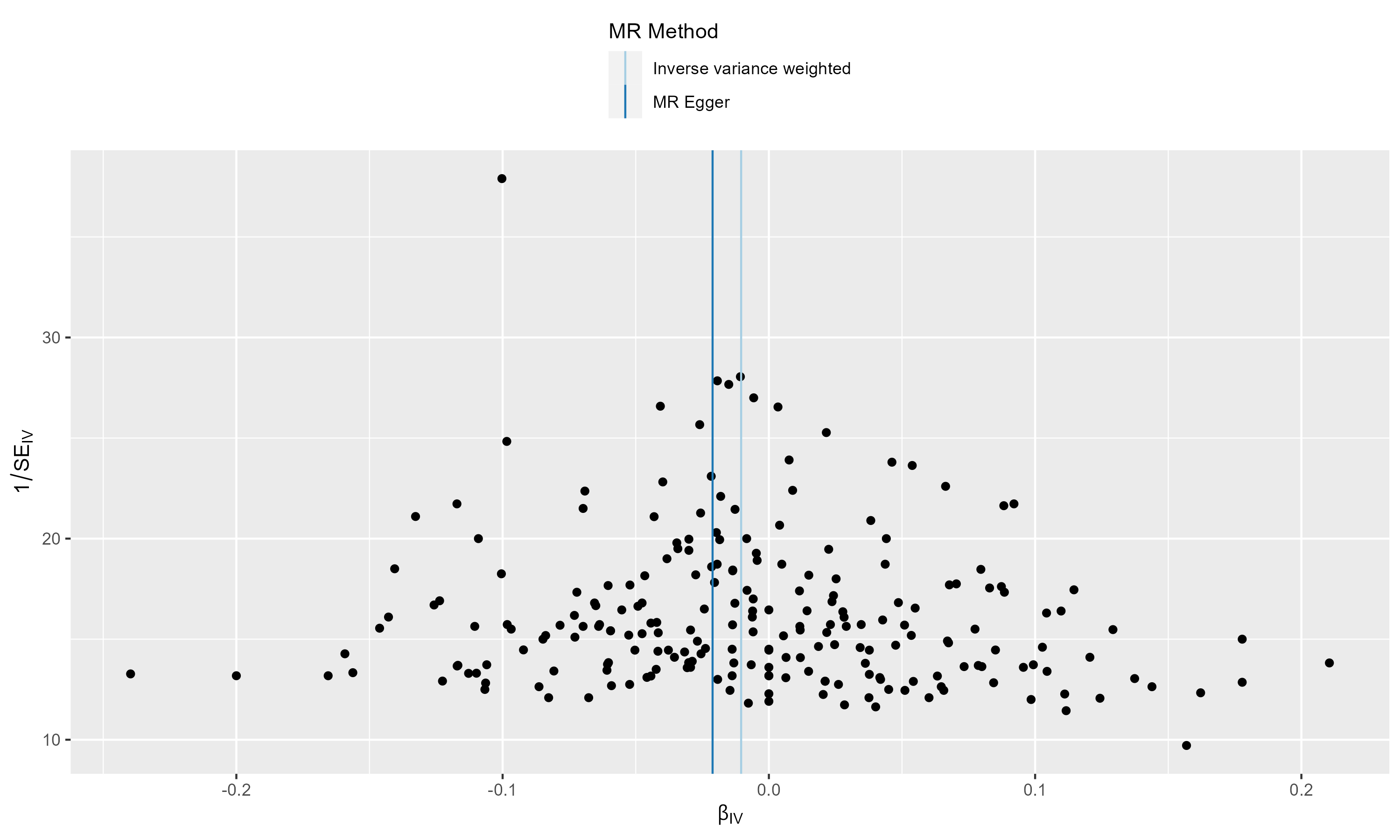

Supplement: Supplementary file 12 — Supplementary Material 12. [file 12890_2024_3150_MOESM12_ESM.zip › Supplementary Figure/funnel plot/Cortex Thickness/funnel_plotFVC_medialorbitofrontal_thickavg.png]

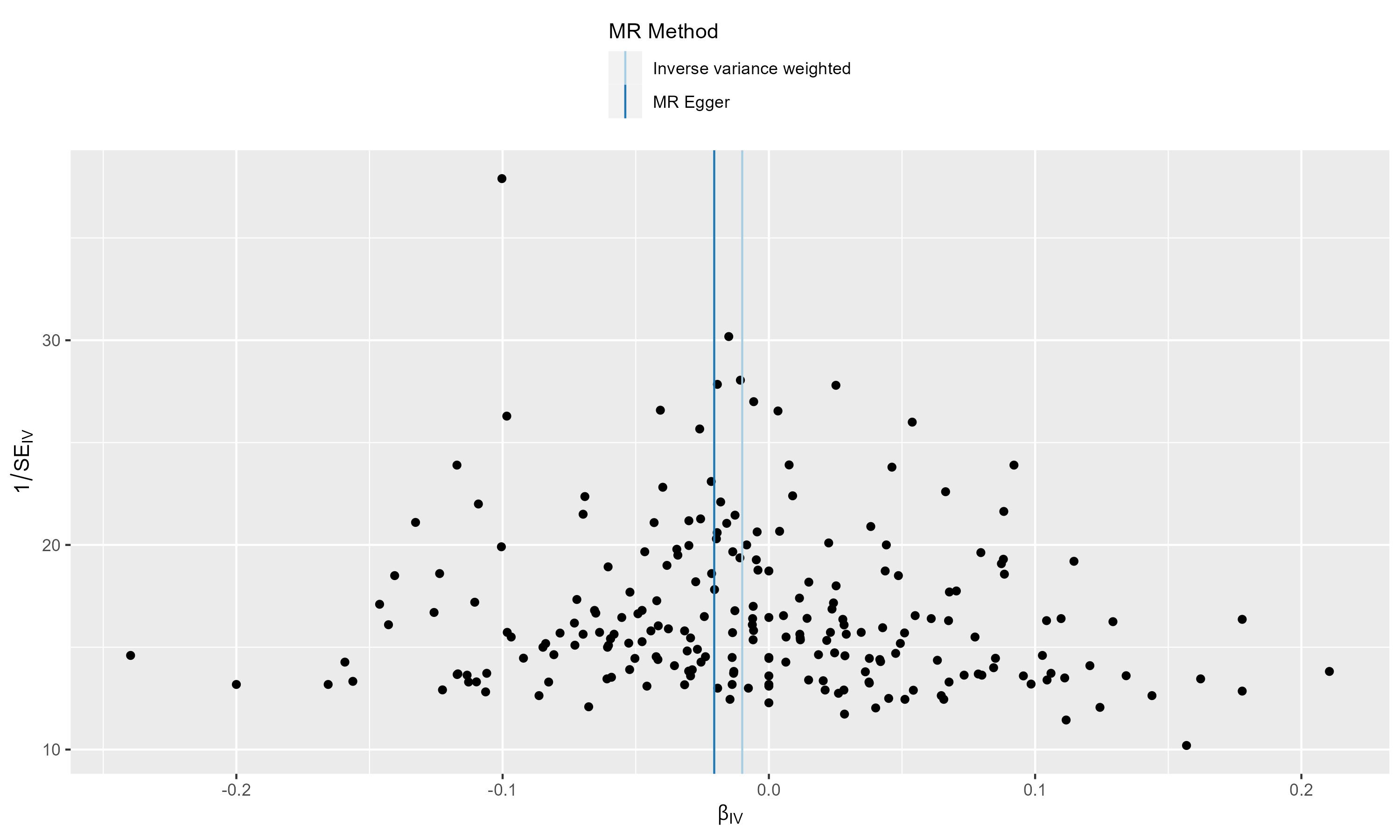

Supplement: Supplementary file 12 — Supplementary Material 12. [file 12890_2024_3150_MOESM12_ESM.zip › Supplementary Figure/funnel plot/Cortex Thickness/funnel_plotFVC_medialorbitofrontal_thickavg_noGC.png]

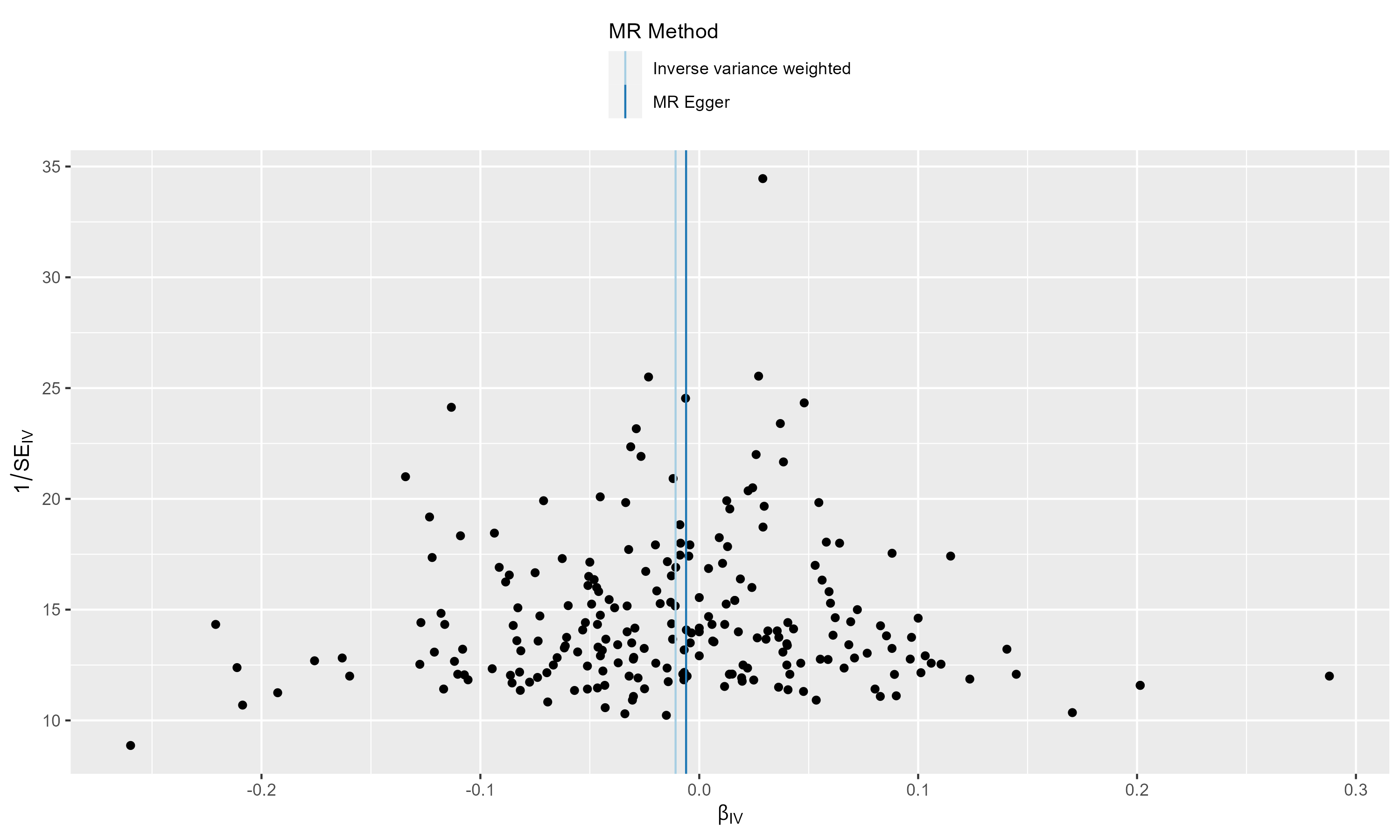

Supplement: Supplementary file 12 — Supplementary Material 12. [file 12890_2024_3150_MOESM12_ESM.zip › Supplementary Figure/funnel plot/Cortex Thickness/funnel_plotFVC_parsorbitalis_thickavg.png]

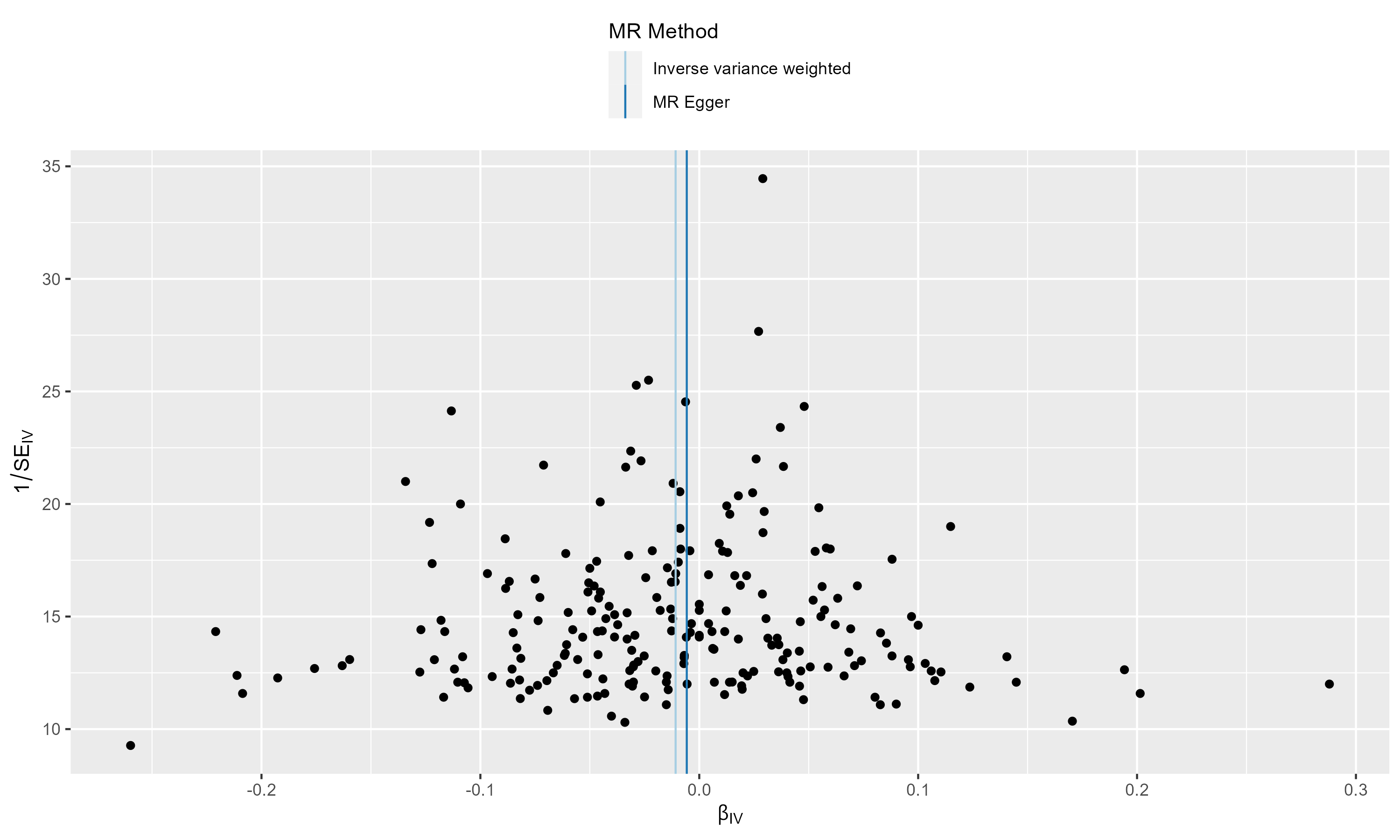

Supplement: Supplementary file 12 — Supplementary Material 12. [file 12890_2024_3150_MOESM12_ESM.zip › Supplementary Figure/funnel plot/Cortex Thickness/funnel_plotFVC_parsorbitalis_thickavg_noGC.png]

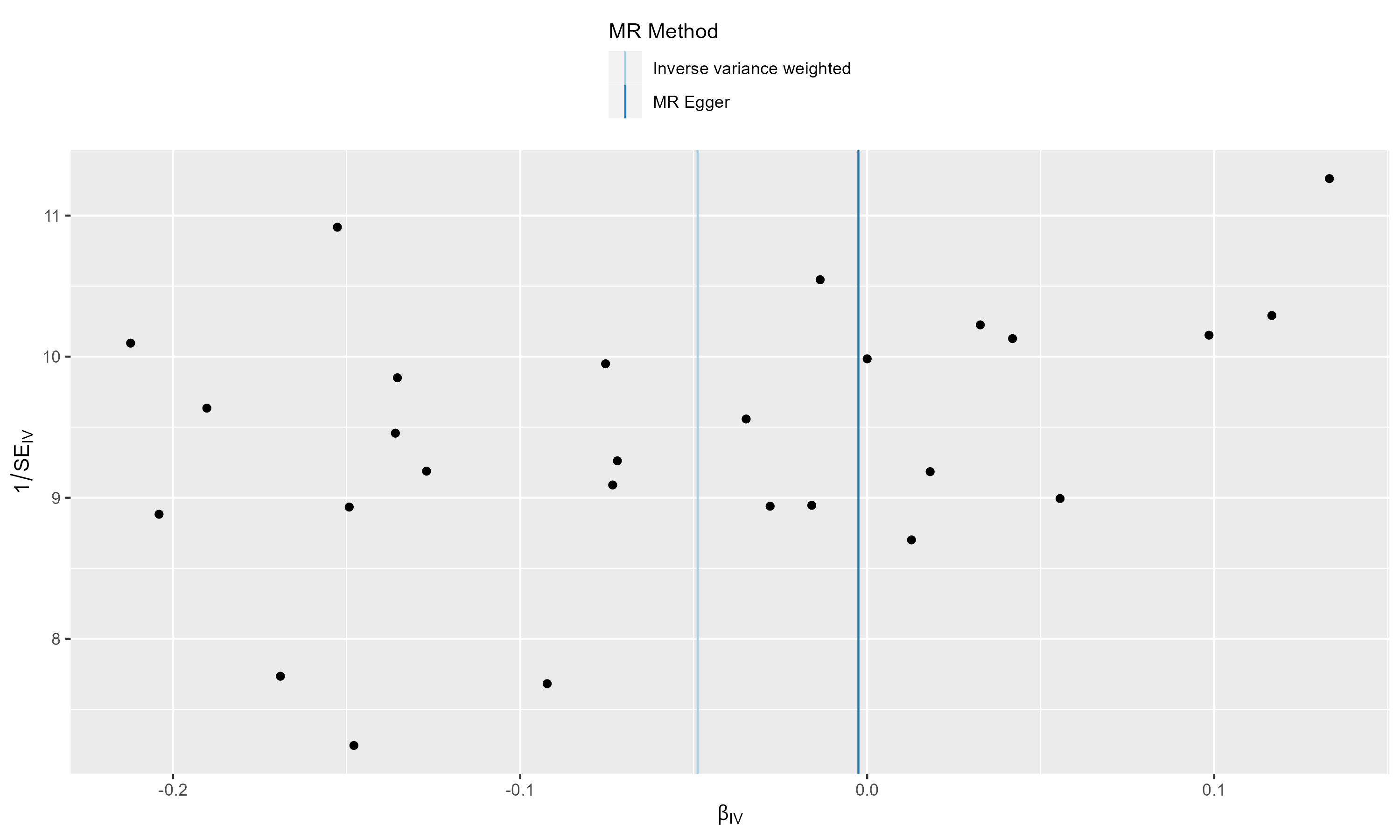

Supplement: Supplementary file 12 — Supplementary Material 12. [file 12890_2024_3150_MOESM12_ESM.zip › Supplementary Figure/funnel plot/Cortex Thickness/funnel_plotPRISM_inferiorparietal_thickavg_GC.png]

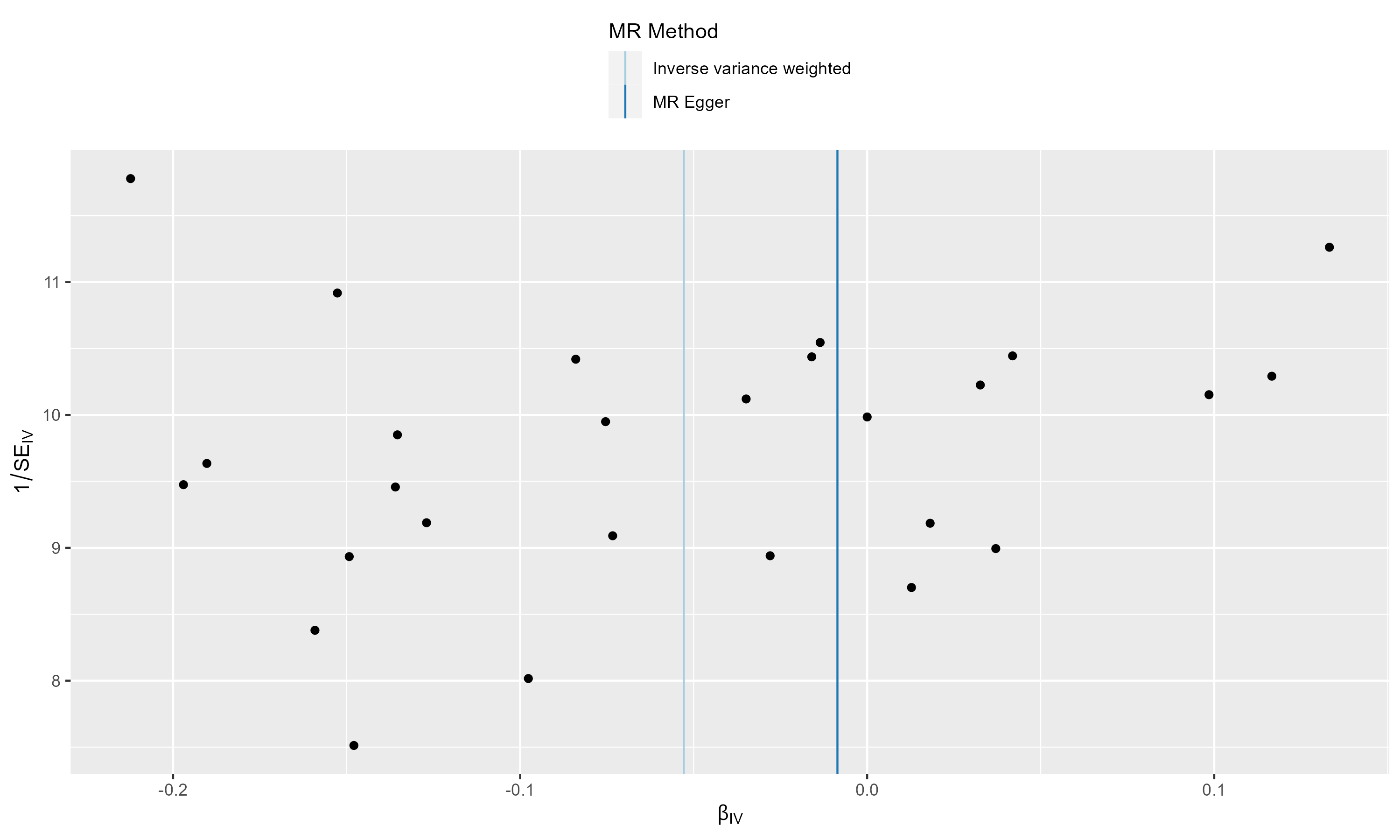

Supplement: Supplementary file 12 — Supplementary Material 12. [file 12890_2024_3150_MOESM12_ESM.zip › Supplementary Figure/funnel plot/Cortex Thickness/funnel_plotPRISM_inferiorparietal_thickavg_noGC.png]

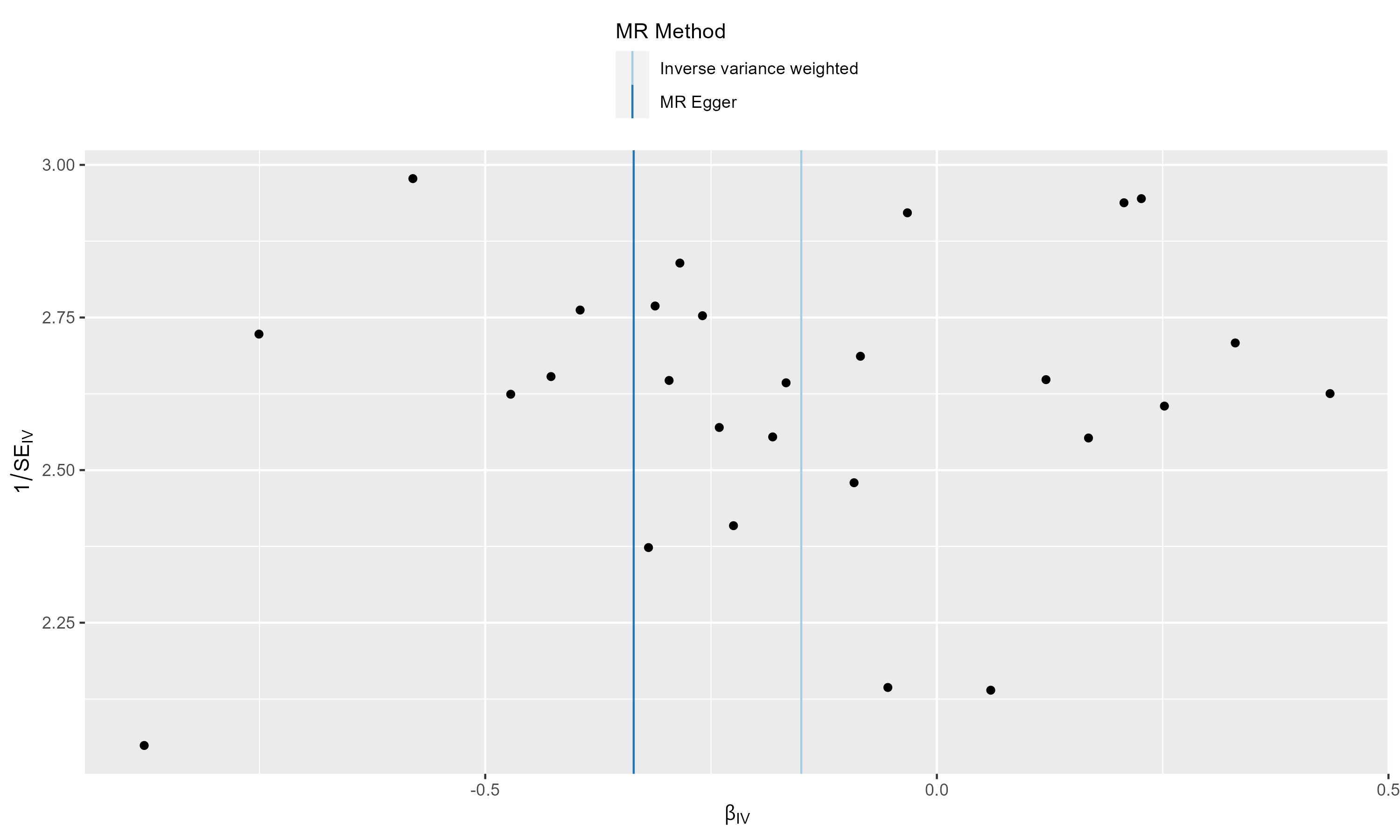

Supplement: Supplementary file 12 — Supplementary Material 12. [file 12890_2024_3150_MOESM12_ESM.zip › Supplementary Figure/funnel plot/Cortex Thickness/funnel_plotPRISM_temporalpole_thickavg_GC.png]

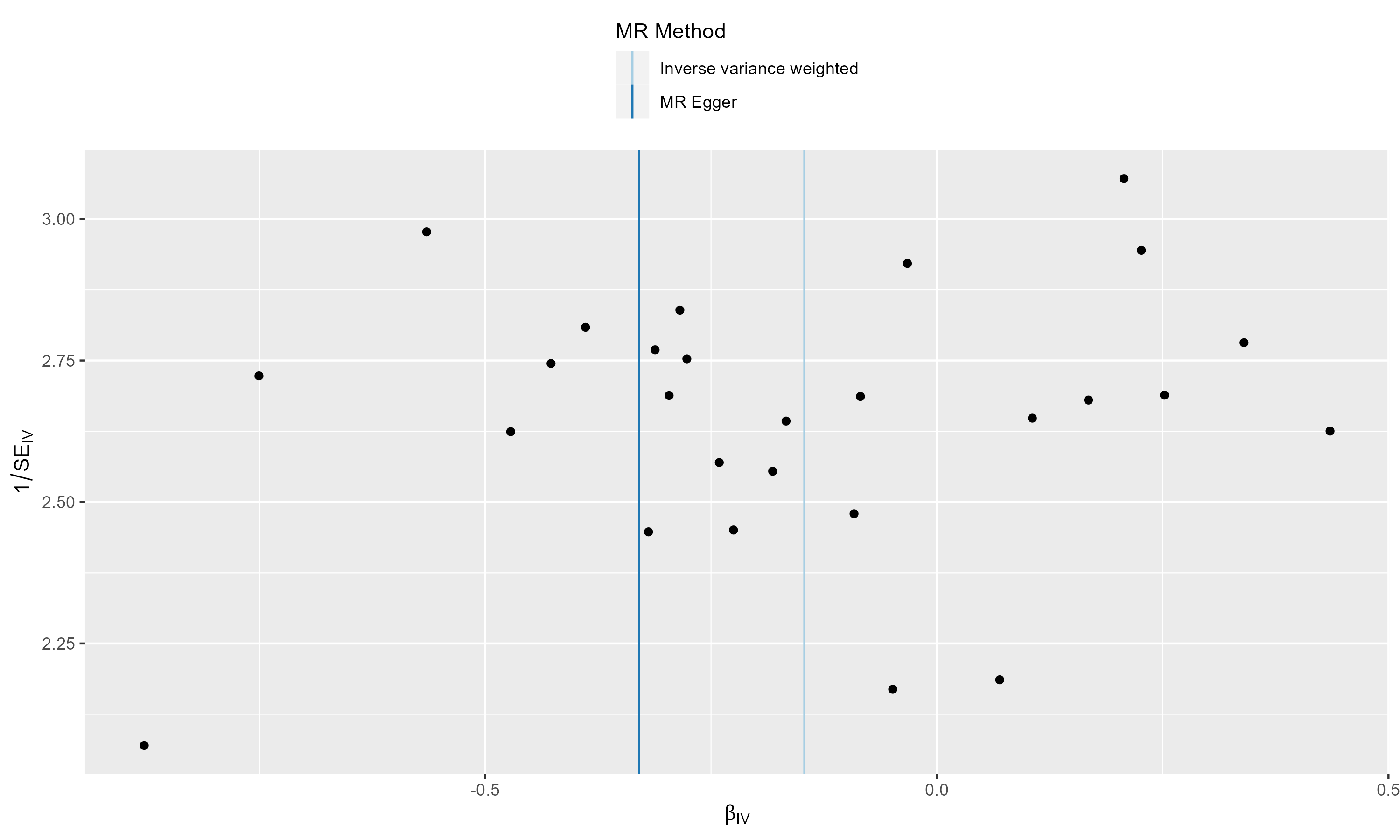

Supplement: Supplementary file 12 — Supplementary Material 12. [file 12890_2024_3150_MOESM12_ESM.zip › Supplementary Figure/funnel plot/Cortex Thickness/funnel_plotPRISM_temporalpole_thickavg_noGC.png]

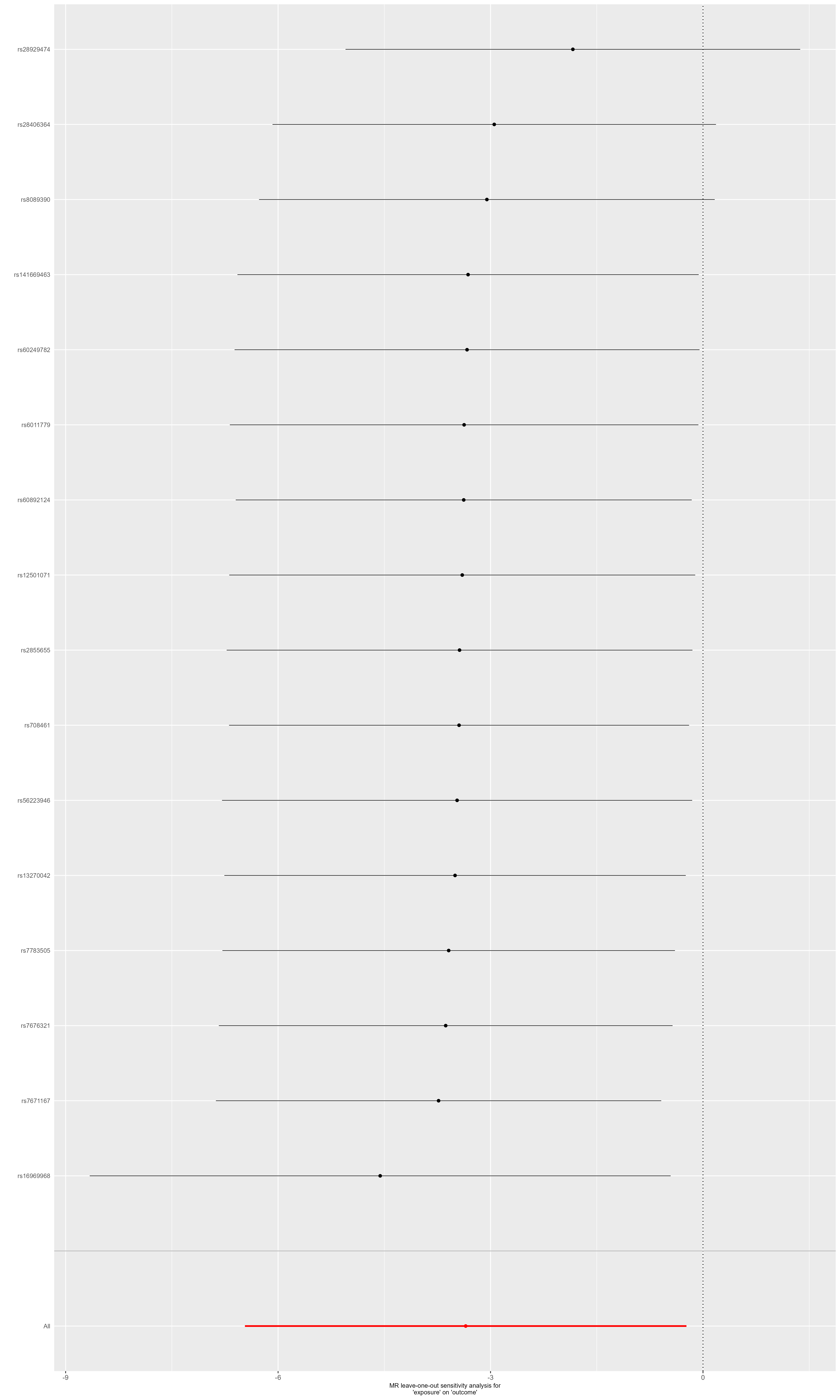

Supplement: Supplementary file 12 — Supplementary Material 12. [file 12890_2024_3150_MOESM12_ESM.zip › Supplementary Figure/leave-one-out analysis/Cortex Surface area/LOOA_COPD_parsorbitalis_surfavg.png]

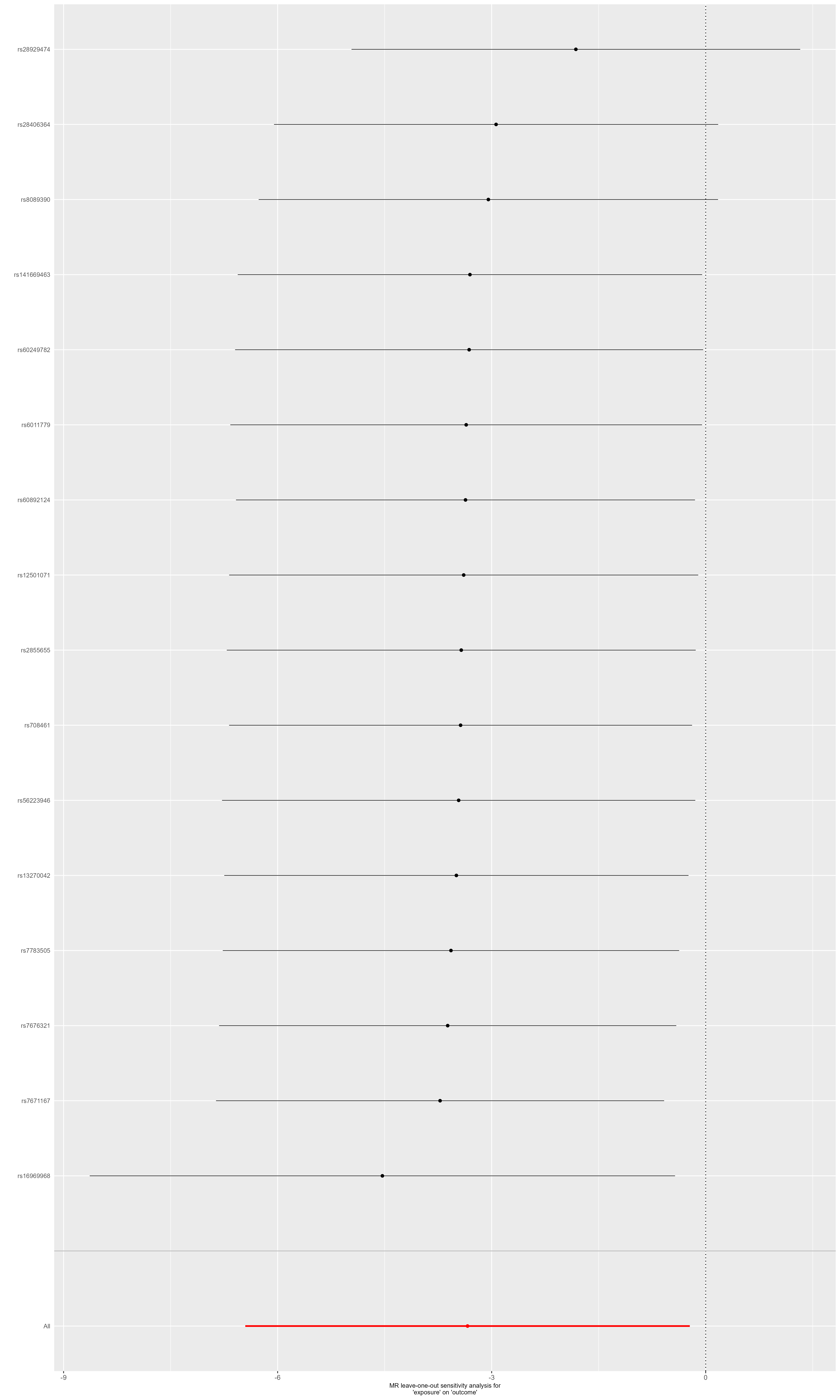

Supplement: Supplementary file 12 — Supplementary Material 12. [file 12890_2024_3150_MOESM12_ESM.zip › Supplementary Figure/leave-one-out analysis/Cortex Surface area/LOOA_COPD_parsorbitalis_surfavg_noGC.png]

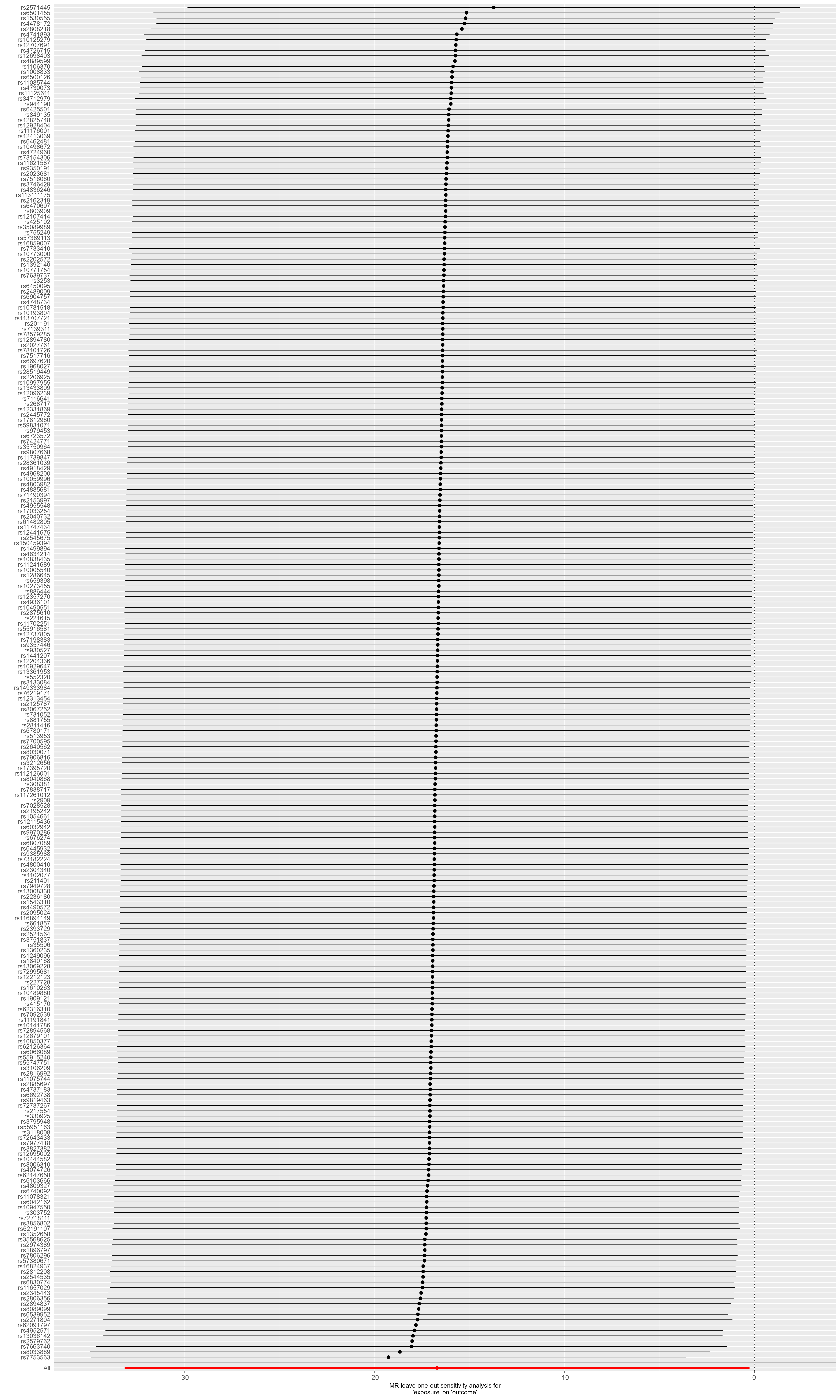

Supplement: Supplementary file 12 — Supplementary Material 12. [file 12890_2024_3150_MOESM12_ESM.zip › Supplementary Figure/leave-one-out analysis/Cortex Surface area/LOOA_FEV1_caudalmiddlefrontal_surfavg.png]

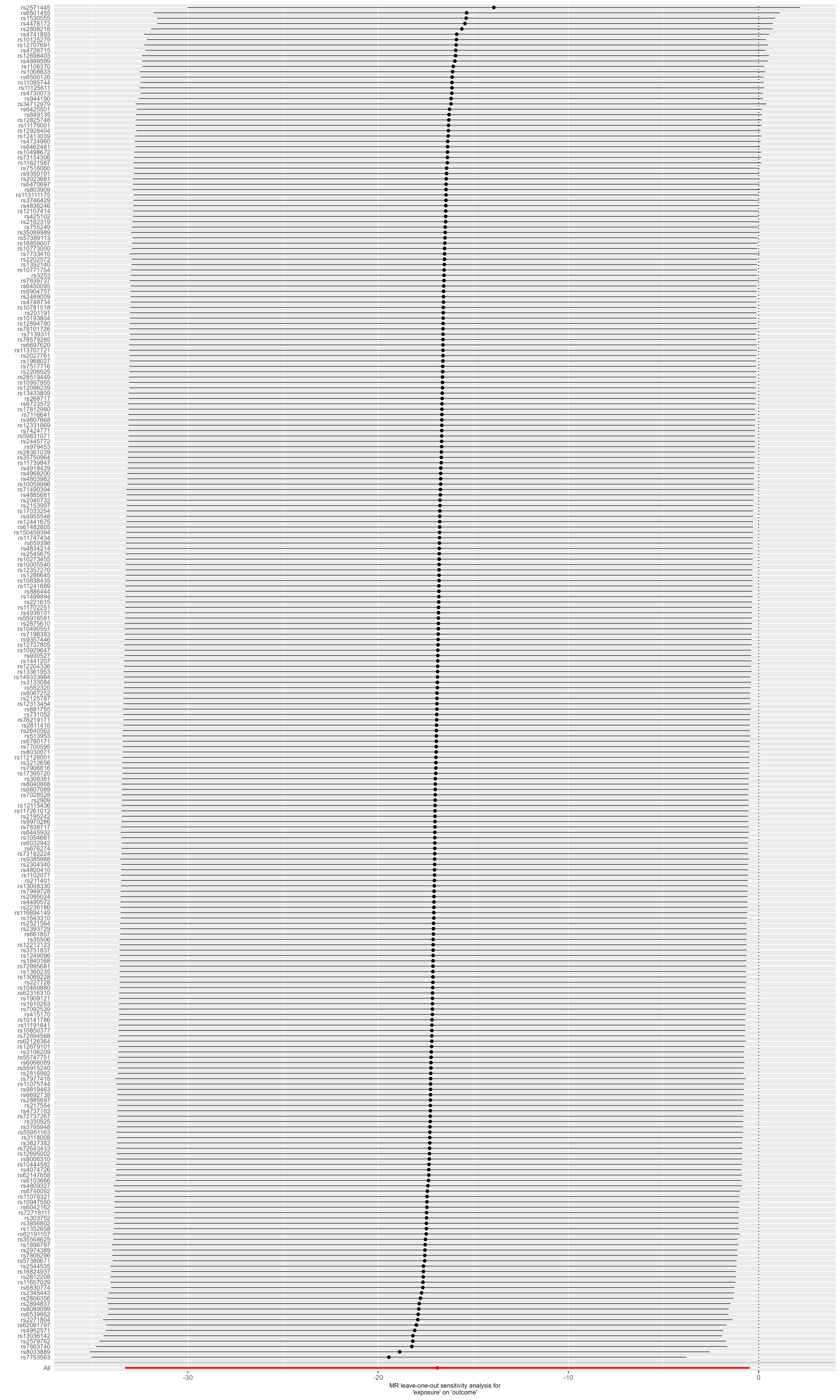

Supplement: Supplementary file 12 — Supplementary Material 12. [file 12890_2024_3150_MOESM12_ESM.zip › Supplementary Figure/leave-one-out analysis/Cortex Surface area/LOOA_FEV1_caudalmiddlefrontal_surfavg_noGC.png]

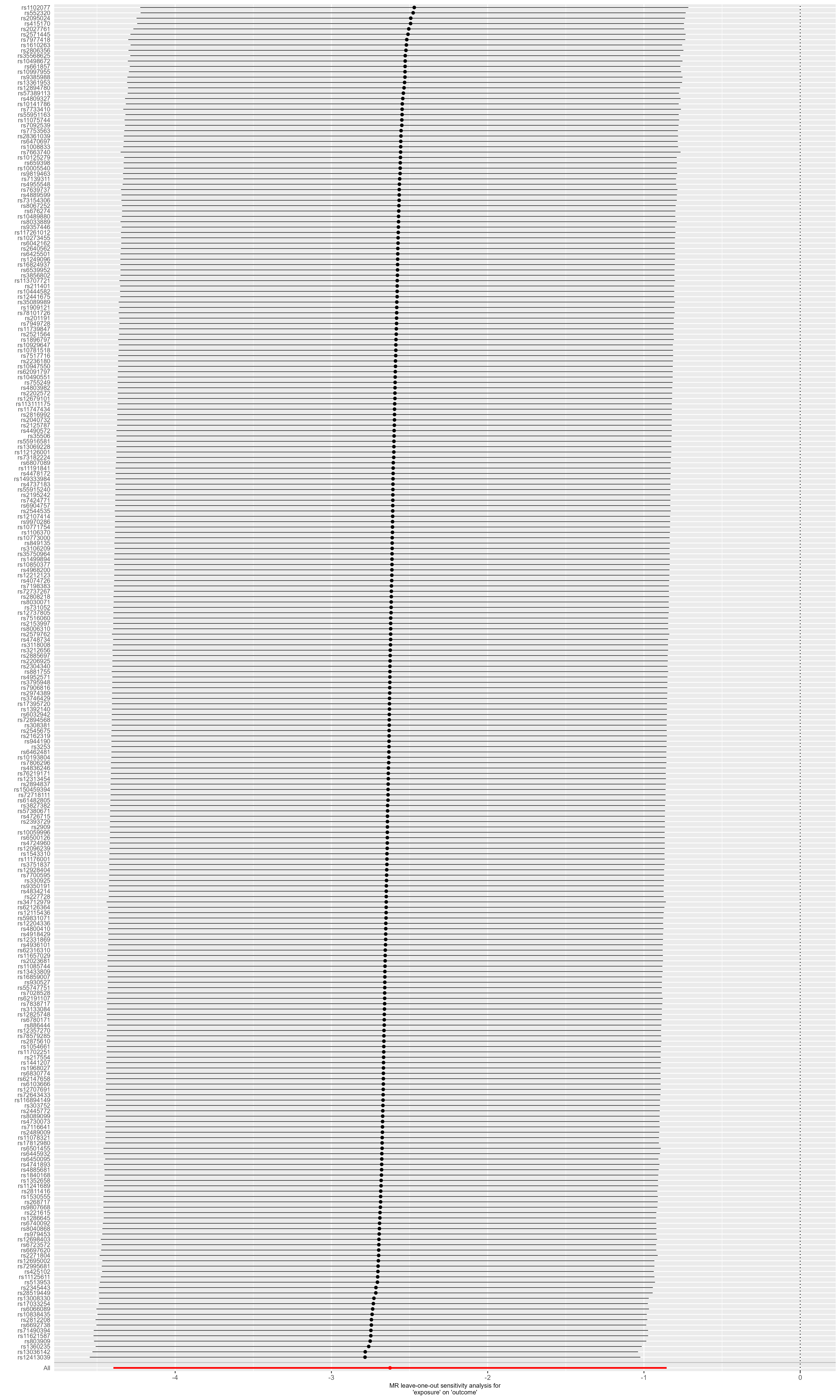

Supplement: Supplementary file 12 — Supplementary Material 12. [file 12890_2024_3150_MOESM12_ESM.zip › Supplementary Figure/leave-one-out analysis/Cortex Surface area/LOOA_FEV1_frontalpole_surfavg.png]

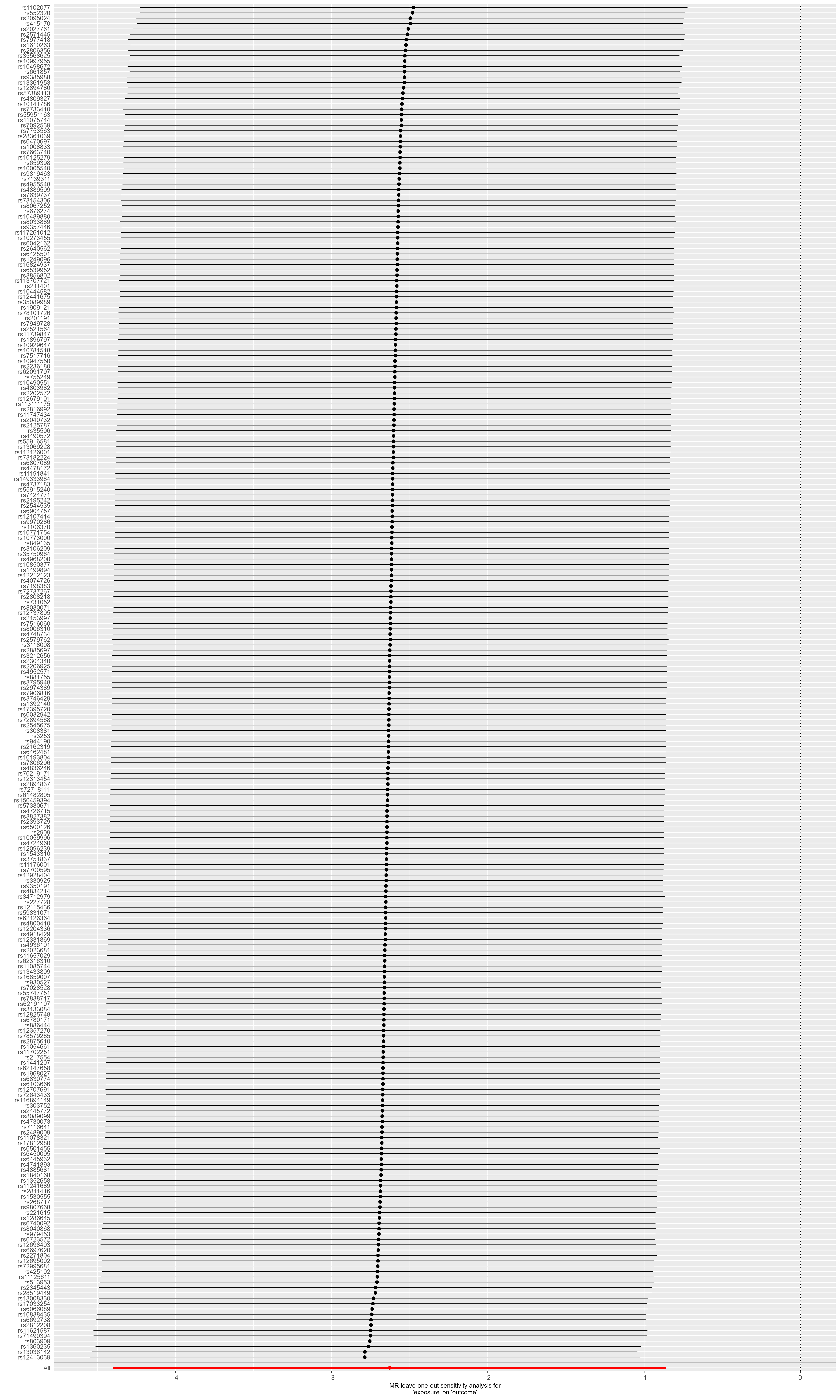

Supplement: Supplementary file 12 — Supplementary Material 12. [file 12890_2024_3150_MOESM12_ESM.zip › Supplementary Figure/leave-one-out analysis/Cortex Surface area/LOOA_FEV1_frontalpole_surfavg_noGC.png]

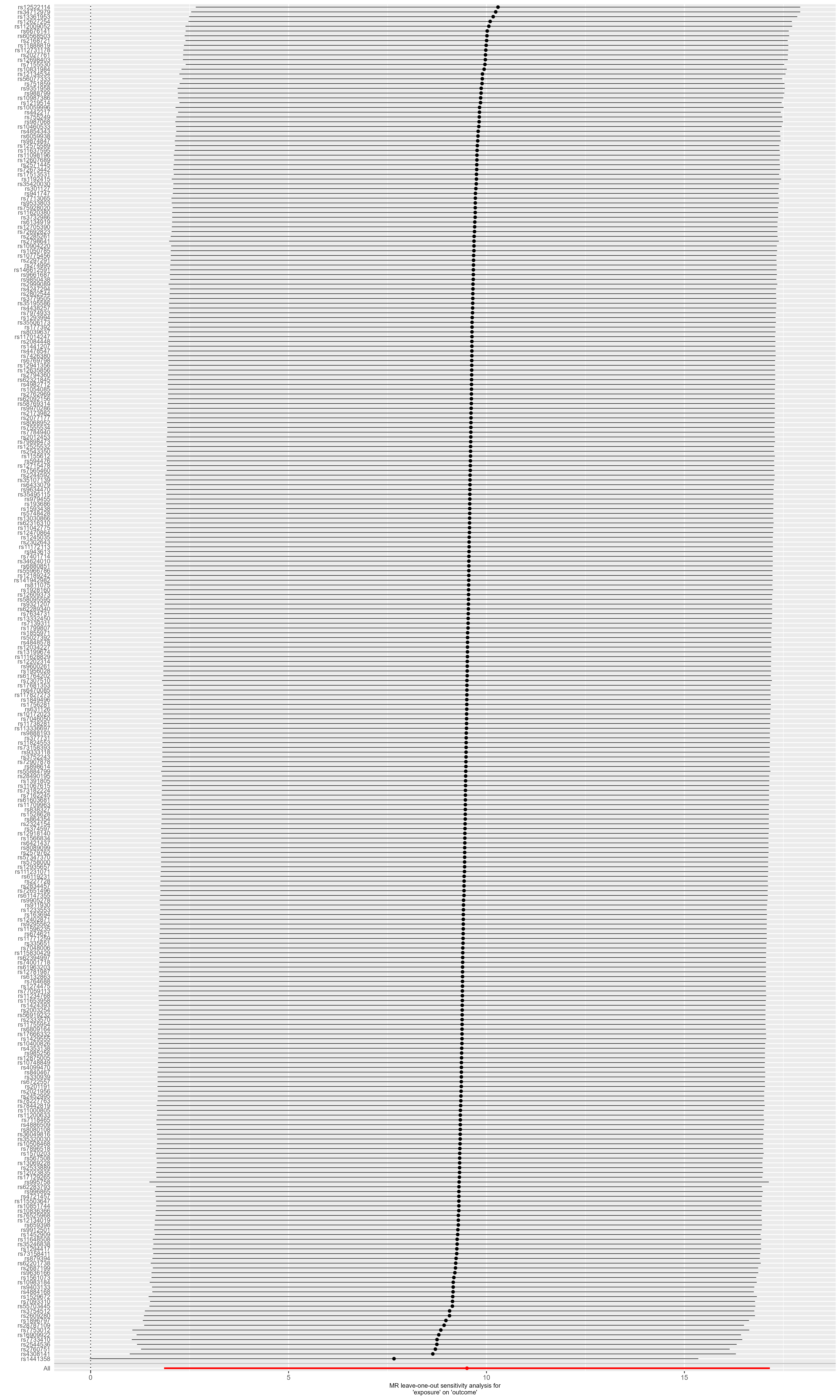

Supplement: Supplementary file 12 — Supplementary Material 12. [file 12890_2024_3150_MOESM12_ESM.zip › Supplementary Figure/leave-one-out analysis/Cortex Surface area/LOOA_FEV1_FVC_paracentral_surfavg.png]

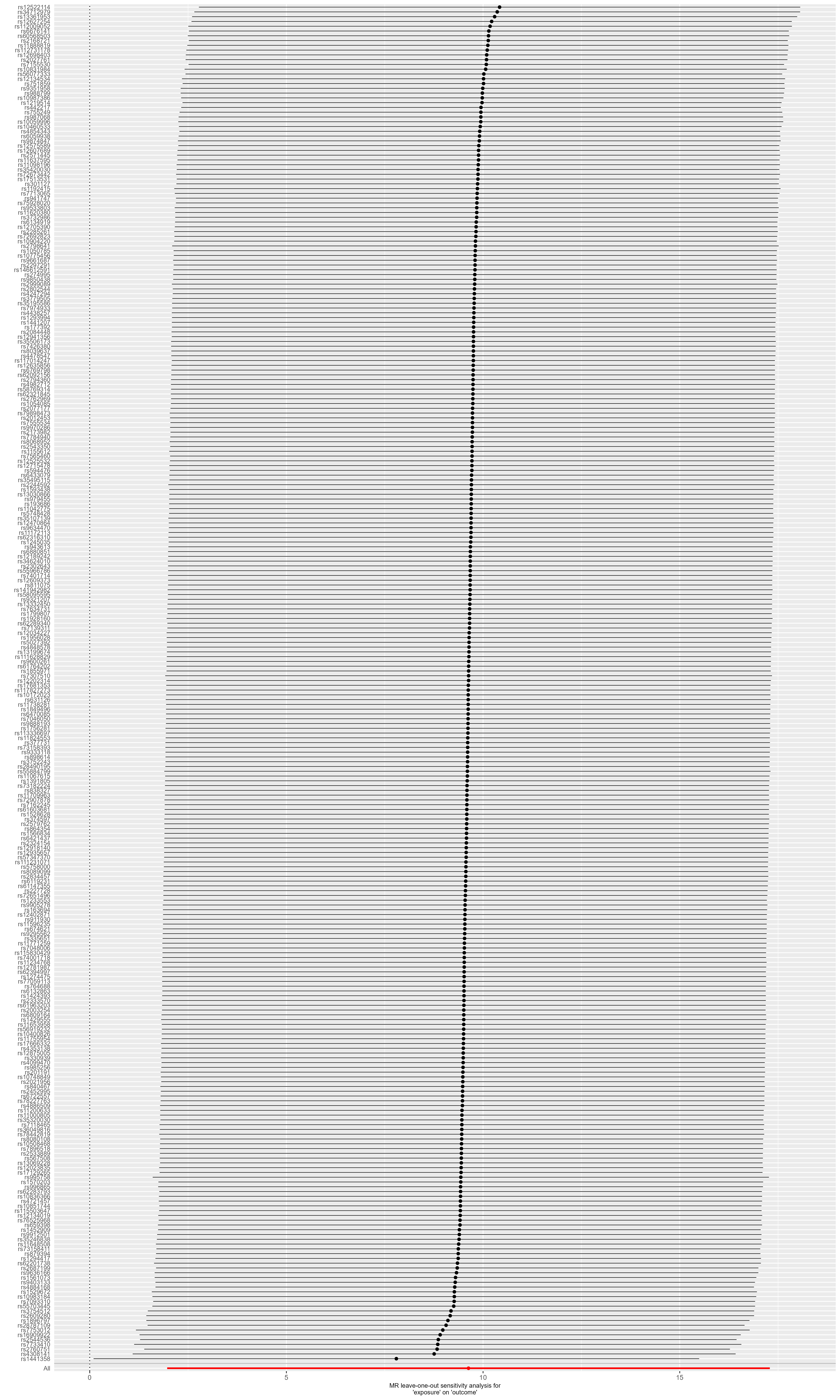

Supplement: Supplementary file 12 — Supplementary Material 12. [file 12890_2024_3150_MOESM12_ESM.zip › Supplementary Figure/leave-one-out analysis/Cortex Surface area/LOOA_FEV1_FVC_paracentral_surfavg_noGC.png]

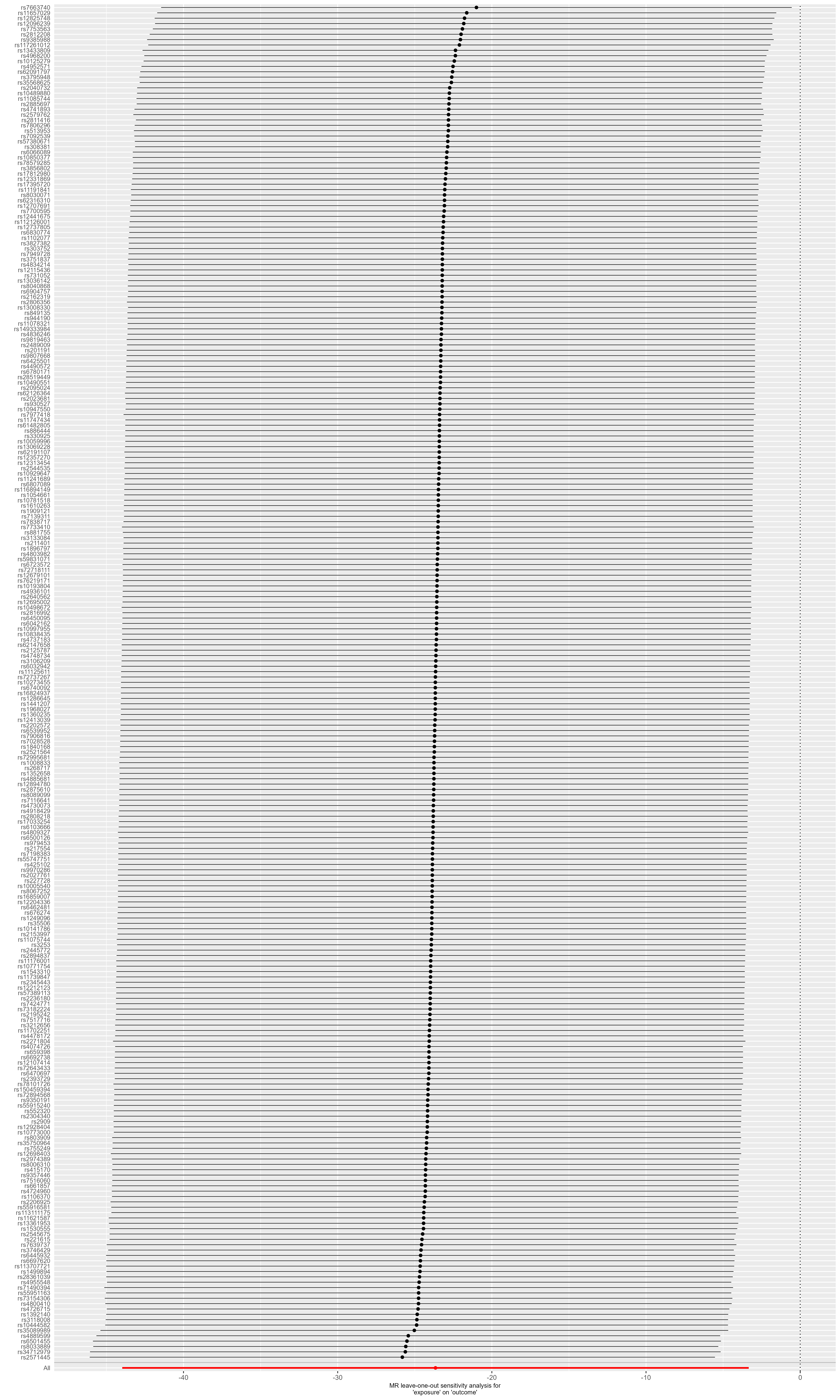

Supplement: Supplementary file 12 — Supplementary Material 12. [file 12890_2024_3150_MOESM12_ESM.zip › Supplementary Figure/leave-one-out analysis/Cortex Surface area/LOOA_FEV1_lingual_surfavg.png]

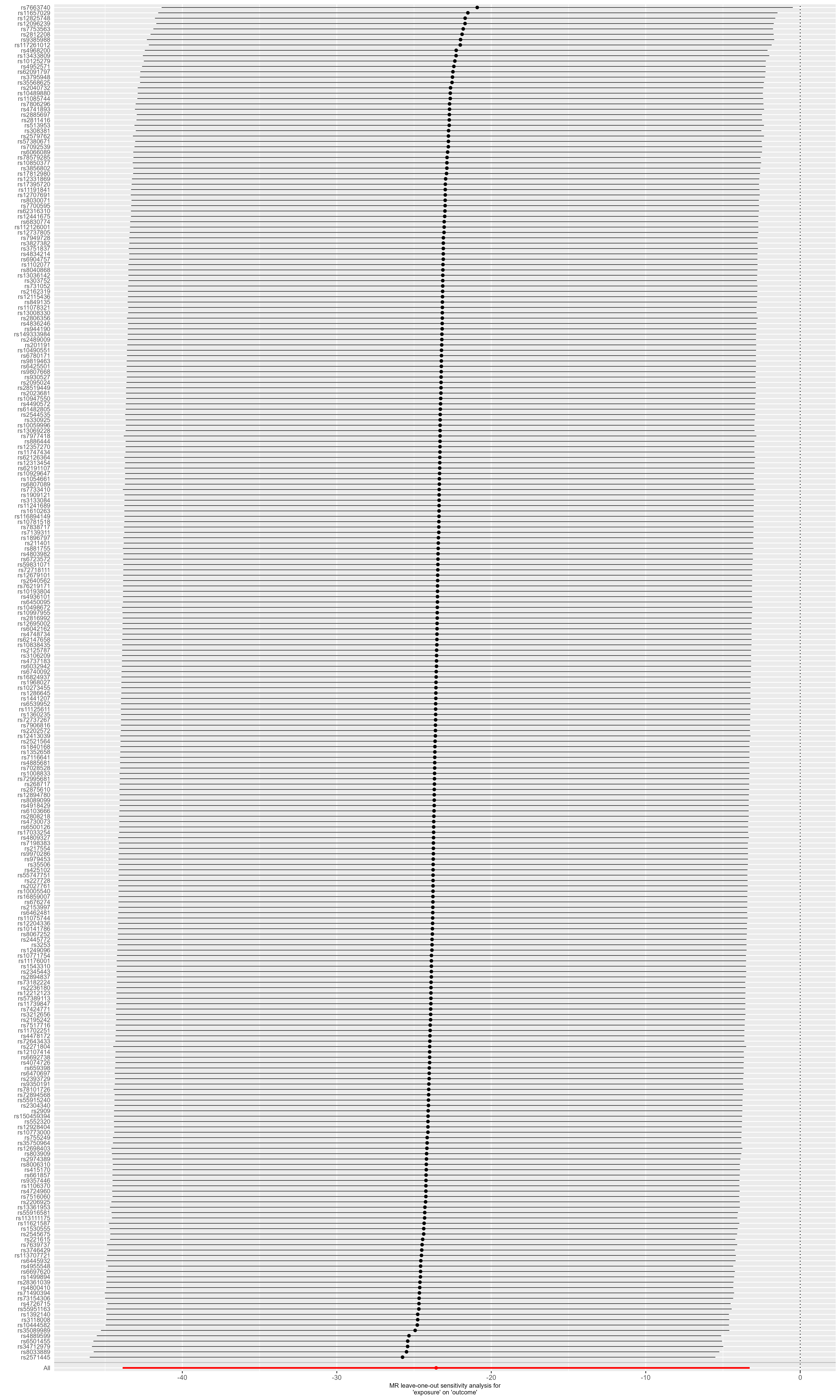

Supplement: Supplementary file 12 — Supplementary Material 12. [file 12890_2024_3150_MOESM12_ESM.zip › Supplementary Figure/leave-one-out analysis/Cortex Surface area/LOOA_FEV1_lingual_surfavg_noGC.png]
